# Photocatalytic C–F alkylation; facile access to multifluorinated arenes†
†Electronic supplementary information (ESI) available. See DOI: 10.1039/c5sc03013g


**DOI:** 10.1039/c5sc03013g

**Published:** 2015-09-29

**Authors:** A. Singh, J. J. Kubik, J. D. Weaver

**Affiliations:** a 107 Physical Science , Department of Chemistry , Oklahoma State University , USA . Email: Jimmie.Weaver@Okstate.Edu

## Abstract

C–F functionalizations that provide C–C bonds are challenging synthetic transformations, due in part to the large C–F bond strength, short bond length, nonpolarizable nature, the production of fluoride, and the regioselectivity-in the case of multifluorinated substrates. However, commercially available highly fluorinated arenes possess great synthetic potential because they already possess the C–F bonds in the desired locations that would be difficult to selectively fluorinate. In order to take advantage of this potential, selective C–F functionalizations must be developed. Herein, we disclose conditions for the photocatalytic reductive alkylation of highly fluorinated arenes with ubiquitous and unactivated alkenes. The mild reaction conditions provide for a broad functional group scope, and the reaction is remarkably efficient using just 0.25 mol% catalyst. Finally, we demonstrate the utility of the strategy by converting highly fluorinated arenes to elaborate (hetero)arenes that contain 2–5 C_aryl_–F bonds *via* synergistic use of photocatalysis and S_N_Ar chemistry.

## Abstract 2

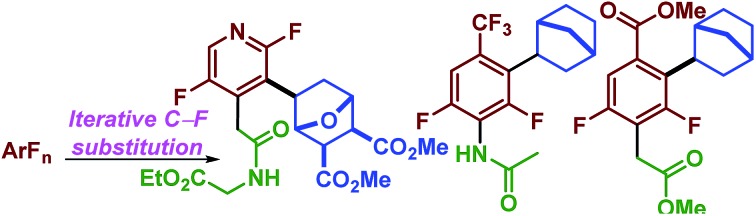
From the top down; access to complex multifluorinated arenes *via* multiple photocatalytic C–F substitutions.

## Introduction

Partially fluorinated arenes make up an extremely important class of molecules within the pharmaceutical^1^ and crop science^2^ industries. In 2013, the FDA approved 27 new small molecule entities.^3^ Of these, 9 contain C–F bonds and 4 (Adempas, Gilotrif, Tafinlar, Tivicay) contain aryl C–F bonds. Aryl fluorides share a similar importance in the crop protection sciences^4^ with over 5.6 billion pounds of pesticides produced annually^5^ with important aromatic fluorides such as Tefluthrin, Lufenuron, Clodinafop and Saflufenacil.^6^

**Scheme 1:**
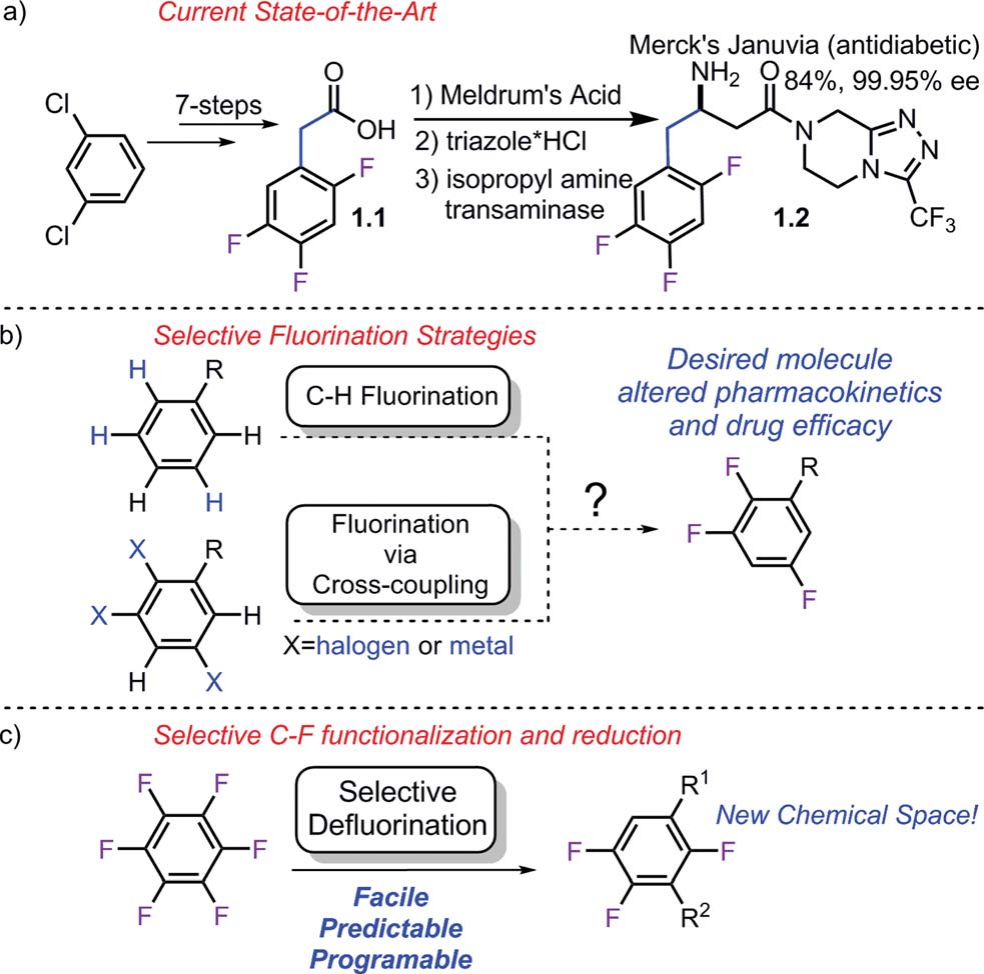
Synthesis of Januvia's fluorinated precursor and comparison of strategies. a) currently employed strategy, b) potential selective fluorination approaches, c) proposed strategy.

Despite their simple appearance, synthesis of partially fluorinated molecules such as **1.1** (Scheme 1a) typically rely on classic but lengthy fluorination strategies. Synthesis of acid **1.1** involves the commonly employed nitration, halex, reduction, Balz–Schiemann sequence. While the requisite acid (**1.1**) used by Merck in the synthesis of Januvia is commercially available, it requires seven steps to synthesize and highlights the need for the development of new strategies to access multifluorinated arenes. More recently, much effort has been focused on developing modern fluorination strategies to facilitate discovery efforts (Scheme 1b). C–H fluorination^7^ strategies as well as more traditional cross-coupling strategies using both nucleophilic^8^ and electrophilic^9^ fluorine have recently been published. Importantly, while these methods have provided numerous methods to conduct selective fluorination, they are not well suited to provide access to multi-fluorinated arenes, since this would require the synthesis of unduly complicated starting materials.

An alternative approach to multifluorinated arenes, which has been championed by others,^10^ is to use highly or perfluorinated arenes, which provides the carbon framework with the desired fluorine atoms already in place. The undesired fluorine atoms can then serve as functional group handles to transform the molecule into the desired product. Thus, they can effectively serve as synthetic linchpins and provide access to fluorinated molecules not previously possible or potentially shorten the synthetic sequence to important molecules such as **1.1**. Importantly, perfluoroarenes are readily available by exhaustive fluorination or the halex process^11^ and many are commercially available.

While at the outset, this strategy sounds conceptually straightforward, there are several significant challenges associated with this approach that have prevented it from being implemented. Namely, C–F bonds are remarkably strong,^12^ short,^13^ and not significantly polarizable^14^-making them both kinetically and thermodynamically robust. Furthermore, even if the bond is broken and fluoride formed, it often forms strong metal–F bonds which can lead to sluggish catalyst turnover, making metal-catalyzed processes difficult.^15^ Finally, if all these issues are circumvented, the regioselectivity of the C–F functionalization could still be problematic given that the arene contains multiple C–F bonds.

Nonetheless, some progress has been made in the area of selective C–F alkylation of highly fluorinated arenes.10b,10c,^16^ For instance, Li has demonstrated that magnesiates are sufficiently nucleophilic to add uncatalyzed to perfluorinated arenes.^17^ The Love group has developed a benzyl imine directed Pt-catalyzed alkylation reaction.16f,16g,16i More recently, Wu has shown that phosphonium ylides are capable of undergoing C–F substitution.^18^ These methods are important examples of selective C–F functionalization. However, there is still a pressing need for the development of novel strategies to alkylate C–F bonds in order to achieve the full synthetic potential of highly fluorinated arenes. Therefore, reactions that take place *via* mechanistically distinct pathways and also provide access to complimentary products are of significant value.

Others have shown that photocatalysis can be used to reduce benzylic bromides,^19^ α-bromo-malonates, –ketones^20^ –chalcones,^21^ aryl-iodides and even a few activated aryl bromides.^22^ In 2014, we demonstrated that visible light photocatalysis could be used to perform selective hydrodefluorination of perfluoroarenes.^23^ This method proved ideal for avoiding many of the challenges facing metal-catalyzed methods for C–F functionalization. Specifically, the catalyst, *fac*-Ir(ppy)_3_, is an 18-electron complex that is tris-cyclometalated and coordinatively saturated and provides little opportunity to form problematic M–F bonds. The reaction was postulated to have taken place by an electron transfer to the arene which typically resulted in a selective C–F bond fragmentation event. Furthermore, the method was mild and functional group tolerant. Thus, we were eager to see if the intermediate could be utilized to form a C–C bond (eqn 3, Scheme 1) which would help us realize our larger goal of making perfluoroarenes a useful synthetic linchpin by providing complimentary reactivity to classic S_N_Ar chemistry common to (hetero)aryl fluorides. We chose to initiate our investigation with alkenes, which we hoped could serve as a surrogate for an alkyl group by *in situ* reduction. From a synthetic viewpoint, the use of alkenes was attractive when compared to other sp^3^-hybridized coupling partners because they would require no prefunctionalization and, between natural compounds and synthetic methods, would provide enormous substrate variety. Herein, we demonstrate the first photocatalytic C–F reductive alkylation and show that when used synergistically with photocatalytic C–F reduction, and S_N_Ar chemistry it is capable of rapidly delivering remarkably complex multifluorinated arenes.

## Results and discussion

Based on our previous work,^23^ we expect the reaction to take place *via* electron transfer from the photocatalyst to the perfluoroarene,^24^ which results in C–F fragmentation and generates a fluoride^25^ and a perfluoroaryl radical. We hoped that this radical could be enticed to undergo C–C bond formation with an alkene rather than H-atom transfer. It is worth noting that most systems used for the generation of aryl radicals typically begin with aryl iodides and bromides, are subject to rapid secondary reduction to give an aryl anion. Consequently, these reactions are typically limited to fast cyclizations^26^ which can compete with reduction.^27^ We hoped that the undesirable over-reduction could be avoided by using an amine reduction system, which would allow the generation of a polyfluoroaryl radical that could undergo productive C–C formation to give an alkyl radical. Finally, the alkyl radical would need to undergo a controlled H-atom abstraction with the amine radical cation, or the amine,^28^ to provide the product without undergoing undesired radical reactions such as dimerization or oxidation/substitution. Thus, we set about developing the reductive alkylation of perfluorinated arenes with alkenes.

We began the optimization using pentafluoropyridine and cyclohexene (Table 1, entry 1). Starting with conditions that facilitated the photocatalytic hydrodefluorination^23^ (HDF) we screened solvents (entry 3–5). When toluene, THF, or DCM were used as the reaction solvent, no reaction occurred (entry 3). The use of nitromethane or dimethylformamide led to unidentified products. While use of DMSO led to product formation, it gave a decrease in the relative amount of alkylated (**1a**) to HDF product (**1a′**).

**Table 1:** Optimization of reaction conditions

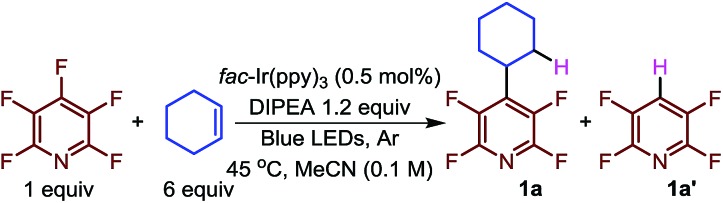				
Entry	Modifications	Conv.^*a*^	**1a**/**1a′**	Time
1	None	100%	2.6	8 h
2	No Ir(ppy)_3_, DIPEA, or light	0%	na	8 h
3	THF, DCM, or toluene instead of MeCN	0%	na	8 h
4	MeNO_2_, DMF instead of MeCN	100%	na^*b*^	2 d
5	DMSO instead of MeCN	99%	1.7	8 h
6	1.2 equiv. cyclohexene	73%	0.8	2.2 h
7	2.4 equiv. cyclohexene	57%	1.5	2.2 h
8	3.6 equiv. cyclohexene	59%	1.8	2.2 h
9	4.8 equiv. cyclohexene	58%	2.1	2.2 h
10	6.0 equiv. cyclohexene	68%	1.8	2.2 h
11	10.0 equiv. cyclohexene	39%	2.5	2.2 h
12	Ir(ppy)_3_, (0.5 mol%)	72%	3.2	5.5 h
13	Ir(ppy)_3_, (0.25 mol%)	73%	2.8	5.5 h
14	Ir(ppy)_3_, (0.125 mol%)	35%	2.9	5.5 h

^*a*^Determined by ^19^F NMR.

^*b*^Unidentified products formed.

We next evaluated the effect of the concentration of the alkene on the reaction outcome (entries 6–11). At low alkene concentration the reaction proceeded noticeably faster, but gave greater amounts of the HDF product. While high alkene concentration (10 equivalents, entry 11) gave a moderately greater product ratio, it proceeded more slowly. We ultimately chose 6 equivalents of alkene as a compromise between the two extremes. Additionally, we investigated the concentration of the photocatalyst (entries 12–14). We found that the loading could be dropped to 0.25 mol% before any effect on the yield was observed.

**Fig. 1:**
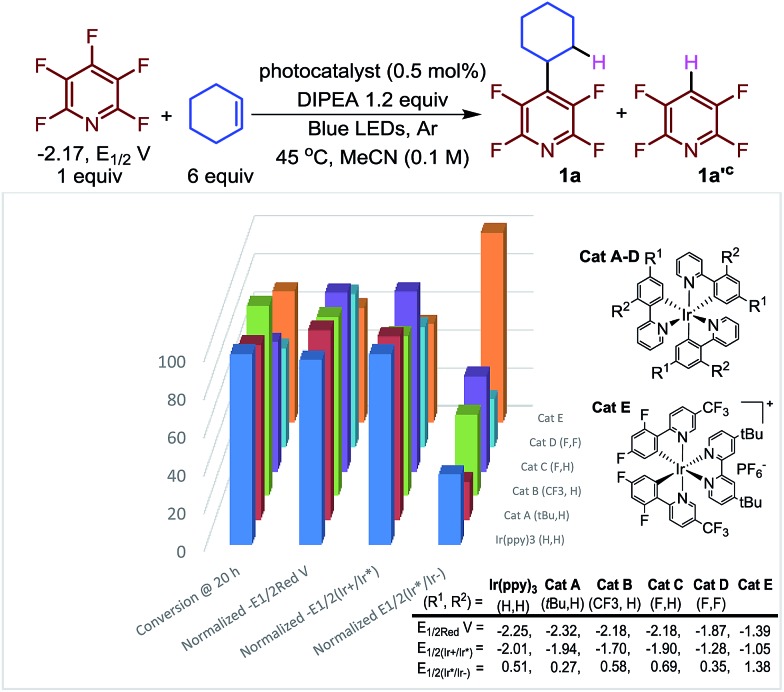
Conversion^a^ as a function of catalyst normalized potential.^b^ (a) Determined by ^19^F NMR. (b) All potentials are given in V *versus* the Standard Calomel Electrode (SCE). The *Y*-axis shows the relative reducing or oxidizing potentials of the catalysts. (c) The ratio of **1a**/**1a′** varied only slightly 2.2–3.2.^29^

Next, we investigated the nature of the catalyst on both the selectivity and conversion of the reaction (Fig. 1). The catalyst we investigated ranged from strongly reducing excited states (*E*_1/2(Ir^+^/Ir*)_, lane 3, Fig. 1), to strongly oxidizing excited states (*E*_1/2(Ir*/Ir^–^)_, lane 4). This makes them more prone to undergo oxidative and reductive quenching of the photocatalyst, respectively.

Previously, we proposed that the photocatalytic HDF took place *via* reductive quenching to give the reduced Ir(ii) species. It is from the reduced catalyst, Ir(ppy)_3_^–^, that electron transfer is thermodynamically favourable. Interestingly, while the relative rates varied, the reaction proceeded with all the photocatalysts despite large differences in potentials. Only small changes to the product distribution (**1a**/**1a′**) were observed which is consistent with a C–C bond forming event which is independent of the catalyst.

In contrast to our previous suggestion,^23^ careful inspection of these results indicate that electron transfer to the perfluoroarene can mostly likely take place *via* either reductive or oxidative quenching cycles or potentially both simultaneously. For instance, Ir(ppy)_3_ and **Cat A** could proceed through an oxidative quenching path,^30^ despite a slight underpotential (C_5_F_5_N –2.17 *vs.* Ir(ppy)_3_ – 2.01 and **Cat A** –1.94, *E*_1/2_*vs.* SCE).^31^ While a reductive quenching of these photocatalysts by the amine is possible (EtNiPr_2_ + 0.68 *vs.* 0.51 (Ir(ppy)_3_), 0.27 (**Cat A**), *E*_1/2_*vs.* SCE)^32^ this process is even more endothermic. The photoexcited state of **Cat B** is less reducing (–1.70 V *vs.* SCE) and less likely to undergo reductive quenching but is more oxidizing (0.58 V *vs.* SCE) and still undergoes efficient coupling. Furthermore, the reduced **Cat B^–^** is a strong reductant (–2.18 V *vs.* SCE), suggesting that when **Cat B** is used, a reductive quenching event followed by electron transfer to the pentafluoropyridine is the dominant path (Table 2).

**Table 2:** Scope of the C–F reductive alkylation^*a*^

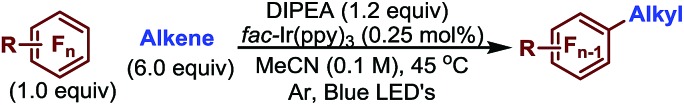
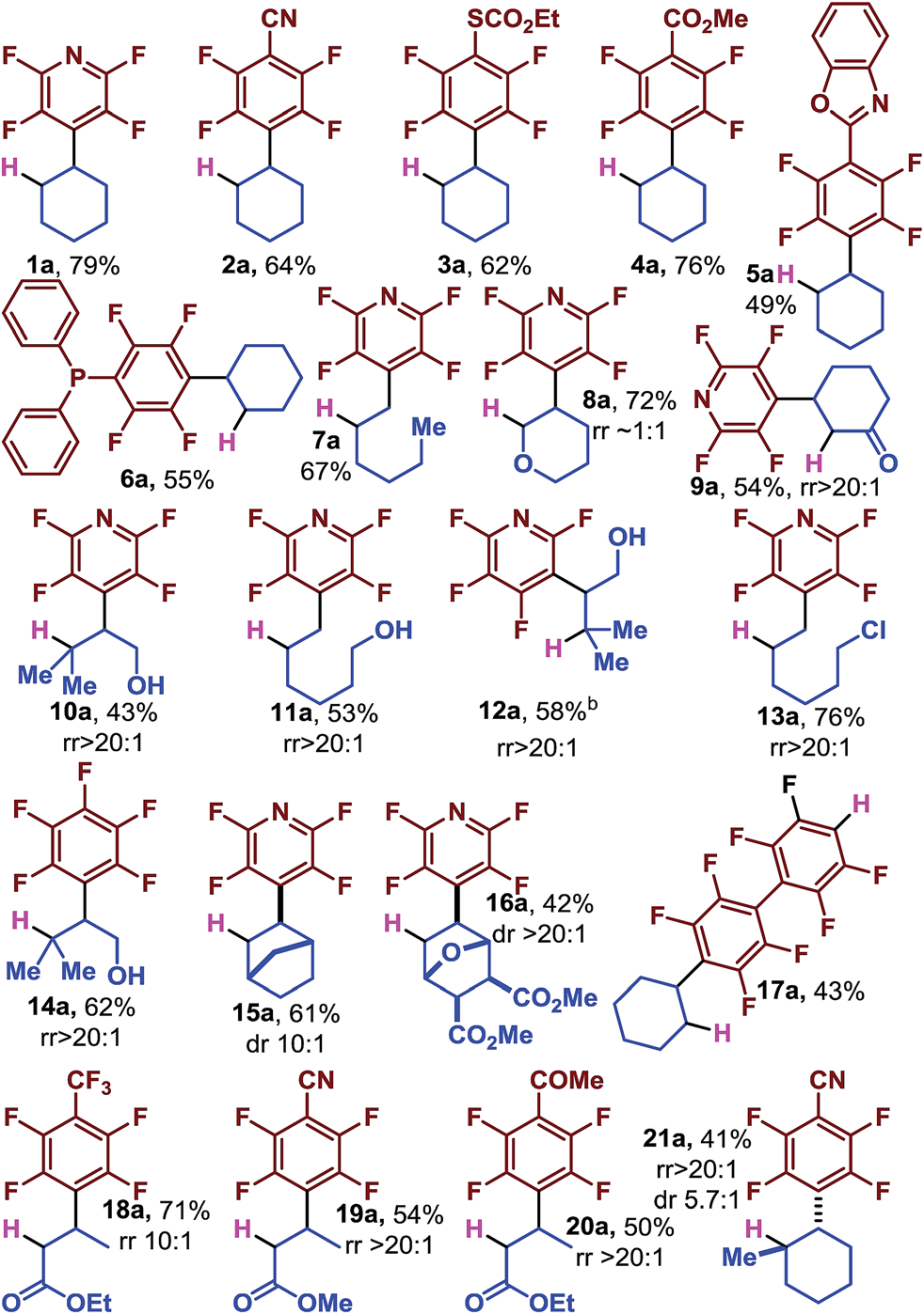

^*a*^Yields correspond to isolated product. The regioisomeric ratio (rr) and diastereomeric ratio (dr) with respect to the alkene were determined by ^1^H NMR of the crude reaction mixture after workup.

Interestingly, **Cat C**, which could engage in a near thermoneutral electron transfer by undergoing reductive quenching and then electron transfer, actually performs more modestly than **Cat B**. While **Cat D** and **Cat E** for which reductive quenching is almost certainly the primary mechanistic path, **Cat E** outperforms **Cat D** despite an even more substantial underpotential. This may be a result of the cationic nature of **Cat E** which may electrostatically stabilize the incipient radical anion formed from pentafluoropyridine.^33^ Further complicating the picture, Scaiano has demonstrated that if α-amino radicals are generated they too can also serve as reductants, in some instances.^28^ Given the relatively small difference observed in the best performing photocatalysts, we opted to use the more common Ir(ppy)_3_ catalyst. Thus, we began to explore the scope of the reaction using *N*,*N*-diisopropylethylamine (DIPEA) (1.2 equiv.), alkene (6.0 equiv.), Ir(ppy)_3_ (0.25 mol%) in MeCN (0.1 M) with visible light irradiation with blue LED strips.

Initially, we looked at a series of perfluoroarenes with cyclohexene to evaluate the generality of the scope with regard to arene. We found that moderate to good yields could be obtained directly from a number of perfluoroarenes including pyridine (**1a**), benzonitrile (**2a**), thiocarbonate (**3a**), esters (**4a**), heterocyclic substituted (**5a**), and even phosphine (**6a**). We were concerned that the intermediate radical might be too reactive to be selective, but we found that differences in the substitution pattern of the alkenes led to excellent selectivity, with addition occurring at the less substituted carbon (**7a**, **10a**, **11a**, **12a** and **13a**). The reaction proceeds smoothly in the presence of electron rich dihydropyran (**8a**) as well as with electron poor cyclohexenone (**9a**) which gives a single regioisomer. **12a** is derived from 3-chloro tetrafluoropyridine and demonstrates the orthogonal reactivity of the photocatalytic functionalization and S_N_Ar chemistry which is selective for the 4-F in the perfluoropyridine system. **12a** is also noteworthy because no 5-*exo*-trig cyclization product (a benzofuran derivative) is detected and highlights the mild reaction conditions. Likewise, the primary alkyl chloride is well tolerated (**13a**).^34^

Complex alkenes are available from synthetic methods and nature and we were eager to see if they could be used to easily incorporate alkyl groups that were not only functionally, but also stereochemically dense. We found norbornene to be an excellent substrate (**15a**) as well as the furan derived [4 + 2] adduct (**16a**) which contains several sensitive functional groups and base labile stereocenters.

Reaction with acrylates provides the saturated β-fluoroarylated esters (**18a–20a**). Reaction with methyl cyclohexene provides the *trans*-1,2-disubstituted cyclohexane with moderate stereoinduction (**21a**, 5.7 : 1 dr). Decafluorobiphenyl undergoes a rapid second photocatalytic C–F fragmentation event and allows facile access to the tandem reductive alkylation and HDF product (**17a**) which can be further elaborated by traditional methods that utilize the enhanced acidity of the Ar–H.^35^

We expected that the highly fluorinated nature of the arenes would facilitate elaboration of the structure by S_N_Ar reactions. However, it was not known how well the photocatalytic reaction would work after the fluorines which also serve to activate the arene towards electron addition were removed^36^ (*E*_1/2 red_ C_6_H_6_ = –3.42 *vs.* SCE^37^ and *E*_1/2 red_ C_6_F_6_ = –2.81 *vs.* SCE^38^). In other words, we were concerned that substitution of the fluorines would make the photochemical reactions too sluggish to be useful. Nonetheless, we next began to look at substrates in which at least one fluorine had already been used to synthesize the desired substrate (Table 3). When the acetophenone derived benzofuran was exposed to cyclohexene we obtained the trifluorinated, alkyl substituted **22a** in good yield.

**Table 3:** Trifluorinated arenes

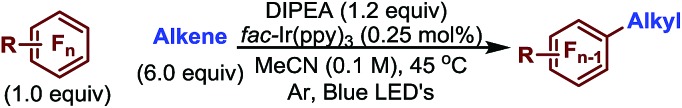
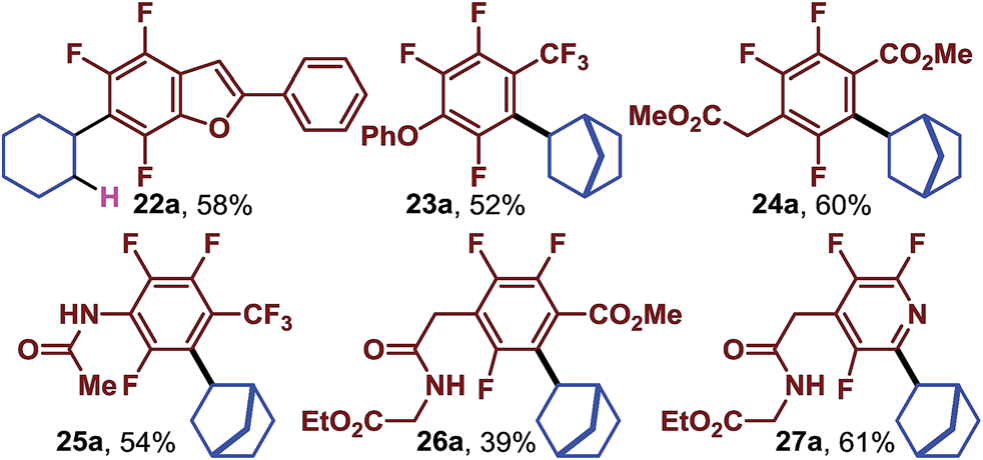

Trifluoroarenes (**23a**, **25a**), (**24a**, **26a**) and **27a** are derived from octafluorotoluene, methylpentafluorobenzoate and pentafluoropyridine, respectively. When the 4-position is substituted, the substrates undergo photocatalytic reductive alkylation at the position *ortho* to the trifluoromethyl group, ester group, or nitrogen atom. When performed together these two substitution manifolds provide rapid access to complex multi-fluorinated arenes which contain diverse functional groups such as aryl ethers, esters, amides and even amino acid derivatives.

By taking advantage of the orthogonal nature of S_N_Ar and the photochemical functionalization, access to isomeric fluoroarenes is possible.

**Scheme 2:**
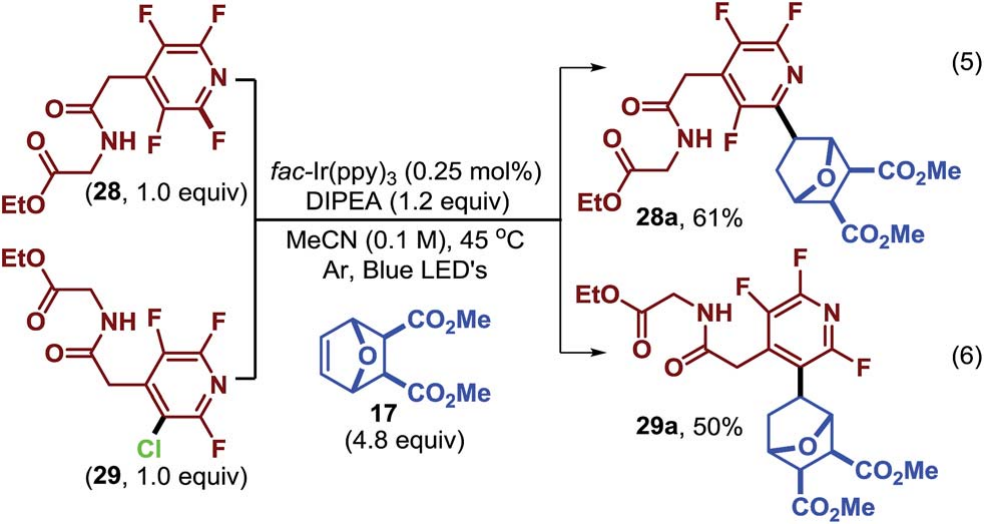
Exploiting fragmentation selectivity to access isomers.

The normal regioselectivity of the photocatalytic C–F fragmentation event can be overridden by utilizing the monochlorinated starting material which results in perfectly selective C–Cl fragmentation. For example, subjecting fluoroarenes **28** and **29** (ref. 39) to photocatalytic conditions we were able to isolate complimentary regioisomers (**28a** and **29a**, Scheme 2) in reasonable yields. In this case, the C–Cl bond is fragmented preferentially to the C–F bond. This highlights the synthetic versatility and predictability of this approach.

**Scheme 3:**
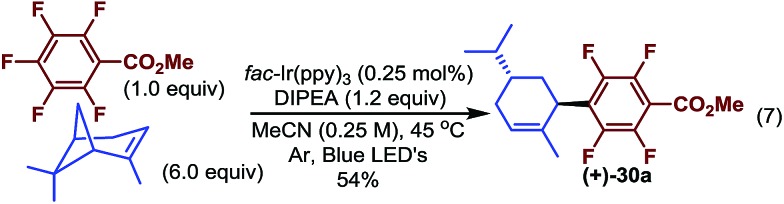
Tandem addition and ring opening.

When vinyl cyclobutane α-pinene was used, an optically active ring-opened trisubstituted cyclohexene was obtained rather than the standard reductively alkylated product (**30a**, Scheme 3). This likely results from perfluoroaryl radical addition to the less substituted terminus of the alkene followed by ring-opening and finally reduction exocyclic radical suggesting that the ring-opening event occurs faster than the reduction of the tertiary radical (under these conditions).^40^ Furthermore, the highly regio- and diastereoselective addition product may provide a convenient method for accessing more complex optically active molecules which contain fluoroaryl groups.

**Scheme 4:**
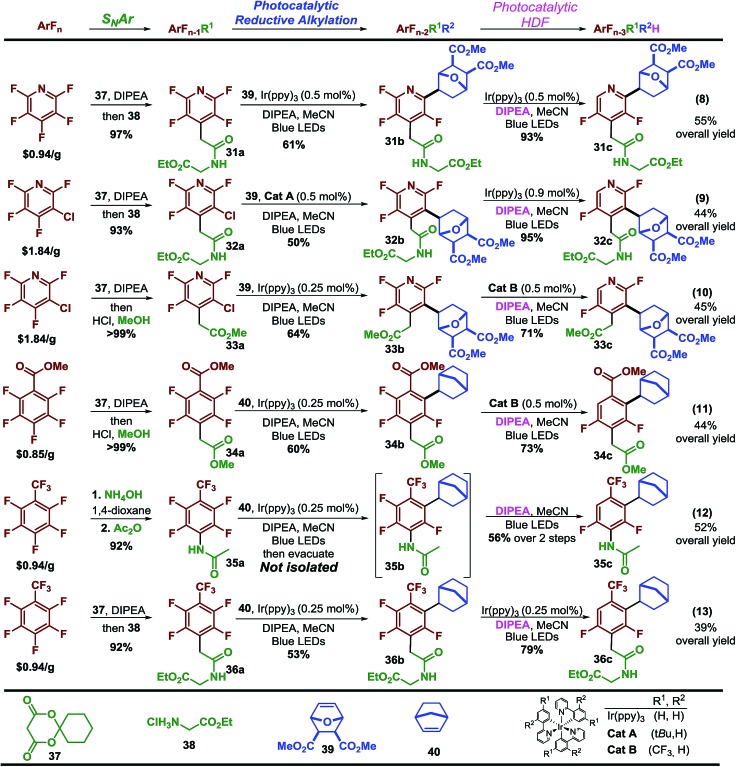
Perfluoroarenes as synthetic linchpins.

Finally, having explored the scope of the reaction we sought to demonstrate that perfluoroarenes possess significant synthetic potential to provide both access to multi-fluorinated arenes and simultaneously serve as synthetic linchpins that allow a high level of structural complexity to be built directly from the core of the fluorinated molecule-a strategy that has been, to date, under utilized. To demonstrate this strategy, we employed a simple and general sequence of S_N_Ar and elaboration, followed by photocatalytic reductive alkylation and finally photocatalytic hydrodefluorination (HDF). This sequence provides access to a diverse and elaborate set of multi-substituted, difluorinated arenes and heteroarenes in reasonable yields, 39%–55% with excellent regiocontrol and fluorine content control starting from just a few inexpensive polyfluorinated arenes (Scheme 4).^41^

For instance, starting with pentafluoropyridine (eqn 8, Scheme 4) the Meldrum's acid adduct^42^ is formed and then decomposed in the presence of the glycine ethyl ester·HCl salt to afford the amide in high yield. The tetrafluoropyridine product is then sequentially subjected to photocatalytic reductive alkylation and HDF to arrive at **31a** in 55% overall yield. Using the 3-chlorotetrafluoropyridine analogue allows synthesis of the regioisomer (**32a**) in 44% yield. The ability to easily access both isomers could potentially facilitate structural activity relationship studies. The commercially available Meldrum's acid adducts^29^ are a versatile functional group handle that can quickly provide other motifs such as esters, which can undergo similar sequences (**33a**). This sequence can be extended to other perfluoroarenes such as methyl pentafluorobenzoate (eqn 11) and octafluorotoluene (eqn 12 and 13). Synthesis of **35a** required only 0.25 mol% Ir(ppy)_3_ to carry out both photocatalytic steps. At the completion of the alkylation step, the excess alkene was removed from the reaction mixture by vacuum. Upon addition of solvent, amine, light, and degassing, the photocatalytic-HDF reaction began. In some cases a purification step is required to remove impurities before progressing, in spite of this substantial amounts of material (**36a**) can be obtained in this short synthetic sequence.

## Conclusions

In conclusion, we have demonstrated the first example of photocatalytic C–F alkylation. The reaction utilizes inexpensive perfluoroarenes and ubiquitous alkenes without any need for functional group interconversions. Furthermore, the reaction takes place with low catalyst loadings, utilizes simple or complex alkenes, and displays excellent functional group tolerance. Importantly, it suggests that photocatalytic electron transfer can serve as convenient method for generating fluoroaryl radicals directly from the C–F bonds and that these highly reactive radicals^43^ can be harnessed for cross-coupling reactions. We have demonstrated that photocatalytic C–F functionalization, when used synergistically with S_N_Ar chemistry, can lead to elaborate molecules that would be difficult, if not impossible, to synthesize. We expect this chemistry should substantially aid the discovery processes given the current unmet need for multi-fluorinated arenes and the rapid and flexible nature of this chemistry.

## Conflict of interest

The authors declare a competing financial interest in that they hold a patent United States Serial no. 62/043,650 concerning the structure and method for the Meldrum's acid adducts.

## Supplementary Material

Supplementary information
