# Supplementary material for: Photocatalytic C–F alkylation; facile access to multifluorinated arenes
Source: Chem Sci. 2015 Sep 29;6(12):7206–12. doi: 10.1039/c5sc03013g (PMC5947535; doi:10.1039/c5sc03013g)

Electronic Supplementary Material (ESI) for Chemical Science.
This journal is © The Royal Society of Chemistry 2015

Phtocatalytic C–F Alkylation; Facile Access to Multifluorinated Arenes

Anuradha Singh, Jacklyn Kubik, and Jimmie D. Weaver*
Department of Chemistry, Oklahoma State University, Stillwater, OK 74078
jimmie.weaver@okstate.edu

Supporting Information

Table of contents:-

General experimental S2

Synthesis of substrates S3

Photocatalytic reductive alkylation reactions and characterization S7

References S27

NMR spectra S29

General Experimental

All reagents were obtained from commercial suppliers (Sigma-Aldrich, Oakwood chemicals, Alfa Aesar, Matrix Scientific, VWR) and used without further purification unless otherwise noted. Acetonitrile (CH_3CN) was dried over molecular sieves. *N*,*N*-diisopropylethylamine was purchased from Sigma-Aldrich and was distilled and stored over anhydrous potassium hydroxide. Photocatalysts Ir(ppy)$_3$ *fac*-tris(2-phenyl pyridinato-C^2, *N*)iridium(III), Ir(tbppy)$_3$ *fac*-tris[2-(4-*tert*-butylphenyl)pyridinato-C^2, *N*)]iridium(III), Ir(CF_3ppy)$_3$ *fac*-tris[2-(4-trifluoromethylphenyl)pyridinato-C^2, *N*)]iridium(III), Ir(Fppy)$_3$, *fac*-tris[2-(4-fluorophenyl)pyridinato-C^2, *N*)]]iridium(III), Ir(dFppy)$_3$ *fac*-tris(2-(4,6-difluorophenyl)pyridinato- C^2, *N*)iridium(III), [Ir(dF(CF_3)ppy)$_2$(dtbbpy)](PF_6) [4,4'-Bis(tert-butyl)-2,2'-bipyridine]bis[3,5-difluoro-2-[5-(trifluoromethyl)-2-pyridinyl]phenyl]iridium(III) hexafluorophosphate were synthesized according to literature procedure.[1] Methyl 2,3,4,5,6-pentafluorobenzoate, 2-(perfluorophenyl)benzo[*d*]oxazole, 1,2,4,5-tetrafluoro-3-phenoxy-6-(trifluoromethyl)benzene, *O*-ethyl *S*-(perfluorophenyl) carbonothioate, *N*-(2,3,5,6-tetrafluoro-4-(trifluoromethyl)phenyl)acetamide, 4,5,6,7-tetrafluoro-2-phenylbenzofuran, methyl 2,3,5,6-tetrafluoro-4-(2-methoxy-2-oxoethyl)benzoate were synthesized according to literature procedures.[2,3] Reactions were monitored by ^{19}F NMR and GC-MS (QP 2010S, Shimadzu equipped with auto sampler). NMR spectra were obtained on a 400 MHz Bruker Avance III spectrometer or a 400 MHz Unity Inova spectrometer. ^{1}H and ^{13}C NMR chemical shifts are reported in ppm relative to the residual solvent peak while ^{19}F is set relative to an external standard. IR spectra were recorded on a Nicolet iS50 FT-IR. Melting points were determined on a Mel-Temp apparatus. Optical rotation was measured on Autopol V by Rudolph Research Analytical. High resolution mass spectra were obtained on LTQ-OrbitrapXL by Thermo Scientific ltd. Purifications were carried out using Teledyne Isco Combiflash Rf 200i flash chromatograph with Redisep Rf normal phase silica (4 g, 12 g, 24 g, 40 g, or 80 g) as well as reverse phase C18 (26 g) column with product detection at 254, 280 nm and by ELSD (evaporative light scattering detector). Some isolations were performed using Sorbent Technology Silica Prep TLC Plates, w/UV254, glass backed, 1000 µm, 20 x 20 cm, and were visualized with ultraviolet light. Substrate synthesis reactions were monitored by thin layer chromatography (TLC), obtained from Sorbent Technology Silica XHL TLC Plates, w/UV254, glass backed, 250 µm, and were visualized with ultraviolet light or potassium permanganate.

Photocatalytic Reaction Set up

Photocatalytic reactions were set up in a light bath as described below. Strips of blue LED's (18 LED's/ft.) were purchased from Solid Apollo. The strips (4.9 ft) were wrapped around on the walls of glass crystallization dish and secured with masking tape and then wrapped with aluminum foil. A lid which rest on the top was fashioned from cardboard and holes were made such that NMR tubes were held firmly in the cardboard lid which was placed on the top of bath. Water was added to the bath such that the tubes were submerged in the water which was maintained at 45 ^{o}C with the aid of a sand bath connected to a thermostat. Use of slightly elevated temperatures allows

a constant reaction temperature to be maintained whereas attempts to run the reaction at ambient temperature led to temperature fluctuation.

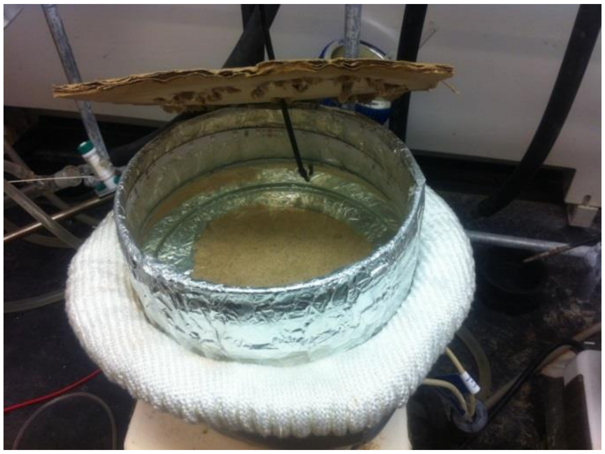

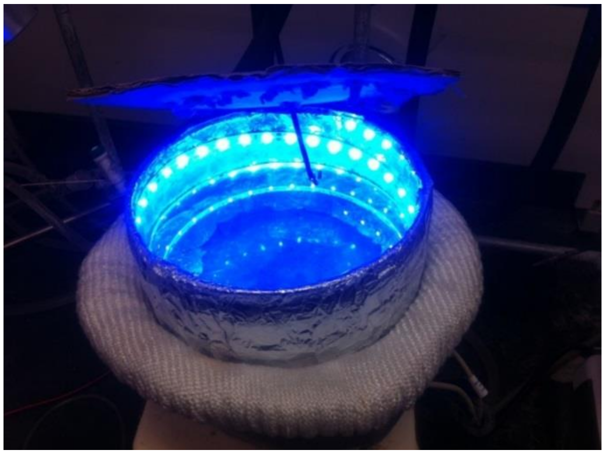

General Procedure A: Aminolysis reaction of fluoroarylated Meldrum's acid adduct with glycine ethyl ester hydrochloride

Fluoroarylated Meldrum's acid adduct was prepared according to the literature procedure[3], though they are available for purchase from Aspira Scientific. Aminolysis with glycine ethyl ester hydrochloride of fluoroarylated Meldrum's acid adduct was performed using a modified literature procedure.[3] In a 25 mL of round bottom pressure flask, fluoroarylated Meldrum's acid adduct (1 equiv) (0.50 g, 0.94 mmol), ethyl 2-aminoacetate hydrochloride (3.0 equiv) and acetonitrile (0.2 M) were added and heated at 80 °C. The reaction was monitored by ^{19}F NMR (reaction was cooled, then an aliquot was transferred to an NMR tube containing C_6D_6 sealed capillary tube). After completion, it was concentrated *in vacuo*, dissolved in ethyl acetate (10 mL), washed with 1M HCl (1 x 20 mL), water (4 x 20 mL), dried over anhydrous $MgSO_4$, filtered and concentrated *in vacuo* to leave the crude product. The resultant crude residue was purified by automated flash chromatography using hexane:ethyl acetate.

Synthesis of Ethyl (2-(2,3,5,6-tetrafluoro-4-(trifluoromethyl)phenyl)acetyl)glycinate

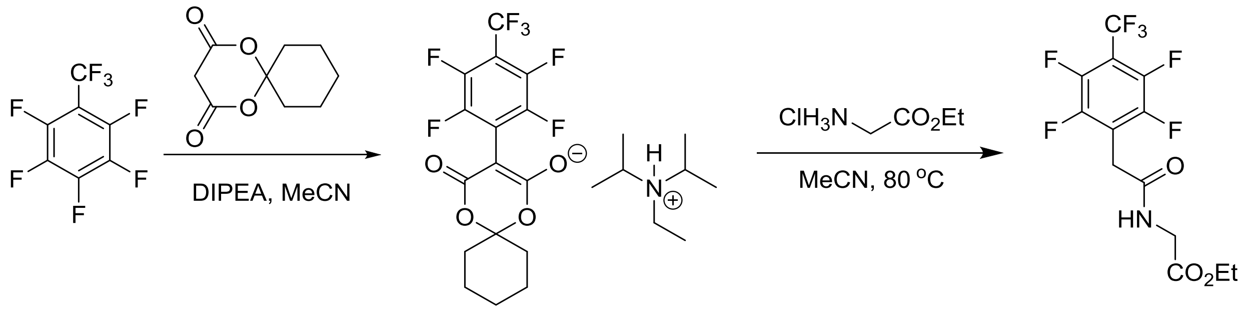

General procedure A was followed was using *N*-ethyl-*N*-isopropylpropan-2-aminium 4-oxo-3-(2,3,5,6-tetrafluoro-4-(trifluoromethyl)phenyl)-1,5-dioxaspiro[5.5]undecan-2-olate[3] (0.50 g, 0.94 mmol), ethyl 2-aminoacetate hydrochloride (0.39 g, 2.8 mmol, 3.0 equiv) and acetonitrile (4 mL). The crude residue was purified by automated flash chromatography using hexane:ethyl acetate (0 % EtOAc for 5 cv and ramped to 100 % EtOAc for 5-30 cv and then held at 100% EtOAc 30-35 cv) on a 12 g silica column to afford **ethyl (2-(2,3,5,6-tetrafluoro-4-(trifluoromethyl)phenyl)acetyl)glycinate** in 92% yield (0.31 g, 0.86 mmol) as a white solid. ^{19}F NMR (376 MHz, Chloroform-*d*) δ -56.36 (t, *J* = 21.6 Hz, 3H), -140.17 (td, *J* = 16.1, 6.2 Hz, 2H), -140.70 (m, 2F). ^{1}H NMR (400 MHz, Chloroform-*d*) δ 6.20 (s, 1H), 4.27 (q, *J* = 7.2 Hz, 2H), 4.10 (d, *J* = 4.9 Hz, 2H), 3.80 (t, *J* = 1.6 Hz, 2H), 1.33 (t, *J* = 7.1 Hz, 3H). ^{13}C{^{1}H} NMR (101 MHz, Chloroform-*d*) δ 169.6, 166.4, 145.5 – 142.5 (m), 118.3 (t, *J* = 18.0 Hz), 146.9 – 144.0 (m), 120.7 (q, 273.1 Hz), 109.1 (ddd, *J* = 47.2, 23.4, 12.0 Hz), 61.8, 41.8, 30.8 – 28.9 (m), 14.0.

Synthesis of methyl 4-(2-((2-ethoxy-2-oxoethyl)amino)-2-oxoethyl)-2,3,5,6-tetrafluorobenzoate

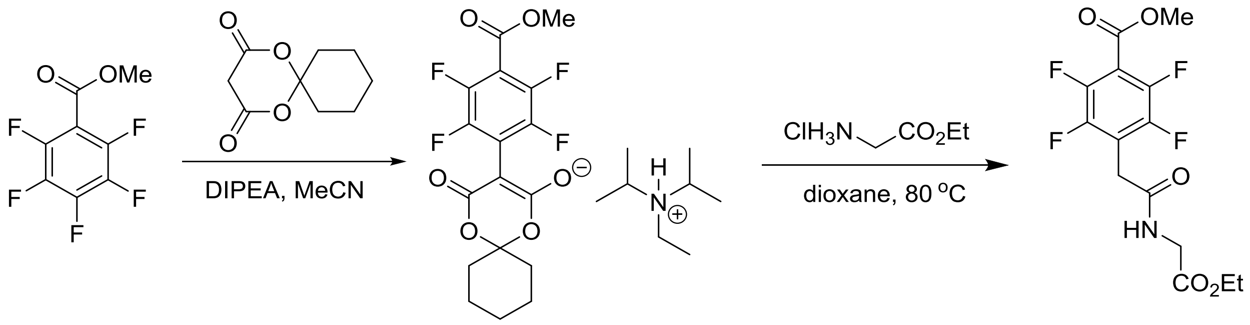

General procedure A was followed using *N*-ethyl-*N*-isopropylpropan-2-aminium 4-oxo-3-(2,3,5,6-tetrafluoro-4-(methoxycarbonyl)phenyl)-1,5-dioxaspiro[5.5]undecan-2-olate[3] (2.0 g, 3.8 mmol), ethyl 2-aminoacetate hydrochloride (1.6 g, 11.4 mmol, 3.0 equiv) and **dioxane** (37 mL, **instead of MeCN** as described in the original procedure) and heated at 80 °C. The crude residue was purified by automated flash chromatography using hexane:ethyl acetate (0 % EtOAc for 5 cv and ramped slowly to 50 % EtOAc for 5-30 cv and then held at 100% EtOAc 30-35 cv) on a 24 g silica column to afford **methyl 4-(2-((2-ethoxy-2-oxoethyl)amino)-2-oxoethyl)-2,3,5,6-tetrafluorobenzoate** in 81% yield (1.05 g, 3.0 mmol) as a white solid. ^{19}F NMR (376 MHz, Chloroform-*d*) δ -139.36 – -139.65 (m, 2F), -141.07 – -141.32 (m, 2F). ^{1}H NMR (400 MHz, Chloroform-*d*) δ 6.15 (s, 1H), 4.27 (q, *J* = 7.1 Hz, 2H), 4.09 (d, *J* = 4.9 Hz, 2H), 4.01 (s, 3H), 3.78 (t, *J* = 1.6 Hz, 2H), 1.32 (t, *J* = 7.2 Hz, 3H). ^{13}C{^{1}H} NMR (101 MHz, Chloroform-*d*) δ 169.6, 167.0, 160.1, 144.5 (ddt, *J* = 256.8, 15.7, 4.6 Hz), 145.1 (ddt, *J* = 248.3, 14.3, 5.2 Hz), 117.2 (t, *J* = 18.2 Hz), 111.6 (t, *J* = 15.7 Hz), 61.7, 53.3, 41.7, 29.8, 14.0.

Synthesis of ethyl (2-(perfluoropyridin-4-yl)acetyl)glycinate

General procedure A was followed using *N*-ethyl-*N*-isopropylpropan-2-aminium 4-oxo-3-(perfluoropyridin-4-yl)-1,5-dioxaspiro[5.5]undecan-2-olate[3] (1.0 g, 2.15 mmol), ethyl 2-aminoacetate hydrochloride (0.90 g, 6.46 mmol, 3.0 equiv) and acetonitrile (5.0 mL) and heated at 80 °C.

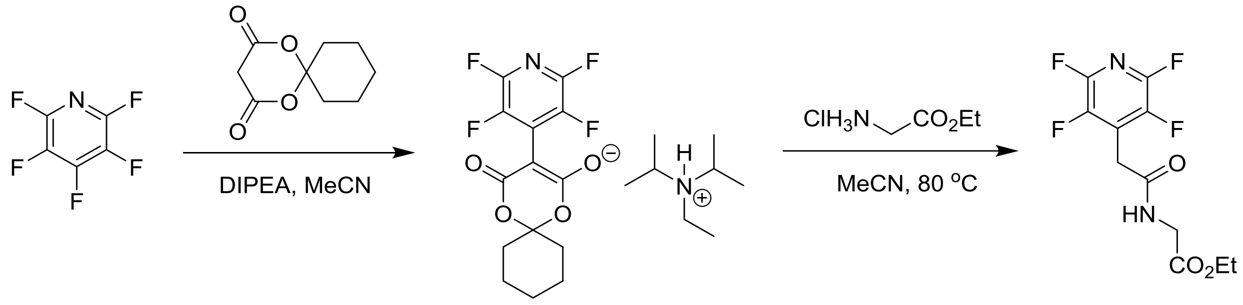

The crude residue was purified by automated flash chromatography using hexane:ethyl acetate (0 % EtOAc for 5 cv and ramped slowly to 50 % EtOAc for 5-30 cv and then held at 100% EtOAc 30-35 cv) on a 24 g silica column to afford **ethyl (2-(perfluoropyridin-4-yl)acetyl)glycinate** in 97 % yield (0.615 g, 2.09 mmol) as a white solid. ^{19}F NMR (376 MHz, Chloroform-*d*) δ -90.42 – -90.82 (m, 2F), -143.14 – -143.58 (m, 2F). ^{1}H NMR (400 MHz, Chloroform-*d*) δ 6.22 (s, 1H), 4.28 (q, *J* = 7.2 Hz, 2H), 4.10 (d, *J* = 4.9 Hz, 2H), 3.83 (s, 2H), 1.33 (t, *J* = 7.1 Hz, 3H). NMR matched with the literature value.[3]

Synthesis of ethyl (2-(3-chloro-2,5,6-trifluoropyridin-4-yl)acetyl)glycinate

General procedure A was followed using *N*-ethyl-*N*-isopropylpropan-2-aminium 3-(3-chloro-2,5,6-trifluoropyridin-4-yl)-4-oxo-1,5-dioxaspiro[5.5]undecan-2-olate[3] (1.0 g, 2.1 mmol), ethyl 2-aminoacetate hydrochloride (0.87 g, 6.24 mmol, 3.0 equiv) and acetonitrile (5 mL) and heated at 80 °C.

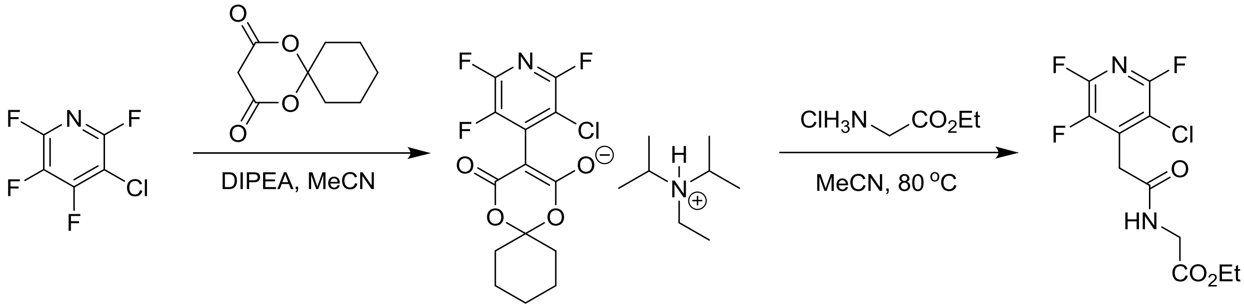

The crude residue was purified by automated flash chromatography using hexane:ethyl acetate (0 % EtOAc for 5 cv and ramped slowly to 50 % EtOAc for 5-30 cv and then held at 100% EtOAc 30-35 cv) on a 24 g silica column to afford e**thyl (2-(3-chloro-2,5,6-trifluoropyridin-4-yl)acetyl)glycinate** in 93 % yield (0.59 g, 1.9 mmol) as a white solid. ^{19}F NMR (376 MHz, Chloroform-*d*) δ -73.39 (dd, *J* = 28.1, 12.5 Hz, 1F), -88.54 (dd, *J* = 21.5, 12.6 Hz, 1F), -143.10

(dd, J = 28.1, 21.4 Hz, 1F). ^{1}H NMR (400 MHz, Chloroform-*d*) δ 6.17 (s, 1H), 4.25 (q, J = 7.2 Hz, 2H), 4.07 (d, J = 4.9 Hz, 2H), 3.90 (d, J = 1.9 Hz, 2H), 1.30 (t, J = 7.1 Hz, 3H). ^{13}C{^{1}H} NMR (101 MHz, Chloroform-*d*) δ 169.5, 165.8, 150.9 (ddd, J = 243.3, 12.4, 2.9 Hz), 147.0 (ddd, J = 247.2, 17.6, 13.6 Hz), 141.7 (ddd, J = 257.7, 26.7, 6.3 Hz), 138.2 (dt, J = 15.2, 2.3 Hz), 114.0 (dd, J = 34.4, 6.6 Hz), 61.8, 41.7, 33.9 (t, J = 2.3 Hz), 14.1.

Synthesis of methyl 2-(3-chloro-2,5,6-trifluoropyridin-4-yl)acetate

Methyl 2-(3-chloro-2,5,6-trifluoropyridin-4-yl)acetate was synthesized by the literature procedure.[3] In a 100 mL round bottom pressure flask, *N*-ethyl-*N*-isopropylpropan-2-aminium 3-(3-chloro-2,5,6-trifluoropyridin-4-yl)-4-oxo-1,5-dioxaspiro[5.5]undecan-2-olate[3] (2.0 g, 4.2 mmol), concentrated hydrochloric acid (434 µL, 5.0 mmol, 1.2 equiv) and methanol (10 mL) and heated at 80 °C. Reaction was monitored by ^{19}F NMR (reaction was cooled, then an aliquot was

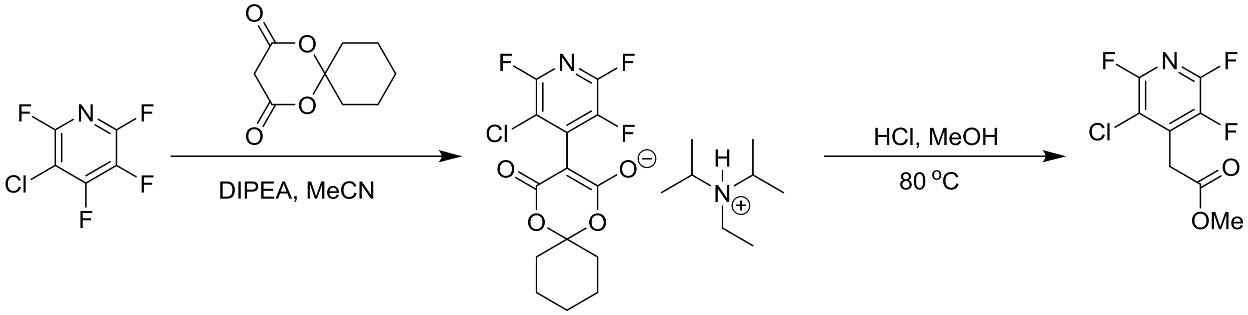

placed in an NMR tube containing C_6D_6 sealed capillary tube). After the completion of reaction, it was concentrated *in vacuo*, dissolved in CH_2Cl_2 (50 mL), washed with sat $NaHCO_3$ (1 x 20 mL), water (2 x 20 mL), dried over anhydrous Na_2SO_4, filtered and concentrated *in vacuo* to leave the crude product. The resultant crude residue was purified by automated flash chromatography using dichloromethane:MeOH (0 % DCM for 15 cv and ramped to 20 % MeOH for 15-20 cv and then held at 20% MeOH 20-25 cv) on a 24 g silica column to afford **methyl 2-(3-chloro-2,5,6-trifluoropyridin-4-yl)acetate** in 100% yield (1.0 g, 4.2 mmol) as a colorless liquid. ^{19}F NMR (376 MHz, Chloroform-*d*) δ -73.48 (dd, J = 28.2, 12.7 Hz, 1F), -88.56 (dd, J = 21.4, 12.7 Hz, 1F), -143.18 – -143.54 (m, 1F). ^{1}H NMR (400 MHz, Chloroform-*d*) δ 3.97 (d, J = 1.9 Hz, 2H), 3.79 (s, 3H). ^{13}C{^{1}H} NMR (101 MHz, Chloroform-*d*) δ 167.3, 150.8 (ddd, J = 243.1, 12.4, 2.9 Hz), 146.9 (ddd, J = 246.9, 17.5, 13.6 Hz), 141.5 (ddd, J = 258.0, 26.8, 6.3 Hz), 138.0 – 137.3 (m), 114.0 (dd, J = 34.6, 6.6 Hz), 52.8, 32.3 (q, J = 2.1 Hz).

Synthesis of Dimethyl-7-oxabicyclo[2.2.1]hept-5-ene exo,exo-2,3-dicarboxylate

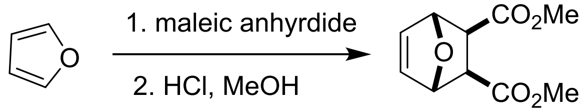

Dimethyl-7-oxabicyclo[2.2.1]hept-5-ene exo,exo-2,3-dicarboxylate was synthesized according to the literature[4] procedure. In a 100 mL round bottom flask, furan (18.0 mL, 249.0 mmol) and maleic anhydride (5.1 g, 52.0 mmol) were added. The reaction mixture was stirred at room temperature overnight. Reaction mixture was concentrated *in vacuo* to obtain the Diels-Alder adduct as white solid which was subjected to reflux in methanol (100 mL) in the presence of catalytic amount of concentrated hydrochloric acid (425 µl, 4.0 mmol) for 2 h. Reaction mixture was cooled to room temperature and methanol was removed via rotary evaporation. Residue was dissolved in DCM (50 mL), washed with sat. $NaHCO_3$ (2 x 30 mL), dried over Na_2SO_4, filtered, and concentrated *in vacuo*. The crude material was purified by flash chromatography using hexane:ethyl acetate (0 % EtOAc for 5 cv and ramped slowly to 50 % EtOAc for 5-30 cv and then held at 100% EtOAc 30-35 cv) on a 24 g silica column to afford **dimethyl-7-oxabicyclo[2.2.1]hept-5-ene exo, exo-2,3-dicarboxylate** in 51% yield over two steps (5.6 g, 26.4 mmol) as a white solid which matched with the literature values.[5] ^{1}H NMR (400 MHz, Chloroform-*d*) δ 6.48 (s, 2H), 5.30 (s, 2H), 3.74 (s, 6H), 2.85 (s, 2H).

Photocatalytic C-F Alkylation

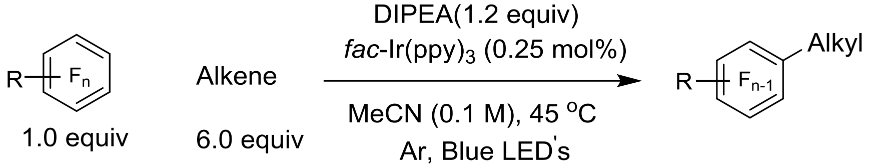

General procedure B for the photocatalytic C-F Alkylation reaction (standard conditions)
An NMR tube was charged with fluoroarenes (0.1 mmol, 1.0 equiv), alkene (0.6 mmol, 6.0 equiv or 0.48 mmol, 4.8 equiv), *N*,*N*-diisopropylethylamine (0.12 mmol, 1.2 equiv) *fac*-tris(2- phenyl pyridinato-C^2, *N*) Iridium(III) ($Ir(ppy)_3$) (0.25 mM, 1 mL in MeCN), sealed glass capillary containing C_6D_6 and was capped with NMR septum (Ace glass, part no. 9096-25). When reaction was run in greater than 0.1 mmol of fluoroarenes, more than one NMR tube was used to set up reaction and each NMR tube had 1 mL of reaction mixture. The reaction was degassed via Ar bubbling for 15 min at 0 °C (to avoid evaporation of *N*,*N*-diisopropylethylamine) and then placed in a light bath (*vide supra*) such that the lower portion of the tube was submerged under the water bath which was maintained at 45 °C. The reaction was monitored periodically by ^{19}F NMR. After the complete consumption of starting material, CH_3CN was removed via rotavap. The residue was treated with deionized water (2 mL) and extracted with DCM (3 x 1 mL). The organic portions were combined and dried with anhydrous $MgSO_4$. The crude product was concentrated *in vacuo* and purified by normal phase or reverse phase chromatography.

General procedure C for the photocatalytic hydrodefluorination reaction
The reaction procedure is the same as **General procedure B**, but if the reaction did not go for completion in 24 h, an additional 3 equiv. of *N*,*N* -diisopropylethylamine were added to the reaction. Then the reaction was redegassed and returned to the light bath. In some cases total 20-

25 equiv of *N,N*-diisopropylethylamine were used. After the complete consumption of starting material the reaction was treated to the same workup described for **General procedure B**.

General procedure D for the photocatalytic C-F alkylation followed hydrodefluorination reaction

The reaction procedure is the same as **General procedure B**, but after the complete consumption of starting material, the volatiles, **including excess alkene**, were removed via rotavap. Then the residue was redissolved in CH_3CN (1 mL). This solution was transferred to an NMR tube containing *N,N*-diisopropylethylamine (0.3 mmol, 3 equiv), a sealed glass (C_6D_6) capillary and was capped with NMR septum, and degassed and resubjected to reaction conditions and workup as described above.

General procedure E for the photocatalytic hydrodefluorination reaction (higher catalyst loading)

The reaction procedure is the same as **General procedure B**, except that the solution of photocatalyst was twice the concentration, *tris*(2- phenyl pyridinato-C^2, *N*) Iridium(III) ($Ir(ppy)_3$) (**0.5 mM**, 1 mL in MeCN). The reaction setup and workup followed that described above. The reaction was monitored periodically by ^{19}F NMR. If the reaction did not go for completion in 24 h, an additional 3 equiv of *N,N* -diisopropylethylamine was added, degassed and reaction continued. Eventually, a total of 20-25 equiv of *N,N*-diisopropylethylamine was used. If after the addition of amine the reaction did not resume (^{19}F NMR) additional catalyst (0.7 or 0.8 mol%) was added and the solution again degassed. After the complete consumption of starting material, the reaction was worked up as previously described.

Synthesis of S-1a (4-cyclohexyl-2,3,5,6-tetrafluoropyridine)

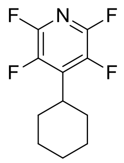

The **General procedure B** was followed using pentafluoropyridine (88 µL, 0.8 mmol, 1 equiv), cyclohexene (483 µL, 4.8 mmol, 6 equiv *N,N*-diisopropylethylamine (167 µL, 0.96 mmol, 1.2 equiv) and 8.0 mL of stock solution of $Ir(ppy)_3$ (1.3 mg, 0.002 mmol, 0.0025 equiv) in CH_3CN was used. The crude material was purified by flash chromatography using hexane : ethyl acetate (0% EtOAc for 8 cv and ramped to 100% EtOAc for 8-14 cv and then held at 100% EtOAc 14-18 cv), on a 24 g silica column to afford **S-1a** in 79% yield (147 mg, 0.6 mmol) as a white solid. ^{19}F NMR (376 MHz, Chloroform-*d*) δ -91.96 – -92.26 (m, 2F), -144.16 – -144.42 (m, 2F). ^{1}H NMR (400 MHz, Chloroform-*d*) δ 3.09 (tt, *J* = 10.7, 5.4 Hz, 1H), 1.97 – 1.72 (m, 7H), 1.50 – 1.24 (m, 3H). $^{13}C\{^1H\}$ NMR (101 MHz, Chloroform-*d*) δ 143.6 (dddd, *J* = 244.4, 17.6, 13.1, 2.4 Hz), 141.9 – 138.8 (m), 139.2–138.9 (m), 36.3, 30.1, 26.3, 25.4. FT-IR cm^{-1} 2941, 2864, 1639, 1446, 1403. GC/MS (m/z, relative intensity) 233 (M^+, 15), 177 (30), 41 (100). HRMS (ESI) Calcd. for $C_{11}H_{11}F_4N$ $[M+H]^+$ 234.0906, found 234.4539. Melting point 62-65 ^oC.

Synthesis of S-2a (4-cyclohexyl-2,3,5,6-tetrafluorobenzonitrile)

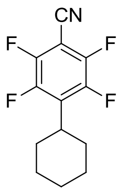

The **General procedure B** was followed using pentafluorobenzonitrile (36 µL, 0.3 mmol, 1 equiv), cyclohexene (182 µL, 1.8 mmol, 6 equiv), *N,N*-diisopropylethylamine (63 µL, 0.36 mmol, 1.2 equiv) and 3.0 mL of stock solution of Ir(ppy)$_3$ (0.5 mg, 0.0008 mmol, 0.0025 equiv) in CH_3CN was used. The crude material was purified by flash chromatography using hexane : ethyl acetate (0-15% EtOAc for 25 cv and ramped to 100% EtOAc for 25-26 cv and then held at 100% EtOAc 26-30 cv), on a 12 g silica column to afford **S-2a** in 64% yield (50 mg, 0.19 mmol) as a colorless liquid. ^{19}F NMR (376 MHz, Chloroform-*d*) δ -133.14 – -133.30 (m, 2F), -139.57 (td, *J* = 16.2, 7.1 Hz, 2F). ^{1}H NMR (400 MHz, Chloroform-*d*) δ 3.09 (tt, *J* = 11.4, 4.4 Hz, 1H), 1.92 – 1.72 (m, 7H), 1.46 – 1.22 (m, 3H). ^{13}C{^{1}H} NMR (101 MHz, Chloroform-*d*) δ 148.7-145.5 (ddt, *J* = 261.2, 17.8, 3.8 Hz), 146.4-143.4 (dddd, *J* = 248.6, 12.4, 7.4, 4.2 Hz), 132.4 (t, *J* = 16.2 Hz), 107.7 (t, *J* = 3.8 Hz), 91.9 – 91.3 (m), 36.3, 30.3, 26.4, 25.4. FT-IR cm^{-1} 2939, 2864, 2242, 1655, 1452, 1398. GC/MS (m/z, relative intensity) 257 (M$^+$, 52), 201 (80), 56 (100), 41 (100). HRMS (ESI) Calcd. for $C_{13}H_{11}F_4N$ [M + Na]$^+$ 280.0725, found 280.1230.

Synthesis of S-3a (*S*-(4-cyclohexyl-2,3,5,6-tetrafluorophenyl) *O*-ethyl carbonothioate)

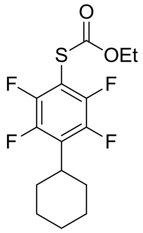

The **General procedure B** was followed using *O*-ethyl *S*-(perfluorophenyl) carbonothioate (27 mg, 0.10 mmol, 1 equiv), cyclohexene (60 µL, 0.6 mmol, 6 equiv), *N,N*-diisopropylethylamine (21 µL, 0.12 mmol, 1.2 equiv) and 1.0 mL of stock solution of Ir(ppy)$_3$ (0.16 mg, 0.00025 mmol, 0.0025 equiv) in CH_3CN was used. The crude material was purified by flash chromatography using hexane : ethyl acetate (0-2% EtOAc for 20 cv and ramped to 100 % then held at 100% EtOAc 20-30 cv), on a 24 g silica column. After that, prep tlc was run in hexane: 10% DCM to afford **S-3a** in 62% yield (21 mg, 0.06 mmol) as a colorless liquid. ^{19}F NMR (376 MHz, Chloroform-*d*) δ -132.89 – -133.09 (m, 2F), -141.69 – -141.85 (m, 2F). ^{1}H NMR (400 MHz, Chloroform-*d*) δ 4.34 (q, *J* = 7.1 Hz, 2H), 3.06 (td, *J* = 11.2, 5.4 Hz, 1H), 1.96 – 1.70 (m, 7H), 1.47 – 1.37 (m, 3H), 1.34 (t, *J* = 7.1 Hz, 3H). ^{13}C{^{1}H} NMR (101 MHz, Chloroform-*d*) δ 165.5, 148.4-145.5 (ddt, *J* = 249.0, 15.8, 3.5 Hz), 146.4-143.5 (dddd, *J* = 247.3, 14.7, 7.8, 4.2 Hz), 128.3 (t, *J* = 16.5 Hz), 104.5 (t, *J* = 20.7 Hz), 65.3, 36.0, 30.6, 26.6, 25.6, 14.2. FT-IR cm^{-1} 2990, 2931, 1746, 1473, 1454. GC/MS (m/z, relative intensity) 336 (M$^+$, 5), 264 (65), 208 (90), 41 (100). HRMS (ESI) Calcd. for $C_{15}H_{16}F_4O_2S$ [M + H]$^+$ 337.0885, found 337.1615.

Note: The isolated compound appears pure by GC-MS and ^{1}H NMR. However, ^{19}F NMR contains some unidentified, inseparable peaks in 3 %, and 9% minor rr respectively.

Synthesis of S-4a (methyl 4-cyclohexyl-2,3,5,6-tetrafluorobenzoate)

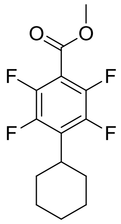

The **General procedure B** was followed using methyl 2,3,4,5,6-pentafluorobenzoate (23 mg, 0.10 mmol, 1 equiv), cyclohexene (60 µL, 0.6 mmol, 6 equiv), *N,N*-diisopropylethylamine (21 µL, 0.12 mmol, 1.2 equiv) and 1.0 mL of stock solution of $Ir(ppy)_3$ (0.16 mg, 0.00025 mmol, 0.0025 equiv) in CH_3CN was used. The crude material was purified by flash chromatography using hexane : ethyl acetate (0-2% EtOAc for 15 cv and ramped to 100% EtOAc held at 100% EtOAc for 15-25 cv), on a 4 g silica column followed by prep tlc using 95-5 Hexane : EtOAc to afford **S-4a** in 76% yield (22 mg, 0.07 mmol) as a colorless liquid. ^{19}F NMR (376 MHz, Chloroform-*d*) δ -140.24 – -140.40 (m, 2F), -142.11 (td, *J* = 14.7, 4.2 Hz, 2F). ^{1}H NMR (400 MHz, Chloroform-*d*) δ 3.96 (s, 3H), 3.05 ((tt, *J* = 11.6, 4.0 Hz, 1H), 1.94 – 1.70 (m, 7H), 1.34 (tt, *J* = 24.7, 12.3 Hz, 3H). ^{13}C{^{1}H} NMR (101 MHz, Chloroform-*d*) δ 160.5 (dd, *J* = 3.6, 1.9 Hz), 146.4 – 145.8 (m), 144.0 – 143.2 (m), 128.6 (t, *J* = 16.4 Hz), 109.8 (t, *J* = 15.7 Hz), 53.1, 35.9 (t, *J* = 1.7 Hz), 30.6 (t, *J* = 2.1 Hz), 26.6, 25.6. FT-IR cm^{-1} 2955, 2890, 1738, 1476, 1446, 1411. GC/MS (m/z, relative intensity) 290 (M+, 30), 234 (50), 203 (60), 41 (100). HRMS (ESI) Calcd. for $C_{14}H_{14}F_4O_2$ $[M + H]^+$ 291.1008, found 291.1559.

Synthesis of S-5a (2-(4-cyclohexyl-2,3,5,6-tetrafluorophenyl)benzo[d]oxazole)

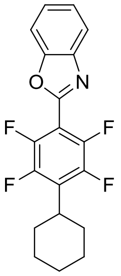

The **General procedure B** was followed using 2-(perfluorophenyl)benzo[d]oxazole (57 mg, 0.20 mmol, 1 equiv), cyclohexene (121 µL, 1.2 mmol, 6 equiv), *N,N*-diisopropylethylamine (42 µL, 0.24 mmol, 1.2 equiv) and 2.0 mL of stock solution of $Ir(ppy)_3$ (0.3 mg, 0.0005 mmol, 0.0025 equiv) in CH_3CN was used. The crude material was purified by flash chromatography using hexane : ethyl acetate (0-3% EtOAc for 13 cv and ramped to 100% EtOAc and then held at 100% EtOAc for 15-30 cv) on a 12 g silica column followed by reverse phase chromatography using MeCN : MeOH (0-0.2% MeOH for 4 cv and then ramped to 100% MeOH and then held at 100% MeOH for 4-30 cv), on a 26 g C18 reverse column to afford **S-5a** in 49% yield (34 mg, 0.09 mmol) as a white solid. ^{19}F NMR (376 MHz, Chloroform-*d*) δ -138.80 – -138.98 (m, 2F), -141.95 (td, *J* = 14.9, 5.2 Hz, 2F). ^{1}H NMR (400 MHz, Chloroform-*d*) δ 7.94 – 7.89 (m, 1H), 7.70 – 7.66 (m, 1H), 7.46 (ddd, *J* = 6.7, 4.3 Hz, 2H), 3.20 – 3.09 (m, 1H), 1.99 – 1.75 (m, 7H), 1.40 (tt, *J* = 15.6, 7.9 Hz, 3H). ^{13}C{^{1}H} NMR (101 MHz, Chloroform-*d*) δ 153.6 – 153.5 (m), 150.5, 146.5 (ddt, *J* = 26.4, 17.5, 4.6 Hz), 144.5 – 143.6 (m), 141.2, 128.5 (t, *J* = 16.5 Hz), 126.2, 125.0, 120.8, 111.0, 36.0 (d, *J* = 1.8 Hz), 30.7 (t, *J* = 2.2 Hz), 26.6, 25.6. FT-IR cm^{-1} 3022, 2939, 2856, 1612, 1543, 1486, 1449. GC/MS (m/z, relative intensity) 349 (M^+, 100), 293 (100), 63 (70), 41 (60). HRMS (ESI) Calcd. for $C_{19}H_{15}F_4NO$ $[M + H]^+$ 350.1168, found 350.1154. Melting point 144-148 ^{o}C

Synthesis of S-6a ((4-cyclohexyl-2,3,5,6-tetrafluorophenyl)diphenylphosphine)

The **General procedure B** was followed using (perfluorophenyl)diphenylphosphane (35 mg, 0.10 mmol, 1 equiv), cyclohexene (60 µL, 0.6 mmol, 6 equiv), *N,N*-diisopropylethylamine (21 µL, 0.12

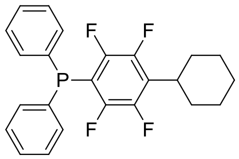

mmol, 1.2 equiv) and 1.0 mL of stock solution of Ir(ppy)$_3$ (0.16 mg, 0.00025 mmol, 0.0025 equiv) in CH_3CN was used. The crude material was purified by flash chromatography using hexane : ethyl acetate (0% EtOAc for 10 cv, ramped to 100% EtOAc and then held at 100% EtOAc for 10-22 cv), on a 12 g silica column followed by prep tlc in hexane to afford **S-6a** in 55% yield (23 mg, 0.06 mmol) as a white solid. ^{19}F NMR (376 MHz, Chloroform-*d*) δ -129.26 – -129.51 (m, 2F), -142.24 (q, *J* = 12.5, 12.0 Hz, 2F). ^{1}H NMR (400 MHz, Chloroform-*d*) δ 7.44 (dt, *J* = 7.7, 3.7 Hz, 4H), 7.37 (d, *J* = 4.2 Hz, 6H), 3.04 (tt, *J* = 10.8, 4.4 Hz, 1H), 1.82 (dd, *J* = 23.9, 9.9 Hz, 7H), 1.34 (tt, *J* = 16.5, 7.8 Hz, 3H). ^{13}C{^{1}H} NMR (101 MHz, Chloroform-*d*) δ 149.9 – 146.5 (m), 146.6 – 143.5 (m), 134.1 , 134.0 , 133.1 , 132.9 , 129.1 , 128.6 , 128.5 , 127.6 (t, *J* = 16.5 Hz), 112.9 (dd, *J* = 35.4, 21.2 Hz), 35.9 , 30.7 , 26.7 , 25.7. FT-IR cm^{-1} 2949, 2853, 1446. GC/MS (m/z, relative intensity) 416 (M^{+}, 100), 183 (20), 108 (20). HRMS (ESI) Calcd. for $C_{24}H_{21}F_4P$ [M + H]$^{+}$ 417.1395, found 417.2508. Melting point 78-80 ^{o}C.

Synthesis of S-7a (2,3,5,6-tetrafluoro-4-hexylpyridine)

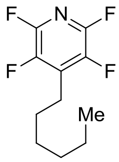

The **General procedure B** was followed using pentafluoropyridine (11 µL, 0.10 mmol, 1 equiv), hexene (75 µL, 0.6 mmol, 6 equiv), *N,N*-diisopropylethylamine (21 µL, 0.12 mmol, 1.2 equiv) and 1.0 mL of stock solution of Ir(ppy)$_3$ (0.16 mg, 0.00025 mmol, 0.0025 equiv) in CH_3CN was used. The crude material was purified by flash chromatography using hexane : ethyl acetate (0-5% EtOAc for 18 cv, ramped to 100% EtOAc then held at 100% EtOAc for 18-40 cv), on a 12 g silica column to afford **S-7a** in 67% yield (16 mg, 0.07 mmol) as a colorless liquid. ^{19}F NMR (376 MHz, Chloroform-*d*) δ -91.94 – -92.18 (m, 2F), -145.61 – -145.85 (m, 2F). ^{1}H NMR (400 MHz, Chloroform-*d*) δ 2.83 (t, *J* = 7.7 Hz, 2H), 1.66 (p, *J* = 7.4 Hz, 2H), 1.46 – 1.30 (m, 6H), 0.96 – 0.87 (m, 3H). ^{13}C{^{1}H} NMR (101 MHz, Chloroform-*d*) δ 144.7 – 141.5 (m), 141.9 – 138.9 (m), 135.5 (tt, *J* = 17.2, 2.6 Hz), 31.3, 28.8, 28.5, 23.7, 22.4, 14.0. FT-IR cm^{-1} 2960, 2931, 1653, 1490. GC/MS (m/z, relative intensity) 235 (M^{+}, 10), 165 (15), 147 (20), 43 (100). HRMS (ESI) Calcd. for $C_{11}H_{13}F_4N$ [M + Na]$^{+}$ 258.0882, found 258.0888.

Synthesis of S-8a (2,3,5,6-tetrafluoro-4-(tetrahydro-2H-pyran-2-yl)pyridine)

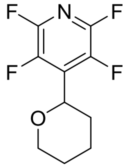

The **General procedure** B was followed using pentafluoropyridine (22 µL, 0.20 mmol, 1 equiv), dihydropyran (108 µL, 1.2 mmol, 6 equiv), *N,N*-diisopropylethylamine (42 µL, 0.24 mmol, 1.2 equiv) and 2.0 mL of stock solution of Ir(ppy)$_3$ (0.3 mg, 0.0005 mmol, 0.0025 equiv) in CH_3CN was used. The crude material was purified by flash chromatography using hexane : EtOAc (0-30% EtOAc for 18 cv, ramped to 100% EtOAc and then held at 100% EtOAc for 30-35 cv), on a 12 g silica column to afford **S-8a** in 72% yield (34 mg, 0.15 mmol, 1:1 rr, NMR yield 75% with trifluoroacetic acid as an internal reference)as a colorless liquid.

Due to volatility of compound 8a we did not attempt hard to remove residual solvent hexane.

HRMS (ESI) Calcd. for $C_{10}H_9F_4NO$ $[M + K]^+$ 274.0257, found 274.2516.

(2,3,5,6-tetrafluoro-4-(tetrahydro-2H-pyran-2-yl)pyridine)

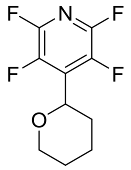

^{19}F NMR (376 MHz, Chloroform-*d*) δ -91.17 – -91.58 (m, 2F), -143.53 – -143.85 (m, 2F). ^{1}H NMR (400 MHz, Chloroform-*d*) δ 4.81 (dd, *J* = 11.4, 2.3 Hz, 1H), 4.13 (ddd, *J* = 11.6, 3.9, 1.9 Hz, 1H), 3.58 (td, *J* = 11.7, 2.2 Hz, 1H), 2.09 – 1.94 (m, 2H), 1.87 – 1.55 (m, 4H). ^{13}C{^{1}H} NMR (101 MHz, Chloroform-*d*) δ 144.9 – 142.4 (m), 141.4 – 138.5 (m), 134.5 – 133.8 (m), 72.2 (t, *J*=2.2 Hz), 69.5, 30.2 (d, *J*=1.7 Hz), 25.4, 23.6. FT-IR cm^{-1} 2917, 2856, 1709, 1642, 1468. GC/MS (m/z, relative intensity) 235 (M^+, 5), 56 (50), 41 (100).

Regioisomer of **S-8a 2,3,5,6-tetrafluoro-4-(tetrahydro-2H-pyran-3-yl)pyridine**

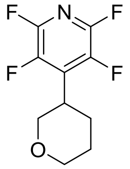

^{19}F NMR (376 MHz, Chloroform-*d*) δ -91.32 – -91.69 (m, 2F), -143.14 – -143.46 (m, 2F). ^{1}H NMR (400 MHz, Chloroform-d) δ 4.11 – 4.00 (m, 1H), 3.93 (ddd, *J* = 11.1, 4.2, 2.0 Hz, 1H), 3.78 (tt, *J* = 11.0, 2.3 Hz, 1H), 3.48 (dtd, *J* = 20.2, 11.6, 3.5 Hz, 2H), 2.15 – 1.94 (m, 2H), 1.93 – 1.64 (m, 2H).

Synthesis of S-9a (3-(perfluoropyridin-4-yl)cyclohexanone)

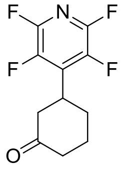

The **General procedure B** was followed using pentafluoropyridine (16 µL, 0.15 mmol, 1 equiv), 2-cyclohexen-1-one (87 µL, 0.9 mmol, 6 equiv), *N,N*-diisopropylethylamine (31 µL, 0.18 mmol, 1.2 equiv) and 1.5 mL of stock solution of $Ir(ppy)_3$ (0.2 mg, 0.0004 mmol, 0.0025 equiv) in CH_3CN was used. The crude material was purified by flash chromatography using hexane : ethyl acetate (0-3% EtOAc for 30 cv, ramped to 100% EtOAc and then held at 100% EtOAc for 30-35 cv), on a 12 g silica column to afford **S-9a** in 54% yield (20 mg, 0.08 mmol) as a colorless liquid. ^{19}F NMR (376 MHz, Chloroform-*d*) δ -90.28 – -90.50 (m, 2F), -143.84 – -144.07 (m, 2F). ^{1}H NMR (400 MHz, Chloroform-*d*) δ 3.56 (tt, *J* = 12.8, 3.9 Hz, 1H), 2.88 (t, *J* = 13.8 Hz, 1H), 2.61 – 2.41 (m, 3H), 2.31 – 2.18 (m, 2H), 2.10 – 2.00 (m, 1H), 1.92 – 1.76 (m, 1H). ^{13}C{^{1}H} NMR (101 MHz, Chloroform-*d*) δ 207.4, 143.7 (dddd, *J* = 245.9, 17.3, 13.0, 2.8 Hz), 141.9 – 138.7 (m), 135.3 (tt, *J* = 14.1, 2.1 Hz), 44.3 (t, *J* = 1.8 Hz), 40.8, 35.5 (t, *J* = 1.9 Hz), 28.8 (t, *J* = 1.8 Hz), 25.3. FT-IR cm^{-1} 2965, 2869, 1714, 1650, 1486, 1452, 1425. GC/MS (m/z, relative intensity) 247 (M^+, 30), 200 (90), 177 (50), 55(100), 41 (80). HRMS (ESI) Calcd. for $C_{11}H_9F_4NO$ $[M + Na]^+$ 270.0518, found 270.1384.

Synthesis of S-10a (3-methyl-2-(perfluoropyridin-4-yl)butan-1-ol)

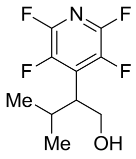

The **General procedure B** was followed using pentafluoropyridine (22 µL, 0.2 mmol, 1 equiv), 3-methylbut-2-en-1-ol (120 µL, 1.2 mmol, 6 equiv), *N,N*-diisopropylethylamine (42 µL, 0.24 mmol, 1.2 equiv) and 2.0 mL of stock solution of $Ir(ppy)_3$ (0.3 mg, 0.0005 mmol, 0.0025 equiv) in CH_3CN was used. The crude

material was purified by flash chromatography using hexane : ethyl acetate (0-15 % EtOAc for 15 cv, ramped to 100 % EtOAc and then held at 100% EtOAc for 15-25 cv), on a 12 g silica column to afford **S-10a** in 43% yield (20 mg, 0.08 mmol) as a colorless liquid. ^{19}F NMR (376 MHz, Chloroform-*d*) δ -91.79 – -92.13 (m, 2F), -142.83 – -143.09 (m, 2F). ^{1}H NMR (400 MHz, Chloroform-*d*) δ 4.03 (dd, *J* = 10.5, 5.5 Hz, 1H), 3.94 (t, *J* = 10.1 Hz, 1H), 3.12 (td, *J* = 9.6, 5.6 Hz, 1H), 2.09 (dt, *J* = 9.1, 6.8 Hz, 1H), 1.40 (s, 1H), 1.01 (d, *J* = 6.6 Hz, 3H), 0.76 (d, *J* = 6.7 Hz, 3H). ^{13}C{^{1}H} NMR (101 MHz, Chloroform-*d*) δ 145.6 – 142.6 (m), 142.7 – 139.9 (m), 136.1 (ddt, *J* = 16.8, 15.0, 1.9 Hz), 62.9 (t, *J* = 2.5 Hz), 48.2 – 48.1 (m), 28.9 (t, *J* = 1.9 Hz), 21.37, 21.35. FT-IR cm^{-1} 3544-3174, 2971, 1650, 1457. GC/MS (m/z, relative intensity) 237 (M^{+}, 10), 205 (40), 192 (35), 177 (55), 41 (100). HRMS (ESI) Calcd. for $C_{10}H_{11}F_4NO$ [M + K]$^{+}$ 276.0414, found 276.1589.
Note: Isolated product contains 10% of an inseparable (alkenylated) product.

Synthesis of S-11a (6-(perfluoropyridin-4-yl)hexan-1-ol)

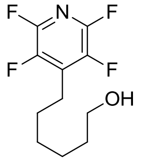

The **General procedure B** was followed using pentafluoropyridine (44 µL, 0.40 mmol, 1 equiv), 5-hexen-1-ol (285 µL, 2.4 mmol, 6 equiv), *N,N*-diisopropylethylamine (84 µL, 0.48 mmol, 1.2 equiv) and 4.0 mL of stock solution of Ir(ppy)$_3$ (0.66 mg, 0.001 mmol, 0.0025 equiv) in CH_3CN was used. The crude material was purified by flash chromatography using hexane : ethyl acetate (0-22% EtOAc for 35 cv, ramped to 100% EtOAc and then held at 100% EtOAc for 35-42 cv) on a 24 g silica column to afford **S-11a** in 53% yield (53 mg, 0.21 mmol) as a colorless liquid. ^{19}F NMR (376 MHz, Chloroform-*d*) δ -91.73 – -92.02 (m, 2F), -145.49 – -145.78 (m, 2F). ^{1}H NMR (400 MHz, Chloroform-*d*) δ 3.58 (t, *J* = 6.5 Hz, 2H), 2.75 (t, *J* = 7.7 Hz, 2H), 1.59 (t, *J* = 7.4 Hz, 2H), 1.50 (td, *J* = 9.7, 8.3, 4.9 Hz, 2H), 1.34 (dq, *J* = 9.7, 6.2, 5.0 Hz, 4H). ^{13}C{^{1}H} NMR (101 MHz, Chloroform-*d*) δ 144.7 – 141.7 (m), 141.9 – 138.9 (m), 135.3 (tt, *J* = 17.0, 2.5 Hz), 62.8, 32.5, 28.9, 28.5, 25.3, 23.6. FT-IR cm^{-1} 3536-3153, 2939, 2861, 1647, 1490. GC/MS (m/z, relative intensity) 251 (M^{+}, 5), 165 (30), 69 (65), 41 (100). HRMS (ESI) Calcd. for $C_{11}H_{13}F_4NO$ [M + H]$^{+}$ 252.1012, found 252.1279.

Synthesis of S-12a (3-methyl-2-(perfluoropyridin-3-yl)butan-1-ol)

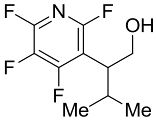

The **General procedure B** was followed using 3-chloro-2,4,5,6-tetrafluoropyridine (12 µL, 0.10 mmol, 1 equiv), 3-methylbut-2-en-1-ol (60 µL, 0.6 mmol, 6 equiv), *N,N*-diisopropylethylamine (21 µL, 0.12 mmol, 1.2 equiv) and 1.0 mL of stock solution of Ir(ppy)$_3$ (0.16 mg, 0.00025 mmol, 0.0025 equiv) in CH_3CN was used. The crude material was purified by flash chromatography using hexane : ethyl acetate (0-22% EtOAc for 30 cv, ramped to 100% EtOAc and then held at 100% EtOAc for 30-35 cv), on a 4 g silica column to afford **S-12a** in 58% yield (14 mg, 0.06 mmol) as a colorless liquid. ^{19}F NMR (376 MHz, Chloroform-*d*) δ -69.57 (s, 1F), -87.70 (dd, *J* = 24, 36 Hz, 1F), -115.48 (dd, *J* = 16.0, 36.0 Hz, 1F),

-167.17 (dt, J = 23.7, 20.5 Hz, 1F). ^{1}H NMR (400 MHz, Chloroform-d) δ 4.04 (dd, J = 10.5, 5.6 Hz, 1H), 3.92 (t, J = 10.1 Hz, 1H), 2.96 (td, J = 9.5, 5.6 Hz, 1H), 2.21 – 2.02 (m, 1H), 1.53 (s, 1H), 1.05 (d, J = 6.6 Hz, 3H), 0.81 (d, J = 6.7 Hz, 3H). ^{13}C{^{1}H} NMR (101 MHz, Chloroform-d) δ 160.6 – 157.4 (m), 153.4 (dddd, J = 242.0, 15.1, 12.6, 3.5 Hz), 149.2 – 146.3 (m), 132.8 (dddd, J = 259.1, 28.5, 16.2, 8.2 Hz), 111.9 (dddd, J = 34.6, 15.5, 7.3, 3.2 Hz), 62.6 (t, J = 2.5 Hz), 46.0 (d, J = 4.0 Hz), 28.4 , 21.2 , 21.1. FT-IR cm^{-1} 3547-3156, 2971, 2880, 1628, 1484, 1462, 1393. GC/MS (m/z, relative intensity) 237 (M^+, 5), 177 (50), 192 (35), 164 (50), 43 (100). HRMS (ESI) Calcd. for $C_{10}H_{11}F_4NO$ $[M + K]^+$276.0414, found 276.0796.
Note: Isolated compound contains 6% of an inseparable alkenylated product.

Synthesis of S-13a (4-(6-chlorohexyl)-2,3,5,6-tetrafluoropyridine)

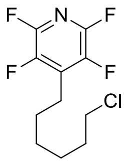

The **General procedure B** was followed using pentafluoropyridine (22 µL, 0.20 mmol, 1 equiv), 6-chloro-1-hexene (158 µL, 1.2 mmol, 6 equiv), *N,N*-diisopropylethylamine (42 µL, 0.24 mmol, 1.2 equiv) and 2.0 mL of stock solution of $Ir(ppy)_3$ (0.3 mg, 0.0005 mmol, 0.0025 equiv) in CH_3CN was used. The crude material was purified by flash chromatography using hexane : ethyl acetate (0% EtOAc for 15 cv, ramped to 100% EtOAc and then held at 100% EtOAc for 15-25 cv), on a 4 g silica column to afford **S-13a** in 76% yield (41 mg, 0.15 mmol) as a colorless liquid. ^{19}F NMR (376 MHz, Chloroform-d) δ -91.67 – -91.94 (m, 2F), -145.52 – -145.76 (m, 2F). ^{1}H NMR (400 MHz, Chloroform-d) δ 3.54 (t, J = 6.6 Hz, 2H), 2.82 (t, J = 7.7 Hz, 2H), 1.78 (p, J = 6.7 Hz, 2H), 1.67 (p, J = 7.6 Hz, 2H), 1.50 (dt, J = 14.2, 6.9 Hz, 2H), 1.41 (q, J = 7.4 Hz, 2H). ^{13}C{^{1}H} NMR (101 MHz, Chloroform-d) δ 144.8 – 141.9 (m), 142.0 – 138.9 (m), 135.1 (tt, J = 17.2, 2.6 Hz), 44.9, 32.3, 28.4, 28.3, 26.4, 23.6. FT-IR cm^{-1} 2949, 2872, 1650, 1462, 1414. GC/MS (m/z, relative intensity) 269 (M^+, 10), 147 (30), 69 (65), 41 (100). HRMS (ESI) Calcd. for $C_{11}H_{12}ClF_4N$ $[M + H]^+$ 270.0673, found 270.1385.

Synthesis of S-14a 3-methyl-2-(perfluorophenyl)butan-1-ol

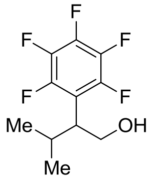

The **General procedure B** was followed using hexafluorobenzene (41 µL, 0.35 mmol, 1 eq), 3-methylbut-2-en-1-ol (210 µL, 2.1 mmol, 6 equiv), *N,N*-diisopropylethylamine (73 µL, 0.42 mmol, 1.2 eq) and 3.5 mL of stock solution of $Ir(ppy)_3$ (0.57 mg, 0.0009 mmol, 0.0025 equiv) in CH_3CN was used. The crude material was purified by flash chromatography using hexane : ether (0-30% ether for 40 cv, ramped to 100% ether and then held at 100% ether for 40-60 cv), on a 24 g silica column to afford **S-14a** in 62% yield (55 mg, 0.22 mmol) as a colorless liquid. ^{19}F NMR (376 MHz, Chloroform-d) δ -141.29 (dd, J = 23.1, 7.9 Hz, 2F), -157.02 (t, J = 20.9 Hz, 1F), -162.53 – -162.83 (m, 2F). ^{1}H NMR (400 MHz, Chloroform-d) δ 4.05 (dd, J = 10.6, 5.6 Hz, 1H), 3.93 (tt, J = 10.4, 1.5 Hz, 1H), 3.05 (td, J = 9.5, 5.5 Hz, 1H), 2.09 (ttd, J = 9.2, 6.7, 4.4 Hz, 1H), 1.05 (d, J = 6.6 Hz, 3H), 0.79 (d,

J = 6.7 Hz, 3H). $^{13}C\{^1H\}$ NMR (101 MHz, Chloroform-*d*) δ 145.63 (dddd, J = 245.4, 14.4, 10.0, 4.0 Hz), 141.3 – 138.1 (m), 137.49 (dddd, J = 256.6, 17.7, 12.2, 5.1 Hz), 115.7 – 115.1 (m), 63.0 (t, J = 2.5 Hz), 46.6 (d, J = 1.9 Hz), 28.6 (t, J = 1.9 Hz), 21.3, 21.2. FT-IR cm^{-1}3564-3132, 2970, 1530, 1497. GC/MS (m/z, relative intensity) 254 (M^+, 5), 194 (80), 181 (75), 43 (100). HRMS (ESI) Calcd. for $C_{11}H_{11}F_5O$ $[M + H]^+$ 255.0808, found 255.1203.

Note: Isolated compound contains 6% of an inseparable alkenylated product.

Synthesis of S-15a 4-exo-(bicyclo[2.2.1]heptan-2-yl)-2,3,5,6-tetrafluoropyridine

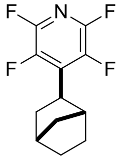

The **General procedure B** was followed using pentafluoropyridine (22 µL, 0.20 mmol, 1 equiv), bicyclo[2.2.1]hept-2-ene (112 mg, 1.2 mmol, 6 equiv), *N*,*N*-diisopropylethylamine (42 µL, 0.24 mmol, 1.2 equiv) and 2.0 mL of stock solution of $Ir(ppy)_3$ (0.3 mg, 0.0005 mmol, 0.0025 equiv) in CH_3CN was used. The crude material was purified by flash chromatography using hexane : ethyl acetate (0%EtOAc for 10 cv, ramped to 100% EtOAc and then held at 100% EtOAc for 10-28 cv) on a 12 g silica column to afford **S-15a** in 61% yield (30 mg, 0.12 mmol, dr 10:1) as a colorless liquid. ^{19}F NMR (376 MHz, Chloroform-*d*) δ -92.25 – -92.60 (m, 2F), -143.01 – -143.25 (m, 2F). ^1H NMR (400 MHz, Chloroform-*d*) δ 3.11 – 3.01 (dd, J = 9.7, 5.9 Hz, 1H), 2.61 (dd, J = 3.6, 1.9 Hz, 1H), 2.42 (t, J = 3.7 Hz, 1H), 1.92 (t, J = 10.8 Hz, 1H), 1.75 – 1.61 (m, 4H), 1.41 – 1.30 (m, 3H). $^{13}C\{^1H\}$ NMR (101 MHz, Chloroform-*d*) δ 143.8 (dddd, J = 244.1, 18.1, 13.5, 2.7 Hz), 141.7 – 138.7 (m), 139.05 (dt, J = 11.4, 2.1 Hz), 41.1 (t, J = 2.3 Hz), 40.3, 38.5 (t, J = 2.1 Hz), 37.5 (t, J = 3.5 Hz), 36.4, 30.9, 28.2. FT-IR cm^{-1} 2957, 2874, 1647, 1454. GC/MS (m/z, relative intensity) 245 (M^+, 5), 178(20), 66 (100). HRMS (ESI) Calcd. for $C_{12}H_{11}F_4N$ $[M + K]^+$ 268.0725, found 268.0412.
Note: The relevant coupling constant of the product matched that of the literature for the exo-isomer.[6] The stereochemistry was assigned based on this coupling constant.

Synthesis of S-16a dimethyl 5-(perfluoropyridin-4-yl)-exo-7-oxabicyclo[2.2.1]heptane-exo, exo-2,3-dicarboxylate

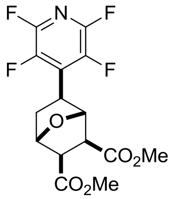

The **General procedure B** was followed using pentafluoropyridine (11 µL, 0.10 mmol, 1 equiv), dimethyl-7-oxabicyclo[2.2.1]hept-5-ene exo,exo-2,3-dicarboxylate (102 mg, 0.48 mmol, **4.8 equiv**), *N,N*-diisopropylethylamine (21 µL, 0.12 mmol, 1.2 equiv) and 1.0 mL of stock solution of $Ir(ppy)_3$ (0.16 mg, 0.00025 mmol, 0.0025 equiv) in CH_3CN was used. The crude material was purified by flash chromatography using hexane : ethyl acetate (0-12% EtOAc for 18 cv, ramped to 100% EtOAc and then held at 100% EtOAc for 18-28 cv), on a 4 g silica column to afford **S-16a** in 42% yield (15 mg, 0.04 mmol) as a colorless liquid. ^{19}F NMR (376 MHz, Chloroform-*d*) δ -90.58 – -90.88 (m), -142.32 – -142.55 (m). ^1H NMR (400 MHz, Chloroform-*d*) δ 5.13 (d, J = 5.5 Hz, 1H), 3.71 (s, 3H), 3.70 (s, 3H), 3.42 (dd, J = 9.0, 5.6 Hz, 1H), 3.21-3.10 (m, 1H), 2.29 – 2.00 (m, 1H). $^{13}C\{^1H\}$ NMR (151 MHz, Chloroform-*d*) δ 170.8, 170.3, 145.0 – 142.7 (m), 141.5 – 138.9 (m),

135.0 (t, J = 12.7 Hz), 81.4, 78.7, 52.6, 52.5, 52.5 51.6, 39.6, 37.5. FT-IR cm^{-1} 2960, 2925, 1722, 1653, 1438, 1377. GC/MS (m/z, relative intensity) 363 (M^+, 5), 332 (10), 304 (20), 272 (25), 59 (100). HRMS (ESI) Calcd. for $C_{15}H_{13}F_4NO_5$ $[M + Na]^+$ 386.0628, found 386.0609.
Note: The relevant coupling constant of the product matched that of the literature for the exo-isomer. The stereochemistry was assigned based on this coupling constant.[7]

Synthesis of S-17a 4-cyclohexyl-2,2',3,3',5,5',6,6'-octafluoro-1,1'-biphenyl

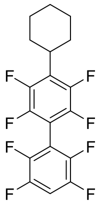

The **General procedure B** was followed using perfluoro-1,1'-biphenyl (33 mg, 0.1 mmol, 1 equiv), cyclohexene (60 µL, 0.6 mmol, 6.0 equiv), *N,N*-diisopropylethylamine (21 µL, 0.12 mmol, 1.2 equiv) and 1.0 mL of stock solution of $Ir(ppy)_3$ (0.16 mg, 0.00025 mmol, 0.0025 equiv) in CH_3CN was used. The crude material was purified by prep tlc using hexane to afford **S-17a** in 43% yield (17 mg, 0.04 mmol) as a white solid. ^{19}F NMR (376 MHz, Chloroform-*d*) δ -137.84 – -138.19 (m, 2F), -138.19 – -138.53 (m, 2F), -139.43 – -139.80 (m, 2F), -142.21 – -142.48 (m, 2F). ^1H NMR (400 MHz, Chloroform-*d*) δ 7.25 – 7.18 (m, 1H), 3.11 (tt, J = 10.9, 5.1 Hz, 1H), 1.97 – 1.73 (m, 8H), 1.52 – 1.30 (m, 2H). $^{13}C\{^1H\}$ NMR (101 MHz, Chloroform-*d*) δ 147.5 – 146.0 (m), 145.3 (dt, J = 12.0, 4.0 Hz), 145.0–143.5 (m), 143.2 – 142.5 (m), 127.3 (t, J = 16.3 Hz), 108.3 (ddt, J = 20.0, 17.0, 2.4 Hz), 107.5 (t, J = 22.2 Hz), 105.1 – 103.8 (m), 36.1 , 30.8 (t, J = 2.2 Hz), 26.8 , 25.8.). FT-IR cm^{-1}2936, 2858, 1506, 1449, 1240. GC/MS (m/z, relative intensity) 380 (M^+, 30), 324 (70), 41(100). HRMS (ESI) Calcd. for $C_{18}H_{12}F_8$ $[M + H]^+$ 381.0890, found 381.1062. Melting point 110-114 °C.

Synthesis of S-18a (ethyl 3-(2,3,5,6-tetrafluoro-4-(trifluoromethyl)phenyl)butanoate)

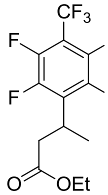

The **General procedure B** was followed using octafluorotoluene (28 µL, 0.20 mmol, 1 equiv), ethyl crotonate (149 µL, 1.2 mmol, 6 equiv), *N,N*-diisopropylethylamine (42 µL, 0.24 mmol, 1.2 equiv) and 2.0 mL of stock solution of $Ir(ppy)_3$ (0.3 mg, 0.0005 mmol, 0.0025 equiv) in CH_3CN was used. The crude material was purified by flash chromatography using hexane : ethyl acetate (0-20% EtOAc for 25 cv, ramped to 100% EtOAc and then held at 100% EtOAc for 25-35 cv) on a 12 g silica column to afford **S-18a** in 71% yield (47 mg, 0.14 mmol, rr 10:1) as a colorless liquid. ^{19}F NMR (376 MHz, Chloroform-*d*) δ -56.36 (t, J = 21.3 Hz, 3F), -140.90 – -141.06 (m, 2F), -141.07 – -141.33 (m, 2F). ^1H NMR (400 MHz, Chloroform-*d*) δ 4.02 (qd, J = 7.2, 3.1 Hz, 2H), 3.72 (dt, J = 8.7, 6.9 Hz, 1H), 2.87 – 2.61 (m, 2H), 1.33 (d, J = 7.2 Hz, 3H), 1.13 (t, J = 7.1 Hz, 3H). $^{13}C\{^1H\}$ NMR (101 MHz, Chloroform-*d*) δ 171.3, 147.1 – 143.7 (m), 146.7 – 142.4 (m), 128.1 (t, J = 15.3 Hz), 120.8 (q, J = 275.5, 275.1 Hz), 108.3 – 107.2 (m), 60.9, 39.2 (t, J = 2.5 Hz), 27.5, 19.2, 14.2. FT-IR cm^{-1} 2990, 2944, 1738, 1663, 1497, 1328. GC/MS (m/z, relative intensity) 332 (M^+, 20), 245 (75), 231 (80), 60 (100). HRMS (ESI) Calcd. for $C_{13}H_{11}F_7O_2$ $[M + H]^+$ 333.0726, found 333.1299.

Synthesis of S-19a (methyl 3-(4-cyano-2,3,5,6-tetrafluorophenyl)butanoate)

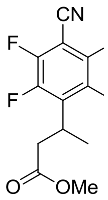

The **General procedure B** was followed using pentafluorobenzonitrile (50 µL, 0.40 mmol, 1 equiv), methyl crotonate (254 µL, 2.4 mmol, 6 equiv), *N,N*-diisopropylethylamine (83 µL, 0.48 mmol, 1.2 equiv) and 4.0 mL of stock solution of Ir(ppy)$_3$ (0.66 mg, 0.001 mmol, 0.0025 equiv) in CH_3CN was used. The crude material was purified by flash chromatography using hexane : ethyl acetate (0-2% EtOAc for 13 cv, ramped to 100 % EtOAc and then held at 100% EtOAc for 13-18 cv) on a 24 g silica column to afford **S-19a** in 54% yield (60 mg, 0.22 mmol) as a colorless liquid. ^{19}F NMR (376 MHz, Chloroform-*d*) δ -132.66 – -132.85 (m, 2F), -139.60 (td, *J* = 16.4, 7.2 Hz, 2F). ^{1}H NMR (400 MHz, Chloroform-*d*) δ 3.80 (dq, *J* = 13.6, 7.2 Hz, 1H), 3.64 (s, 3H), 3.00 – 2.70 (m, 2H), 1.40 (d, *J* = 7.2 Hz, 3H). ^{13}C{^{1}H} NMR (101 MHz, Chloroform-*d*) δ 171.7, 147.1 (ddt, *J* = 261.8, 17.3, 3.7 Hz), 144.91 (dddd, *J* = 249.3, 12.4, 7.1, 4.4 Hz), 130.7 (t, *J* = 15.8 Hz), 107.6, 92.4 (tt, *J* = 17.3, 2.5 Hz), 52.1, 38.8 (t, *J* = 2.8 Hz), 27.8, 19.2 (t, *J* = 2.0 Hz). FT-IR cm^{-1} 2995, 2856, 2253, 1738, 1492, 1462. GC/MS (m/z, relative intensity) 275 (M^{+}, 20), 202 (45), 188 (60), 74 (100), 59 (80). HRMS (ESI) Calcd. for $C_{13}H_{11}F_4NO_2$ [M + Na]$^{+}$ 312.0624, found 312.9055.

Synthesis of S-20a (methyl 3-(4-cyano-2,3,5,6-tetrafluorophenyl)butanoate)

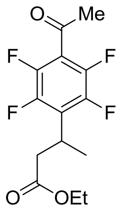

The **General procedure B** was followed using pentafluoroacetophenone (28 µL, 0.2 mmol, 1 equiv), ethyl crotonate (148 µL, 1.2 mmol, 6 equiv), *N,N*-diisopropylethylamine (42 µL, 0.12 mmol, 1.2 equiv) and 2.0 mL of stock solution of Ir(ppy)$_3$ (0.3 mg, 0.0005 mmol, 0.0025 equiv) in CH_3CN was used. The crude material was purified by flash chromatography using hexane : ether (0-7% EtOAc for 32 cv, ramped to 100% EtOAc and then held at 100% EtOAc for 32-40 cv) on a 12 g silica column to afford **S-20a** in 50% yield (30 mg, 0.1 mmol) as a brown liquid. ^{19}F NMR (376 MHz, Chloroform-*d*) δ -141.88 (dd, *J* = 21.7, 12.5 Hz, 2F), -142.18 (dd, *J* = 21.9, 12.6 Hz, 2F). ^{1}H NMR (400 MHz, Chloroform-*d*) δ 4.09 (qd, *J* = 7.1, 2.9 Hz, 2H), 3.77 (h, *J* = 7.3 Hz, 1H), 2.89 – 2.68 (m, 2H), 2.61 (s, 3H), 1.39 (d, *J* = 7.2 Hz, 3H), 1.20 (t, *J* = 7.1 Hz, 3H). ^{13}C{^{1}H} NMR (101 MHz, Chloroform-*d*) δ 192.3, 171.4, 146.6 – 144.9 (m), 144.0 – 142.4 (m), 126.7 (t, *J* = 15.8 Hz), 117.8 (t, *J* = 16.3 Hz), 60.8, 39.4 (t, *J* = 2.7 Hz), 32.5 (t, *J* = 2.5 Hz), 27.4, 19.3 (t, *J* = 1.9 Hz), 14.2. FT-IR cm^{-1} 2984, 2941, 1736, 1711, 1481. GC/MS (m/z, relative intensity) 306 (M^{+}, 25), 219 (30), 43 (100). HRMS (ESI) Calcd. for $C_{14}H_{14}F_4O_3$ [M + H]$^{+}$ 307.0957, found 307.1149.

Synthesis of S-21a (2,3,5,6-tetrafluoro-4-(2-methylcyclohexyl)benzonitrile)

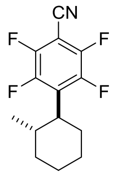

The **General procedure B** was followed using pentafluorobenzonitrile (12 μL, 0.10 mmol, 1 equiv), 1-methyl -1- cyclohexene (72 μL, 0.6 mmol, 6 equiv), *N,N*-diisopropylethylamine (21 μL, 0.12 mmol, 1.2 equiv) and 1.0 mL of stock solution of Ir(ppy)$_3$ (0.16 mg, 0.00025 mmol, 0.0025 equiv) in CH_3CN was used. The crude material was purified by flash chromatography using hexane : ethyl acetate (0-3% EtOAc for 13 cv, ramped to 100 % EtOAc and then held at 100% EtOAc for 13-18 cv) on a 12 g silica column to afford **S-21a** in 41% yield (11 mg, 0.04 mmol, dr 5.7:1) as a colorless liquid. ^{19}F NMR (376 MHz, Chloroform-*d*) δ -133.02 – -133.32 (m, 2F), -138.12 – -139.97 (m, 2F). ^{1}H NMR (400 MHz, Chloroform-*d*) δ 2.75 (td, *J* = 11.5, 3.8 Hz, 1H), 2.00 – 1.69 (m, 6H), 1.40 – 1.24 (m, 2H), 1.14 – 1.02 (m, 1H), 0.73 (d, *J* = 6.5 Hz, 3H). ^{13}C{^{1}H} NMR (101 MHz, Chloroform-*d*) δ 147.1 (ddt, *J* = 262.3, 18.2, 3.9 Hz), 146.3 – 143.5 (m), 131.8 (t, *J* = 16.5 Hz), 107.8 (t, *J* = 3.7 Hz), 92.0 – 91.4 (m), 43.6, 35.6, 34.9 (t, *J* = 2.3 Hz), 30.9, 26.5, 26.1, 208. FT-IR cm^{-1} 2957, 2931, 2247, 1653, 1494. GC/MS (m/z, relative intensity) 271 (M$^+$, 20), 201 (40), 70 (90), 55 (100). HRMS (ESI) Calcd. for $C_{14}H_{13}F_4N$ [M + Na]$^+$ 294.0882, found 294.1669.

Note: the isolated product has 15% of the minor diastereomer. The major diastereomer was determined from the ^{1}H-^{1}H coupling constants. Assuming a chair conformer with the aryl group equatorial, for the trans isomer (major) the benzylic C–H couples to 3-^{1}H's with dihedral angles of 177°, 179°, 63° whereas for the cis-isomer (minor), the dihedral angle was found 58.8°, 177°, 64°. Consequently, the trans isomer appears as a td whereas the cis isomer gives a dt.

Synthesis of S-22a (7-cyclohexyl-4,5,6-trifluoro-2-phenylbenzofuran)

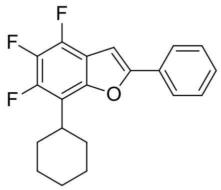

The **General procedure B** was followed using 4,5,6,7-tetrafluoro-2-phenylbenzofuran (27 mg, 0.10 mmol, 1 equiv), cyclohexene (60 µL, 1.2 mmol, 6 equiv), *N,N*-diisopropylethylamine (21 µL, 0.12 mmol, 1.2 equiv) and 1.0 mL of stock solution of Ir(ppy)$_3$ (0.16 mg, 0.00025 mmol, 0.0025 equiv) in CH_3CN was used. The crude material was purified by prep tlc using hexane to afford **S-22a** in 58% yield (19 mg, 0.06 mmol) as white solid. ^{19}F NMR (376 MHz, Chloroform-*d*) δ -142.36 (d, *J* = 19.4 Hz, 1F), -146.56 (d, *J* = 20.6 Hz, 1F), -149.88 (t, *J* = 20.1 Hz, 1F). ^{1}H NMR (400 MHz, Chloroform-*d*) δ 7.86 (d, *J* = 7.2 Hz, 2H), 7.47 (t, *J* = 7.4 Hz, 2H), 7.40 (t, *J* = 7.3 Hz, 1H), 7.09 (d, *J* = 2.8 Hz, 1H), 3.11 (tt, *J* = 11.9, 3.3 Hz, 1H), 2.03 – 1.72 (m, 7H), 1.50 – 1.27 (m, 3H). ^{13}C{^{1}H} NMR (101 MHz, Chloroform-*d*) δ 157.7 , 144.7 (ddd, *J* = 240.2, 12.5, 7.1 Hz), 143.1 – 140.2 (m), 139.2 – 138.1 (m), 129.5 , 129.4 , 129.1 , 125.5 , 125.3 , 120.3 – 119.7 (m), 118.6 (dt, *J* = 20.3, 3.4 Hz), 101.4 – 100.4 (m), 98.1 – 97.9 (m), 97.7– 97.4 (m), 35.9, 31.5, 27.1, 26.0. FT-IR cm^{-1} 3150, 3062, 2939, 2853, 1511, 1489, 1446. GC/MS (m/z, relative intensity) 330 (M$^+$, 100), 287 (80), 261 (90), 41 (90). HRMS (ESI) Calcd. for $C_{20}H_{17}F_3O$ [M + H]$^+$ 331.1310, found 331.1498.
Melting point 100-105 ^{o}C

Note: the isolated product contains 5% minor regioisomer by GC-MS. The regiochemistry was assigned according to multiplicity of peaks on ^{19}F NMR.

Synthesis of S-23a 2-(2,4,5-trifluoro-3-phenoxy-6-(trifluoromethyl)phenyl)exo-bicyclo[2.2.1]heptane

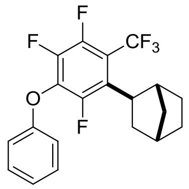

The **General procedure B** was followed using 1,2,4,5-tetrafluoro-3-phenoxy-6-(trifluoromethyl)benzene (93 mg, 0.30 mmol, 1 equiv), 2-norbornene (169 mg, 1.8 mmol, 6 equiv), *N,N*-diisopropylethylamine (63 µL, 0.36 mmol, 1.2 equiv) and 3.0 mL of stock solution of Ir(ppy)$_3$ (0.5 mg, 0.0008 mmol, 0.0025 equiv) in CH_3CN was used. The crude material was purified by prep tlc using hexane to afford **S-23a** in 52% yield (61 mg, 0.16 mmol) as a colorless liquid. ^{19}F NMR (376 MHz, Chloroform-*d*) δ -52.63 (d, *J* = 37.7 Hz, 3F), -124.76 (d, *J* = 10.8 Hz, 1F), -138.96 (qdd, *J* = 37.8, 20.8, 11.9 Hz, 1F), -148.97 (dd, *J* = 20.8, 3.7 Hz, 1F). ^{1}H NMR (400 MHz, Chloroform-*d*) δ 7.34 (t, *J* = 8.0 Hz, 2H), 7.13 (t, *J* = 7.4 Hz, 1H), 6.95 (d, *J* = 8.1 Hz, 2H), 3.01 (t, *J* = 8.2 Hz, 1H), 2.36 (s, 2H), 1.91-1.71 (m, 3H), 1.57 (dddt, *J* = 27.1, 15.7, 8.4, 3.6 Hz, 2H), 1.36 (t, *J* = 9.9 Hz, 1H), 1.32 – 1.18 (m, 2H). ^{13}C{^{1}H} NMR (101 MHz, Chloroform-*d*) δ 157.1 , 151.2 (dt, *J* = 249.4, 3.2 Hz), 148.0 – 145.0 (m), 143.4 (ddd, *J* = 253.4, 16.6, 5.1 Hz), 136.0 (ddd, *J* = 19.0, 11.8, 3.1 Hz), 130.0 , 129.7 (dd, *J* = 15.1, 4.1 Hz), 124.0 – 123.7 (m), 123.9 , 115.5 , 114.4 (dt, *J* = 31.1, 6.4 Hz), 43.6 (d, *J* = 2.5 Hz), 42.2 , 39.6 (d, *J* = 4.3 Hz), 37.7 (d, *J* = 12.5 Hz), 37.0 , 32.3 , 27.8 (d, *J* = 2.9 Hz). FT-IR cm^{-1} 2963, 2874, 1636, 1588, 1494, 1312. GC/MS (m/z, relative intensity) 386 (M$^+$, 15), 318 (80), 77 (100), 67 (60). HRMS (ESI) Calcd. for $C_{20}H_{16}F_6O$ [M + H]$^+$ 387.1184, found 387.1780.

Note: isolated product contains 5% ((2,4-difluoro-3-phenoxy-6-(trifluoromethyl)phenyl)exo-bicyclo[2.2.1]heptane), a product of subsequent hydrodefluorination of **S-23a**.

Synthesis of S-24a methyl 2-(exo-bicyclo[2.2.1]heptan-2-yl)-3,5,6-trifluoro-4-(2-methoxy-2-oxoethyl)benzoate

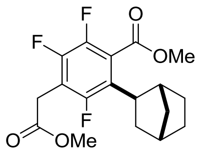

The **General procedure B** was followed using methyl 2,3,5,6-tetrafluoro-4-(2-methoxy-2-oxoethyl)benzoate (168 mg, 0.60 mmol, 1 equiv), 2-norbornene (341 mg, 3.6 mmol, 6 equiv), *N,N*-diisopropylethylamine (125 µL, 0.72 mmol, 1.2 equiv) and 6.0 mL of stock solution of Ir(ppy)$_3$ (0.98 mg, 0.0015 mmol, 0.0025 equiv) in CH_3CN was used. The crude material was purified by flash chromatography using hexane : ether (0-21% ether for 40 cv, ramped to 100% ether and then held at 100% ether for 40-50 cv) on a 12 g silica column to afford **S-24a** in 60% yield (128 mg, 0.36 mmol) as a colorless liquid. ^{19}F NMR (376 MHz, Chloroform-*d*) δ -115.92 (d, *J* = 14.9 Hz, 1F), -139.56 (dd, *J* = 22.4, 3.4 Hz, 1F), -144.48 (dd, *J* = 22.5, 14.9 Hz, 1F). ^{1}H NMR (400 MHz, Chloroform-*d*) δ 3.95 (s, 3H), 3.73 (s, 3H), 3.71 (s, 2H), 2.64 (dd, *J* = 9.1, 6.7 Hz, 1H), 2.45 (s, 1H), 2.33 (s, 1H), 1.81 (m,

2H), 1.70 (dd, *J* = 20.8, 11.2 Hz, 1H), 1.60 – 1.56 (m, 1H), 1.30 – 1.14 (m, 4H). $^{13}C\{^1H\}$ NMR (101 MHz, Chloroform-*d*) δ 169.4 , 165.0(t, *J* = 3.3 Hz), 154.4 (ddd, *J* = 246.8, 5.1, 2.9 Hz), 146.8 (ddd, *J* = 249.7, 14.5, 8.5 Hz), 143.9 (ddd, *J* = 247.7, 14.5, 3.5 Hz), 126.9 (dd, *J* = 17.5, 4.5 Hz), 123.7 (dd, *J* = 15.0, 8.1 Hz), 114.3 (dd, *J* = 24.9, 15.7 Hz), 53.1, 52.7, 44.0 , 42.7 (d, *J* = 3.1 Hz), 39.2 (d, *J* = 3.9 Hz), 37.4 (d, *J* = 8.6 Hz), 36.8 , 31.8 , 28.6 – 28.3 (m), 28.3 (d, *J* = 2.1 Hz). FT-IR cm^{-1} 2957, 2874, 1741, 1481, 1438, 1363. GC/MS (m/z, relative intensity) 356 (M^+, 5), 288 (20), 81 (55), 59 (100). HRMS (ESI) Calcd. for $C_{18}H_{19}F_3O_4$ $[M + Na]^+$ 379.1133, found 379.1116.

Synthesis of S-25a N-(3-(exo-bicyclo[2.2.1]heptan-2-yl)-2,5,6-trifluoro-4-(trifluoromethyl)phenyl)acetamide

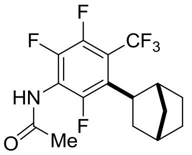

The **General procedure B** was followed using *N*-(2,3,5,6-tetrafluoro-4-(trifluoromethyl)phenyl)acetamide (28 mg, 0.10 mmol, 1 equiv), 2-norbornene (56 mg, 0.6 mmol, 6 equiv), *N,N*-diisopropylethylamine (21 µL, 0.12 mmol, 1.2 equiv) and 1.0 mL of stock solution of $Ir(ppy)_3$ (0.16 mg, 0.00025 mmol, 0.0025 equiv) in CH_3CN was used. The crude material was purified by flash chromatography using hexane : ethyl acetate (0-15% EtOAc for 35 cv, ramped to 100% EtOAc and then held at 100% EtOAc for 35-45 cv) on a 12 g silica column to afford **S-25a** in 54% yield (19 mg, 0.05 mmol) as a colorless liquid. ^{19}F NMR (376 MHz, Chloroform-*d*) δ -52.91 (d, *J* = 37.7 Hz, 3F), -118.03 – -118.21 (m, 1F), -138.19 (s, 1F), -139.52 – -140.05 (m, 1F). ^1H NMR (400 MHz, Chloroform-*d*) δ 6.94 (s, 1H), 2.97 (t, *J* = 8.2 Hz, 1H), 2.38 (t, *J* = 3.9 Hz, 1H), 2.33 (s, 1H), 2.24 (s, 3H), 1.91 – 1.79 (m, 2H), 1.74 (t, *J* = 10.7 Hz, 1H), 1.67 – 1.47 (m, 2H), 1.41 – 1.18 (m, 3H). $^{13}C\{^1H\}$ NMR (101 MHz, Chloroform-*d*) δ 168.0 , 151.3 (dt, *J* = 246.6, 3.2 Hz), 147.6 – 144.5 (m), 144.26 (ddd, *J* = 253.3, 16.5, 5.5 Hz), 128.9 – 1287 (m), 122.9 (dq, *J* = 275.4, 3.0, 2.6 Hz), 119.5 – 118.9 (m), 116.7 – 115.7 (m), 43.5 (d, *J* = 2.4 Hz), 42.0, 39.6 (d, *J* = 4.3 Hz), 37.7 (d, *J* = 12.6 Hz), 37.0, 32.3 , 27.9 (d, *J* = 2.9 Hz), 23.2. FT-IR cm^{-1} 3241, 3199, 2963, 2874, 1685, 1634, 1524, 1478, 1374. GC/MS (m/z, relative intensity) 351 (M^+, 5), 284 (30), 241 (40), 43 (100). HRMS (ESI) Calcd. for $C_{16}H_{15}F_6NO$ $[M + H]^+$ 352.1136, found 352.1801. Melting point 131-134 ^oC.

Synthesis of S-26a methyl 2-(exo-bicyclo[2.2.1]heptan-2-yl)-4-(2-((2-ethoxy-2-oxoethyl)amino)-2-oxoethyl)-3,5,6-trifluorobenzoate

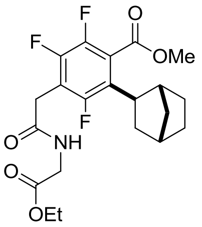

The **General procedure B** was followed except that higher catalyst loading (0.5 mol%) was used, using methyl 4-(2-((2-ethoxy-2-oxoethyl)amino)-2-oxoethyl)-2,3,5,6-tetrafluorobenzoate (140 mg, 0.40 mmol, 1 equiv), 2-norbornene (231 mg, 2.4 mmol, 6 equiv), N,N-diisopropylethylamine (84 µL, 0.48 mmol, 1.2 equiv) and 4.0 mL of stock solution of Ir(ppy)3 (1.3 mg, 0.002 mmol, 0.005 equiv) in CH3CN was used. The crude material was purified by flash chromatography using hexane : ethyl acetate (0-18% EtOAc for 50 cv, ramped to 100% EtOAc

and then held at 100% EtOAc for 50-60 cv) on a 24 g silica column to afford S-26a in 39% yield (67 mg, 0.16 mmol) as a yellow viscous liquid. ^{19}F NMR (376 MHz, Chloroform-d) δ -115.62 (d, J = 14.9 Hz, 1F), -139.10 (dd, J = 22.5, 3.1 Hz, 1F), -144.01 (dd, J = 22.6, 14.9 Hz, 1F). ^{1}H NMR (400 MHz, Chloroform-d) δ 6.10 (s, 1H), 4.22 (q, J = 7.1 Hz, 2H), 4.04 (d, J = 5.1 Hz, 2H), 3.94 (s, 3H), 3.66 (s, 2H), 2.64 (dd, J = 9.6, 6.3 Hz, 1H), 2.45 (s, 1H), 2.33 (s, 1H), 1.83 – 1.63 (m, 3H), 1.55 (dd, *J* = 8.5, 3.2 Hz, 2H), 1.28 (t, *J* = 7.1 Hz, 3H), 1.21 (dd, *J* = 8.9, 2.6 Hz, 3H). ^{13}C{^{1}H} NMR (101 MHz, Chloroform-*d*) δ 169.7 , 167.8 , 165.0 (t, *J* = 3.6 Hz), 154.4 (dq, *J* = 247.0, 2.8 Hz), 146.7 (ddd, *J* = 249.5, 14.3, 8.4 Hz), 143.9 (ddd, *J* = 248.3, 14.5, 3.6 Hz), 127.1 (dd, *J* = 17.5, 4.5 Hz), 123.9 (dd, *J* = 15.2, 7.8 Hz), 114.6 (dd, *J* = 25.0, 15.8 Hz), 61.8, 53.1, 44.0(d, *J* = 2.4 Hz), 42.6 (d, *J* = 3.2 Hz), 41.8, 39.2 (d, *J* = 3.8 Hz), 37.4 (d, *J* = 8.5 Hz), 36.8, 31.8, 30.4 – 30.3 (m), 28.2 (d, *J* = 2.2 Hz), 14.2. FT-IR cm^{-1} 3312, 2594, 1745, 1664, 1484, 1301, 1202. GC/MS (m/z, relative intensity) 427 (M^{+}, 10), 283 (20), 104 (100), 67 (100). HRMS (ESI) Calcd. for $C_{21}H_{24}F_3NO_5$ [M + H]$^{+}$ 428.1685, found 428.1664.

Note: isolated product contains two minor products. The first is a minor regioisomer, 5%, which underwent alkylation meta to the methyl ester. The second compound, 9%, is (methyl 2-bicyclo[2.2.1]heptan-2-yl)-4-(2-((2-ethoxy-2-oxoethyl)amino)-2-oxoethyl)-3,5-difluorobenzoate) which is the product of subsequent hydrodefluorination of **S-26a** by ^{19}F NMR.

Synthesis of S-27a ethyl (2-(2-(exo-bicyclo[2.2.1]heptan-2-yl)-3,5,6-trifluoropyridin-4-yl)acetyl)glycinate

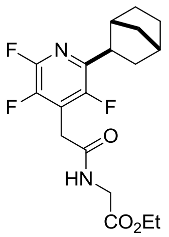

The **General procedure B** was followed **except that higher catalyst loading (0.5 mol%) was used**, using ethyl 2-(2-(perfluoropyridin-4-yl)acetamido)acetate (88 mg, 0.3 mmol, 1 eq), 2-norbornene (170 mg, 1.8 mmol, 6 equiv), *N*,*N*-diisopropylethylamine (63 µL, 0.36 mmol, 1.2 eq) and 3.0 mL of stock solution of Ir(ppy)$_3$ (0.98 mg, 0.0015 mmol, 0.005 equiv) in CH_3CN was used. The crude material was purified by flash chromatography using hexane : ethyl acetate (0-20 % EtOAc for 55 cv, ramped to 100% EtOAc and then held at 100% EtOAc for 55-65 cv) on a 24 g silica column to afford **S-27a** in 61% yield (68 mg, 0.18 mmol) as a colorless liquid. ^{19}F NMR (376 MHz, Chloroform-*d*) δ -68.71 (dd, *J* = 28.6, 12.2 Hz, 1F), -91.54 (dd, *J* = 23.5, 12.4 Hz, 1F), -146.89 (ddt, *J* = 28.8, 23.9, 2.6 Hz, 1F). ^{1}H NMR (400 MHz, Chloroform-*d*) δ 6.24 (s, 1H), 4.21 (q, *J* = 7.1 Hz, 2H), 4.02 (d, *J* = 5.1 Hz, 2H), 3.79 (s, 2H), 2.75 (t, *J* = 7.9 Hz, 1H), 2.44 (s, 1H), 2.35 (s, 1H), 1.84-1.67 (m, 3H), 1.57 (dt, *J* = 10.0, 3.1 Hz, 2H), 1.27 (t, *J* = 7.2 Hz, 3H), 1.2 (m, 3H). ^{13}C{^{1}H} NMR (101 MHz, Chloroform-*d*) δ 169.6 , 167.2 , 152.5 (ddd, *J* = 244.7, 11.5, 2.4 Hz), 146.4 (ddd, *J* = 243.5, 18.2, 16.2 Hz), 141.6 (ddd, *J* = 251.7, 25.4, 6.1 Hz), 138.5 (ddd, *J* = 11.4, 7.4, 2.3 Hz), 125.5 (ddd, *J* = 27.7, 6.7, 1.5 Hz), 61.9, 42.0 (d, *J* = 4.2 Hz), 41.93 (d, *J* = 5.1 Hz), 41.8, 38.9 (d, *J* = 3.7 Hz), 37.6 (d, *J* = 8.7 Hz), 36.5, 33.7 (q, *J* = 2.7 Hz), 31.7, 28.3 (d, *J* = 2.2 Hz), 14.2. GC/MS (m/z, relative intensity) 370 (M^{+}, 12), 211(25), 104 (100), 67 (100), 41

(100). FT-IR cm^{-1} 32495, 3091, 2965, 1753, 1656, 1624, 1562, 1468. HRMS (ESI) Calcd. for $C_{18}H_{21}F_3N_2O_3$ [M + H]$^+$ 371.1583, found 371.1567.

Synthesis of S-28a dimethyl 5-(4-(2-((2-ethoxy-2-oxoethyl)amino)-2-oxoethyl)-3,5,6-trifluoropyridin-2-yl)-7-exo-oxabicyclo[2.2.1]heptane-exo, exo-2,3-dicarboxylate

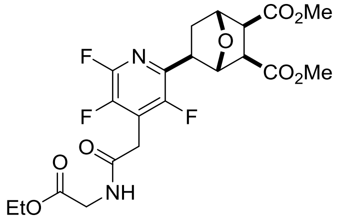

The **General procedure B** was followed **except that higher catalyst loading (0.5 mol%) was used,** using methyl 2-(perfluoropyridin-4-yl)acetate (88 mg, 0.30 mmol, 1 equiv), dimethyl-7-oxabicyclo[2.2.1]hept-5-ene exo,exo-2,3-dicarboxylate (305 mg, 1.4 mmol, 4.8 equiv), *N,N*-diisopropylethylamine (63 µL, 0.36 mmol, 1.2 equiv) and 3.0 mL of stock solution of Ir(ppy)$_3$ (0.98 mg, 0.0015 mmol, 0.005 equiv) in CH_3CN was used. The crude material was purified by flash chromatography using hexane with triethyl amine : ethyl acetate (0-40% EtOAc for 100 cv, ramped to 100% EtOAc and then held at 100% EtOAc for 100-120 cv) on a 24 g silica column to afford **S-28a** in 61% yield (89 mg, 0.18 mmol) as a yellow viscous liquid. ^{19}F NMR (376 MHz, Chloroform-*d*) δ -72.14 (dd, *J* = 27.9, 12.5 Hz, 1F), -90.22 (dd, *J* = 22.9, 12.5 Hz, 1F), -145.88 – -146.12 (m, 1F). ^{1}H NMR (400 MHz, Chloroform-*d*) δ 6.43 (t, *J* = 5.4 Hz, 1H), 5.14 (d, *J* = 4.9 Hz, 1H), 4.99 (s, 1H), 4.20 (q, *J* = 7.1 Hz, 2H), 4.12 – 3.91 (m, 4H), 3.68 (s, 3H), 3.65 (s, 3H), 3.34 (dd, *J* = 9.5, 6.4 Hz, 1H), 3.26 – 3.09 (m, 2H), 2.15 (dd, *J* = 12.7, 9.3 Hz, 1H), 1.96 (dt, *J* = 12.3, 5.9 Hz, 1H), 1.27 (t, *J* = 7.2 Hz, 3H). ^{13}C{^{1}H} NMR (101 MHz, Chloroform-*d*) δ 171.5 , 170.8 , 169.6 , 167.8 , 153.0(ddd, *J* = 243.8, 12.0, 2.5 Hz), 149.0 – 145.2 (m), 142.1 (ddd, *J* = 252.0, 24.7, 5.8 Hz), 138.6 (dq, *J* = 12.7, 2.5 Hz), 122.0 (dd, *J* = 27.2, 6.7 Hz), 82.3 , 78.9 , 61.7 , 53.4 , 52.4 , 52.3 , 51.1 , 41.7 , 40.1 , 38.7 , 33.2 – 33.0 (m), 14.2.). FT-IR cm^{-1} 3293, 3105, 2997, 1742, 1635, 1567, 1481, 1430, 1374, 1202. HRMS (ESI) Calcd. for $C_{21}H_{23}F_3N_2O_8$ [M + Na]$^+$ 511.1304, found 511.1346.

Synthesis of S-29a dimethyl 5-(4-(2-((2-ethoxy-2-oxoethyl)amino)-2-oxoethyl)-2,5,6-trifluoropyridin-3-yl)-7-exo-oxabicyclo[2.2.1]heptane-exo, exo-2,3-dicarboxylate

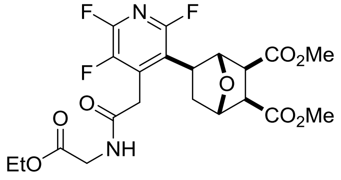

The **General procedure B** was followed **except catalyst *fac*-tris(2-4- *tert*-butylphenyl pyridinato-C^2, *N*) Iridium(III) (Ir(*t*buppy)$_3$ (0.5 mol%) was used,** using ethyl (2-(3-chloro-2,5,6-trifluoropyridin-4-yl)acetyl)glycinate (16 mg, 0.050 mmol, 1 equiv), dimethyl-7-oxabicyclo[2.2.1]hept-5-ene exo,exo-2,3-dicarboxylate (51 mg, 0.24 mmol, 4.8 equiv), *N,N*-diisopropylethylamine (10 µL, 0.06 mmol, 1.2 equiv) and 0.5 mL of stock solution of Ir(*t*buppy)$_3$ (0.2 mg, 0.00025 mmol, 0.005 equiv) in CH_3CN was used. The crude material was purified by flash chromatography using hexane with 1% triethyl amine : ethyl acetate (0-45% EtOAc for 50 cv, ramped to 100% EtOAc and then held at 100% EtOAc for 50-70 cv) on a 24 g silica column to afford **S-29a** in 50% yield (12 mg, 0.025 mmol) as a viscous

yellow liquid. ^{19}F NMR (376 MHz, Acetone-d_6) δ -71.83 (dd, *J* = 27.9, 12.9 Hz, 1F), -94.58 (dd, *J* = 23.5, 12.9 Hz, 1F), -148.98 – -149.17 (m, 1F). ^{1}H NMR (400 MHz, Acetone-d_6) δ 7.80 (s, 1H), 4.98 (d, *J* = 5.0 Hz, 1H), 4.89 (s, 1H), 4.15 (q, *J* = 7.2 Hz, 2H), 4.10 (dd, *J* = 6.3, 2.4 Hz, 2H), 3.98 (d, *J* = 5.7 Hz, 2H), 3.60 (s, 3H), 3.58 (s, 3H), 3.46 (dd, *J* = 9.1, 6.4 Hz, 1H), 3.40 – 3.24 (m, 2H), 2.25 (dd, *J* = 12.4, 9.3 Hz, 1H), 1.92 (dt, *J* = 11.8, 5.9 Hz, 1H), 1.23 (t, *J* = 7.1 Hz, 3H). ^{13}C{^{1}H} NMR (101 MHz, Acetone-d_6) δ 172.2 , 171.6, 170.4, 168.6, 153.7(ddd, *J* = 244.9, 12.3, 2.1 Hz), 147.4(ddd, *J* = 240.4, 18.3, 16.2 Hz), 144.6 – 141.5 (m), 141.3 (dd, *J* = 6.1, 2.0 Hz), 124.3 (dd, *J* = 27.9, 6.5 Hz), 83.2 (d, *J* = 1.8 Hz), 79.4 , 61.5, 53.7 , 51.9, 51.9, 51.5 , 41.9, 41.2 (d, *J* = 4.0 Hz), 38.7(d, *J* = 1.9 Hz), 33.5(d, *J* = 3.5 Hz), 14.4. FT-IR cm^{-1} 3301, 3089, 3003, 2922, 1742, 1653, 1637, 1557, 1481, 1433, 1377, 1213. HRMS (ESI) Calcd. for $C_{21}H_{23}F_3N_2O_8$ $[M + Na]^+$ 511.1304, found 511.1330.

Synthesis of S-30a methyl (1'R,3'S)-2,3,5,6-tetrafluoro-3'-isopropyl-6'-methyl-1',2',3',4'-tetrahydro-[1,1'-biphenyl]-4-carboxylate

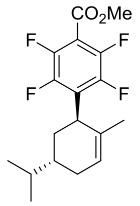

The **General procedure B** was followed using methyl 2,3,4,5,6-pentafluorobenzoate (46 mg, 0.2 mmol, 1 equiv), α-pinene (192 µL, 1.2 mmol, 6.0 equiv), *N*,*N*-diisopropylethylamine (42 µL, 0.24 mmol, 1.2 equiv) and 2.0 mL of stock solution of Ir(ppy)$_3$ (0.3 mg, 0.0005 mmol, 0.0025 equiv) in CH_3CN was used. The crude material was purified by flash chromatography using hexane : ethyl acetate (0-3% EtOAc for 15 cv, ramped to 100 % EtOAc and then held at 100% EtOAc for 15-25 cv) on a 12 g silica column and then by reverse phase flash chromatography using MeCN: isopropanol (0-3% isopropanol for 8 cv, ramped and then held at 100% isopropanol for 8-18 cv) on a C18 26 g column to afford **S-30a** in 54% yield (37 mg, 0.11 mmol) as a colorless liquid. ^{19}F NMR (376 MHz, Chloroform-*d*) δ -140.13 – -140.31 (m, 2F), -141.95 – -142.17 (m, 2F). ^{1}H NMR (400 MHz, Chloroform-*d*) δ 5.62 (td, *J* = 3.2, 1.7 Hz, 1H), 3.97 (s, 3H), 3.74 (t, *J* = 5.5 Hz, 1H), 2.29 – 2.05 (m, 1H), 1.99 – 1.68 (m, 3H), 1.57 (s, 3H), 1.49 (dd, *J* = 13.6, 6.9 Hz, 1H), 0.95 – 0.81 (m, 6H). ^{13}C{^{1}H} NMR (101 MHz, Chloroform-*d*) δ 160.6 , 146.9 – 143.7 (m), 146.0 – 142.8 (m), 130.6 , 127.5 (t, *J* = 15.2 Hz), 124.6 , 124.5 , 110.4 (td, *J* = 15.6, 4.5 Hz), 53.3 , 40.5 , 36.5 , 33.4 , 30.8 , 28.7 , 22.3 , 20.4 , 20.1.). FT-IR cm^{-1} 2957, 2923, 1744, 1650, 1478, 1436, 1304, 1224. GC/MS (m/z, relative intensity) 344 (M^+, 10), 301 (20), 95 (40), 41 (100). HRMS (ESI) Calcd. for $C_{18}H_{20}F_4O_2$ $[M + H]^+$ 345.1478, found 345.1299. $[\alpha]_D^{25}$= 53 (c 0. 75, CH_2Cl_2).

Synthesis of S-31a dimethyl 5-(4-(2-((2-ethoxy-2-oxoethyl)amino)-2-oxoethyl)-3,5-difluoropyridin-2-yl)-7-exo-oxabicyclo[2.2.1]heptane-exo, exo-2,3-dicarboxylate

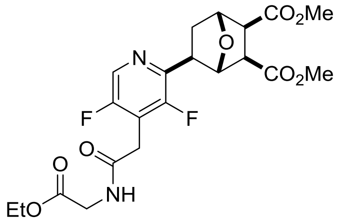

The **General procedure E** was followed using dimethyl 5-(4-(2-((2-ethoxy-2-oxoethyl)amino)-2-oxoethyl)-3,5,6-trifluoropyridin-2-yl)-7-oxabicyclo[2.2.1]heptane-exo, exo-2,3-dicarboxylate (30 mg, 0.061 mmol, 1 equiv), *N,N*-diisopropylethylamine (31 µL, 0.18 mmol, 3 equiv) and 0.6 mL of stock solution of Ir(ppy)$_3$ (0.5 mg, 0.0008 mmol) in CH_3CN was used. The crude material (crude shows 1.5:1 regioisomer by ^{19}F NMR) was purified by flash chromatography using hexane with triethyl amine : ethyl acetate (0-65% EtOAc for 55 cv, ramped to 100% EtOAc and then held at 100% EtOAc for 55-65 cv) on a 4 g silica column to afford **S-31a** in 93% yield (26 mg, 0.055 mmol) as a pale yellow viscous liquid. ^{19}F NMR (376 MHz, Acetone-*d*$_6$) δ -70.46 (d, *J* = 29.0 Hz, 1F), -135.75 (d, *J* = 29.0 Hz, 1F). ^{1}H NMR (400 MHz, Acetone-*d*$_6$) δ 7.95 (d, *J* = 1.9 Hz, 1H), 7.78 (t, *J* = 6.0 Hz, 1H), 4.98 (d, *J* = 5.0 Hz, 1H), 4.89 (s, 1H), 4.14 (q, *J* = 7.2 Hz, 2H), 4.05 – 3.94 (m, 4H), 3.60 (s, 3H), 3.58 (s, 3H), 3.48 (dd, *J* = 9.4, 6.3 Hz, 1H), 3.41 – 3.25 (m, 2H), 2.24 (dd, *J* = 12.3, 9.2 Hz, 1H), 1.92 (dt, *J* = 11.9, 5.7 Hz, 1H), 1.22 (t, *J* = 7.1 Hz, 3H). ^{13}C{^{1}H} NMR (151 MHz, Acetone-*d*$_6$) δ 172.2 , 171.6 , 170.5 , 169.1 , 158.3 (d, *J* = 236.2 Hz), 157.6 (dd, *J* = 248.1, 3.9 Hz), 137.1 (dd, *J* = 15.7, 6.2 Hz), 132.0 (dd, *J* = 29.7, 17.6 Hz), 127.2 (d, *J* = 31.1 Hz), 83.2 , 79.4 , 61.5 , 53.9, 51.9, 51.9 , 51.5 , 41.9, 41.6 (d, *J* = 3.9 Hz), 38.8 , 33.0 , 14.5. . FT-IR cm^{-1} 3371, 2986, 1742, 1688, 1538, 1414. HRMS (ESI) Calcd. for $C_{21}H_{24}F_2N_2O_8$ $[M + Na]^+$ 493.1398, found 493.1362.

Note: the stereochemical assignment was made on the basis of coupling constant value that matched with the literature[8] value of this bicyclic system.

Synthesis of S-32a dimethyl 5-(4-(2-((2-ethoxy-2-oxoethyl)amino)-2-oxoethyl)-2,5-difluoropyridin-3-yl)-7-exo-oxabicyclo[2.2.1]heptane-exo, exo-2,3-dicarboxylate

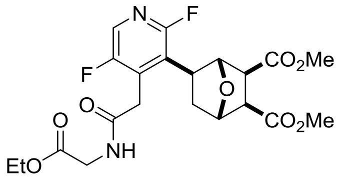

The **General procedure E** was followed using dimethyl 5-(4-(2-((2-ethoxy-2-oxoethyl)amino)-2-oxoethyl)-2,5,6-trifluoropyridin-3-yl)-7-oxabicyclo[2.2.1]heptane-exo, exo-2,3-dicarboxylate (20 mg, 0.041 mmol, 1 equiv), *N,N*-diisopropylethylamine (21 µL, 0.12 mmol, 3 equiv) and 0.4 mL of stock solution of Ir(ppy)$_3$ (0.3 mg, 0.0005 mmol) in CH_3CN was used. The crude material was purified by flash chromatography using hexane with triethyl amine : ethyl acetate (0-70% EtOAc for 25 cv, ramped to 100% EtOAc and then held at 100% EtOAc for 25-35 cv) on a 4 g silica column to afford **S-32a** in 95% yield (18 mg, 0.038 mmol, 1:1.5 rr) as a pale yellow viscous liquid. ^{19}F NMR (376 MHz, Acetone-*d*$_6$) δ -70.43 (dd, *J* = 29.0, 9.9 Hz, 1F), -135.73 (d, *J* = 28.6 Hz, 1F). ^{1}H NMR (400 MHz, Acetone-*d*$_6$) δ 7.94 (s, 1H), 7.81 – 7.73 (m, 1H), 4.98 (d, *J* = 4.9 Hz, 1H), 4.89 (s, 1H), 4.14 (q, *J* = 7.1 Hz, 2H), 4.08 – 3.99 (m, 2H), 3.97 (d, *J* = 5.7 Hz, 2H), 3.60 (s, 3H), 3.58 (s, 3H), 3.47 (dd, *J* = 9.4, 6.3 Hz, 1H), 3.41 – 3.25 (m, 2H), 2.24 (dd, *J* = 12.3, 9.2 Hz, 1H), 1.92 (dt, *J* = 12.0, 5.8 Hz, 1H), 1.22 (t, *J* = 7.5 Hz, 3H). ^{13}C{^{1}H} NMR (101 MHz, Acetone-*d*$_6$) δ 172.3 , 171.7 , 170.6 , 169.2 , 158.9 (dd, *J* = 248.0,

4.1 Hz), 158.3 (d, J = 236.2 Hz), 137.1 (dd, J = 15.6, 6.3 Hz), 132.0 (dd, J = 29.7, 17.6 Hz), 127.3 (d, J = 31.2 Hz), 83.3 (d, J = 1.8 Hz), 79.5 , 61.6 , 54.0 , 52.0 , 52.0 , 51.6 , 42.0 , 41.7 (d, J = 4.0 Hz), 38.9 (d, J = 1.9 Hz), 34.0 – 32.7 (m), 14.6. FT-IR cm^{-1} 3309, 2954, 2997, 1739, 1661, 1546, 1465, 1358. HRMS (ESI) Calcd. for $C_{21}H_{24}F_2N_2O_8$ $[M + Na]^+$ 493.1398, found 493.1372.

Minor regioisomer:

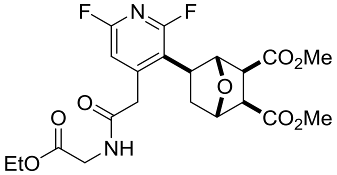

^{19}F NMR (376 MHz, Acetone-d_6) δ -67.15 (d, J = 11.8 Hz, 1F), -75.48 (d, J = 11.9 Hz, 1F). ^{1}H NMR (400 MHz, Acetone-d_6) δ 7.74 (s, 1H), 6.99 (d, J = 2.0 Hz, 1H), 4.97 (d, J = 5.0 Hz, 1H), 4.85 (s, 1H), 4.15 (q, J = 7.1 Hz, 2H), 4.04 – 3.90 (m, 4H), 3.61 (s, 3H), 3.58 (s, 3H), 3.52 – 3.44 (dd, J = 9.4, 6.3 Hz, 1H), 3.41 – 3.23 (m, 2H), 2.24 (dd, J = 12.1, 9.1 Hz, 1H), 1.90 (dt, J = 11.8, 5.8 Hz, 1H), 1.23 (t, J = 7.1 Hz, 3H).

Note: the stereochemical assignment was made on the basis of coupling constant value that matched with the literature[8] value of this bicyclic system.

Not in manuscript.

Synthesis of S-33a' dimethyl 5-(2,5,6-trifluoro-4-(2-methoxy-2-oxoethyl)pyridin-3-yl)- exo-7-oxabicyclo[2.2.1]heptane- exo, exo-2,3-dicarboxylate)

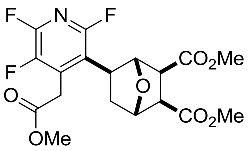

The **General procedure B** was followed using methyl 2-(3-chloro-2,5,6-trifluoropyridin-4-yl)acetate (48 mg, 0.20 mmol, 1 equiv), dimethyl-7-oxabicyclo[2.2.1]hept-5-ene exo,exo-2,3-dicarboxylate (203 mg, 0.96 mmol, 4.8 equiv), *N*,*N*-diisopropylethylamine (42 µL, 0.24 mmol, 1.2 equiv) and 2.0 mL of stock solution of $Ir(ppy)_3$ (0.3 mg, 0.0005 mmol, 0.0025 equiv) in CH_3CN was used. The crude material was purified by flash chromatography using hexane : ethyl acetate (0-30 % EtOAc for 55 cv, ramped to 100% EtOAc and then held at 100% EtOAc for 55-70 cv) on a 12 g silica column to afford **S-33'a** in 64% yield (53 mg, 0.13 mmol) as a yellow viscous liquid. ^{19}F NMR (376 MHz, Chloroform-d) δ -71.64 (dd, J = 28.1, 12.5 Hz, 1F), -90.20 (dd, J = 22.7, 12.5 Hz, 1F), -145.85 (ddt, J = 28.1, 22.5, 2.8 Hz, 1F). ^{1}H NMR (400 MHz, Chloroform-d) δ 5.08 (d, J = 5.0 Hz, 1H), 4.88 (s, 1H), 4.05 (s, 2H), 3.71 (s, 3H), 3.66 (s, 3H), 3.64 (s, 3H), 3.24 (dd, J = 9.6, 6.1 Hz, 1H), 3.20 – 3.03 (m, 2H), 2.21 – 2.05 (m, 1H), 1.91 (dt, J = 12.2, 5.7 Hz, 1H). ^{13}C{^{1}H} NMR (101 MHz, Chloroform-d) δ 171.2, 170.5, 169.2, 152.9 (ddd, J = 244.3, 11.9, 2.9 Hz), 147.2 (ddd, J = 245.1, 18.1, 16.0 Hz), 142.0 (ddd, J = 253.0, 25.2, 5.9 Hz), 138.0 (ddd, J = 13.0, 4.8, 2.5 Hz), 122.0 (dd, J = 27.3, 6.7 Hz), 82.2, 78.6, 77.5, 53.3, 52.8, 52.3 (d, J = 5.5 Hz), 51.1, 40.2, 38.4, 31.9. FT-IR cm^{-1} 2960, 2920, 2850, 1731, 1631, 1460. GC/MS (m/z, relative intensity) 417 (M^+, 5), 185 (15), 59 (100).

Synthesis of S-33a dimethyl 5-(2,5-difluoro-4-(2-methoxy-2-oxoethyl)pyridin-3-yl)-exo-7-oxabicyclo[2.2.1]heptane-exo, exo-2, 3-dicarboxylate)

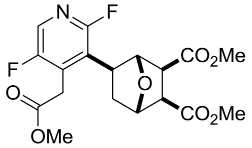

The **General procedure E** was followed using dimethyl 5-(2,5,6-trifluoro-4-(2-methoxy-2-oxoethyl)pyridine-3-yl)- **exo-**7-oxabicyclo [2.2.1]heptane-**exo, exo-**2,3-dicarboxylate) **S-33a'** (70 mg, 0.17 mmol, 1 equiv), *N,N*-diisopropylethylamine (89 µL, 0.51 mmol, 3 equiv) and 1.7 mL of stock solution of Ir(ppy)$_3$ in CH_3CN was used. The crude material was purified by flash chromatography using hexane : ethyl acetate (0-50% EtOAc for 50 cv, ramped to 100% EtOAc and then held at 100% EtOAc for 50-60 cv) on a 24 g silica column to afford **S-33a** in 71% yield (48 mg, 0.12 mmol) as a colorless liquid. ^{19}F NMR (376 MHz, Chloroform-*d*) δ -71.14 (d, *J* = 28.7 Hz, 1F), -132.70 (d, *J* = 28.7 Hz, 1F). ^{1}H NMR (400 MHz, Chloroform-*d*) δ 7.91 (s, 1H), 5.13 (d, *J* = 4.9 Hz, 1H), 4.94 (s, 1H), 4.04 (s, 2H), 3.73 (s, 3H), 3.69 (s, 3H), 3.67 (s, 3H), 3.32 (dd, *J* = 9.6, 6.3 Hz, 1H), 3.14 (m, 2H), 2.13 (dd, *J* = 12.7, 9.3 Hz, 1H), 2.01 – 1.90 (m, 1H). ^{13}C{^{1}H} NMR (101 MHz, Chloroform-*d*) δ 171.3, 170.6, 169.9, 158.3 (dd, *J* = 40.1, 2.8 Hz), 155.8 (dd, *J* = 55.1, 2.8 Hz), 134.5 (dd, *J* = 16.0, 4.9 Hz), 132.0 (dd, *J* = 29.2, 17.3 Hz), 125.3 (d, *J* = 30.1 Hz), 82.2 (d, *J* = 1.4 Hz), 78.7 (d, *J* = 3.3 Hz), 53.6, 52.8, 52.4, 52.4, 51.2, 40.5, 38.7, 31.7 (dd, *J* = 4.4, 2.5 Hz). FT-IR cm^{-1} 3008, 2954, 1737, 1468, 1430, 1409, 1347. GC/MS (m/z, relative intensity) 399 (M^{+}, 5), 113 (10), 59 (100). HRMS (ESI) Calcd. for $C_{18}H_{19}F_2NO_6$ [M + H]$^{+}$ 384.1258, found 384.0040.

Note: the stereochemical assignment was made on the basis of coupling constant value that matched with the literature[8] value of this bicyclic system.

Synthesis of S-34a (methyl 2-(exo-bicyclo[2.2.1]heptan-2-yl)-3,5-difluoro-4-(2-methoxy-2-oxoethyl)benzoate)

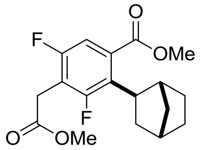

The **General procedure F** was followed using **(**methyl 2-(exo-bicyclo[2.2.1]heptan-2-yl)-3,5,6-trifluoro-4-(2-methoxy-2-oxoethyl)benzoate) (79 mg, 0.22 mmol, 1 equiv), *N,N*-diisopropylethylamine (765 µL, 4.4 mmol, 20 equiv) and 2.2 mL of stock solution of Ir(CF$_3$ppy)$_3$ (0.8 mg, 0.011 mmol, 0.005 equiv)in CH_3CN was used. The crude material was purified by flash chromatography using hexane : ethyl acetate (0-5% EtOAc for 25 cv, ramped to 100% EtOAc and then held at 100% EtOAc for 25-35 cv) on a 4 g silica column to afford **S-34a** in 73% yield (54 mg, 0.06 mmol) as a colorless liquid. ^{19}F NMR (376 MHz, Chloroform-*d*) δ -109.10 (s, 1F), -117.76 (t, *J* = 8.0 Hz, 1F). ^{1}H NMR (400 MHz, Chloroform-*d*) δ 7.18 (dd, *J* = 9.0, 1.3 Hz, 1H), 3.90 (s, 3H), 3.72 (s, 3H), 3.70 (s, 2H), 3.14 (t, *J* = 8.0 Hz, 1H), 2.44 (s, 1H), 2.33 (s, 1H), 1.79 (dd, *J* = 26.4, 9.0 Hz, 3H), 1.56 (m, 1H), 1.37 – 1.19 (m, 3H). ^{13}C{^{1}H} NMR (101 MHz, Chloroform-*d*) δ 169.9, 167.8 – 167.5 (m), 160.4 (dd, *J* = 114.3, 7.9 Hz), 157.9 (dd, *J* = 111.4, 7.9 Hz), 133.5 (t, *J* = 8.7 Hz), 129.4 (dd, *J* = 15.7, 4.1 Hz), 114.2 (dd, *J* = 23.4, 19.1 Hz), 112.2 (dd, *J* = 23.7, 3.6 Hz), 77.4, 52.7, 52.5, 42.9 (t, *J* = 3.2 Hz), 39.5 (d, *J* = 4.1 Hz), 37.7, 37.6, 37.0, 32.0, 28.4 – 28.2 (m). FT-IR cm^{-1} 2957, 2874, 1740, 1441, 1323. GC/MS (m/z, relative intensity) 338 (M^{+}, 10), 306 (30), 270(25), 59 (100). HRMS (ESI) Calcd. for $C_{18}H_{20}F_2O_3$ [M + H]$^{+}$ 323.1459, found 323.1821.

Synthesis of S-35a N-(3-(exo-bicyclo[2.2.1]heptan-2-yl)-2,6-difluoro-4-(trifluoromethyl)phenyl)acetamide

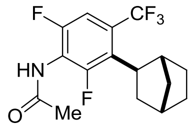

The **General procedure D** was followed using ethyl 2-(2-(2,3,5,6-tetrafluoro-4-(trifluoromethyl)phenyl)acetamido)acetate (56 mg, 0.20 mmol, 1 equiv), 2-norbornene (112 mg, 1.2 mmol, 6 equiv), *N*,*N*-diisopropylethylamine (42 µL, 0.24 mmol, 1.2 equiv, for second step 104 µL, 0.6 mmol, 3 equiv) and 2.0 mL of stock solution of Ir(ppy)$_3$ in CH_3CN was used. The crude material was purified by flash chromatography using hexane : ethyl acetate (0-11% EtOAc for 57 cv, ramped to 100% EtOAc and then held at 100% EtOAc for 57-65 cv) on a 24 g silica column to afford **S-35a** in 56% yield (37 mg, 0.11 mmol) as a colorless liquid. ^{19}F NMR (376 MHz, Chloroform-*d*) δ -58.6 (s, 3F), -111.6 (s, 1F), -117.8(s, 1F). ^{1}H NMR (400 MHz, Chloroform-*d*) δ 7.29 (s, 1H), 6.79 (s, 1H), 2.97 (t, *J* = 8.1 Hz, 1H), 2.38 (s, 1H), 2.30 (s, 1H), 2.23 (s, 3H), 1.94 – 1.83 (m, 1H), 1.75 (q, *J* = 11.0 Hz, 1H), 1.56 (ddt, *J* = 27.6, 11.4, 5.7 Hz, 2H), 1.39 – 1.21 (m, 4H). ^{13}C{^{1}H} NMR (151 MHz, Chloroform-*d*) δ 168.0, 156.6 (dd, *J* = 250.9, 4.4 Hz), 154.8 (dd, *J* = 250.8, 4.9 Hz), 129.4 – 128.3 (m), 123.4 (q, *J* = 274.8, 274.1 Hz), 117.9 (dd, *J* = 23.2, 10.3 Hz), 110.5 – 110.1 (m), 43.5, 41.6, 39.3, 37.4, 37.4, 36.9, 32.2, 27.7. FT-IR cm^{-1} 3257, 3196, 3014, 2957, 2872, 1687, 1532, 1444, 1355. GC/MS (m/z, relative intensity) 333 (M^{+}, 5), 223 (25), 43 (100). HRMS (ESI) Calcd. for $C_{16}H_{16}F_5NO$ [M + Na]$^{+}$ 356.1050, found 356.1035.

Not in manuscript.

Synthesis of S-36a’ ethyl (2-(exo-3-(bicyclo[2.2.1]heptan-2-yl)-2,5,6-trifluoro-4-(trifluoromethyl)phenyl)acetyl)glycinate

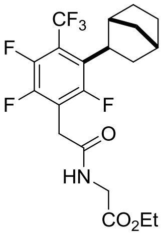

The **General procedure B** was followed using ethyl 2-(2-(2,3,5,6-tetrafluoro-4-(trifluoromethyl)phenyl)acetamido)acetate (144 mg, 0.40 mmol, 1 equiv), 2-norbornene (231 mg, 2.4 mmol, 6 equiv), *N*,*N*-diisopropylethylamine (84 µL, 0.48 mmol, 1.2 equiv) and 4.0 mL of stock solution of Ir(ppy)$_3$ (1.3 mg, 0.002 mmol, 0.005 equiv) in CH_3CN was used. The crude material was purified by flash chromatography using hexane : ethyl acetate (0-45% EtOAc for 50 cv, ramped to 100% EtOAc and then held at 100% EtOAc for 50-60 cv) on a 40 g silica column to afford **S-36a’** in 53% yield (93 mg, 0.21 mmol) as a yellow viscous liquid. ^{19}F NMR (376 MHz, Chloroform-*d*) δ -53.08 (d, *J* = 37.6 Hz, 3F), -111.52 (d, *J* = 14.1 Hz, 1F), -137.63 (dd, *J* = 21.0, 3.7 Hz, 1F), -140.84 – -141.31 (m, 1F). ^{1}H NMR (400 MHz, Chloroform-*d*) δ 6.28 – 6.16 (m, 1H), 4.22 (q, *J* = 7.2 Hz, 2H), 4.05 (d, *J* = 5.0 Hz, 2H), 3.69 (s, 2H), 2.95 (t, *J* = 8.1 Hz, 1H), 2.39 – 2.30 (m, 2H), 1.87 (dddt, *J* = 22.8, 11.1, 6.7, 2.6 Hz, 2H), 1.71 (dd, *J* = 21.3, 9.2 Hz, 1H), 1.65 – 1.46 (m, 2H), 1.40 – 1.17 (m, 6H). ^{13}C{^{1}H} NMR (101 MHz, Chloroform-*d*) δ 169.8 , 167.4 , 156.8 – 153.2 (m), 147.7 (ddd, *J* = 249.0, 15.7, 9.1 Hz), 147.03 – 143.98 (m), 128.8 (d, *J* = 17.5 Hz), 122.8 (dddd, *J* = 279.7, 276.9, 273.0, 3.3 Hz), 117.8 (ddd, *J* = 30.8, 8.2, 5.8 Hz), 117.2 (dd, *J* = 25.4, 15.8 Hz),

61.9, 43.4 (d, J = 2.5 Hz), 41.9 (d, J = 2.8 Hz), 39.6 (d, J = 4.4 Hz), 37.7 (d, J = 12.8 Hz), 37.0, 32.4, 30.4 – 30.2 (m), 27.8 (d, J = 2.9 Hz), 14.2. FT-IR cm^{-1} 3327, 3305, 2957, 2877, 1749, 1666, 1553, 1492, 1371. GC/MS (m/z, relative intensity) 437 (M^+, 5), 370 (50), 102 (80), 67 (100).
Note: isolated product contains three minor products. The first is a minor regioisomer, ethyl (2-(2-(bicyclo[2.2.1]heptan-2-yl)-3,5,6-trifluoro-4-(trifluoromethyl)phenyl)acetyl)glycinate, 5%, which underwent alkylation meta to the trifluoromethyl group. The second and third compounds, 3% and 4%, are ethyl (2-(2-(bicyclo[2.2.1]heptan-2-yl)-3,6-difluoro-4-(trifluoromethyl)phenyl)acetyl)glycinate and ethyl (2-(3-(bicyclo[2.2.1]heptan-2-yl)-2,6-difluoro-4-(trifluoromethyl)phenyl)acetyl)glycinate, which are the product of subsequent hydrodefluorination of minor regioisomer and major regioisomer of **S-36a'** respectively by ^{19}F NMR.

Synthesis of 36a ethyl (2-(3-(exo-bicyclo[2.2.1]heptan-2-yl)-2,6-difluoro-4-(trifluoromethyl)phenyl)acetyl)glycinate

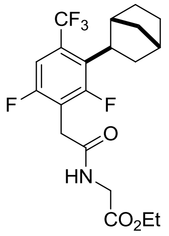

The **General procedure D** was followed using **ethyl (2-(3-(bicyclo[2.2.1]heptan-2-yl)-2,5,6-trifluoro-4-(trifluoromethyl)phenyl)acetyl)glycinate** (30 mg, 0.07 mmol, 1 equiv), *N,N*-diisopropylethylamine (37 µL, 0.21 mmol, 3 equiv) and 0.7 mL of stock solution of Ir(ppy)$_3$ in CH_3CN was used. The crude material was purified by flash chromatography using hexane : ethyl acetate (0-19% EtOAc for 25 cv, ramped to 100% EtOAc and then held at 100% EtOAc for 25-35 cv) on a 4 g silica column to afford **S-36a** in 79% yield (23 mg, 0.05 mmol) as a colorless liquid. ^{19}F NMR (376 MHz, Chloroform-*d*) δ -58.79 (s, 3H), -105.73 (d, J = 6.9 Hz, 1F), -115.73 (t, J = 8.2 Hz, 1F). ^{1}H NMR (400 MHz, Chloroform-*d*) δ 7.23 (dd, J = 9.4, 1.7 Hz, 1H), 6.08 (s, 1H), 4.22 (q, J = 7.2 Hz, 2H), 4.05 (d, J = 5.0 Hz, 2H), 3.68 (s, 2H), 2.96 (t, J = 8.5 Hz, 1H), 2.38 (t, J = 4.2 Hz, 1H), 2.30 (d, J = 3.2 Hz, 1H), 1.98 – 1.82 (m, 2H), 1.72 (dd, J = 11.9, 9.3 Hz, 1H), 1.64 – 1.48 (m, 3H), 1.39 – 1.19 (m, 6H). ^{13}C{^{1}H} NMR (151 MHz, Chloroform-*d*) δ 169.8, 168.0, 160.5 (dd, J = 250.9, 6.8 Hz), 158.8 (dd, J = 247.3, 8.9 Hz), 130.6 (dt, J = 30.5, 9.8 Hz), 128.9 – 128.6 (m), 123.4 (q, J = 273.1 Hz), 116.1 – 115.0 (m), 109.7, 61.8, 43.4, 41.8, 41.4, 39.3 (d, J = 4.0 Hz), 37.6 (d, J = 12.6 Hz), 37.0, 32.4, 27.9 (d, J = 2.7 Hz), 14.3. FT-IR cm^{-1} 3312, 2954, 2874, 1750, 1667, 1554, 1487, 1347. GC/MS (m/z, relative intensity) 419 (M^+, 5), 352 (50), 104 (100), 67 (95). HRMS (ESI) Calcd. for $C_{20}H_{22}F_5NO_3$ $[M + H]^+$ 420.1598, found 420.1581.
Note: isolated product contains inseparable starting material (4%) and regioisomer 9% by ^{19}F NMR.

References

1. *J. Organomet. Chem.* **2015**, *776*, 51.
2. *J. Am. Chem. Soc.* **2014**, *136*, 3002.
3. *J.Org.Chem*. **2014**, *79*, 10466.
4. *Green Chem*. **2013**, *15*, 1318.
5. *J. Chem. Crystallogr*. **2007**, *37*, 543.

6. *J. Organomet. Chem.* , **1989**, *368*, 249.
7. *ARKIVOC* **2003** (ii) 15.
8.(a) NMR Spectroscopy of the Non-Metallic Elements. Berger, S ; Braun, S. ; Kalinowski, H. O. (b) *J. Chem. Faraday Trans*. 2, **1972**, *68*, 241.(c) *J. Magn. Reson*. **1976**, *22*, 207. (d) *J. Chem. Soc*. B, **1969**, 434.

^{19}F NMR (376 MHz, Chloroform-*d*, @ rt) of S-1a 4-cyclohexyl-2,3,5,6-tetrafluoropyridine (376 MHz, Chloroform-*d*, @ rt)

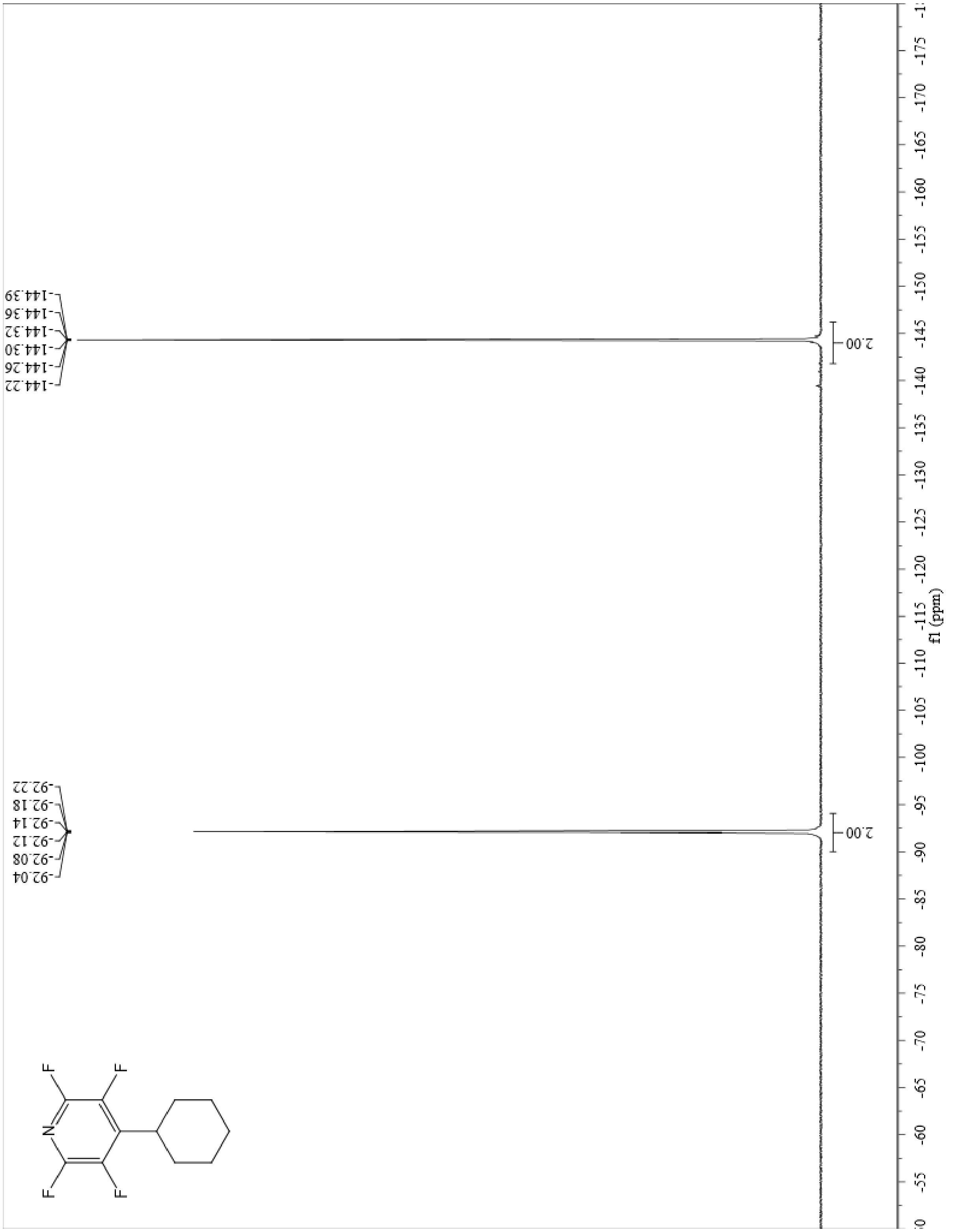

^{1}H NMR (400 MHz, Chloroform-*d*, @ rt) of S-1a 4-cyclohexyl-2,3,5,6-tetrafluoropyridine

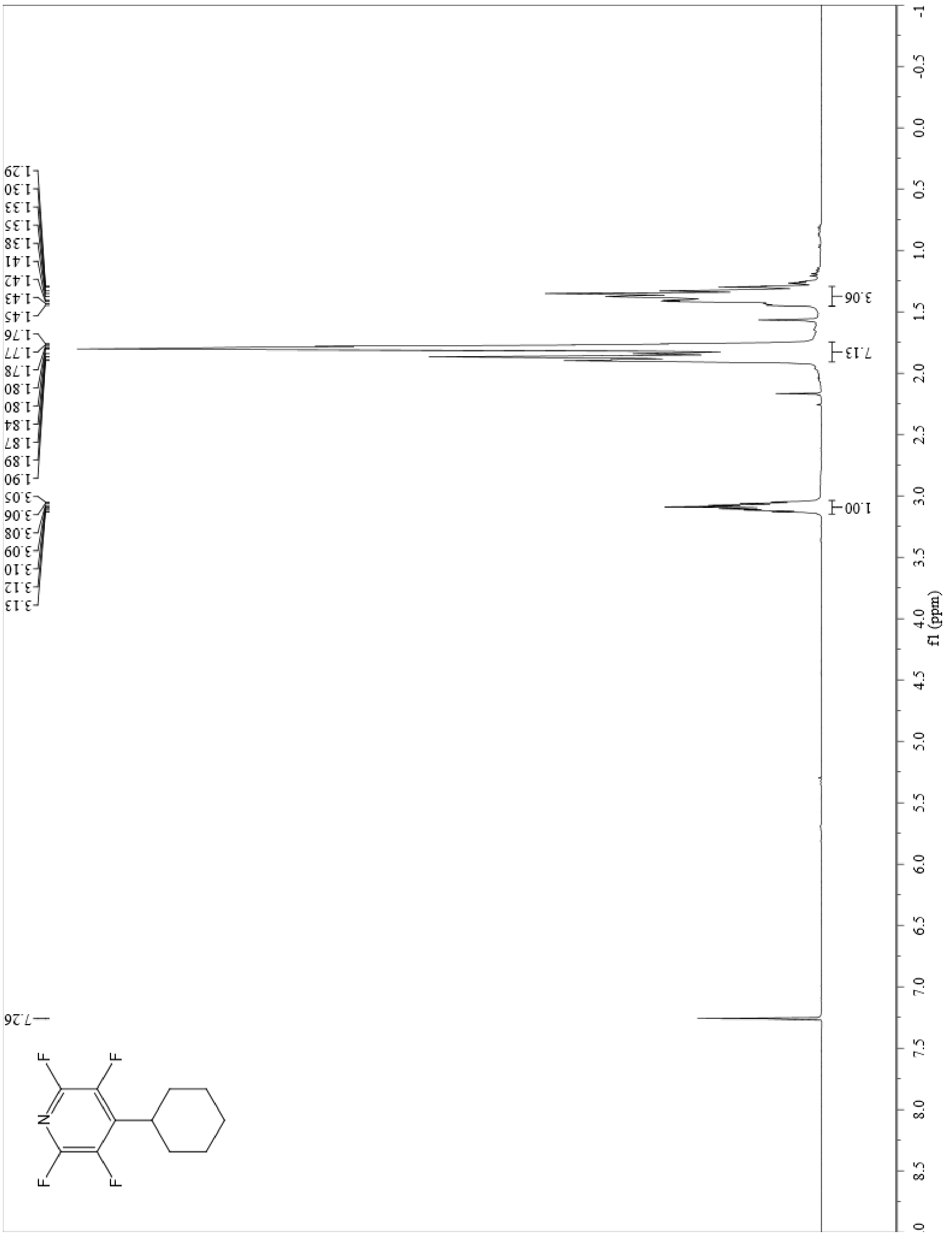

$^{13}C\{^{1}H\}$ NMR (101 MHz, Chloroform-*d*, @ rt) of 1a 4-cyclohexyl-2,3,5,6-tetrafluoropyridine

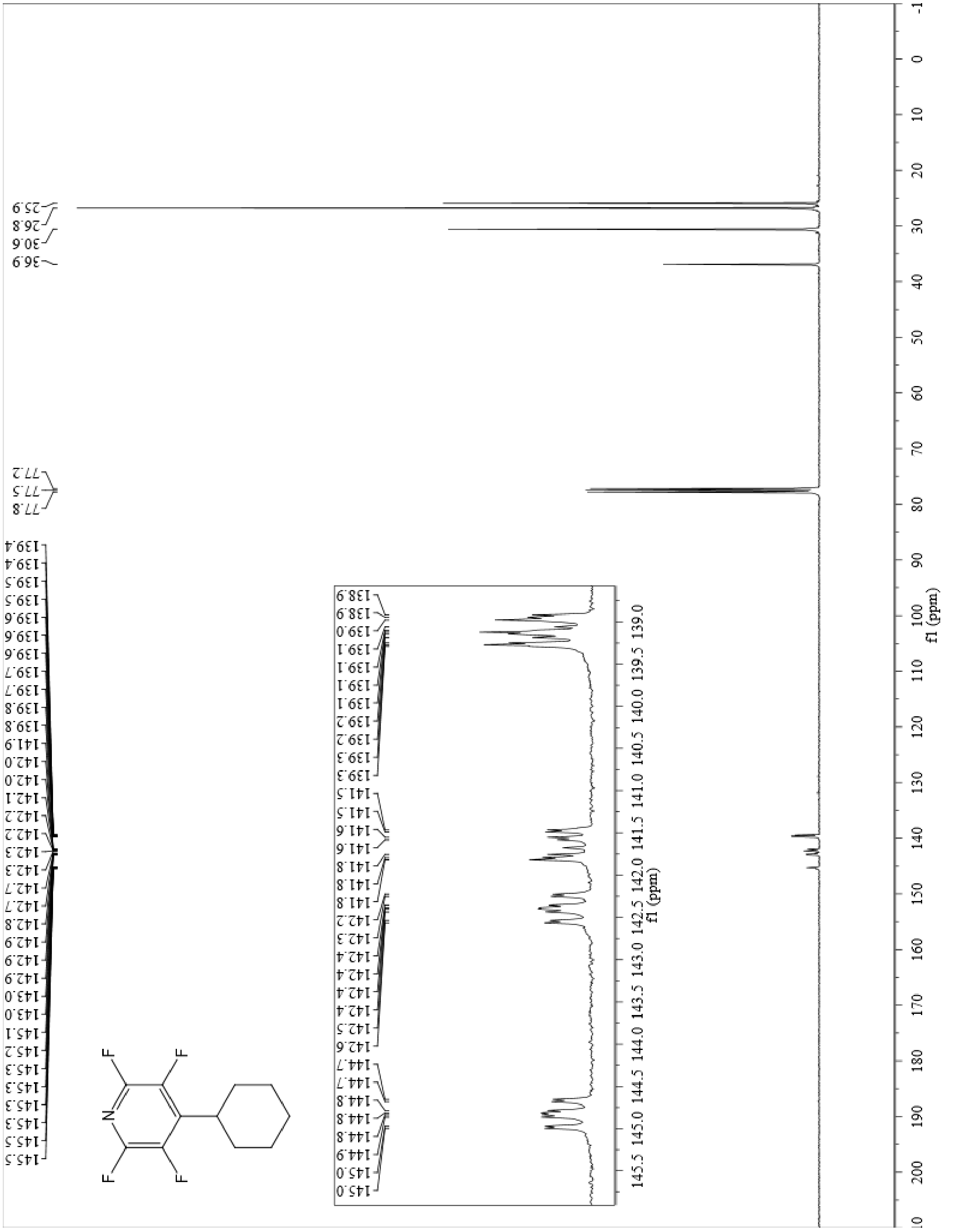

GC and MS of 1a 4-cyclohexyl-2,3,5,6-tetrafluoropyridine

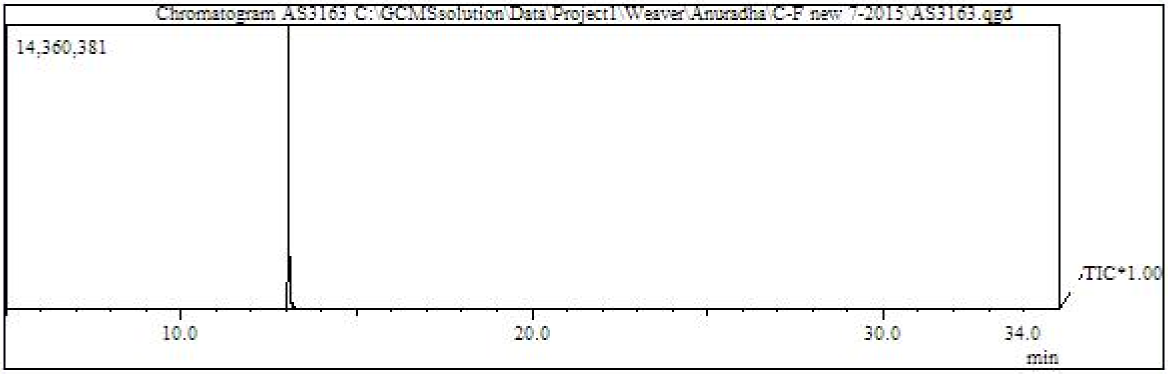

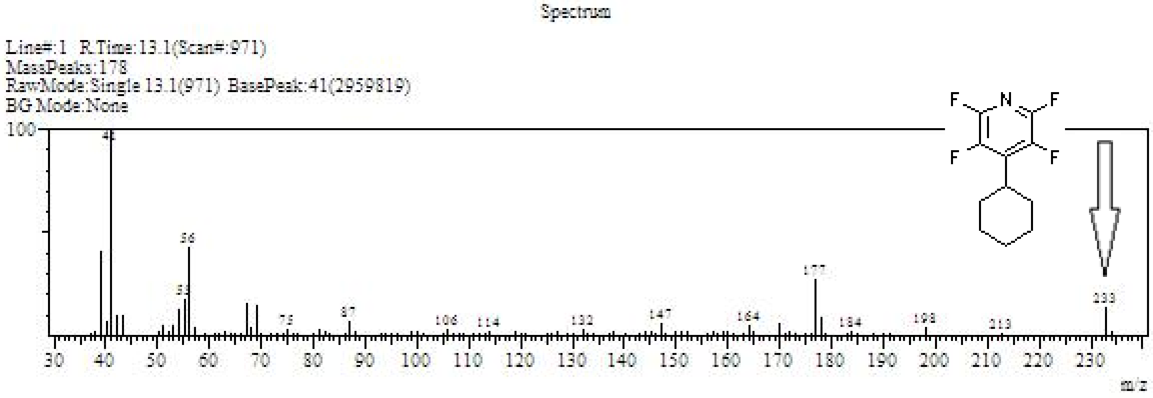

^{19}F NMR (376 MHz, Chloroform-*d*, @ rt) of S-2a 4-cyclohexyl-2,3,5,6-tetrafluorobenzonitrile

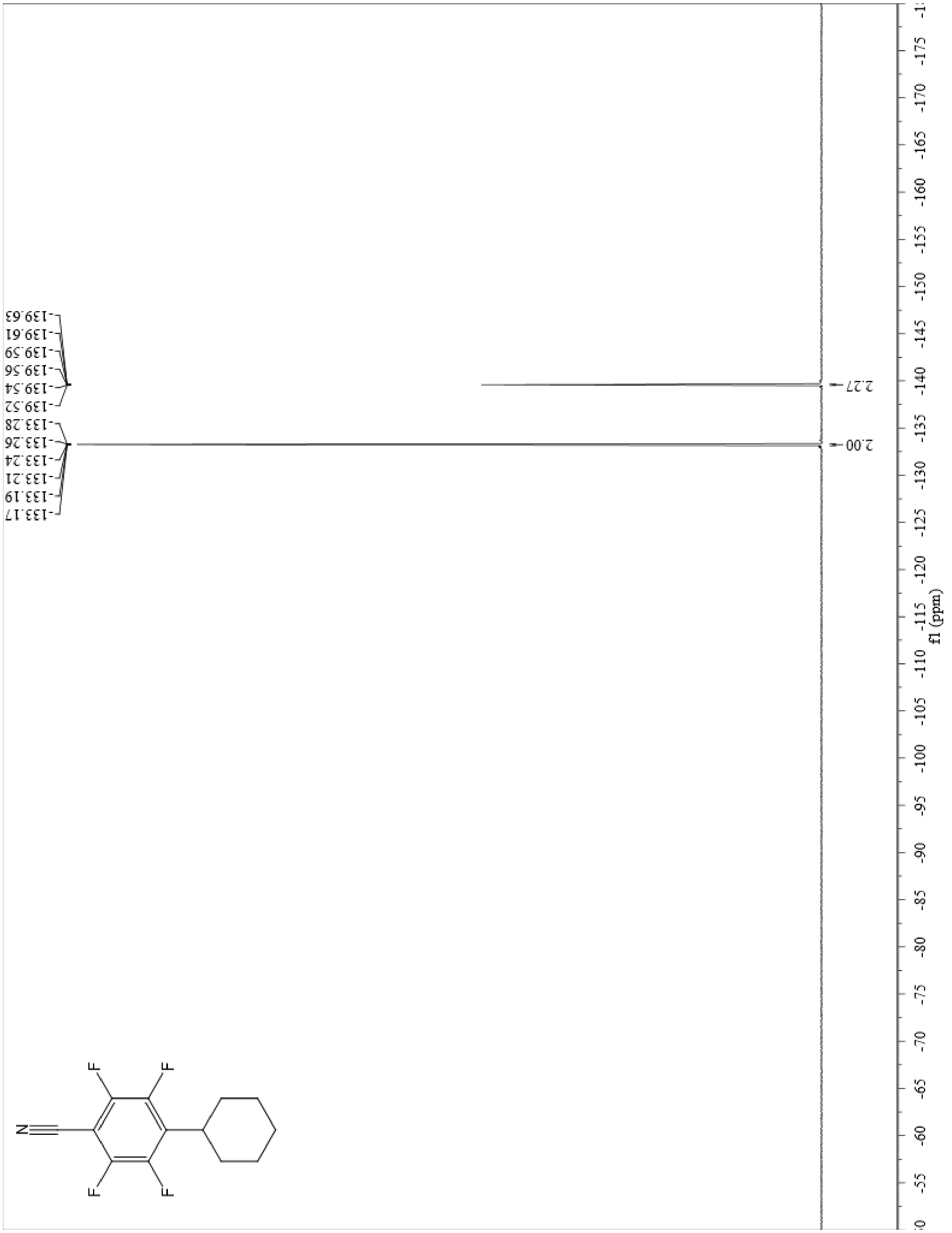

^{1}H NMR (400 MHz, Chloroform-*d*, @ rt) of S-2a 4-cyclohexyl-2,3,5,6-tetrafluorobenzonitrile

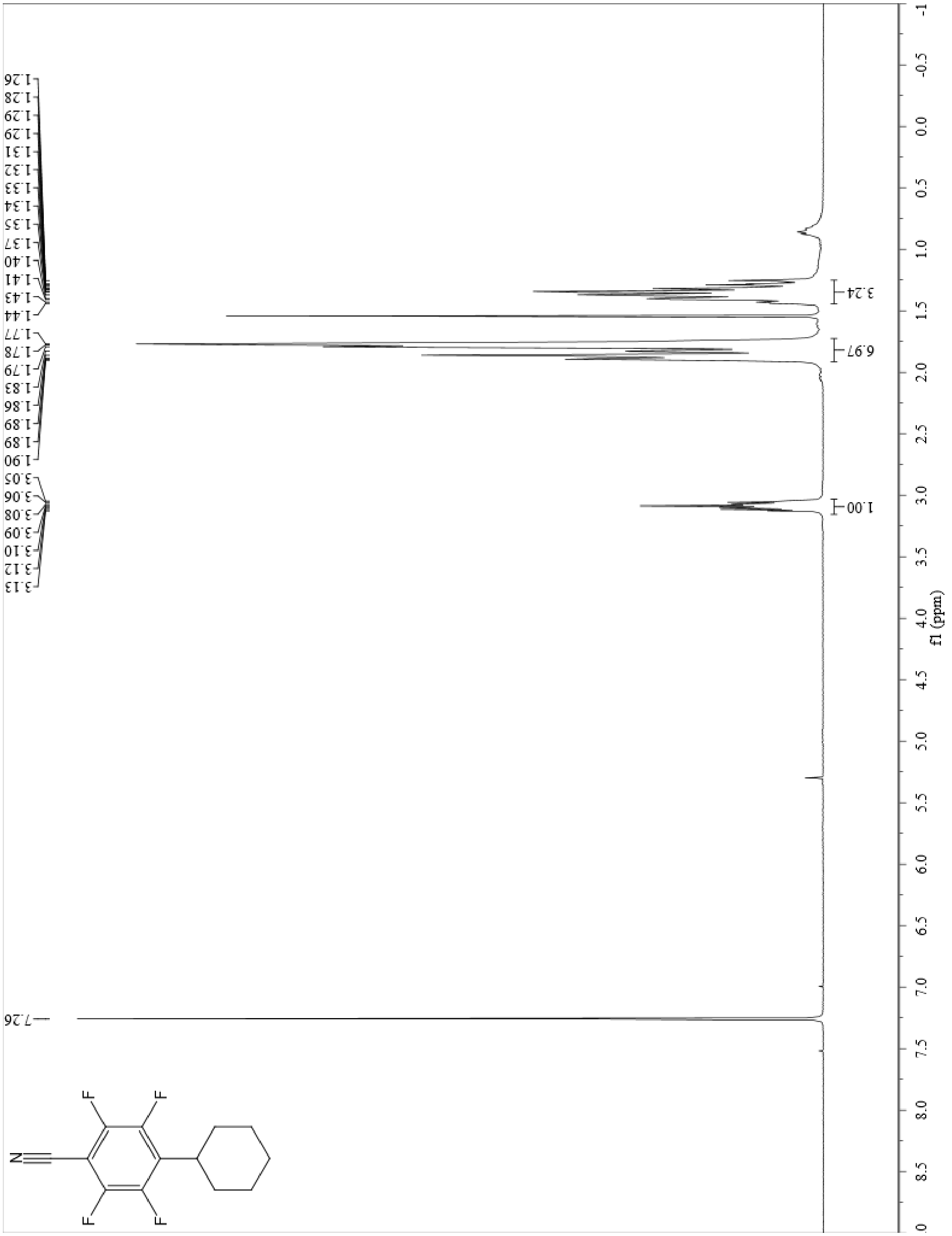

$^{13}C\{^{1}H\}$ NMR (101 MHz, Chloroform-*d*, @ rt) of S-2a 4-cyclohexyl-2,3,5,6-tetrafluorobenzonitrile

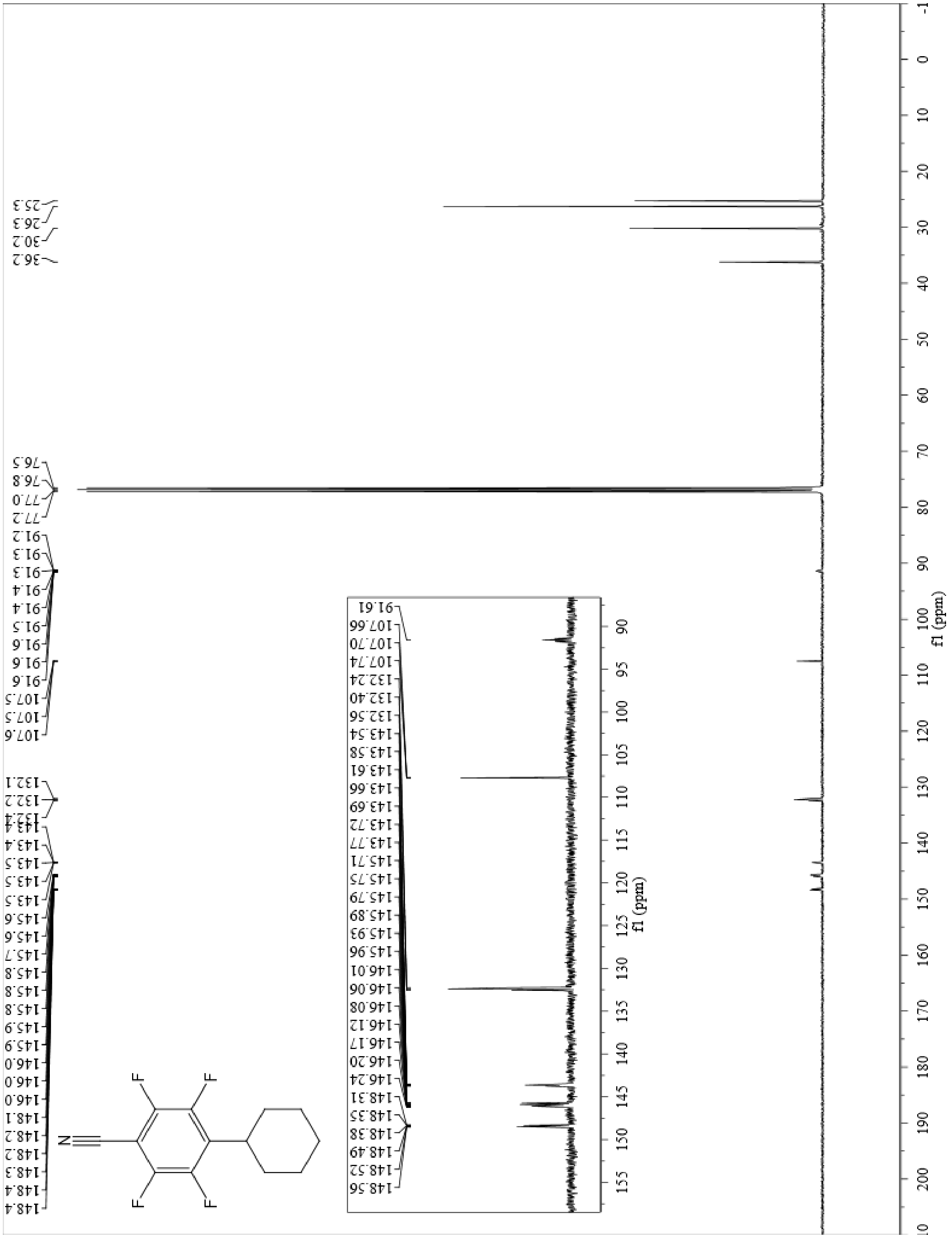

GC and MS of S-2a 4-cyclohexyl-2,3,5,6-tetrafluorobenzonitrile

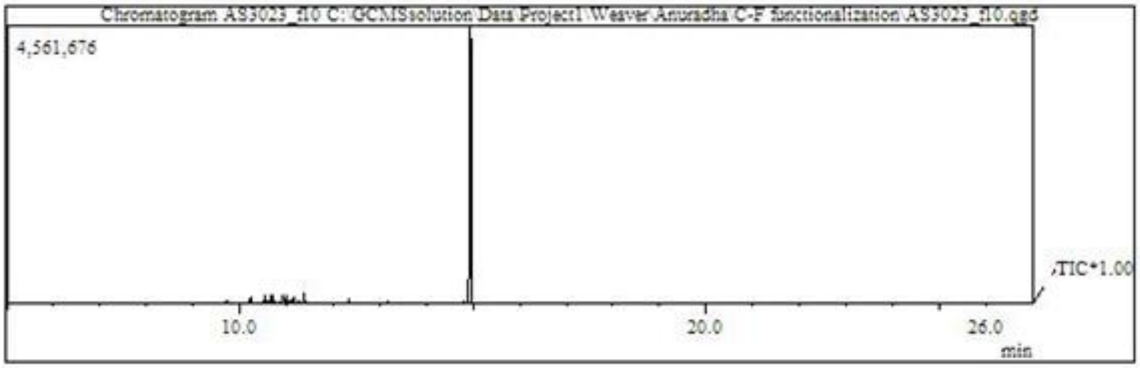

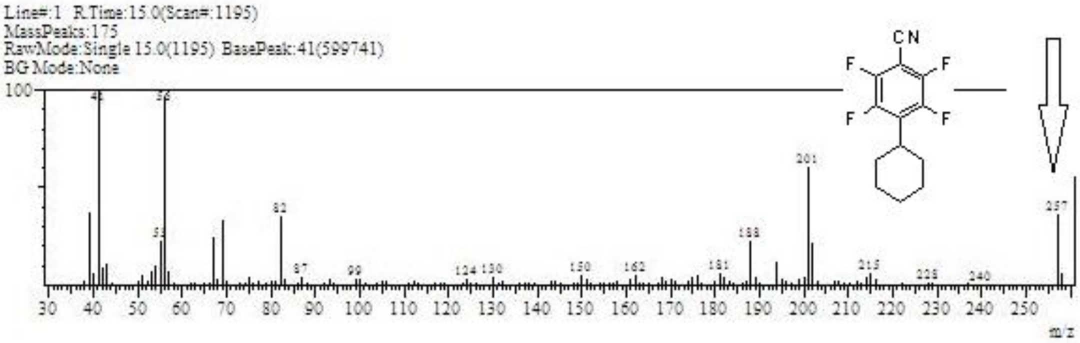

^{19}F NMR (376 MHz, Chloroform-*d*, @ rt) of S-3a S-(4-cyclohexyl-2,3,5,6-tetrafluorophenyl) O-ethyl carbonothioate

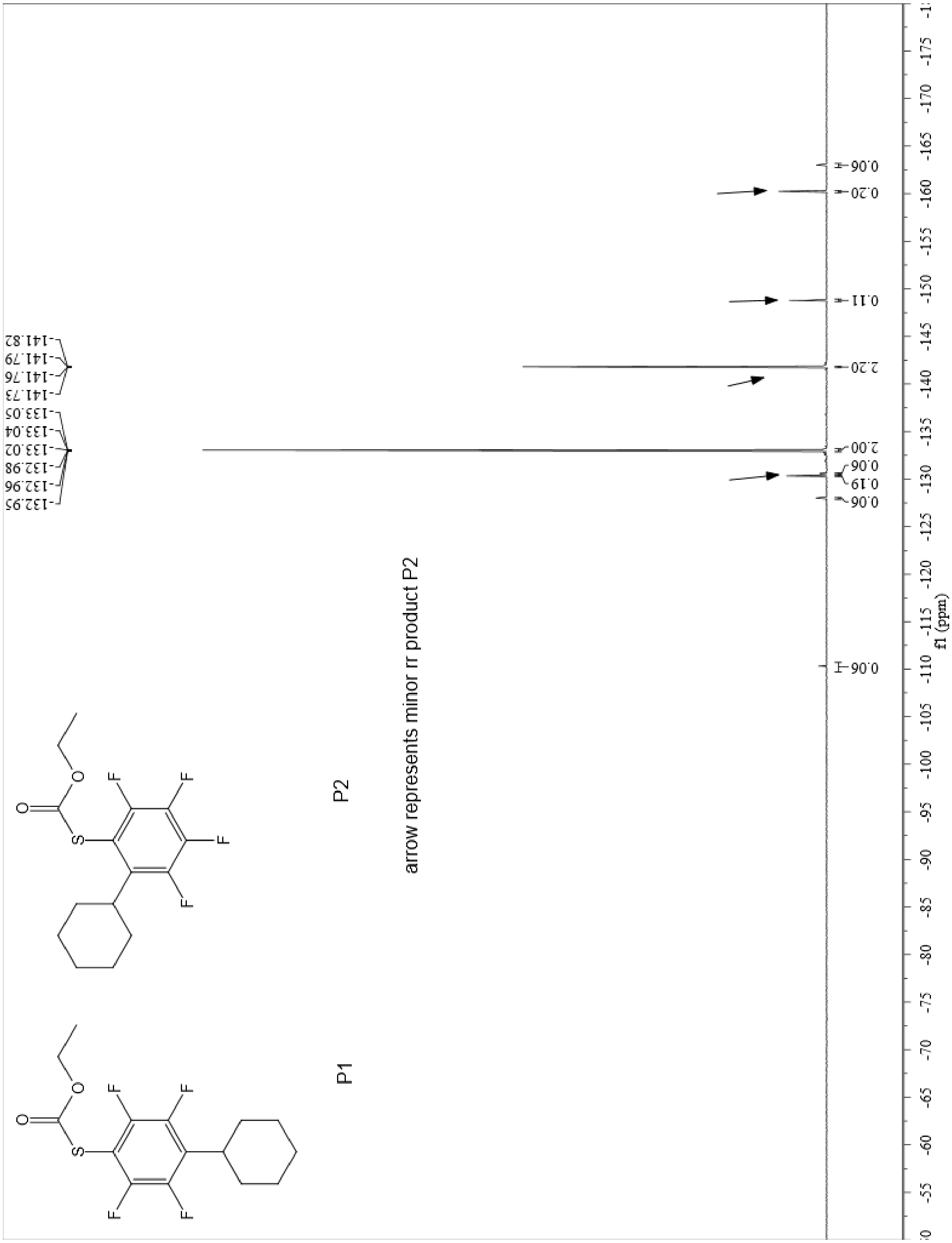

^{1}H NMR (400 MHz, Chloroform-*d*, @ rt) of S-3a S-(4-cyclohexyl-2,3,5,6-tetrafluorophenyl) O-ethyl carbonothioate

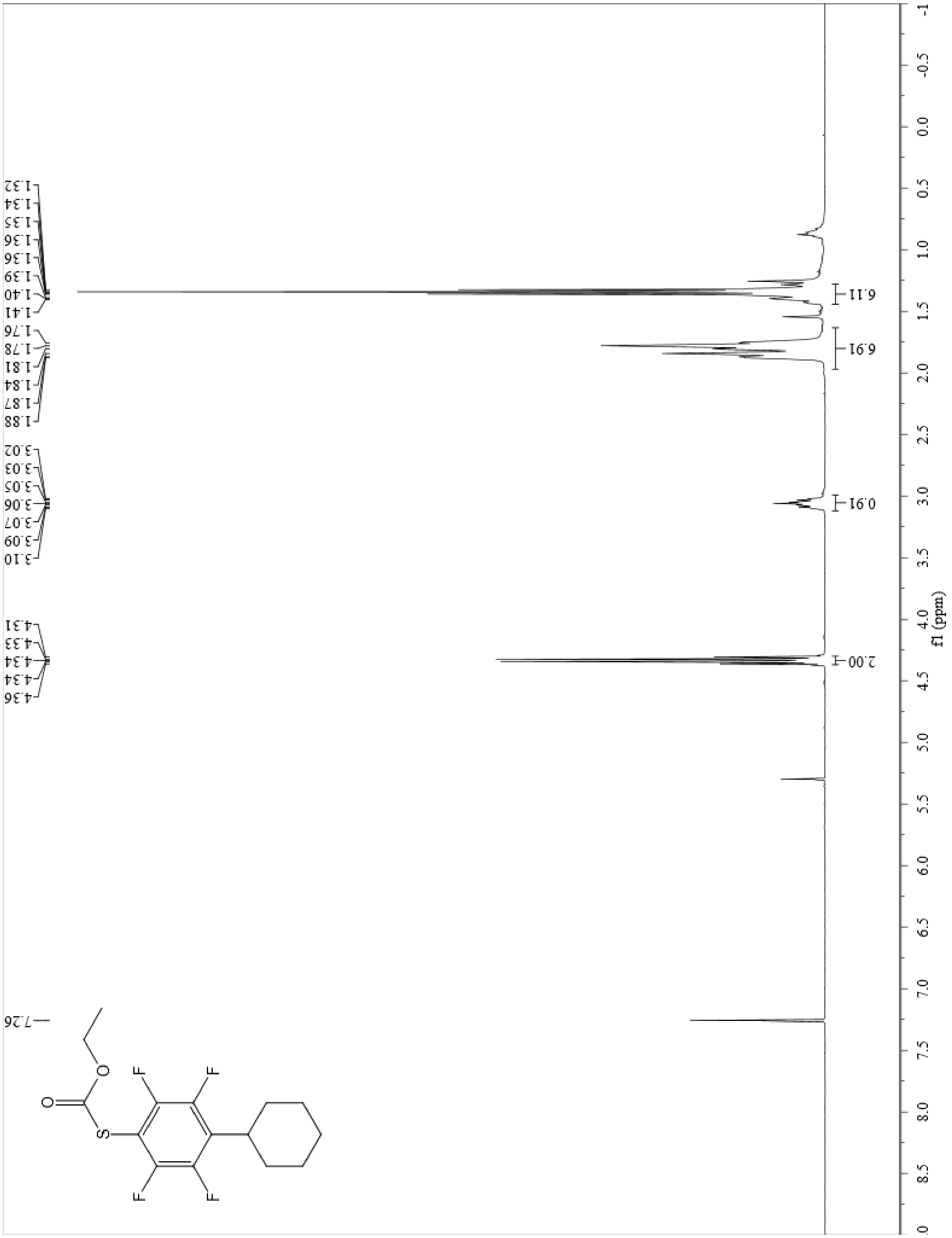

$^{13}C\{^1H\}$ NMR (101 MHz, Chloroform-*d*, @ rt) of S-3a S-(4-cyclohexyl-2,3,5,6-tetrafluorophenyl) O-ethyl carbonothioate

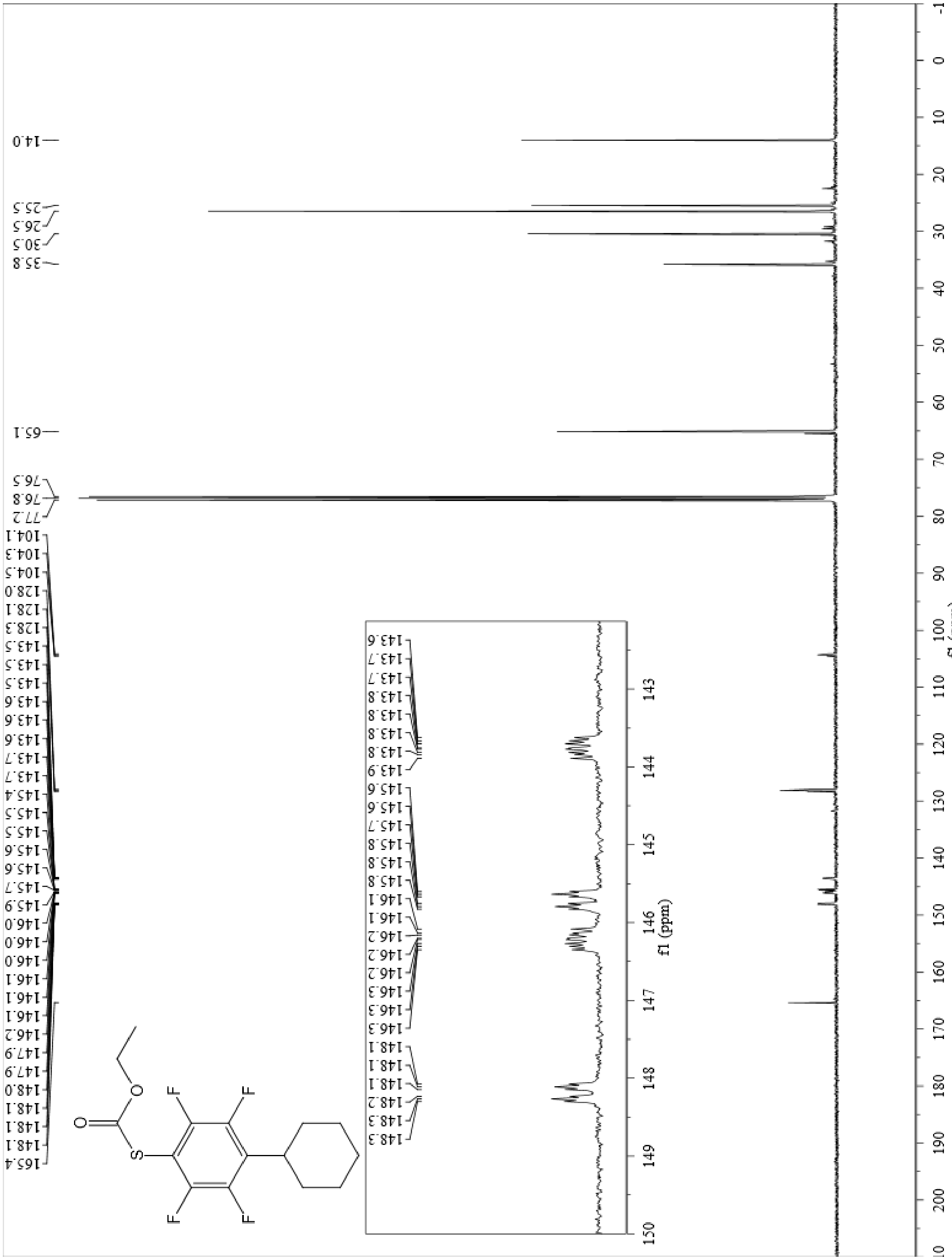

GC and MS of S-3a S-(4-cyclohexyl-2,3,5,6-tetrafluorophenyl) O-ethyl carbonothioate

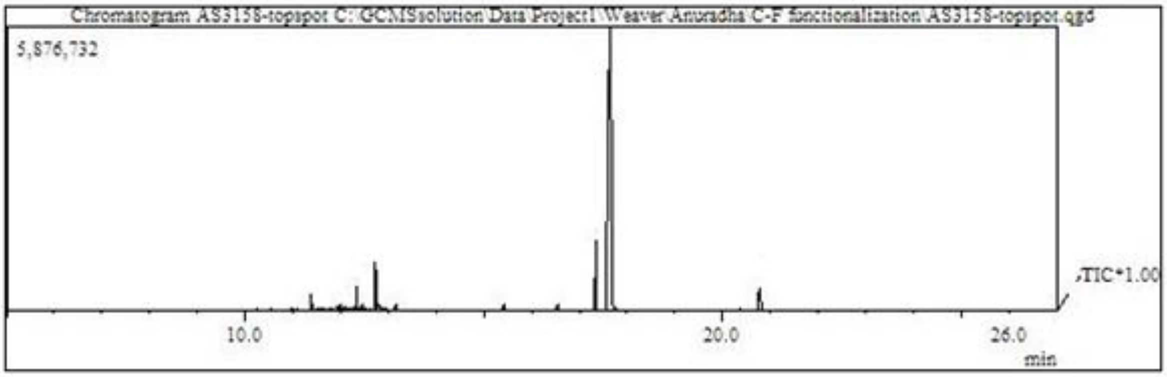

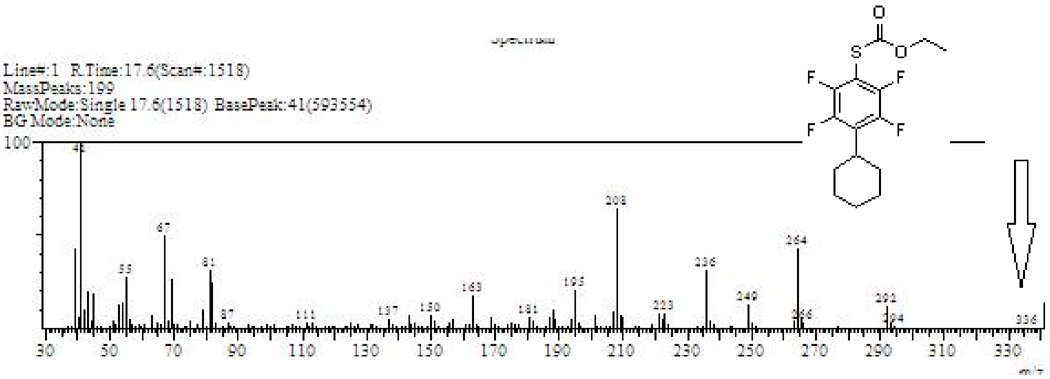

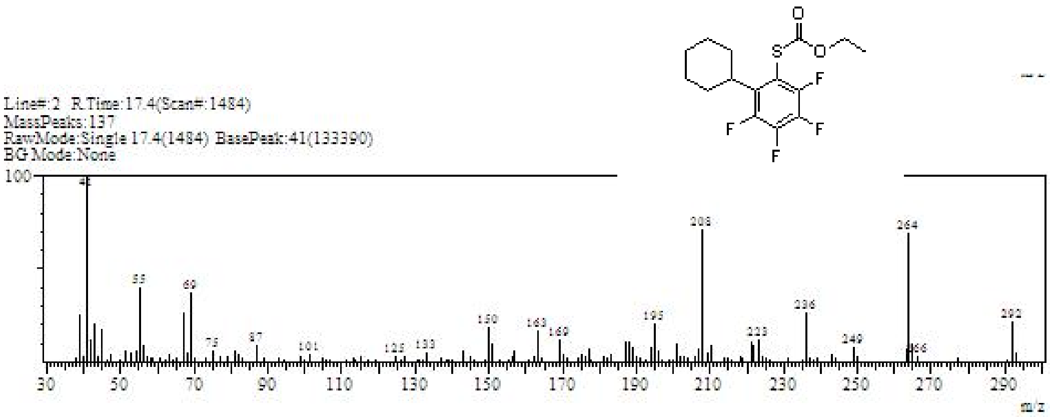

^{19}F NMR (376 MHz, Chloroform-*d*, @ rt) of S-4a methyl 4-cyclohexyl-2,3,5,6-tetrafluorobenzoate

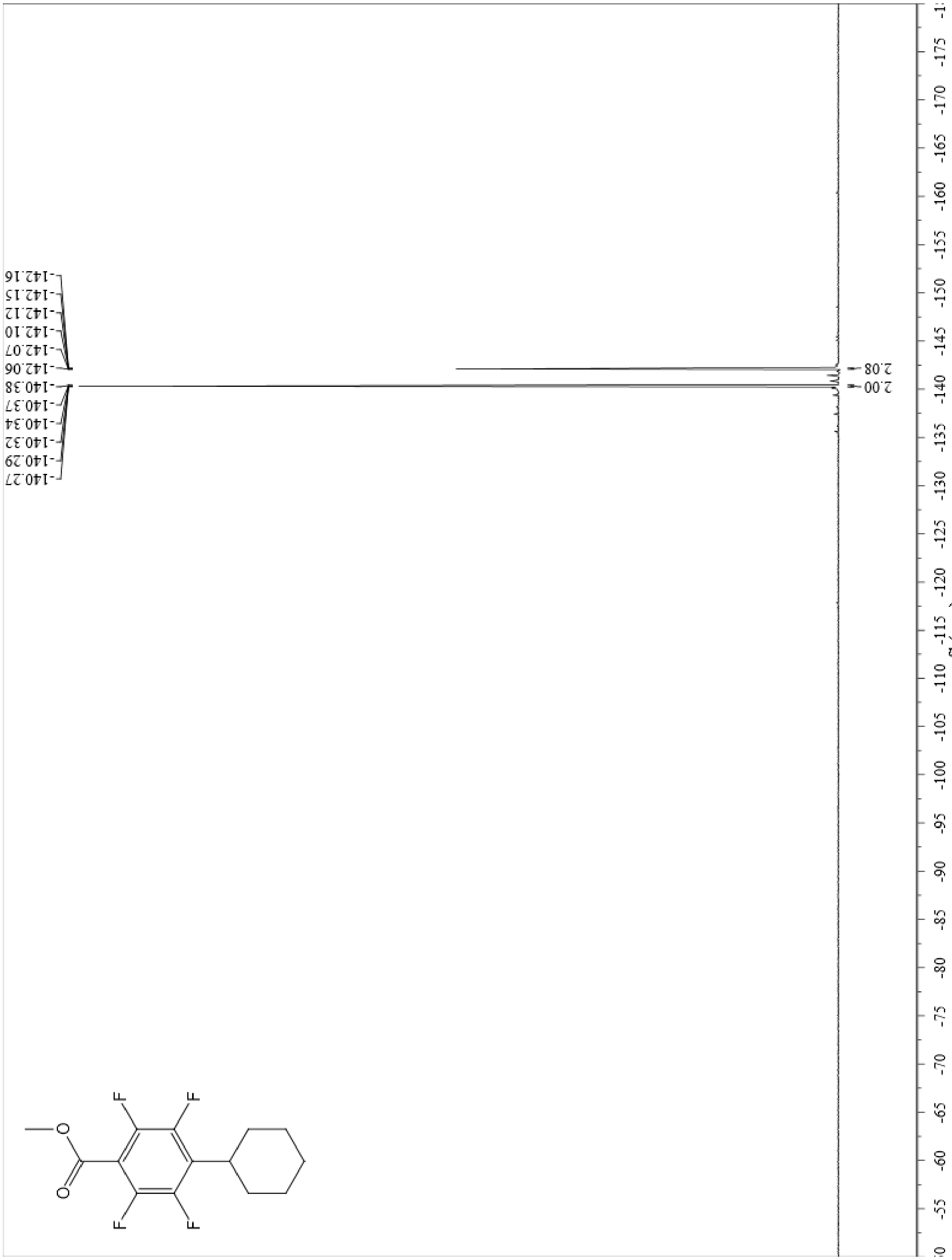

^{1}H NMR (400 MHz, Chloroform-*d*, @ rt) of S-4a methyl 4-cyclohexyl-2,3,5,6-tetrafluorobenzoate

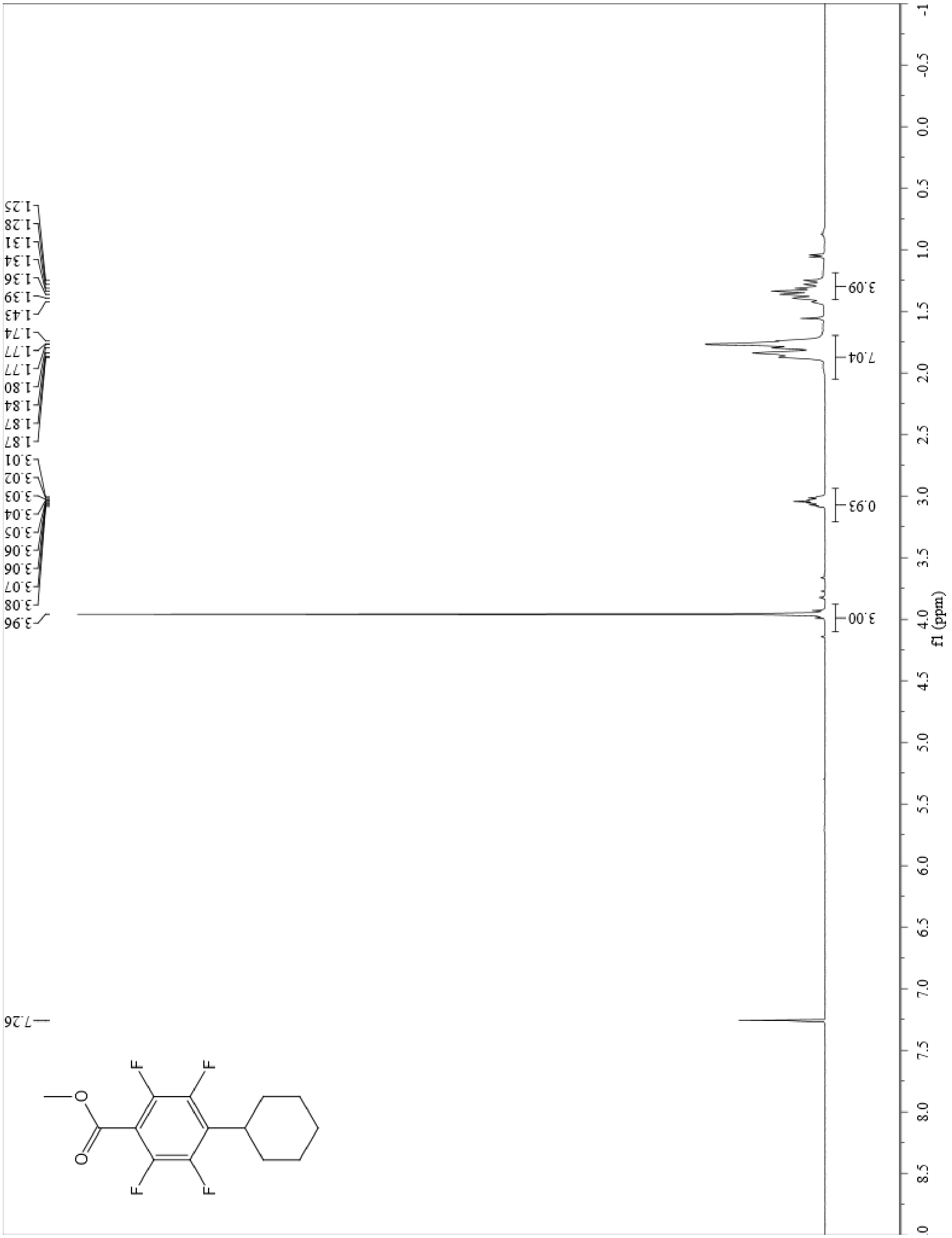

$^{13}C\{^{1}H\}$ NMR (101 MHz, Chloroform-*d*, @ rt) of S-4a methyl 4-cyclohexyl-2,3,5,6-tetrafluorobenzoate

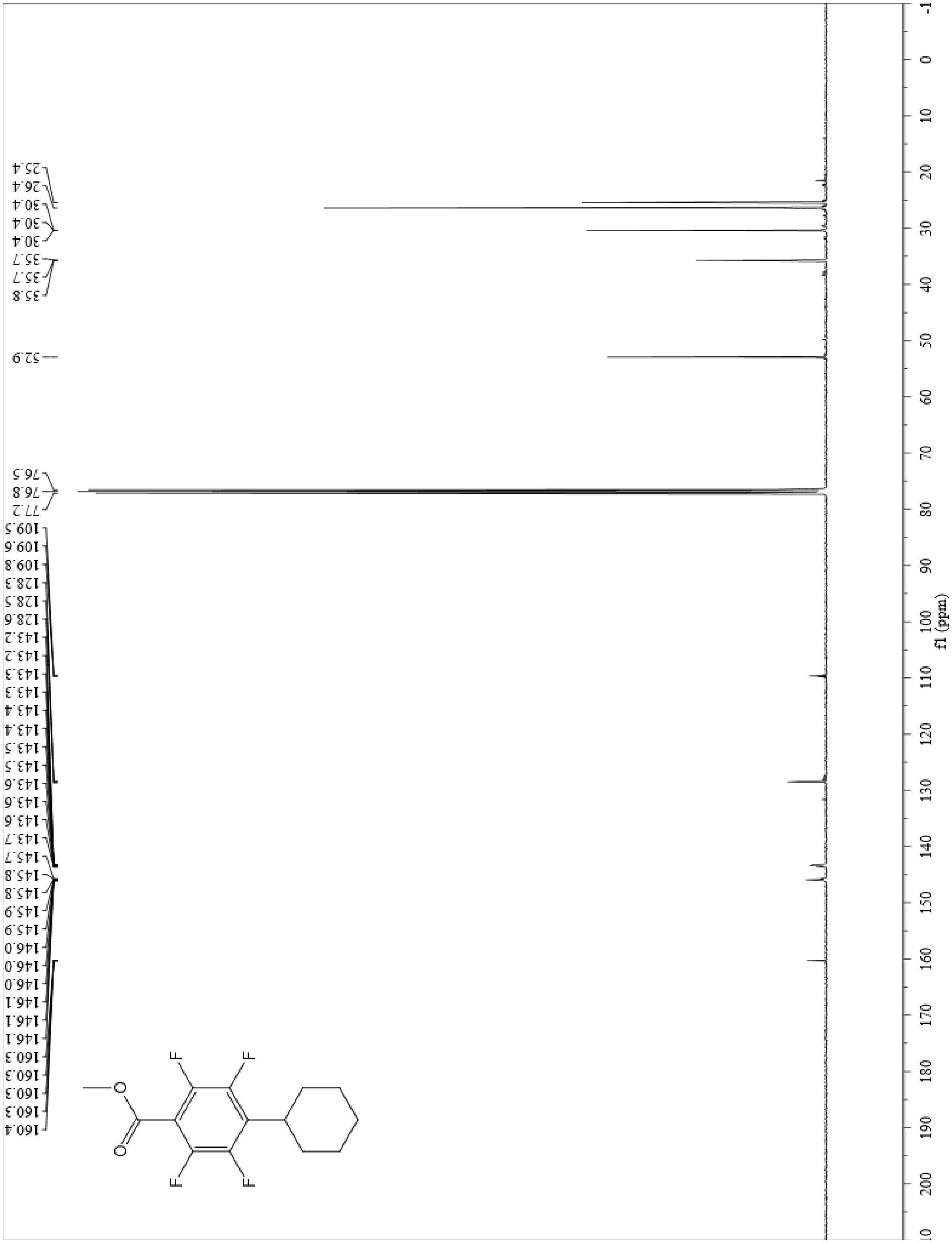

GC and MS of S-4a methyl 4-cyclohexyl-2,3,5,6-tetrafluorobenzoate

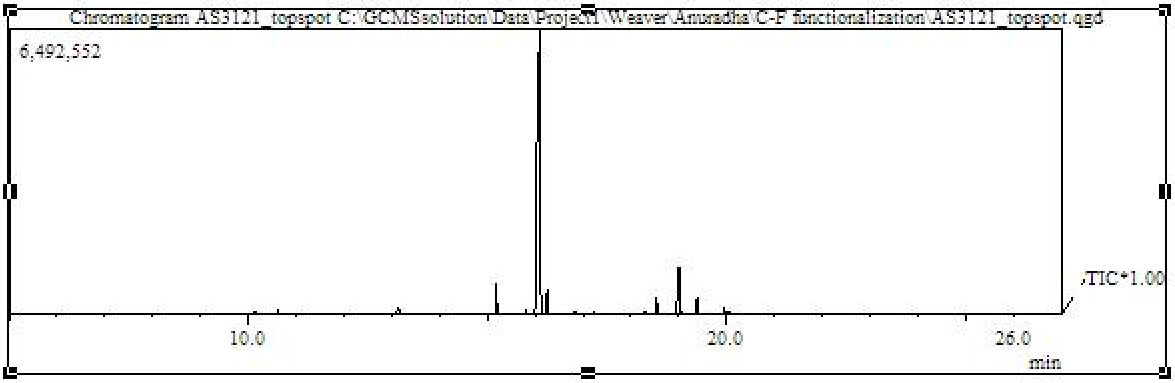

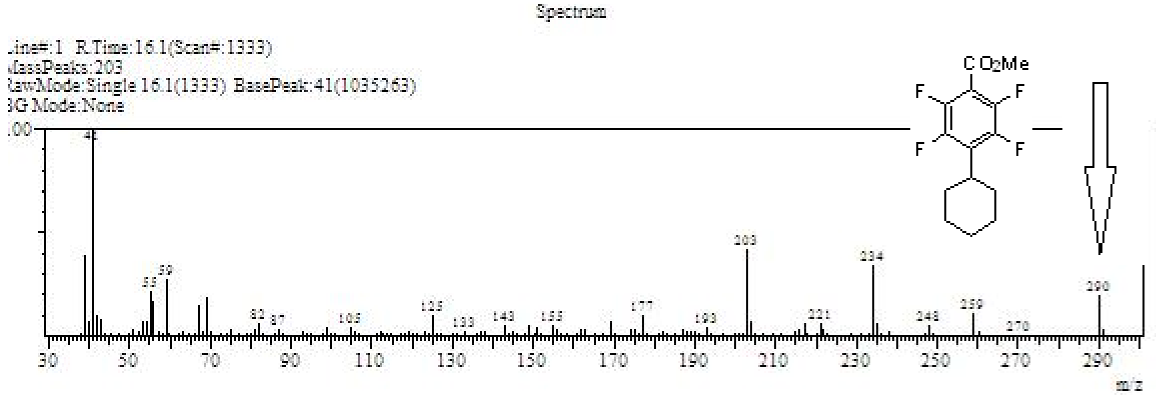

^{19}F NMR (376 MHz, Chloroform-*d*, @ rt) of S-5a 2-(4-cyclohexyl-2,3,5,6-tetrafluorophenyl)benzo[d]oxazole

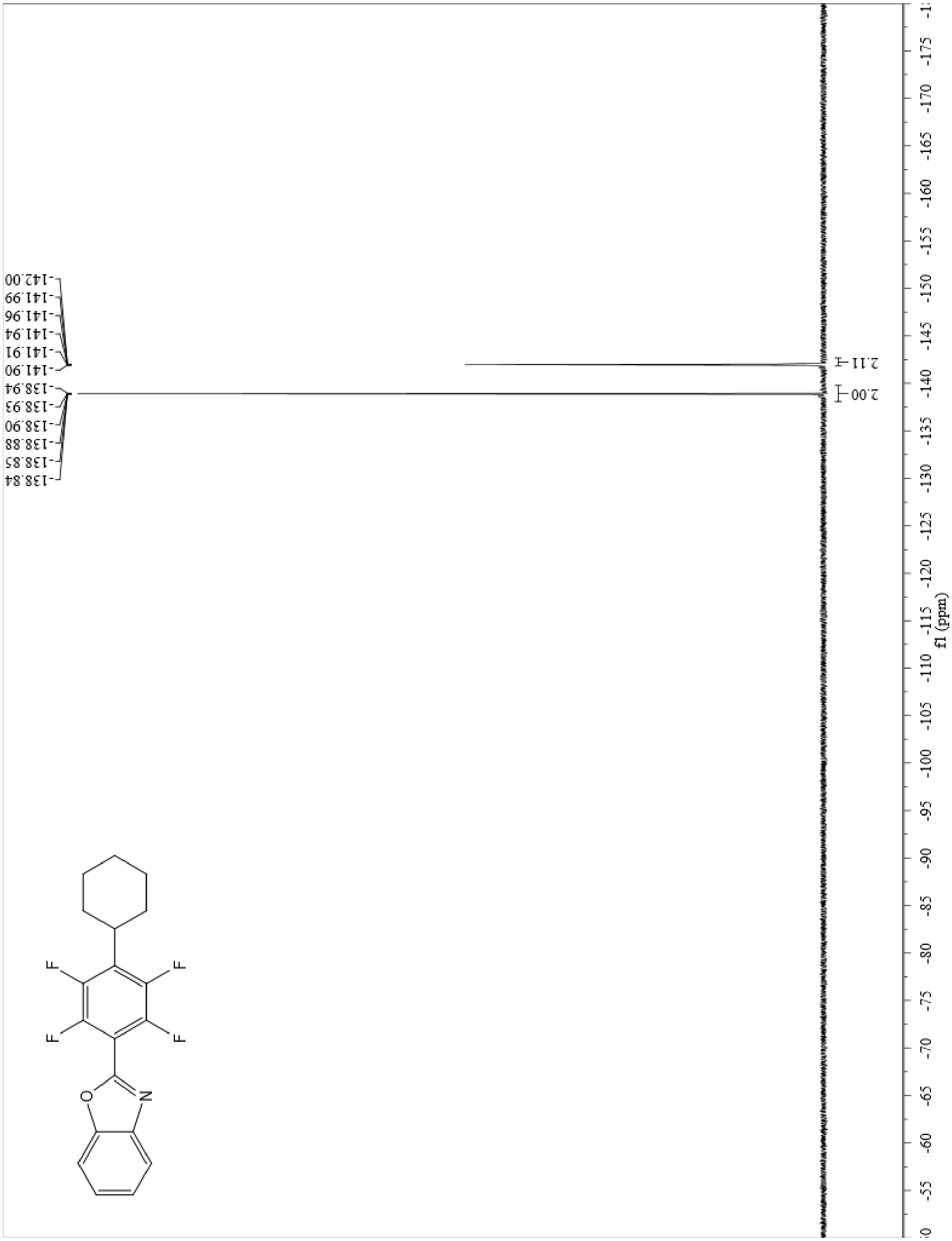

^{1}H NMR (400 MHz, Chloroform-*d*, @ rt) of S-5a 2-(4-cyclohexyl-2,3,5,6-tetrafluorophenyl)benzo[d]oxazole

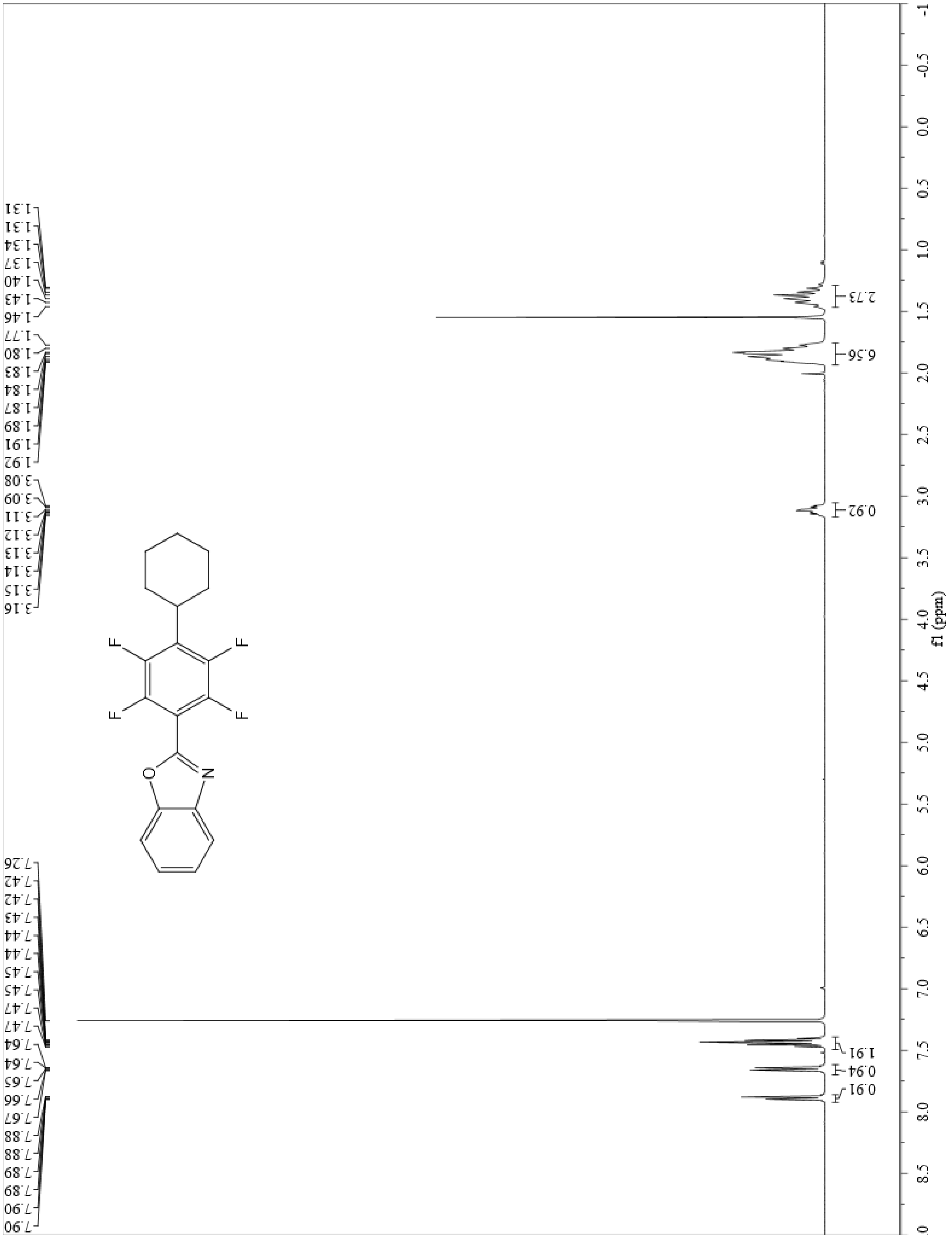

$^{13}C\{^{1}H\}$ NMR (101 MHz, Chloroform-*d*, @ rt) of S-5a 2-(4-cyclohexyl-2,3,5,6-tetrafluorophenyl)benzo[d]oxazole

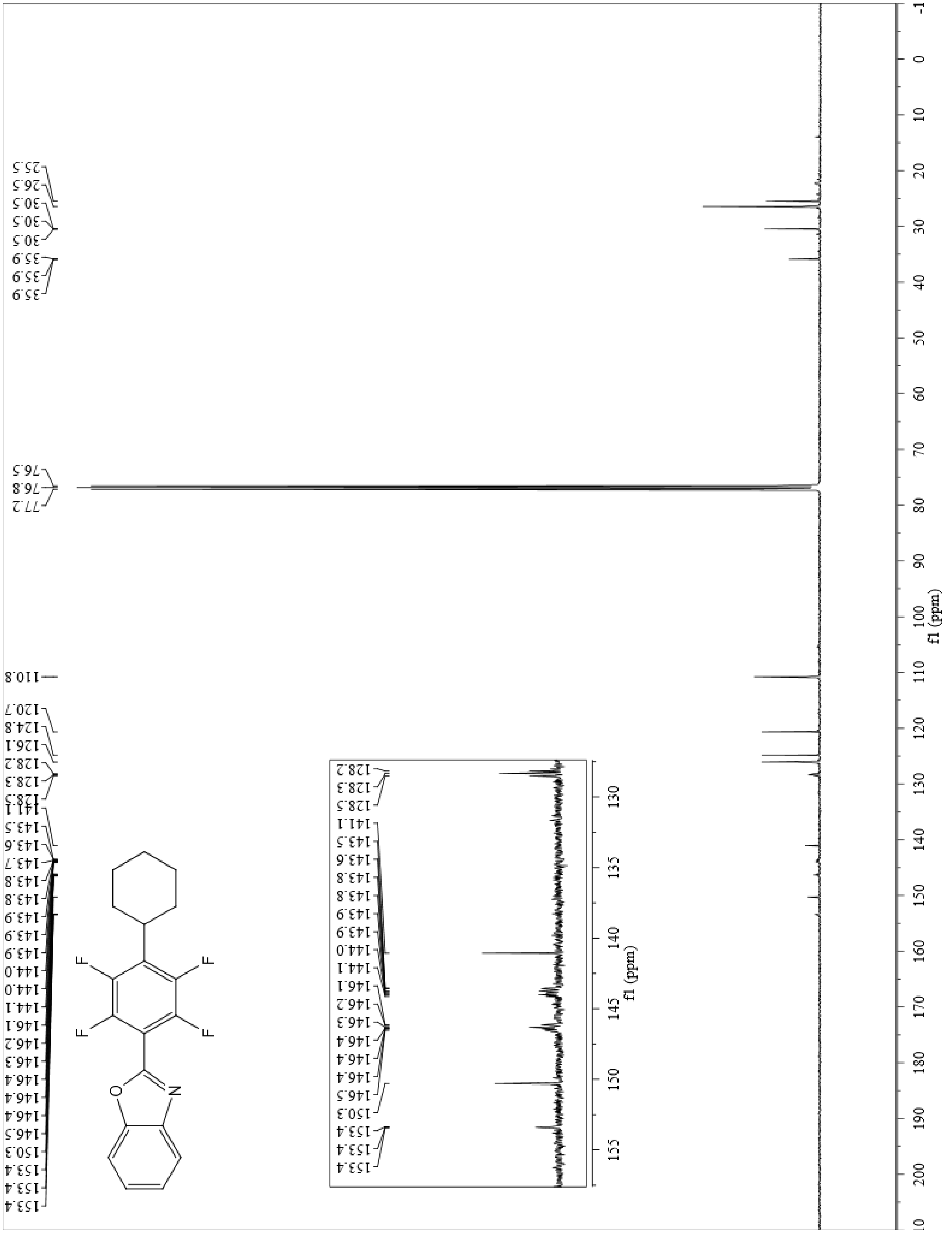

GC and MS of S-5a 2-(4-cyclohexyl-2,3,5,6-tetrafluorophenyl)benzo[d]oxazole

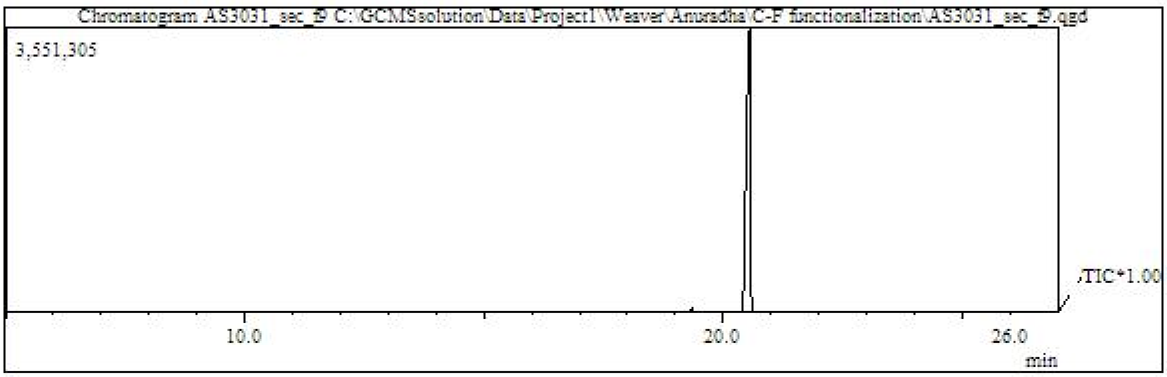

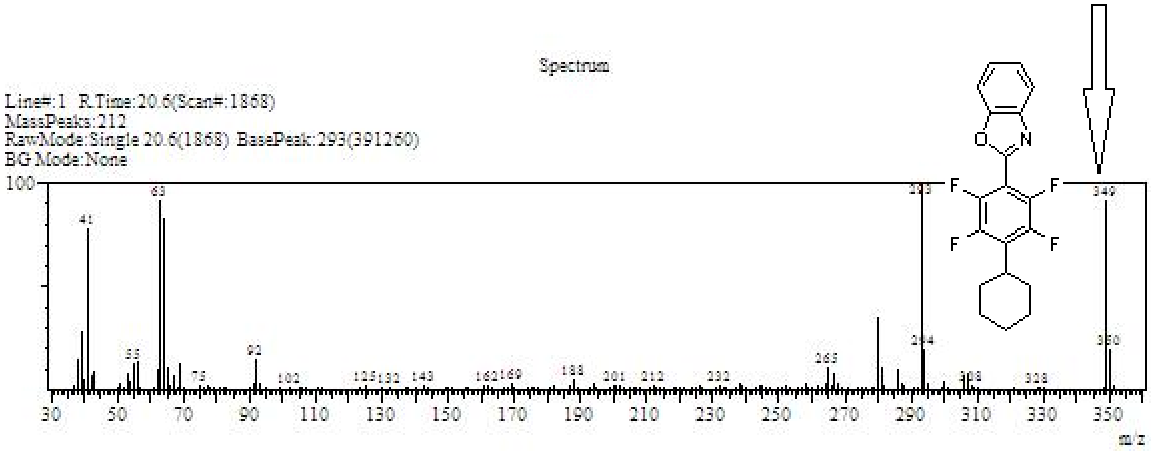

^{19}F NMR (376 MHz, Chloroform-*d*, @ rt) of S-6a (4-cyclohexyl-2,3,5,6-tetrafluorophenyl)diphenylphosphine

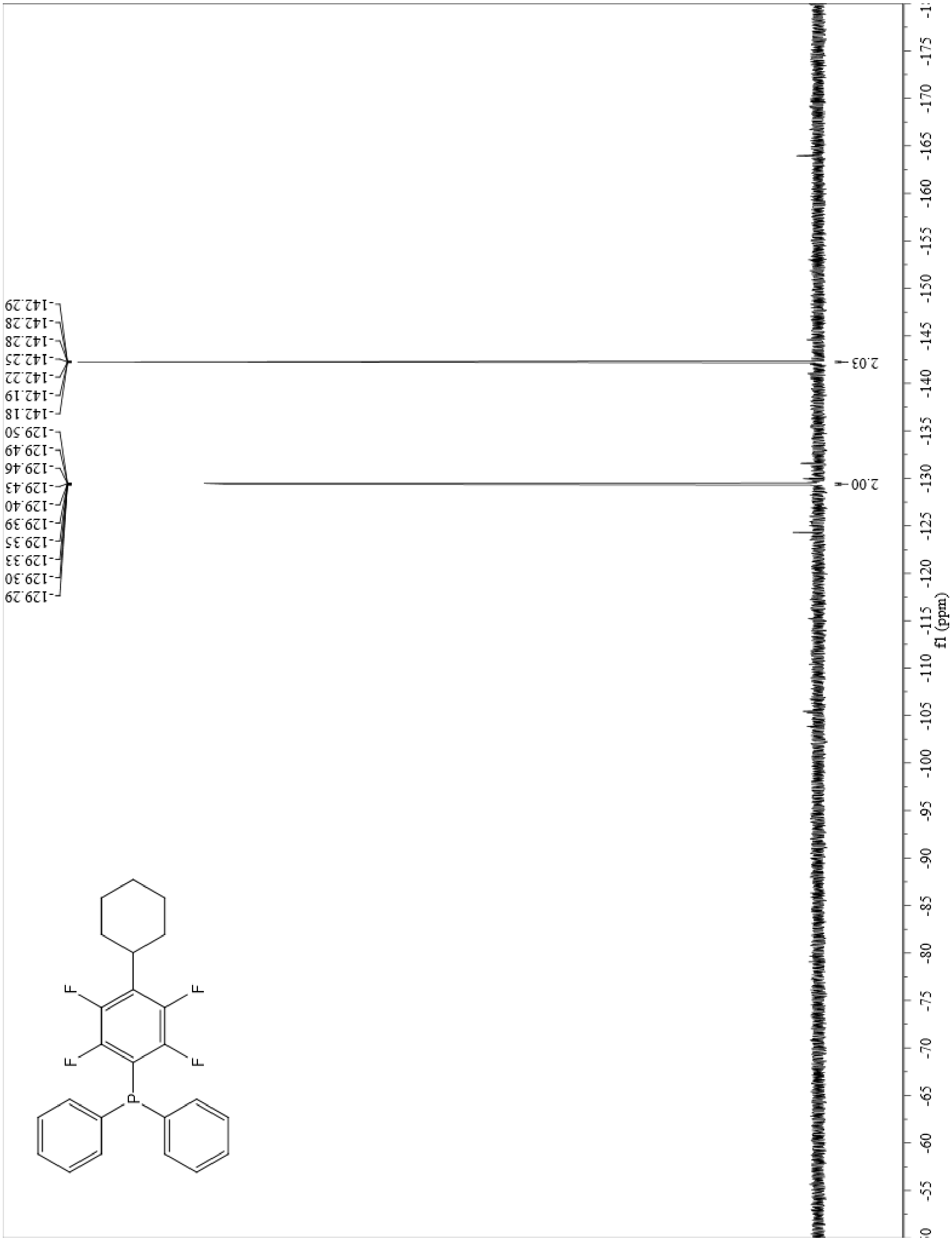

^{1}H NMR (400 MHz, Chloroform-*d*, @ rt) of S-6a (4-cyclohexyl-2,3,5,6-tetrafluorophenyl)diphenylphosphine

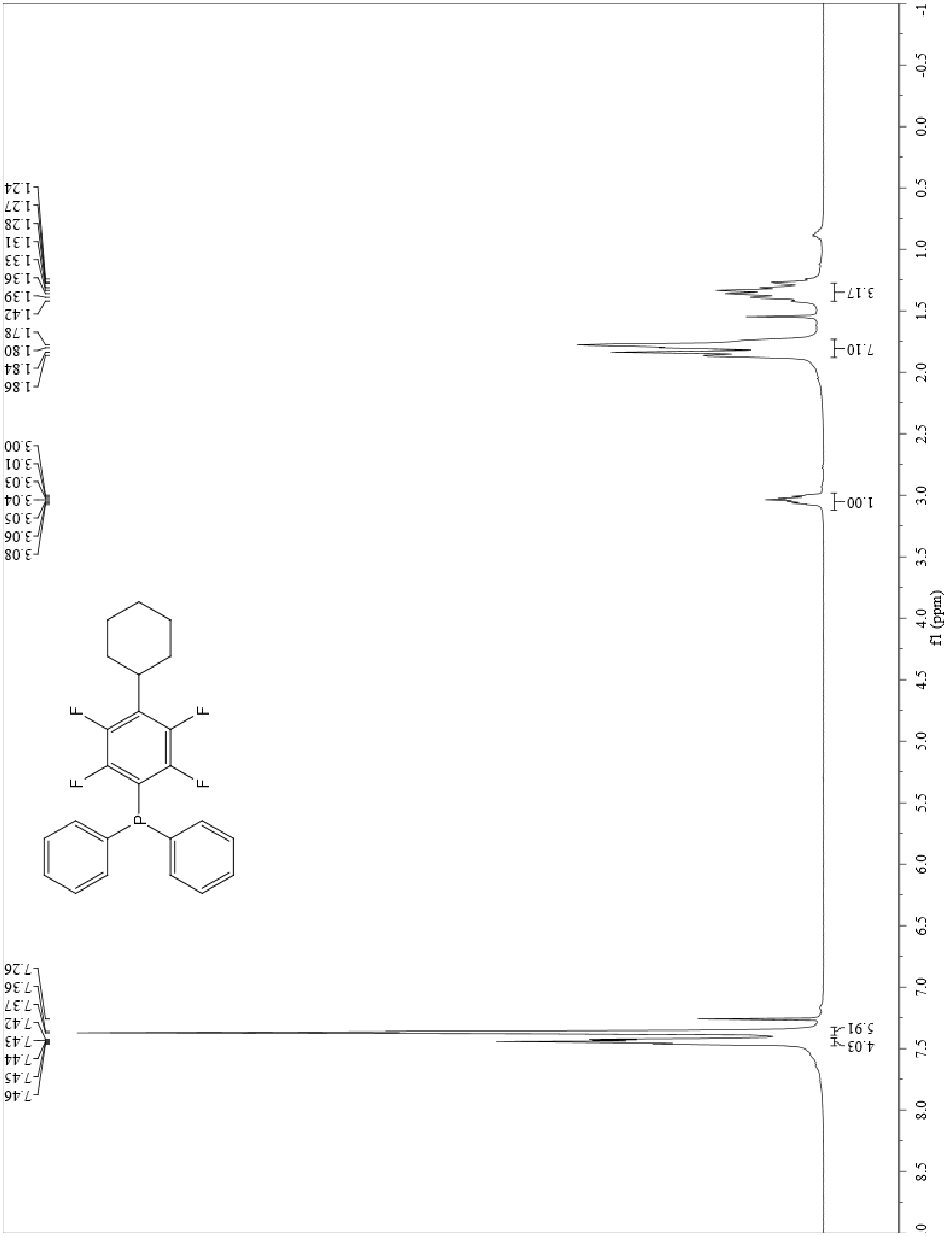

$^{13}C\{^1H\}$ NMR (101 MHz, Chloroform-*d*, @ rt) of S-6a (4-cyclohexyl-2,3,5,6-tetrafluorophenyl)diphenylphosphine

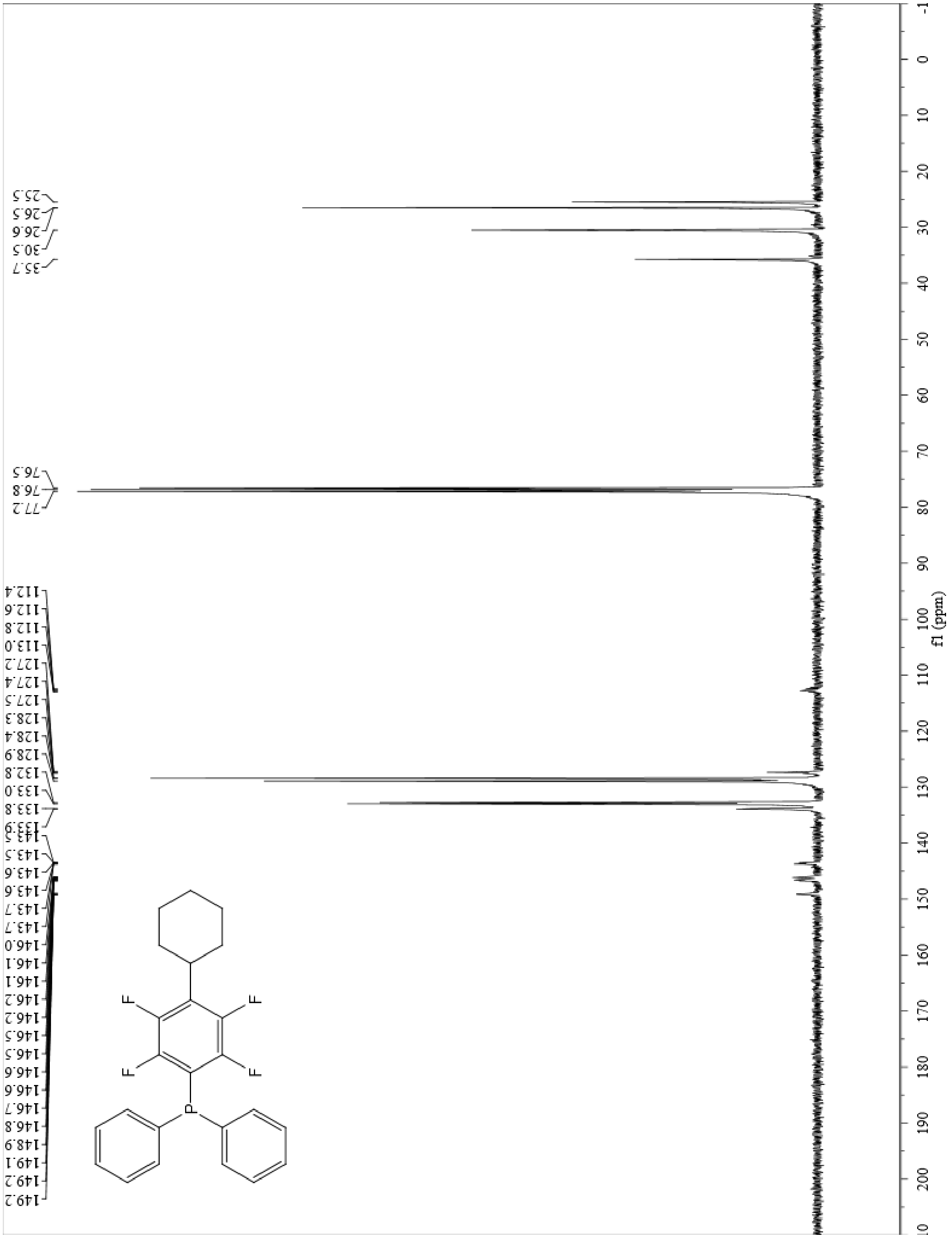

GC and MS of S-6a (4-cyclohexyl-2,3,5,6-tetrafluorophenyl)diphenylphosphine

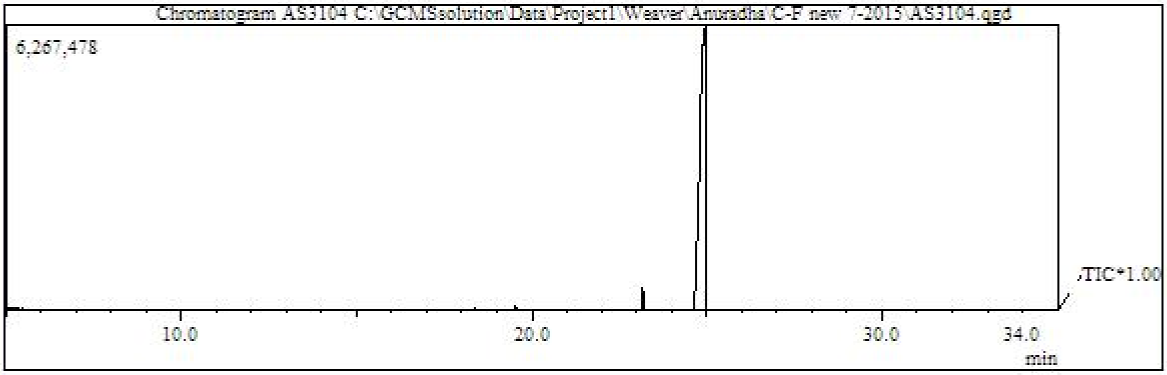

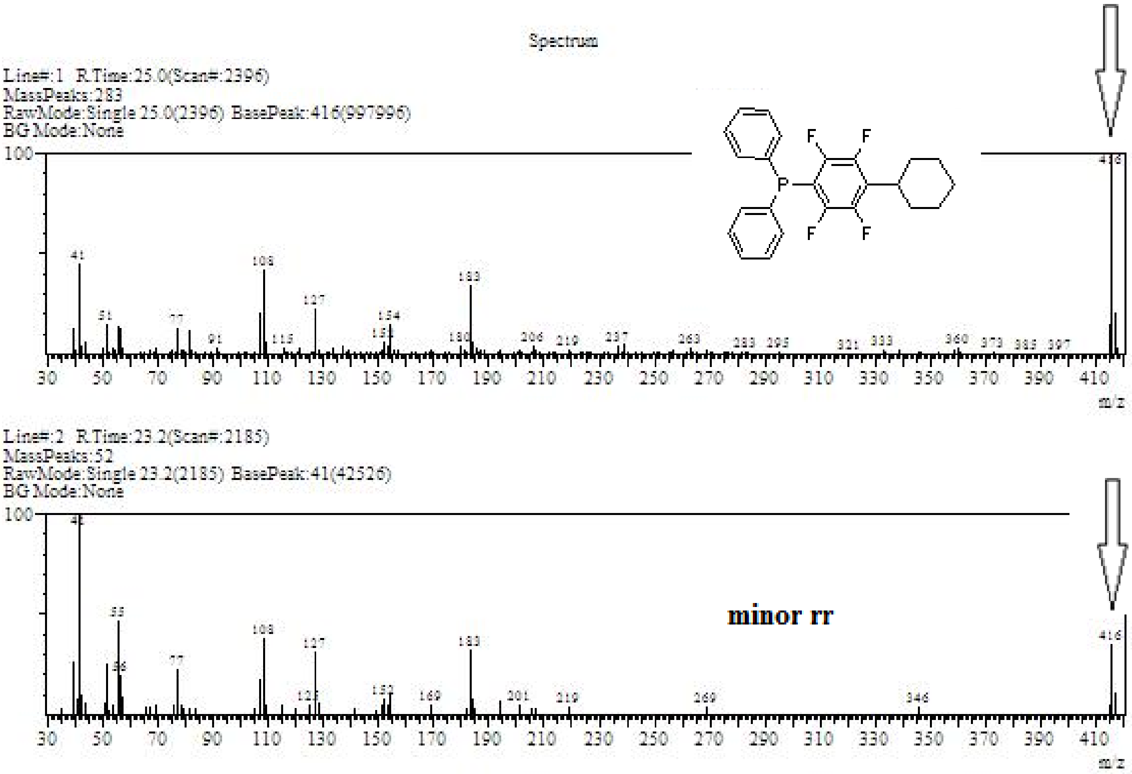

^{19}F NMR (376 MHz, Chloroform-*d*, @ rt) of S-7a 2,3,5,6-tetrafluoro-4-

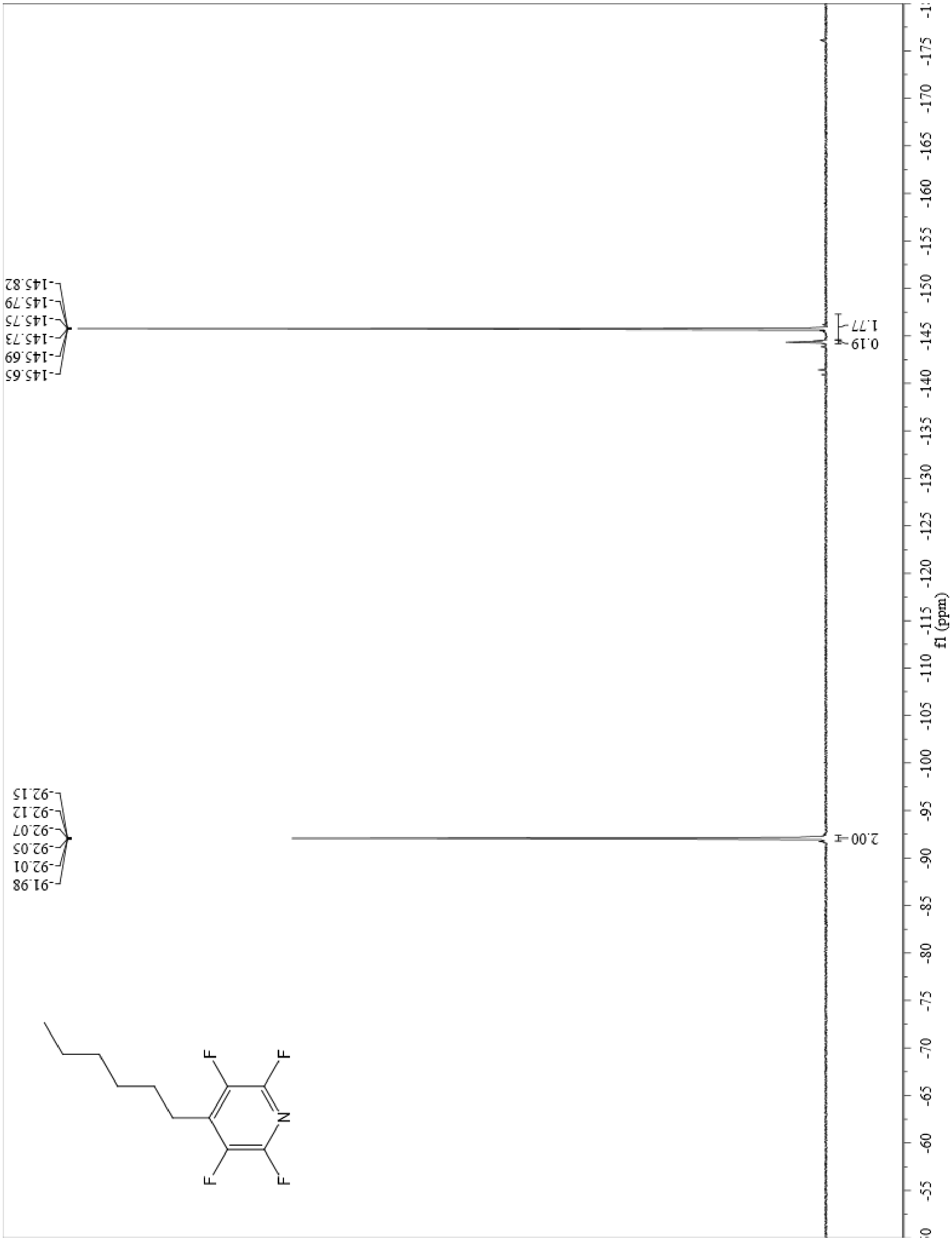

^{1}H NMR (400 MHz, Chloroform-*d*, @ rt) of S-7a 2,3,5,6-tetrafluoro-4-hexylpyridine

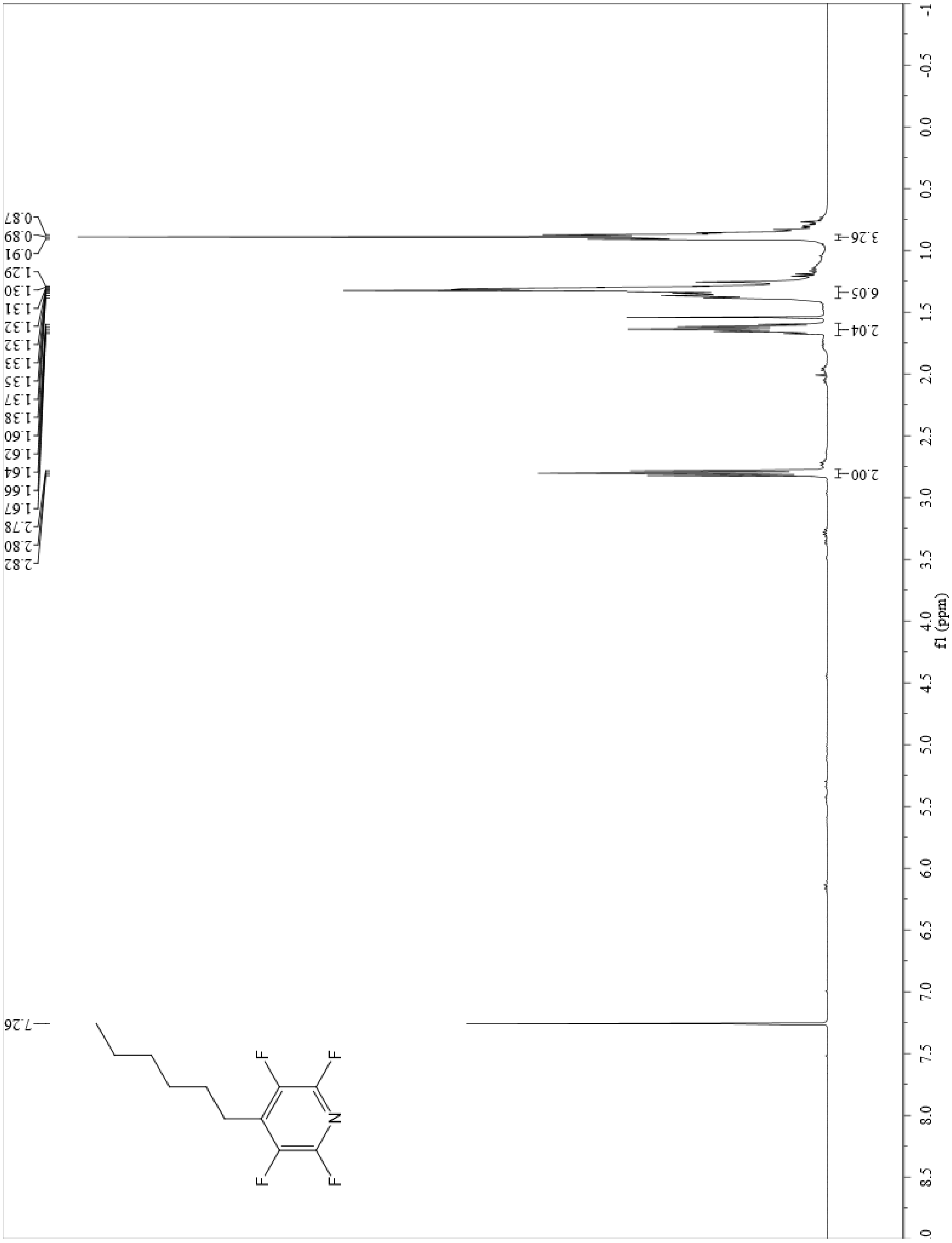

$^{13}C\{^1H\}$ NMR of (101 MHz, Chloroform-*d*, @ rt) S-7a 2,3,5,6-tetrafluoro-4-hexylpyridine

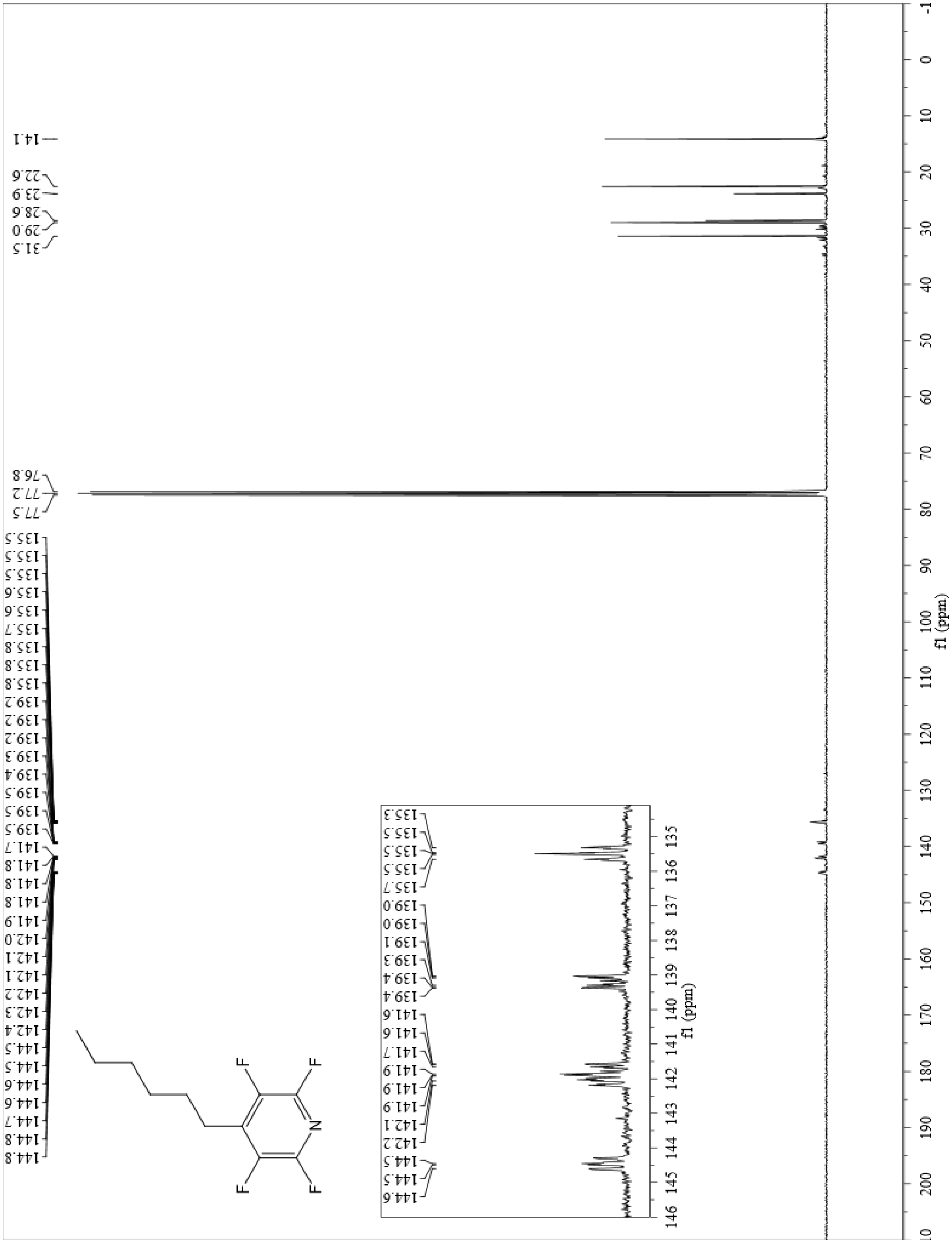

GC and MS of S-7a 2,3,5,6-tetrafluoro-4-hexylpyridine

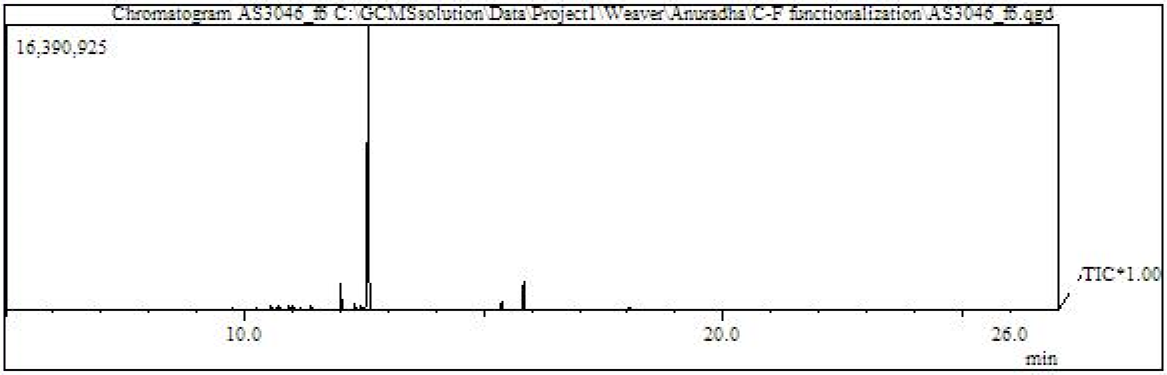

Spectrum

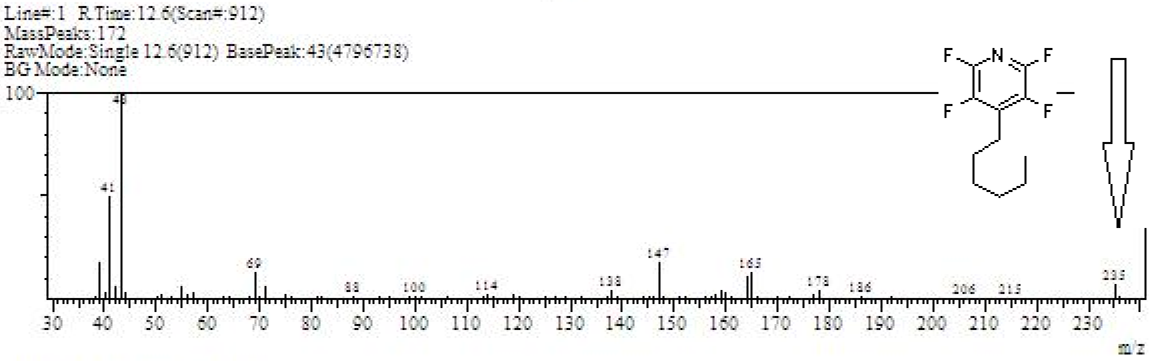

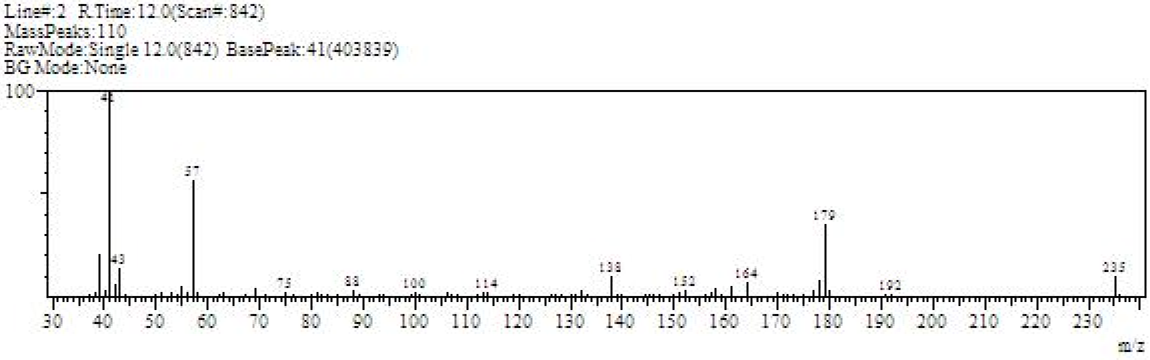

^{19}F NMR (376 MHz, Chloroform-*d*, @ rt) of S-8a 2,3,5,6-tetrafluoro-4-(tetrahydro-2H-pyran-2-yl)pyridine

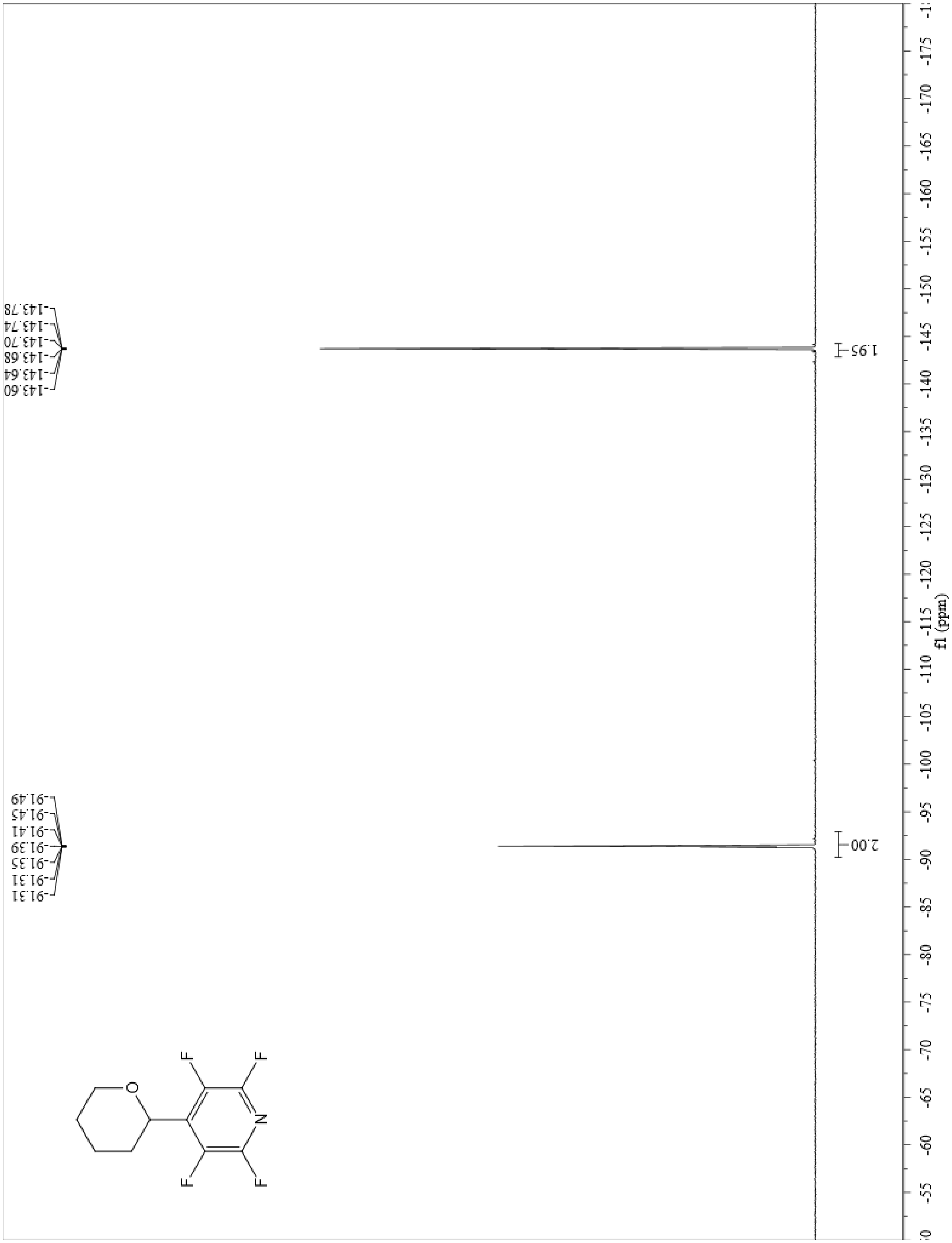

^{1}H NMR (400 MHz, Chloroform-*d*, @ rt) of S-8a 2,3,5,6-tetrafluoro-4-(tetrahydro-2H-pyran-2-yl)pyridine

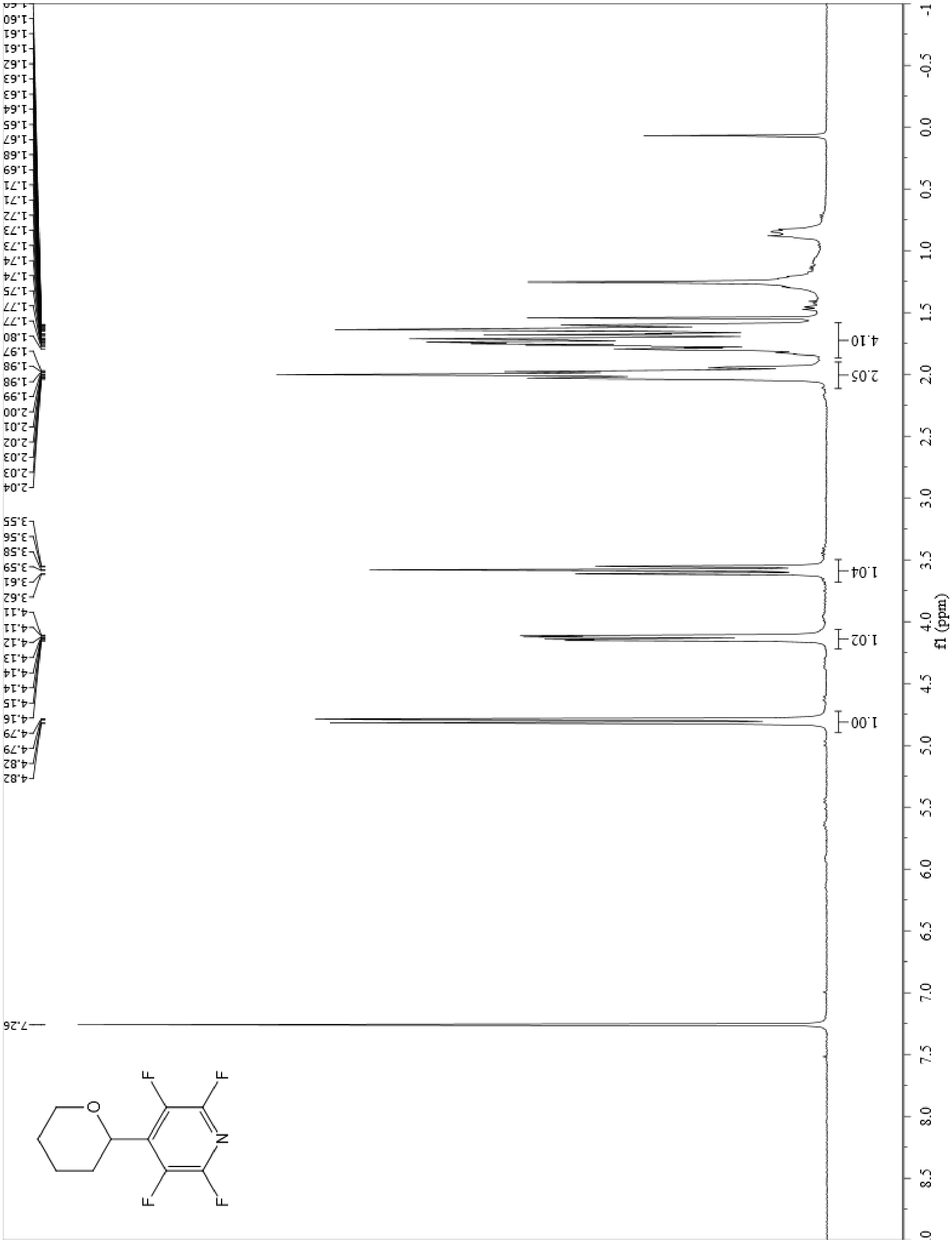

$^{13}C\{^{1}H\}$ NMR of (101 MHz, Chloroform-*d*, @ rt) S-8a 2,3,5,6-tetrafluoro-4-(tetrahydro-2H-pyran-2-yl)pyridine

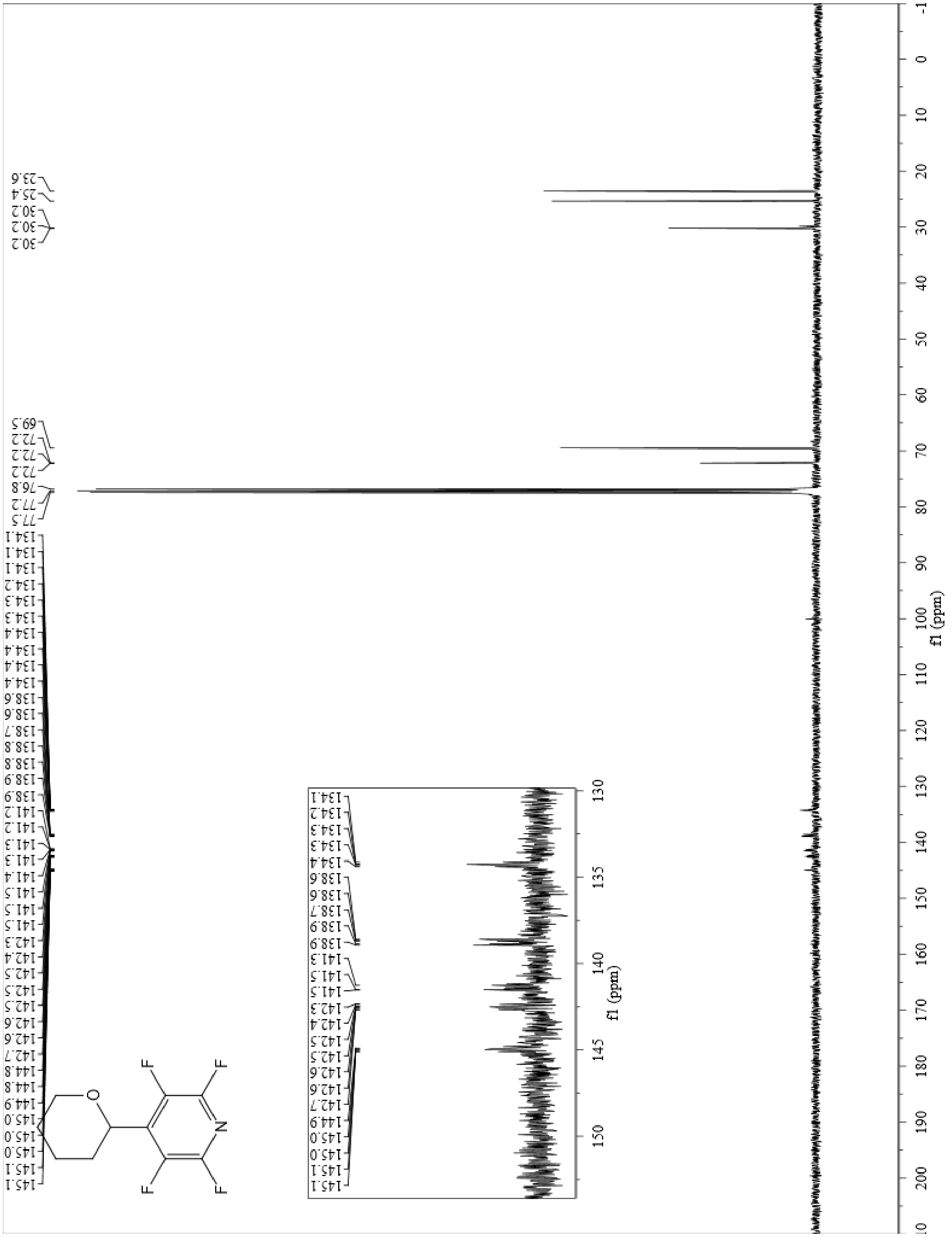

^{19}F NMR (376 MHz, Chloroform-*d*, @ rt) of S-8a 2,3,5,6-tetrafluoro-4-(tetrahydro-2H-pyran-3-yl)pyridine

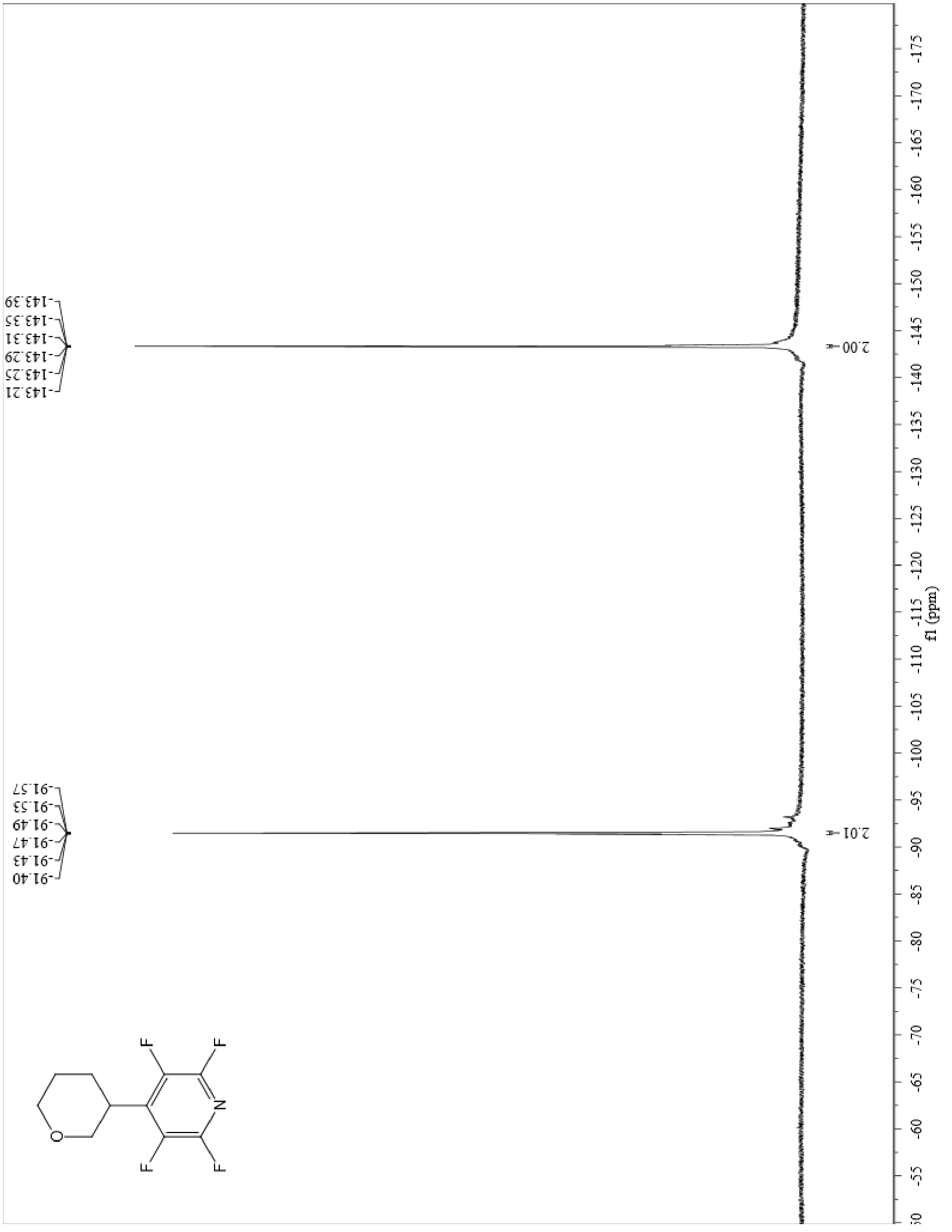

^{1}H NMR (400 MHz, Chloroform-*d*, @ rt) of S-8a 2,3,5,6-tetrafluoro-4-(tetrahydro-2H-pyran-3-yl)pyridine

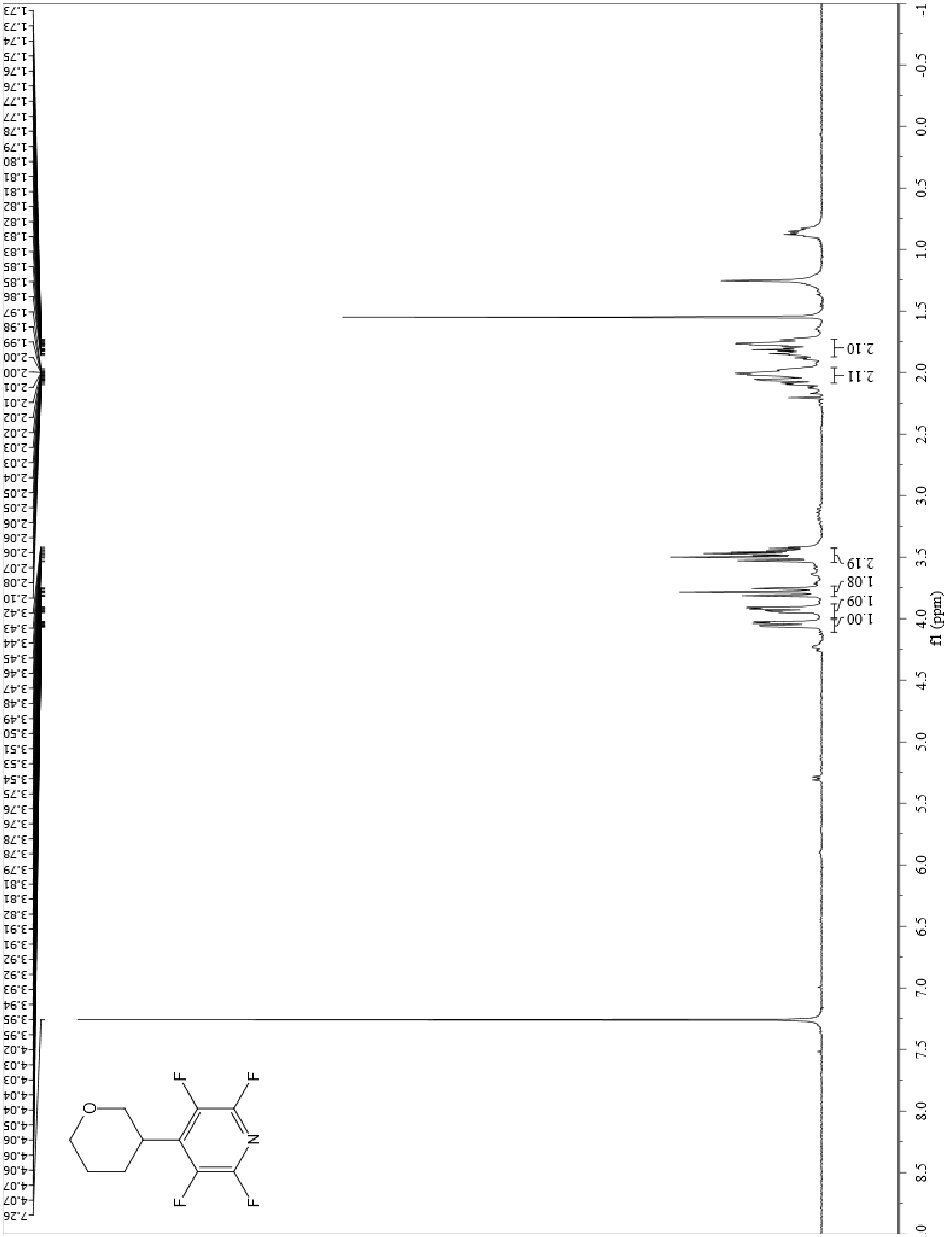

GC and MS of S-8a 2,3,5,6-tetrafluoro-4-(tetrahydro-2H-pyran-2-yl)pyridine

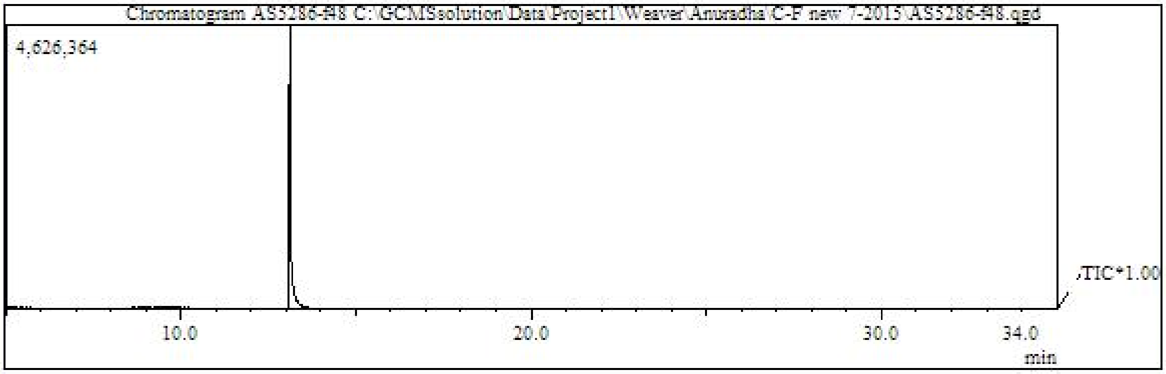

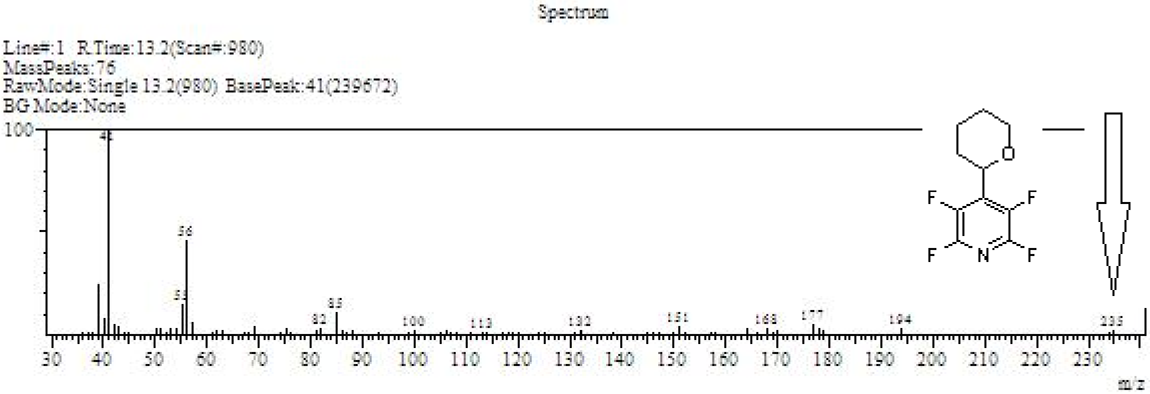

^{19}F NMR (376 MHz, Chloroform-*d*, @ rt) of S-9a 3-(perfluoropyridin-4-yl)cyclohexanone

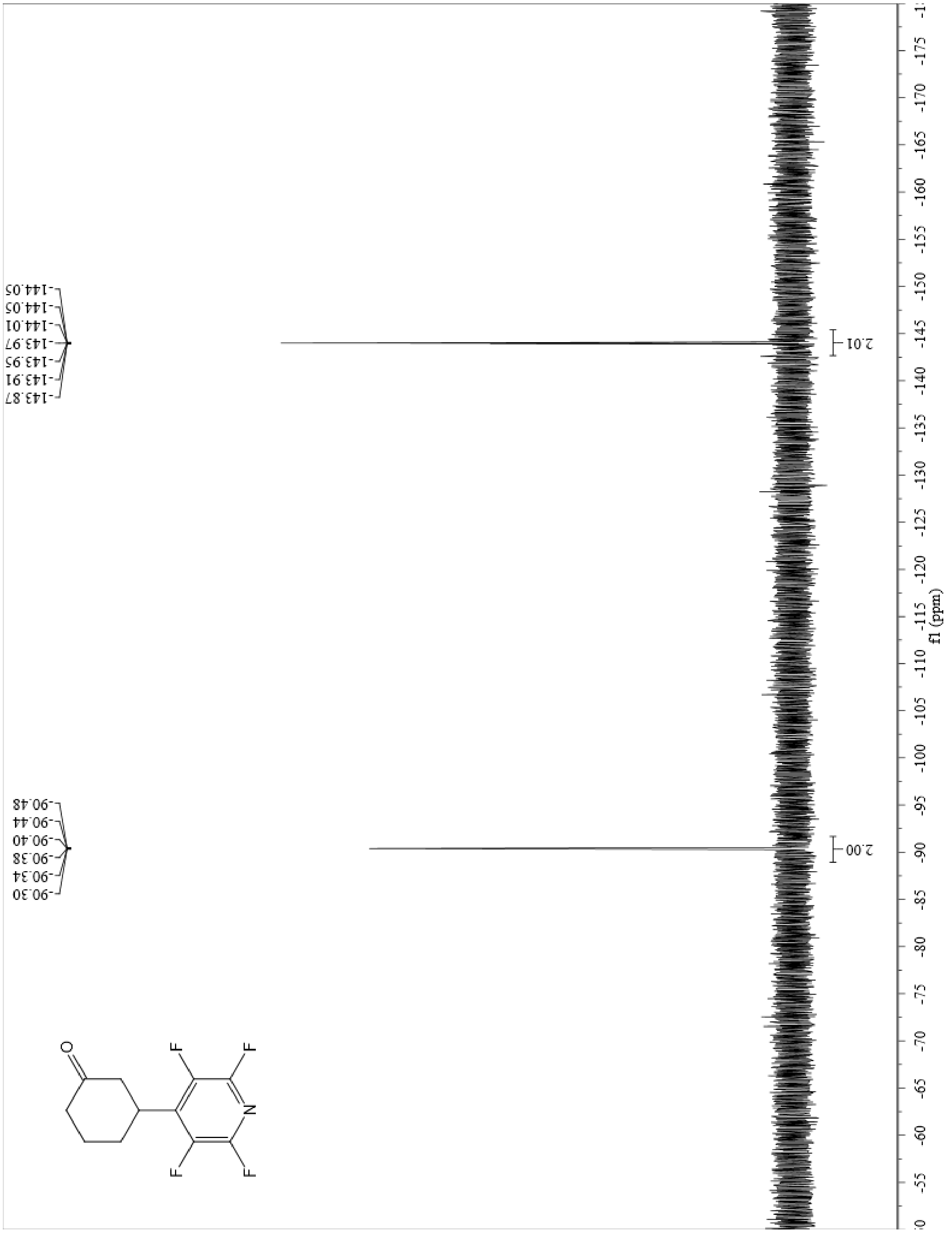

^{1}H NMR (400 MHz, Chloroform-*d*, @ rt) of S-9a 3-(perfluoropyridin-4-yl)cyclohexanone

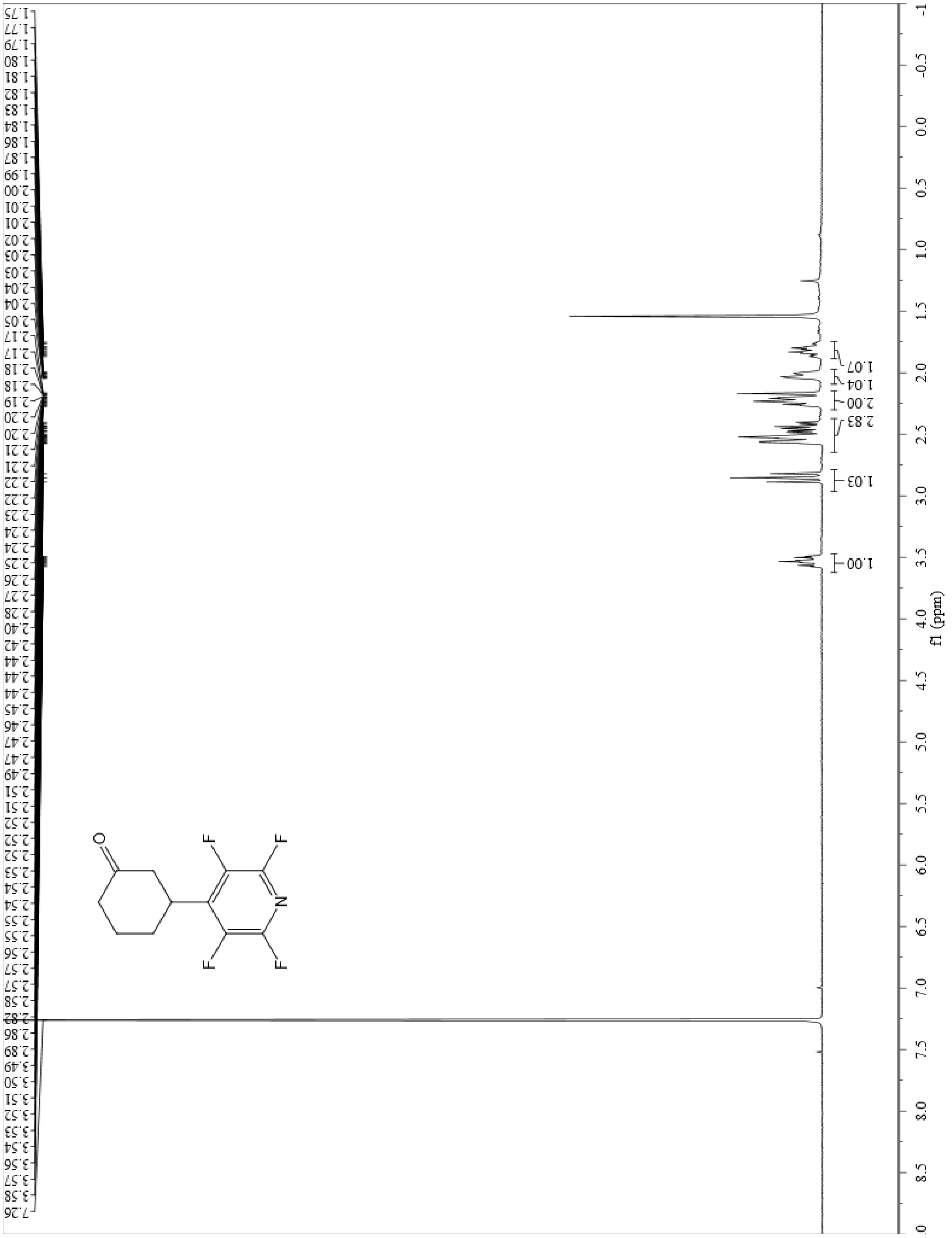

$^{13}C\{^1H\}$ NMR (101 MHz, Chloroform-*d*, @ rt) of S-9a 3-(perfluoropyridin-4-yl)cyclohexanone

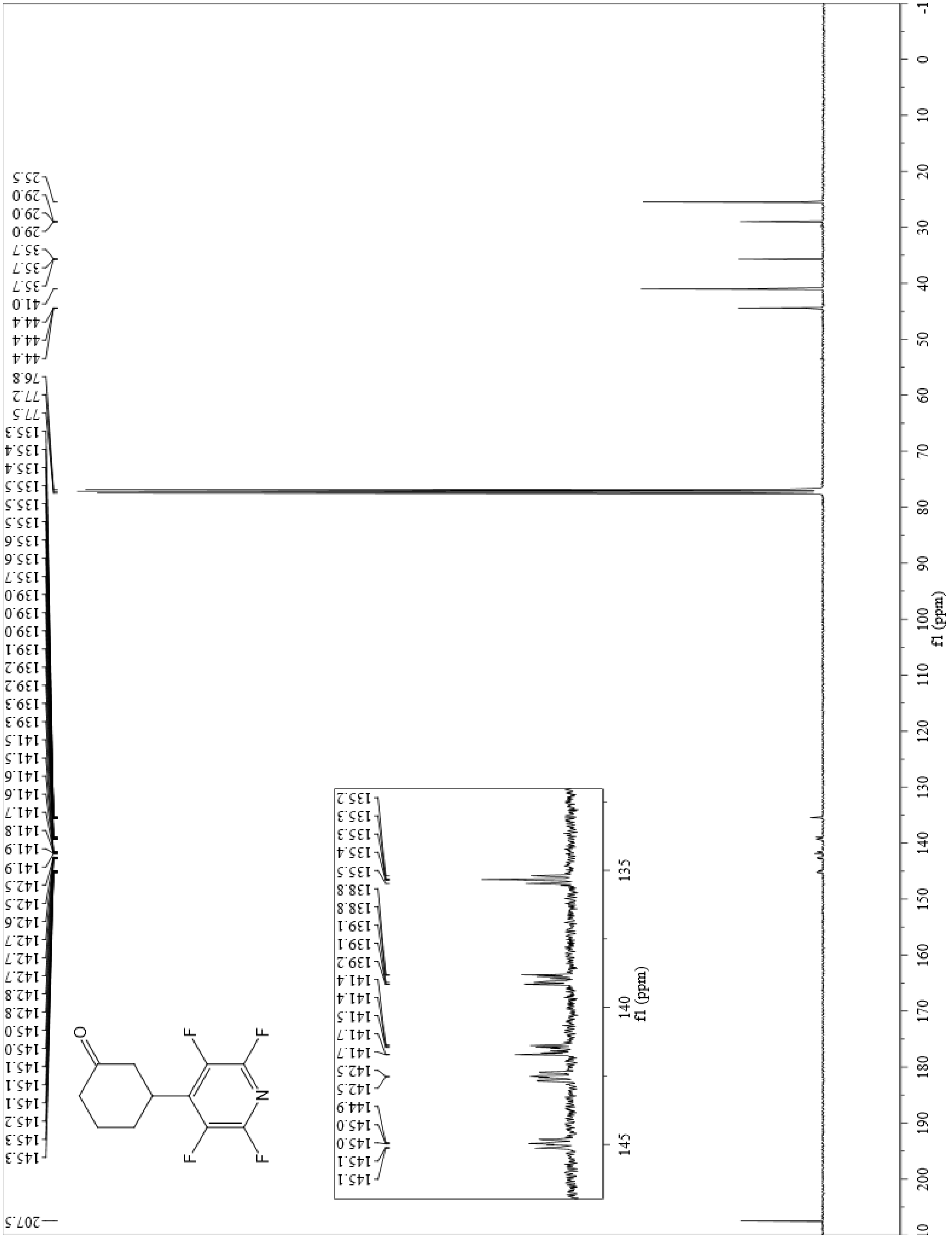

GC and MS of S-9a 3-(perfluoropyridin-4-yl)cyclohexanone

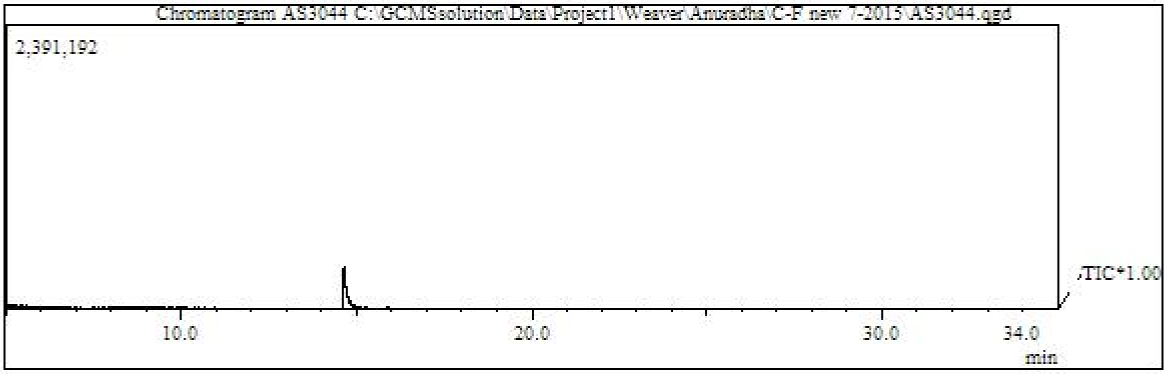

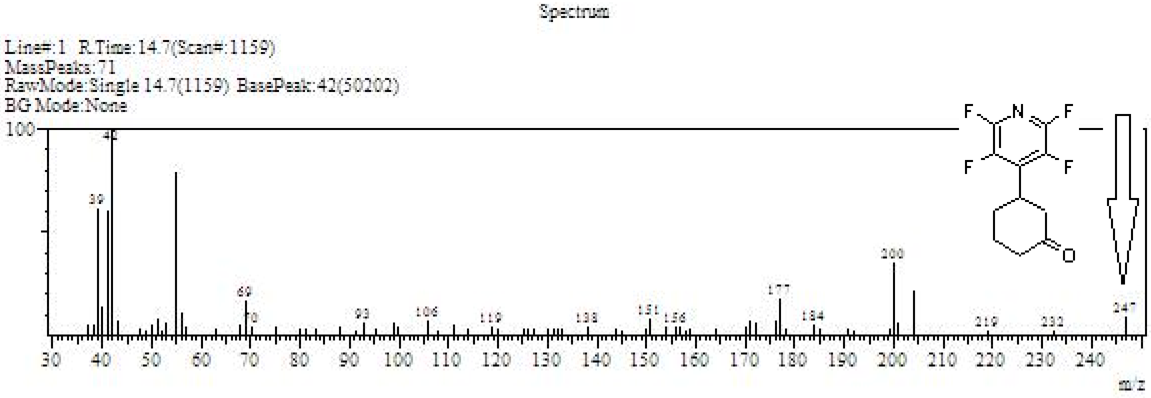

^{19}F NMR (376 MHz, Chloroform-*d*, @ rt) of S-10a 3-methyl-2-(perfluoropyridin-4-yl)butan-1-ol

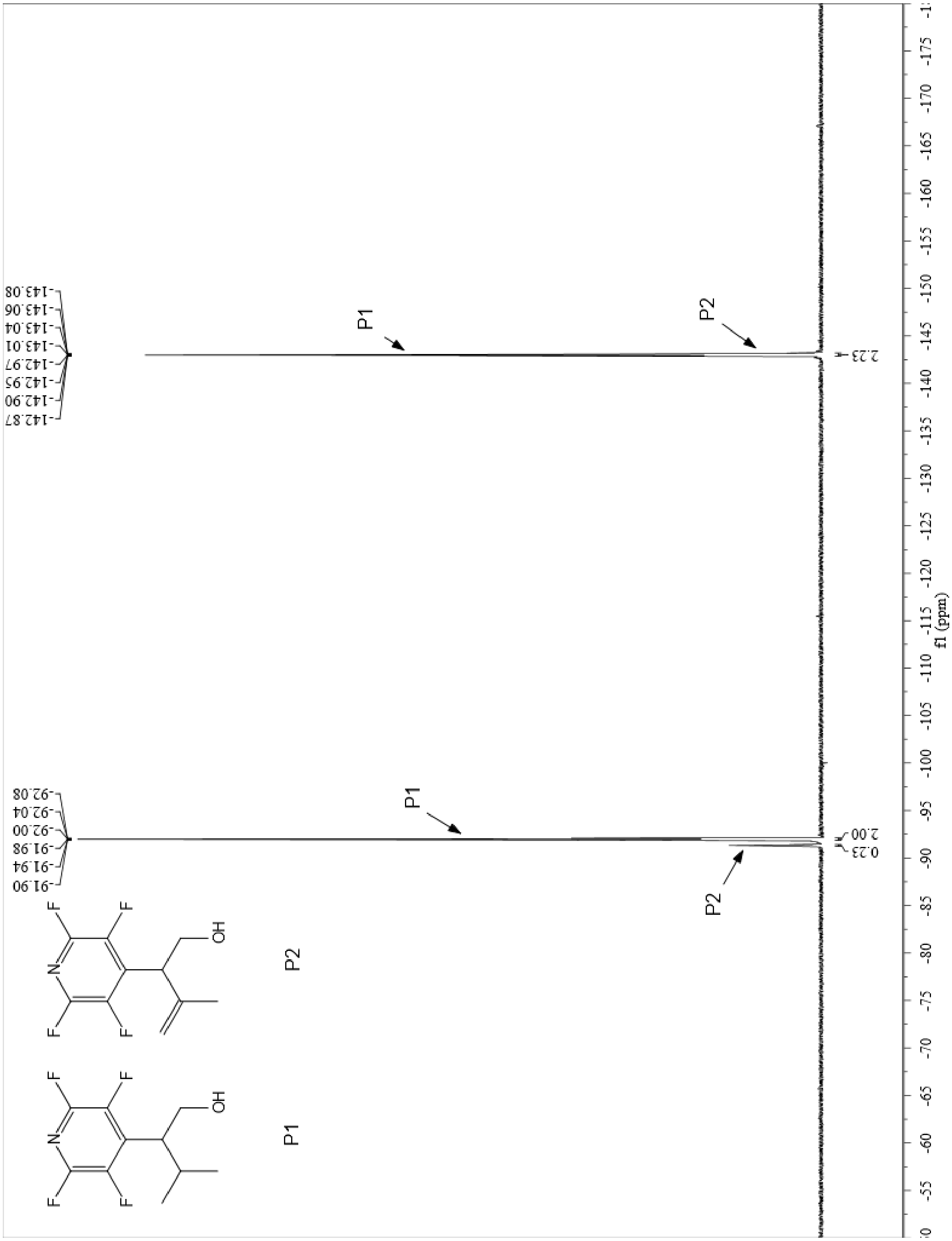

^{1}H NMR (400 MHz, Chloroform-*d*, @ rt) of S-10a 3-methyl-2-(perfluoropyridin-4-yl)butan-1-ol

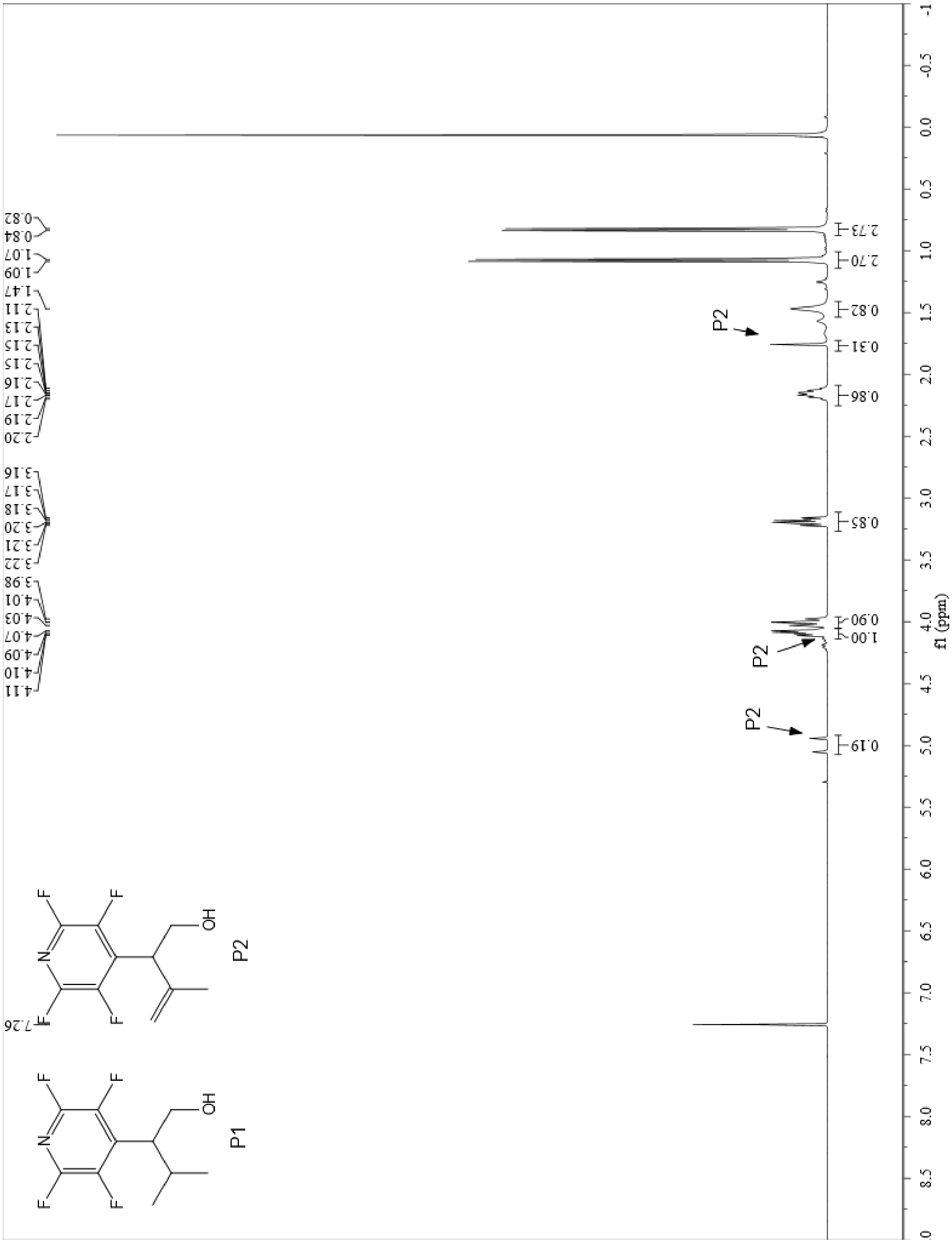

$^{13}C\{^{1}H\}$ NMR (101 MHz, Chloroform-*d*, @ rt) of S-10a 3-methyl-2-(perfluoropyridin-4-yl)butan-1-ol

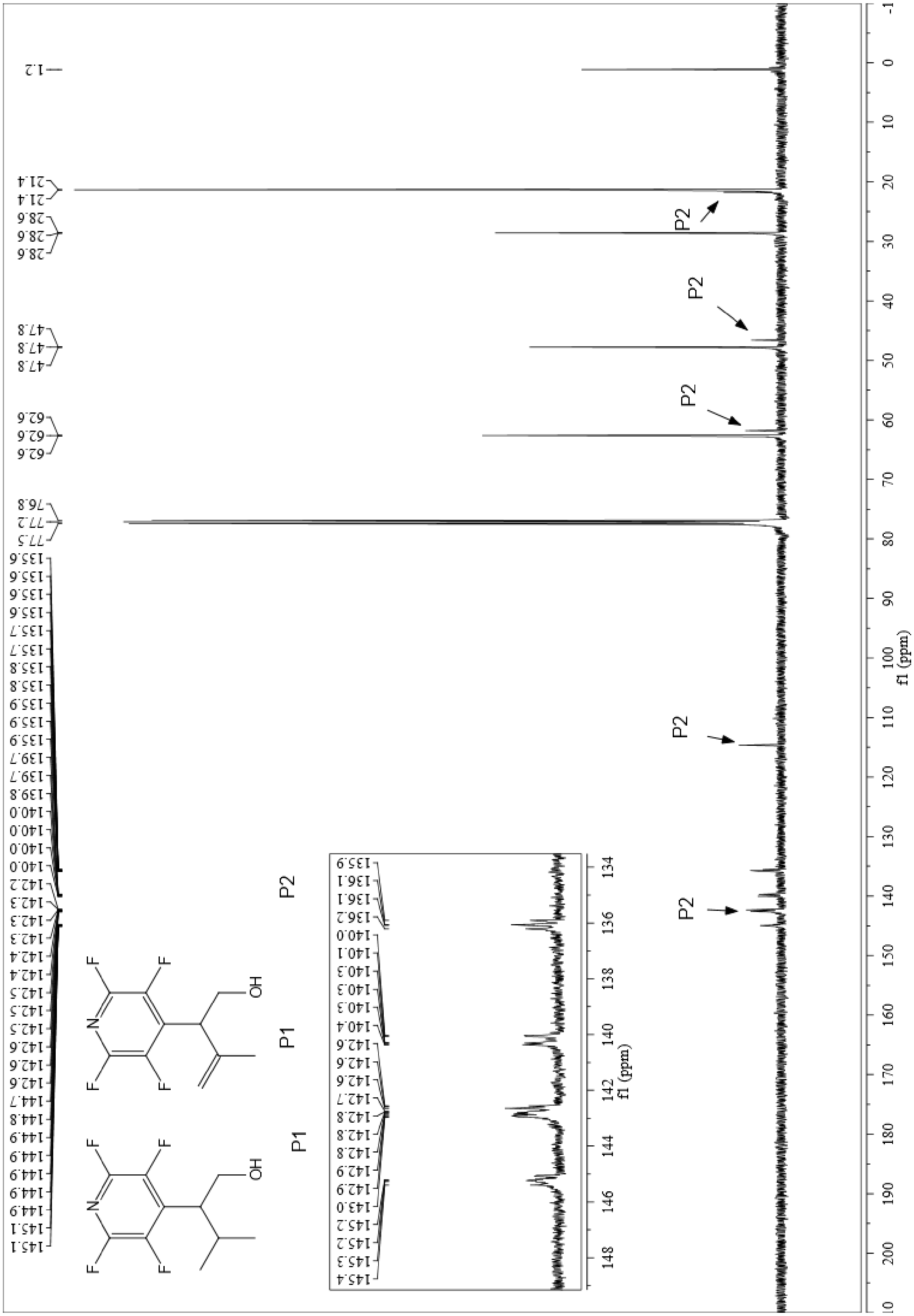

GC and MS of S-10a 3-methyl-2-(perfluoropyridin-4-yl)butan-1-ol

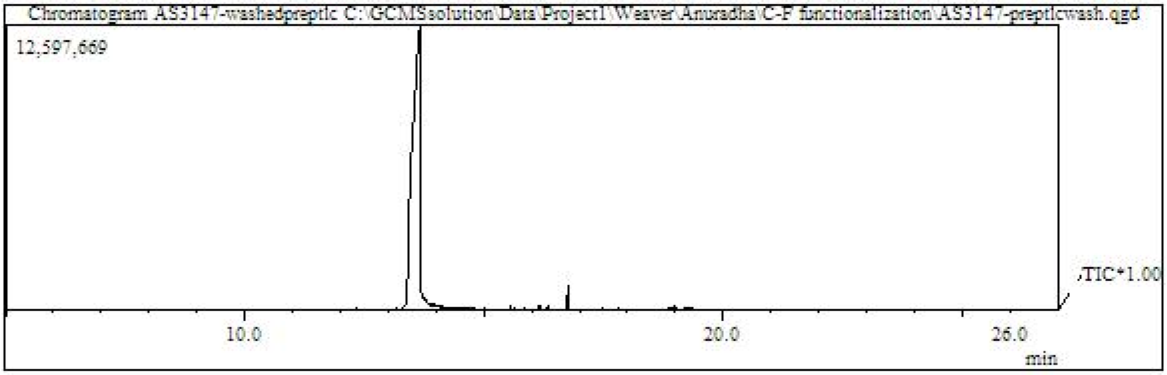

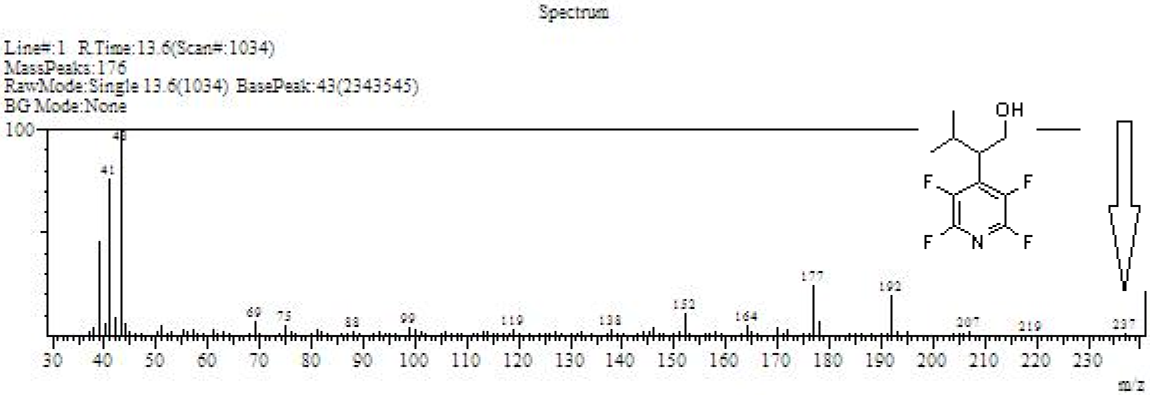

^{19}F NMR (376 MHz, Chloroform-*d*, @ rt) of S-11a 6-(perfluoropyridin-4-yl)hexan-1-ol

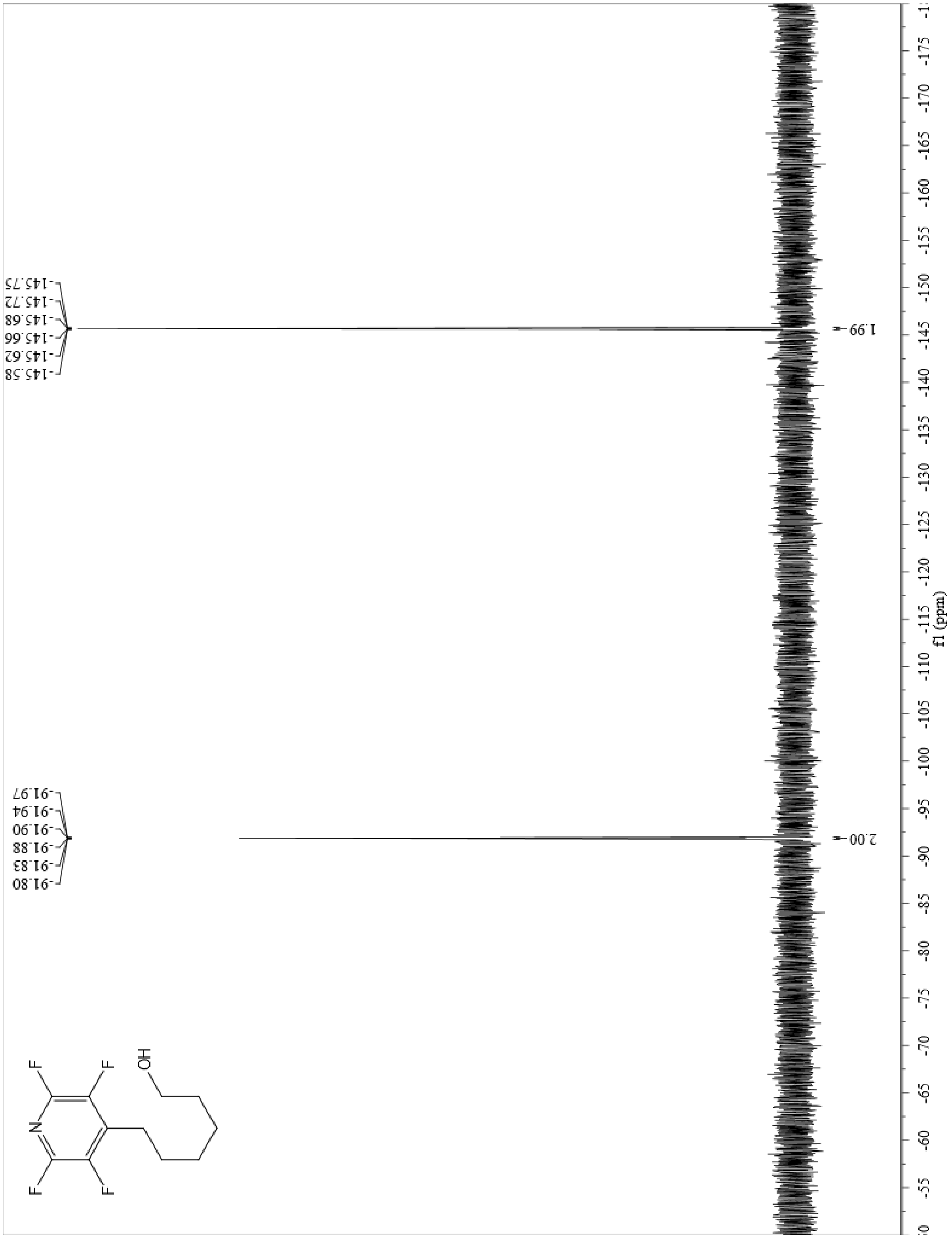

^{1}H NMR (400 MHz, Chloroform-*d*, @ rt) of S-11a 6-(perfluoropyridin-4-yl)hexan-1-ol

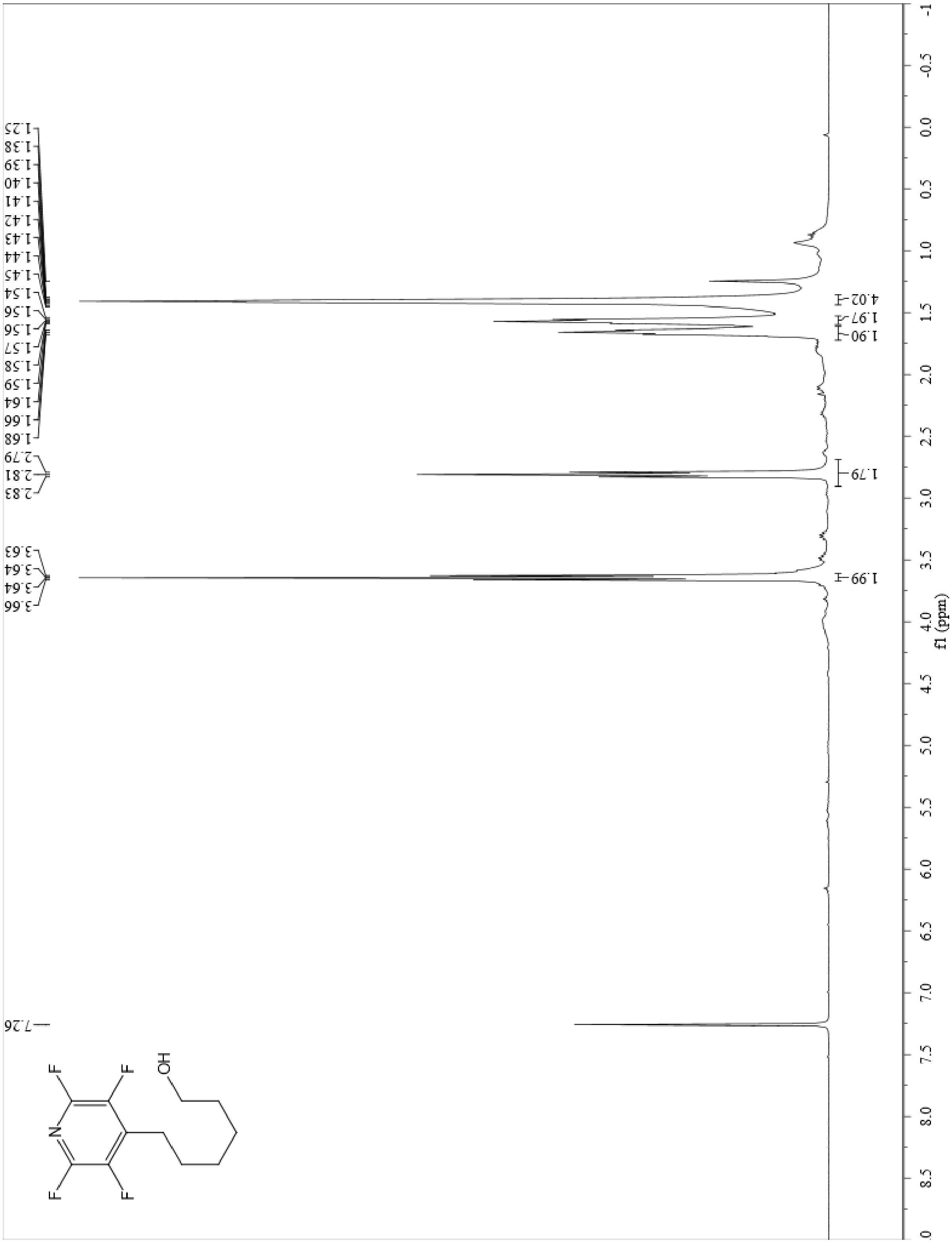

$^{13}C\{^1H\}$ **NMR (101 MHz, Chloroform-*d*, @ rt) of S-11a 6-(perfluoropyridin-4-yl)hexan-1-ol**

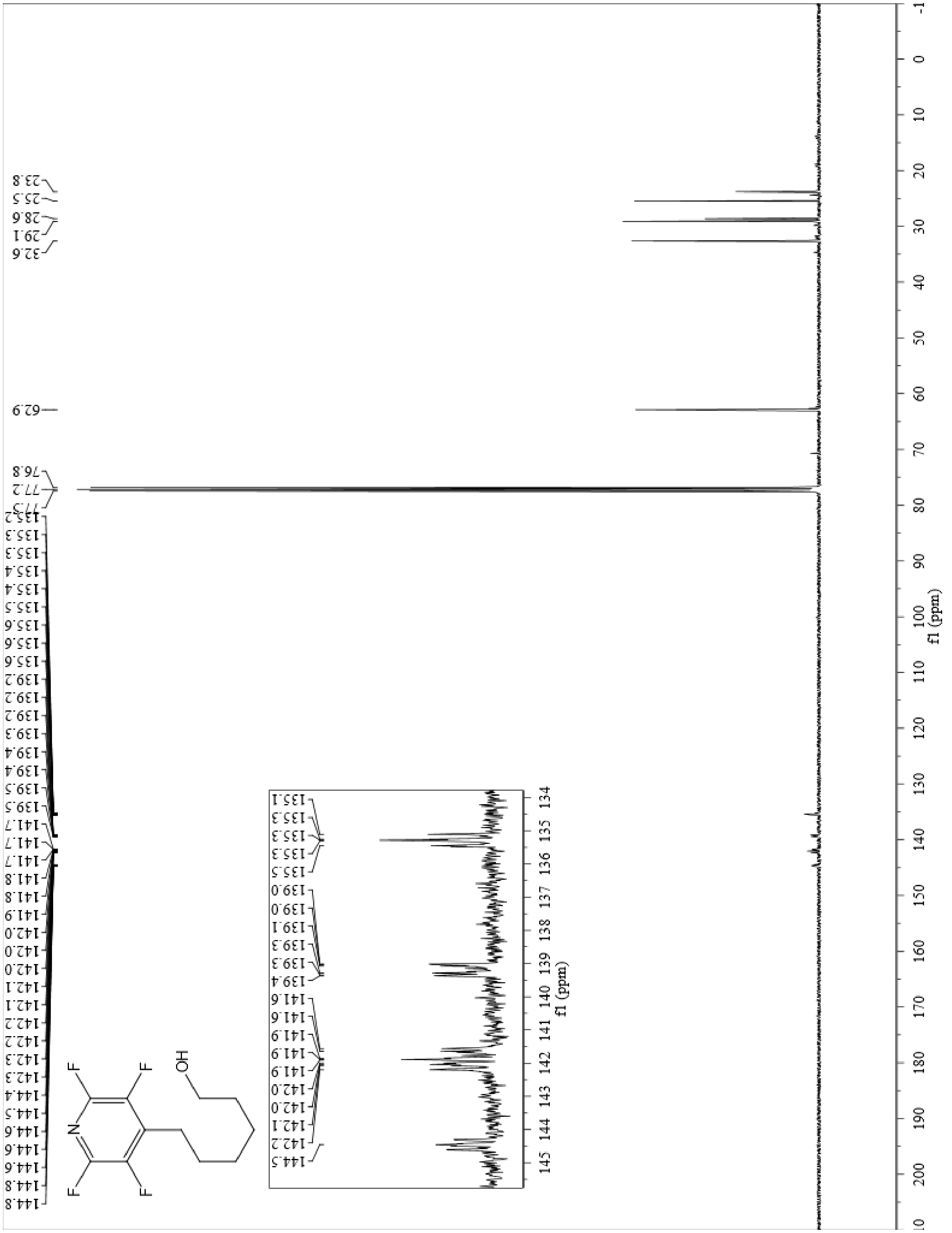

GC and MS of S-11a 6-(perfluoropyridin-4-yl)hexan-1-ol

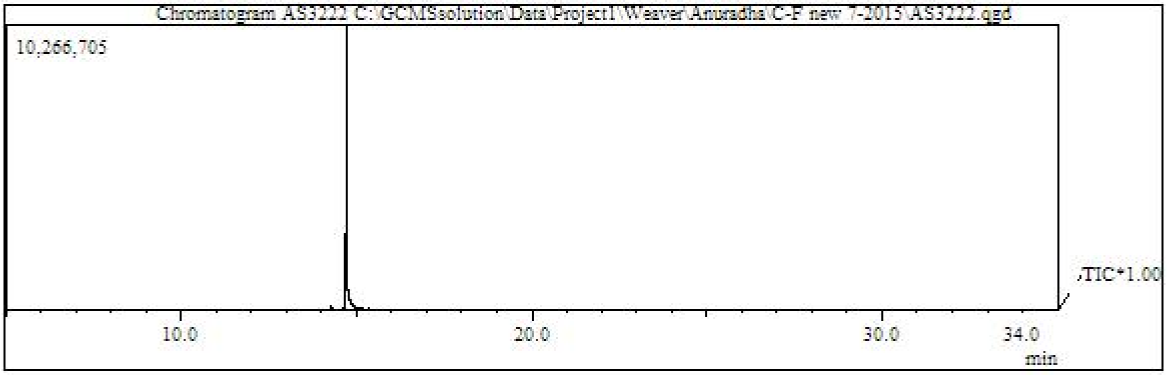

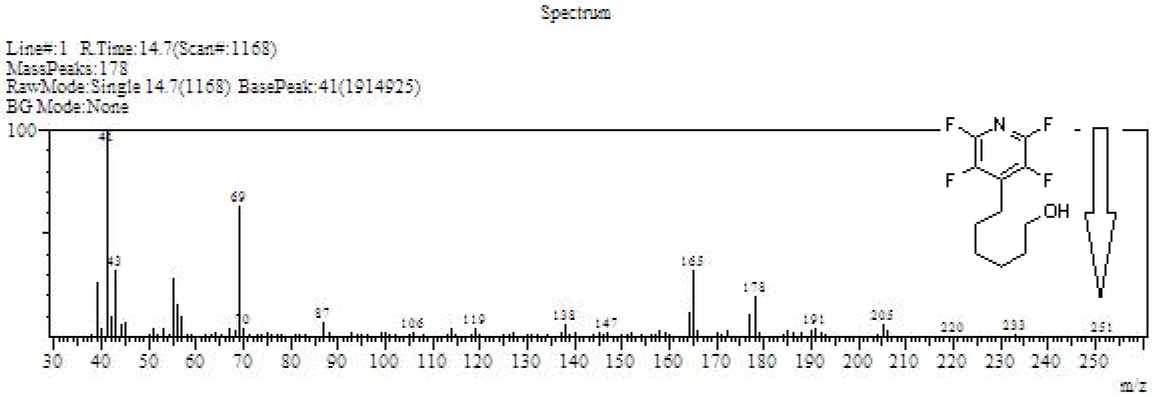

^{19}F NMR of S-12a 3-methyl-2-(perfluoropyridin-3-yl)butan-1-ol (376 MHz, Chloroform-*d*, @ rt)

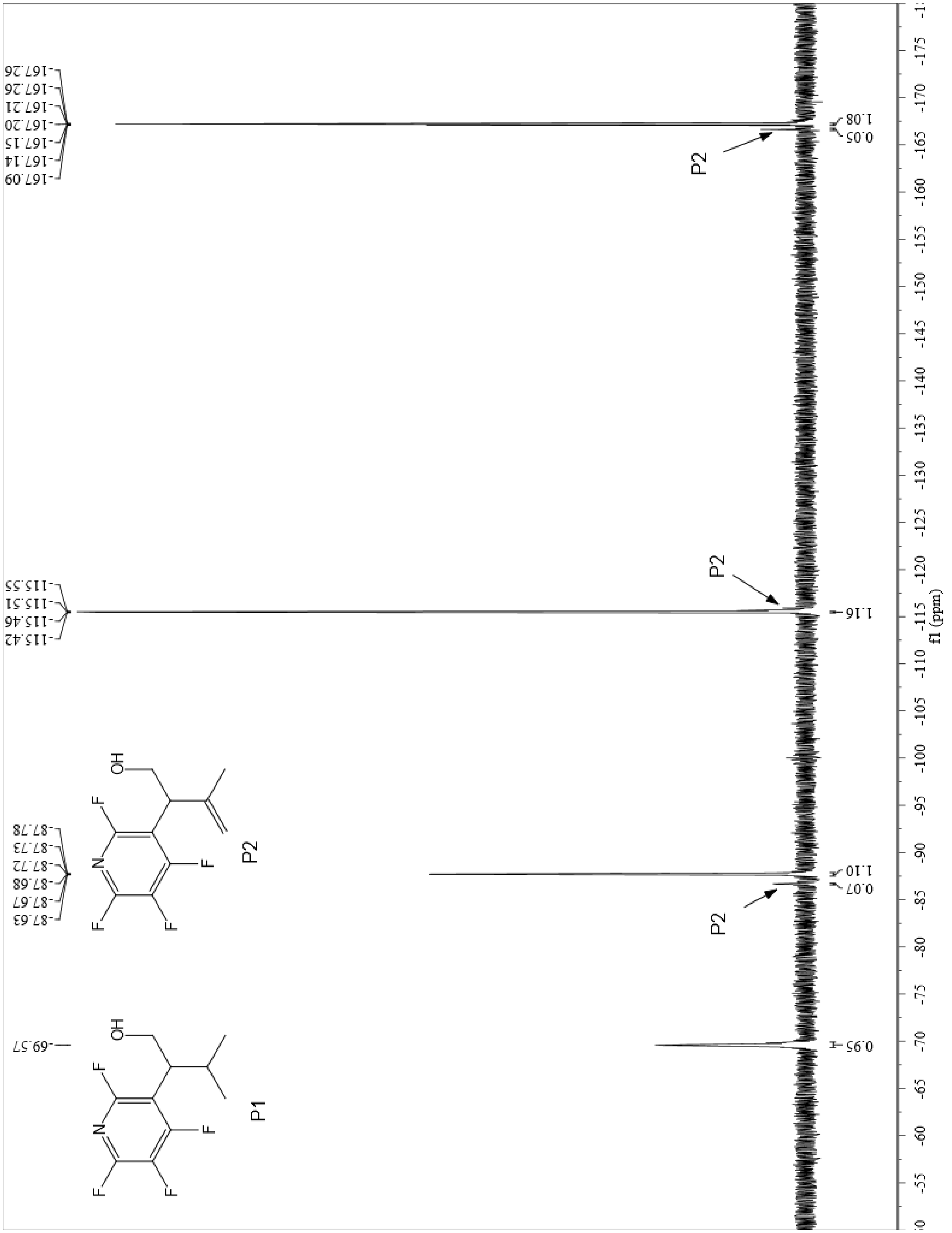

^{1}H NMR (400 MHz, Chloroform-*d*, @ rt) of S-12a 3-methyl-2-(perfluoropyridin-3-yl)butan-1-ol

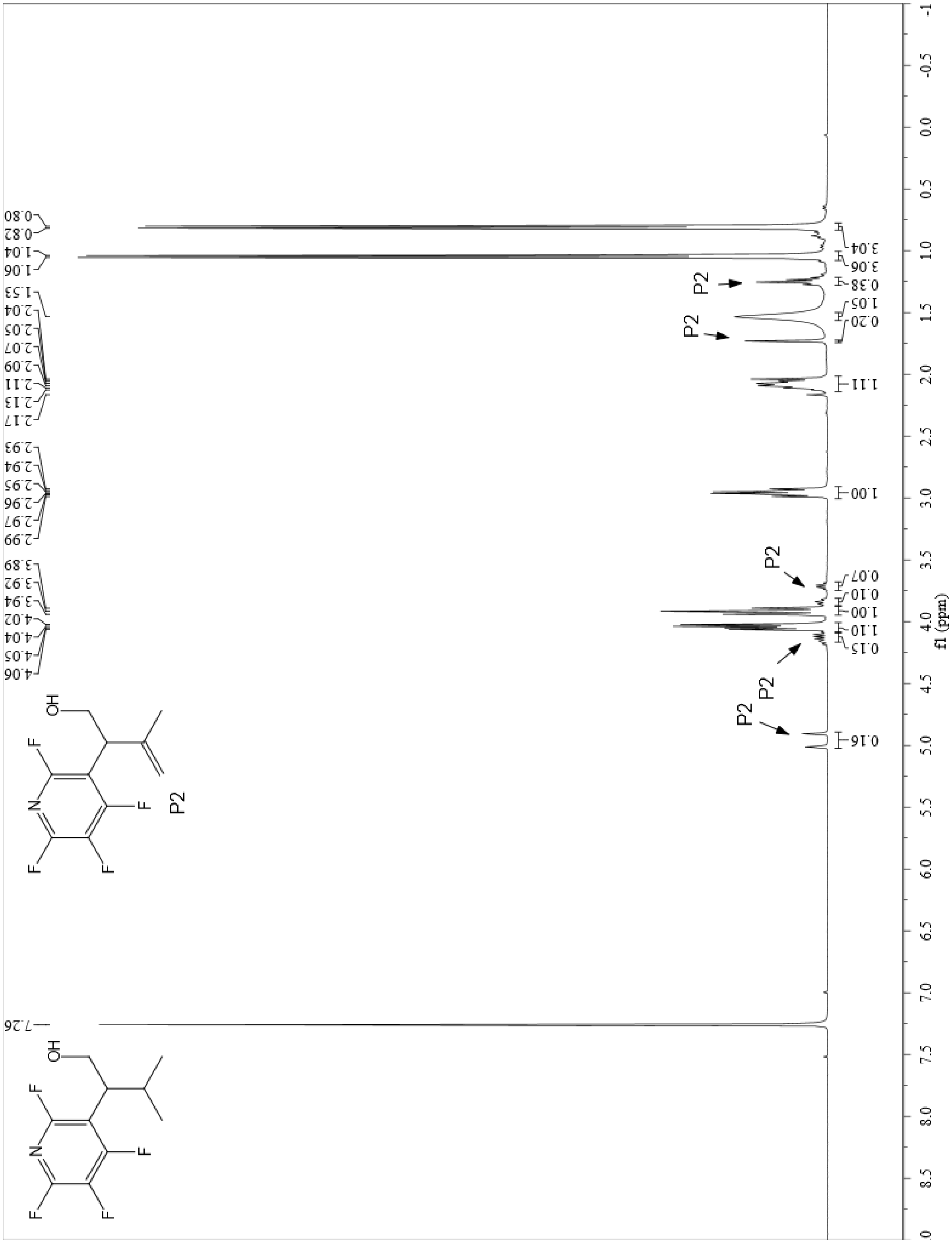

$^{13}C\{^{1}H\}$ NMR (101 MHz, Chloroform-*d*, @ rt) of S-12a 3-methyl-2-(perfluoropyridin-3-yl)butan-1-ol

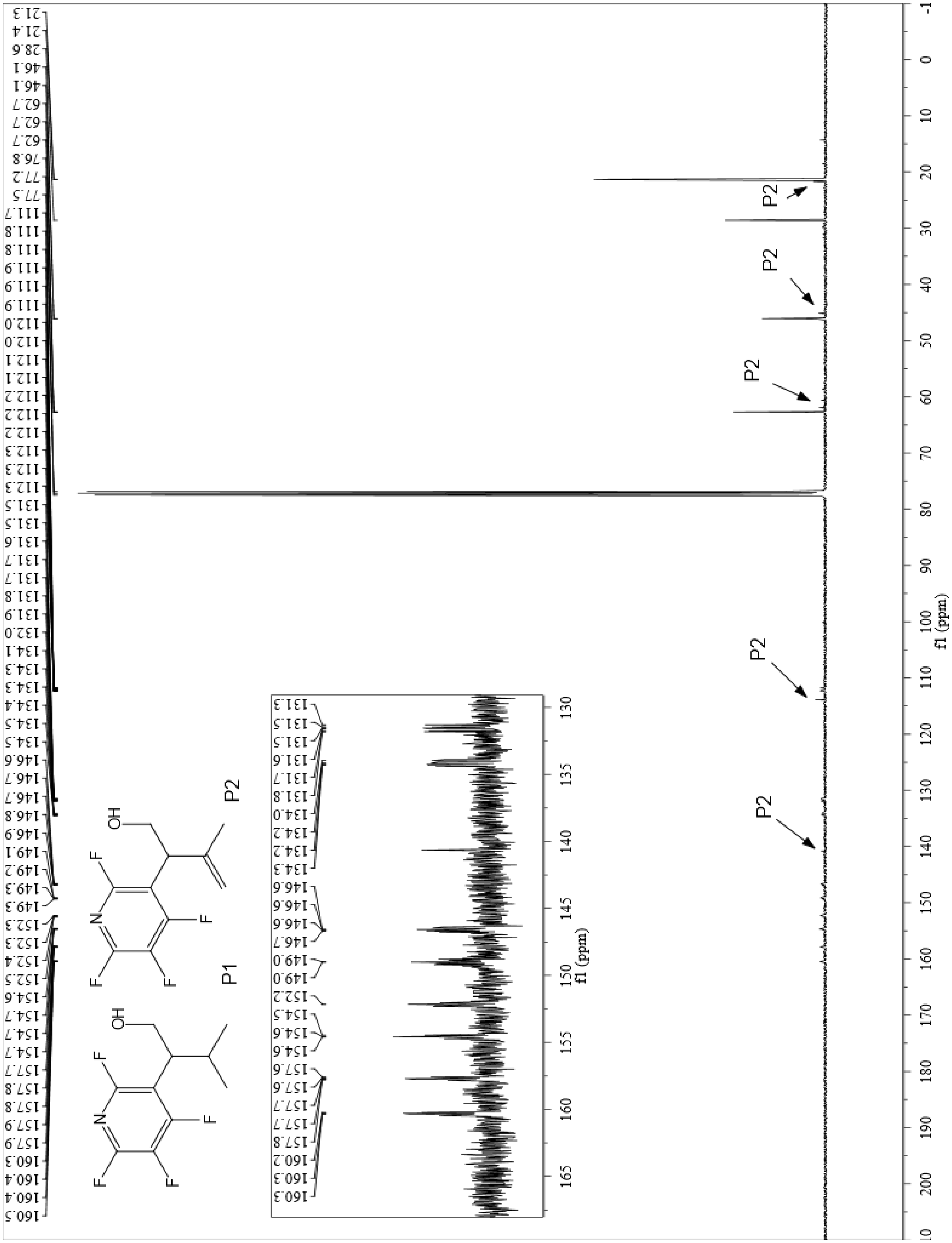

GC and MS of S-12a 3-methyl-2-(perfluoropyridin-3-yl)butan-1-ol

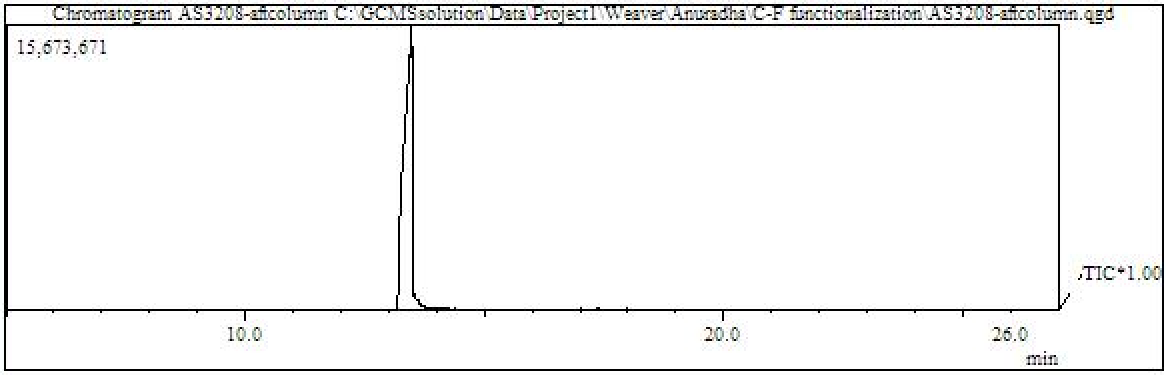

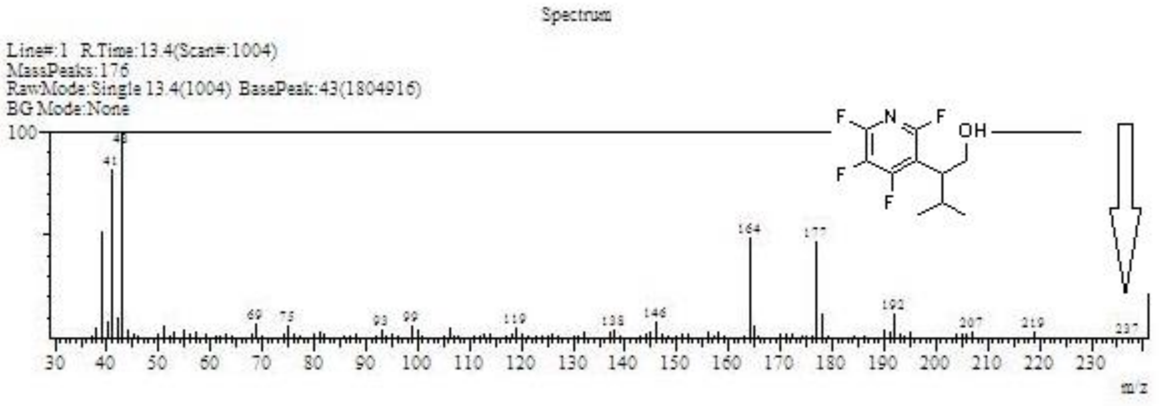

^{19}F NMR (376 MHz, Chloroform-*d*, @ rt) of S-13a 4-(6-chlorohexyl)-2,3,5,6-tetrafluoropyridine

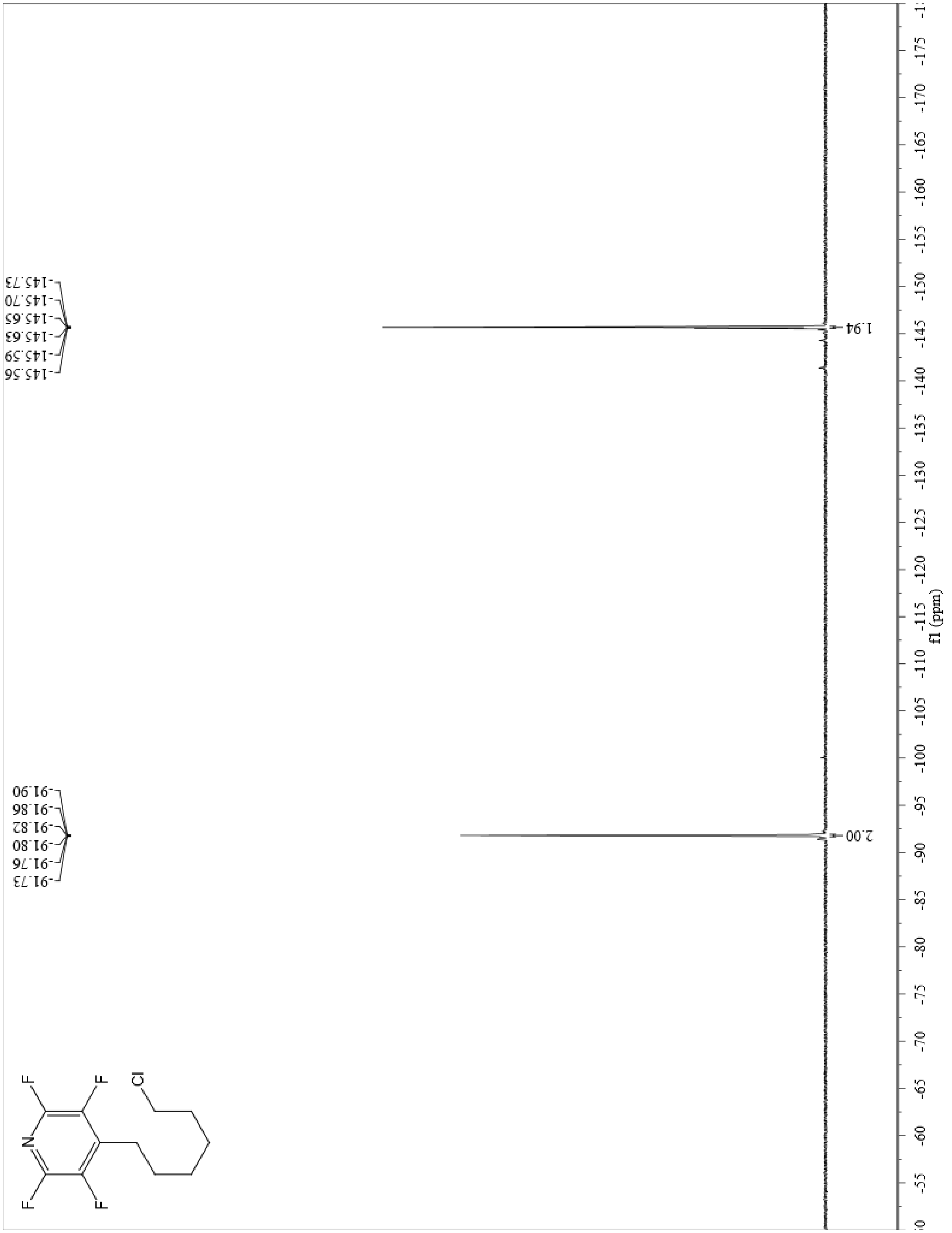

¹H NMR (400 MHz, Chloroform-*d*, @ rt) of S-13a 4-(6-chlorohexyl)-2,3,5,6-tetrafluoropyridine

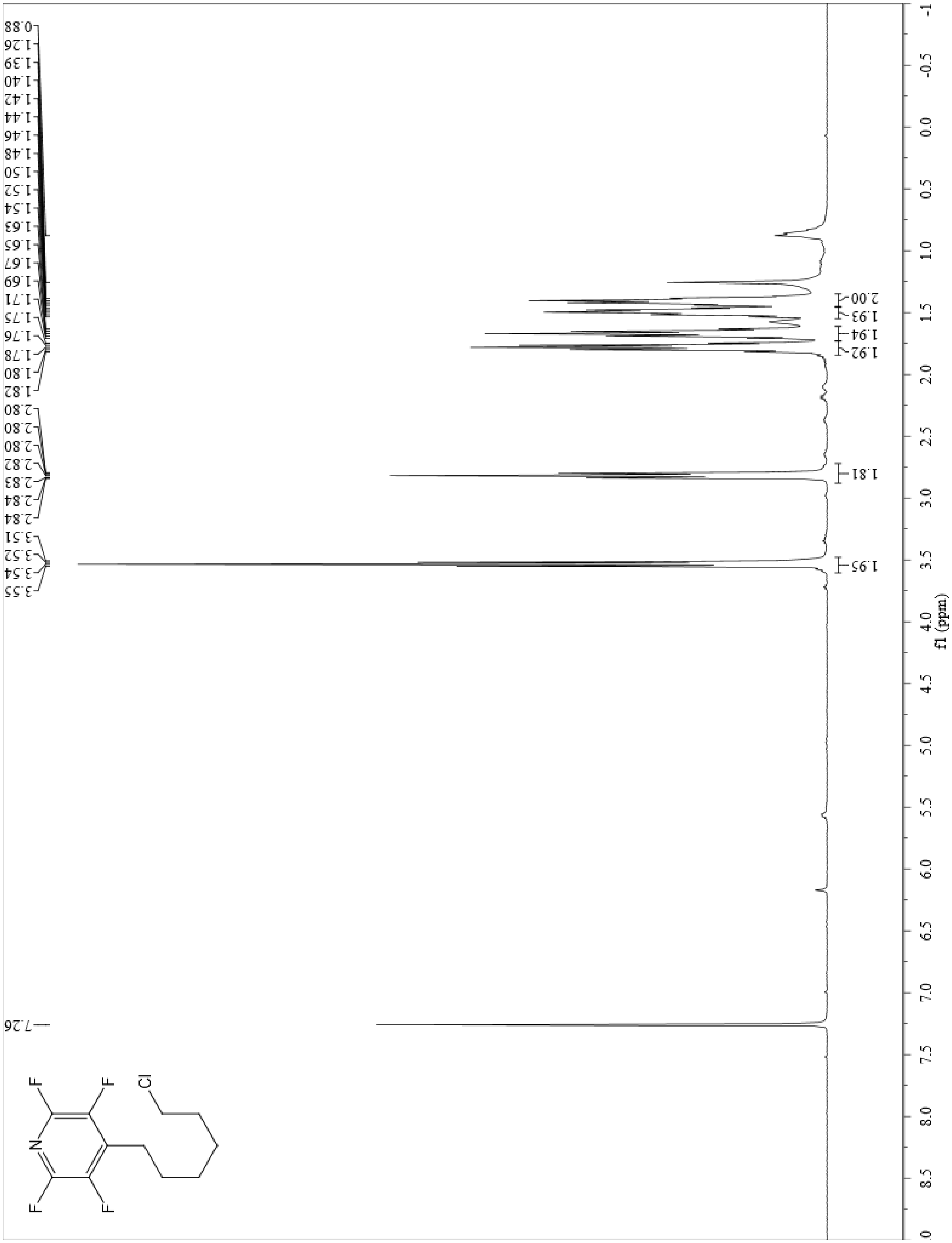

$^{13}C\{^{1}H\}$ NMR of (101 MHz, Chloroform-*d*, @ rt) S-13a 4-(6-chlorohexyl)-2,3,5,6-tetrafluoropyridine

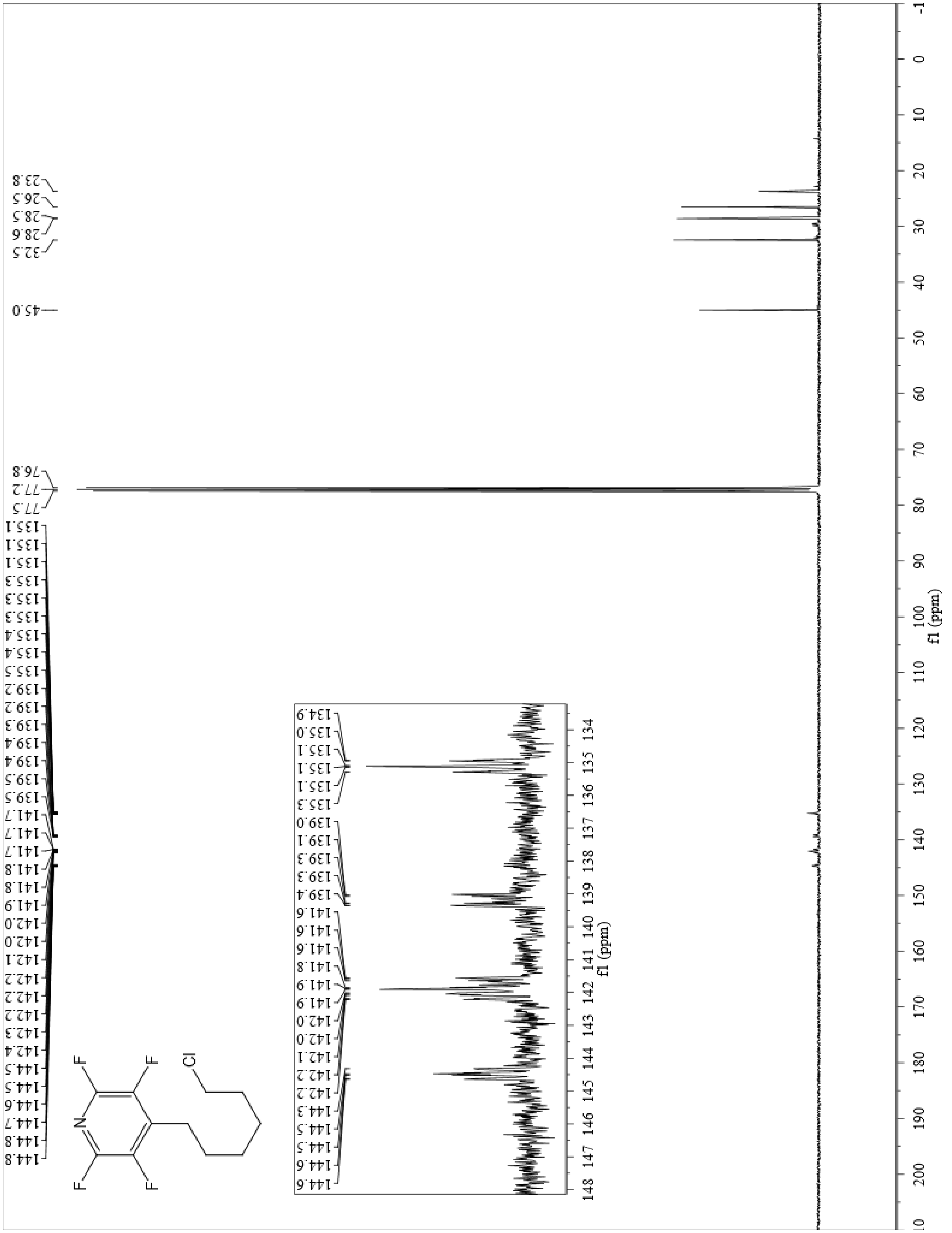

GC and MS of S-13a 4-(6-chlorohexyl)-2,3,5,6-tetrafluoropyridine

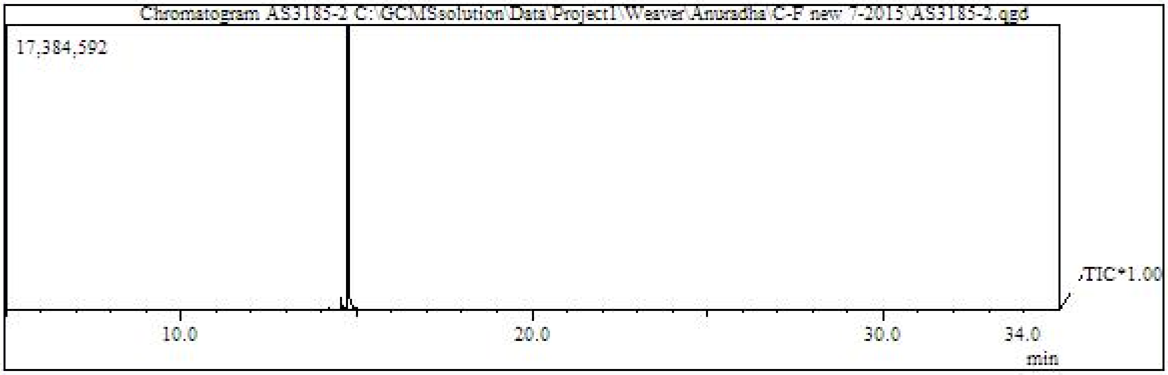

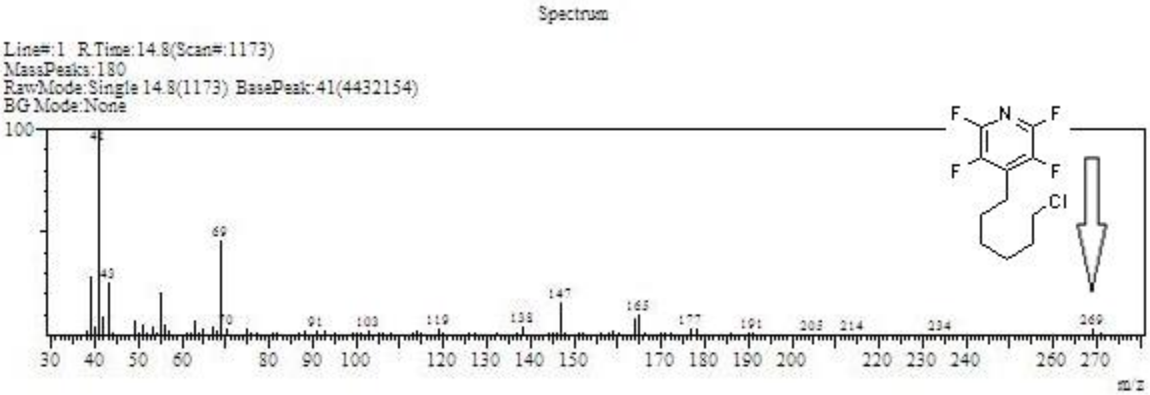

^{19}F NMR (376 MHz, Chloroform-*d*, @ rt) of S-14a 3-methyl-2-(perfluorophenyl)butan-1-ol

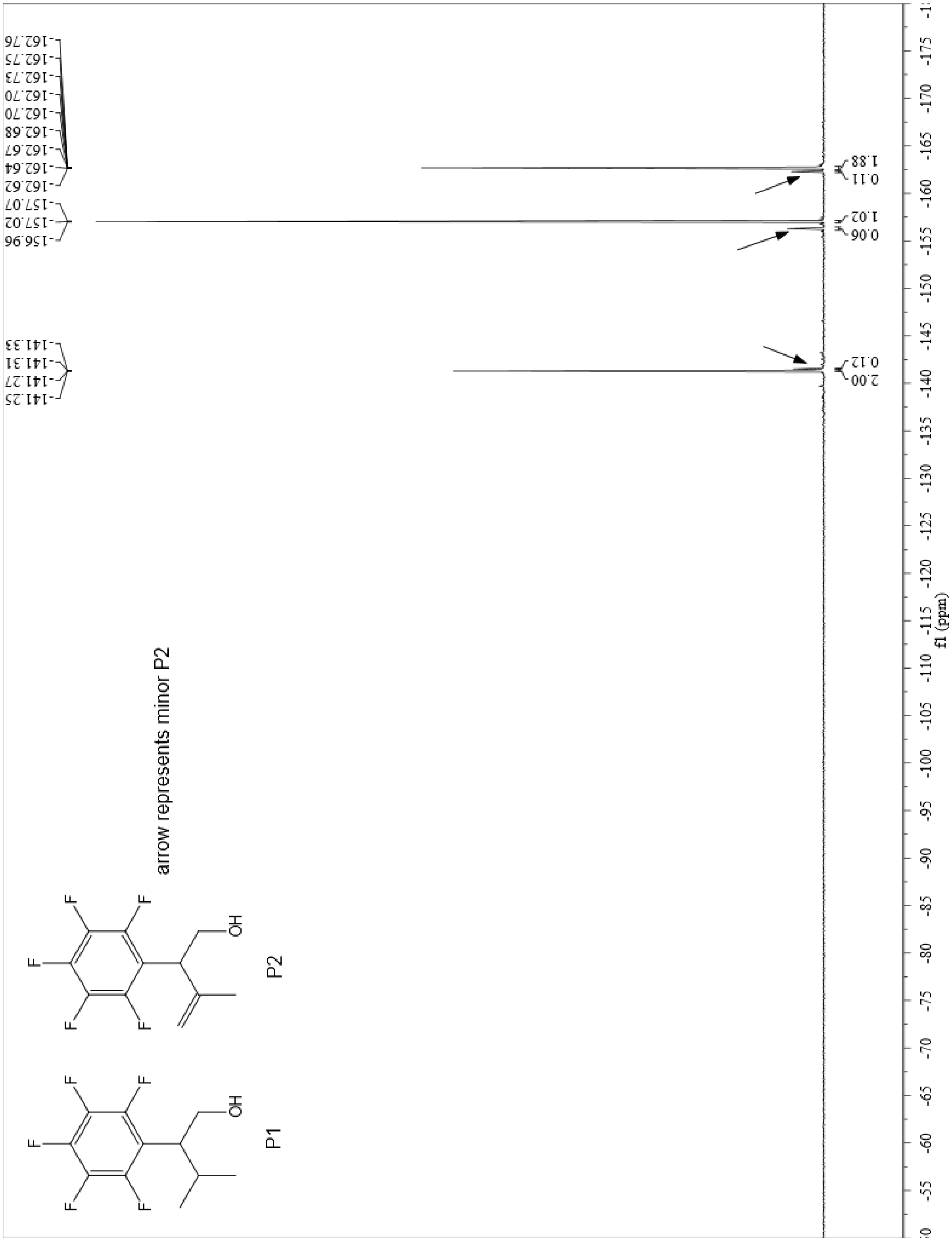

^{1}H NMR (400 MHz, Chloroform-*d*, @ rt) of S-14a 3-methyl-2-(perfluorophenyl)butan-1-ol

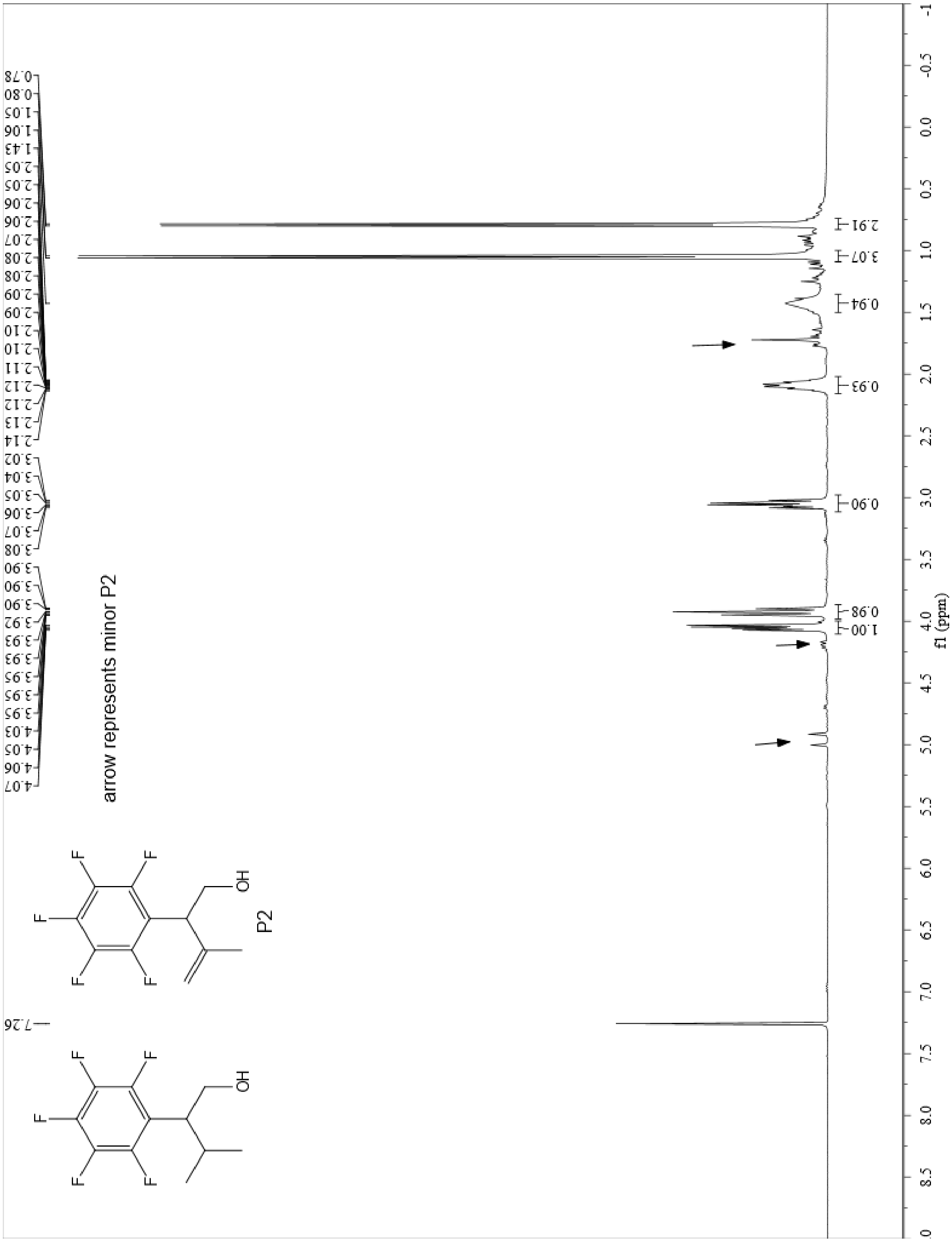

$^{13}C\{^{1}H\}$ NMR (101 MHz, Chloroform-*d*, @ rt) of S-14a 3-methyl-2-(perfluorophenyl)butan-1-ol

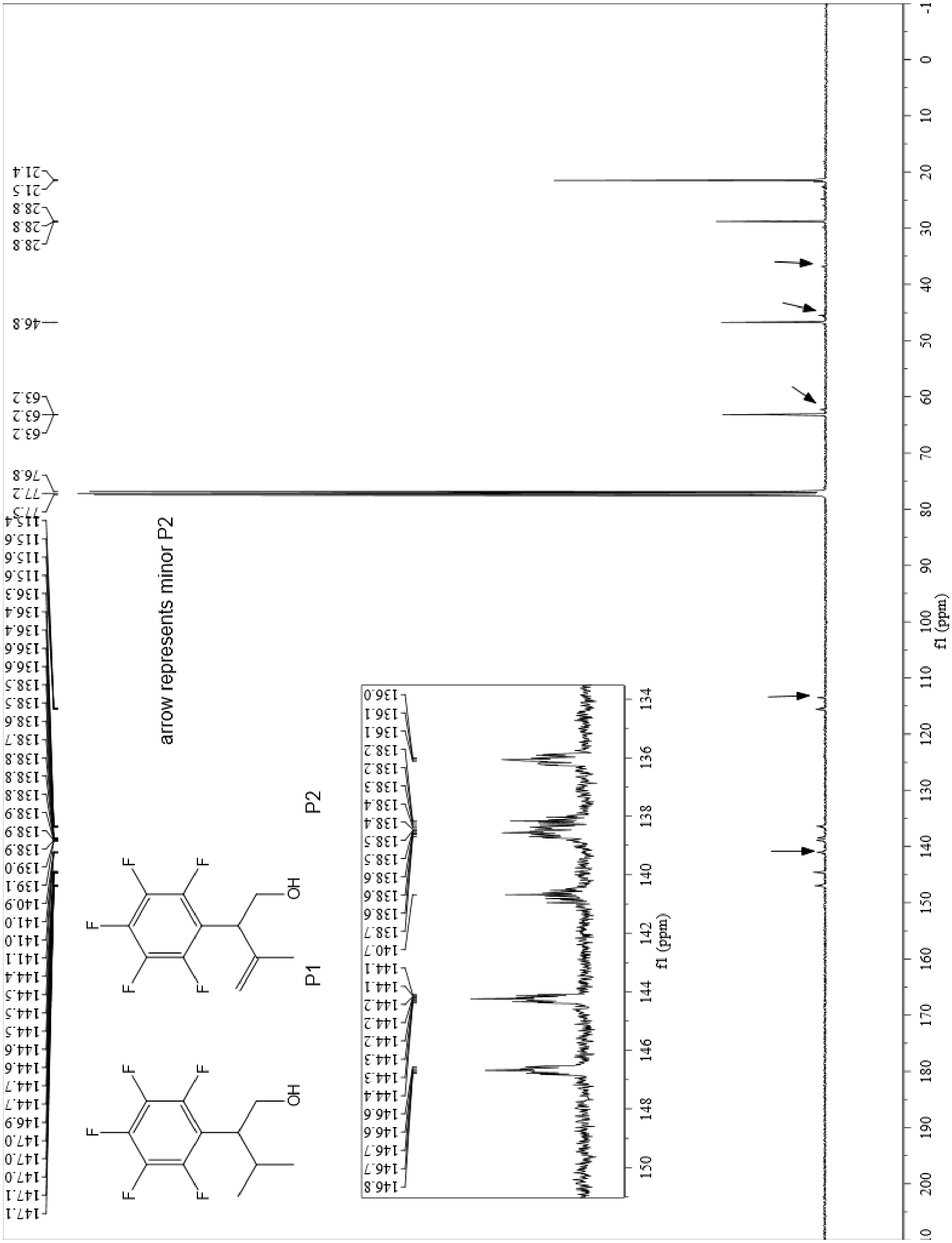

GC and MS of S-14a 3-methyl-2-(perfluorophenyl)butan-1-ol

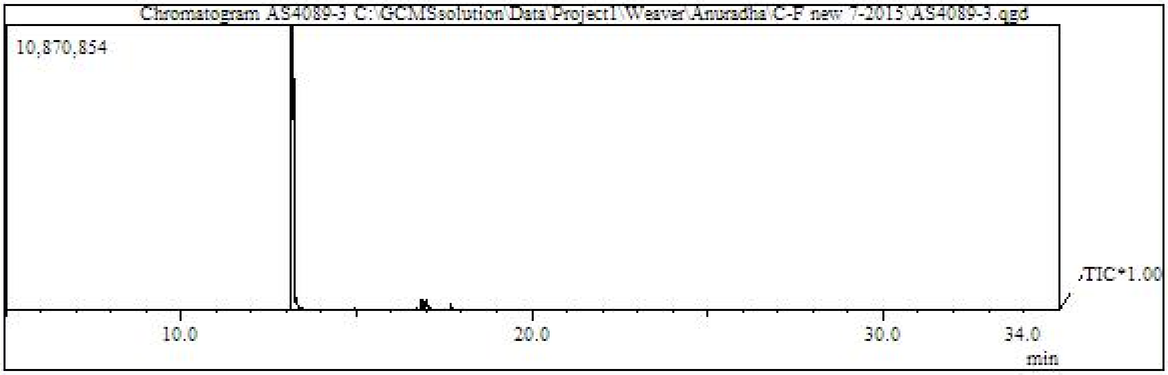

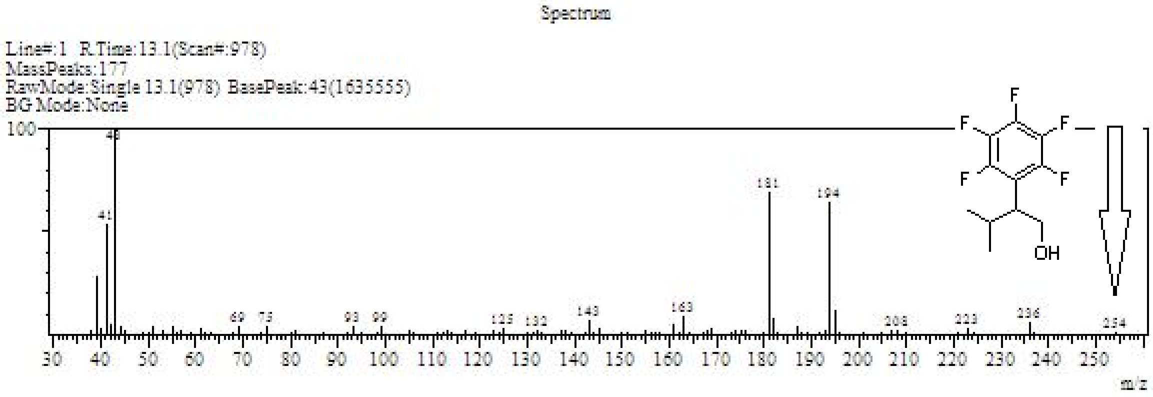

^{19}F NMR (376 MHz, Chloroform-*d*, @ rt) of S-15a 4-(bicyclo[2.2.1]heptan-2-yl)-2,3,5,6-tetrafluoropyridine

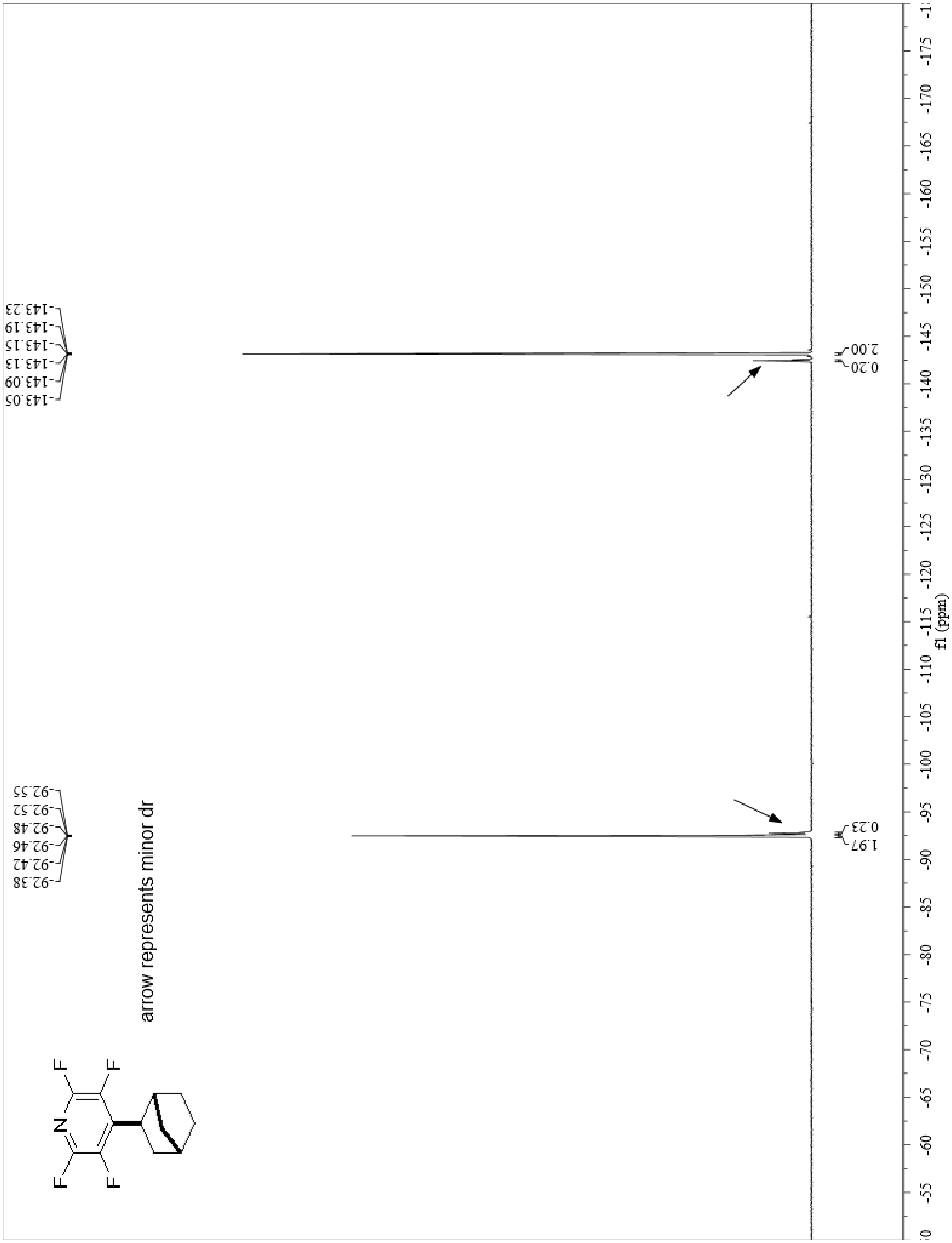

^{1}H NMR (400 MHz, Chloroform-*d*, @ rt) of S-15a 4-(bicyclo[2.2.1]heptan-2-yl)-2,3,5,6-tetrafluoropyridine

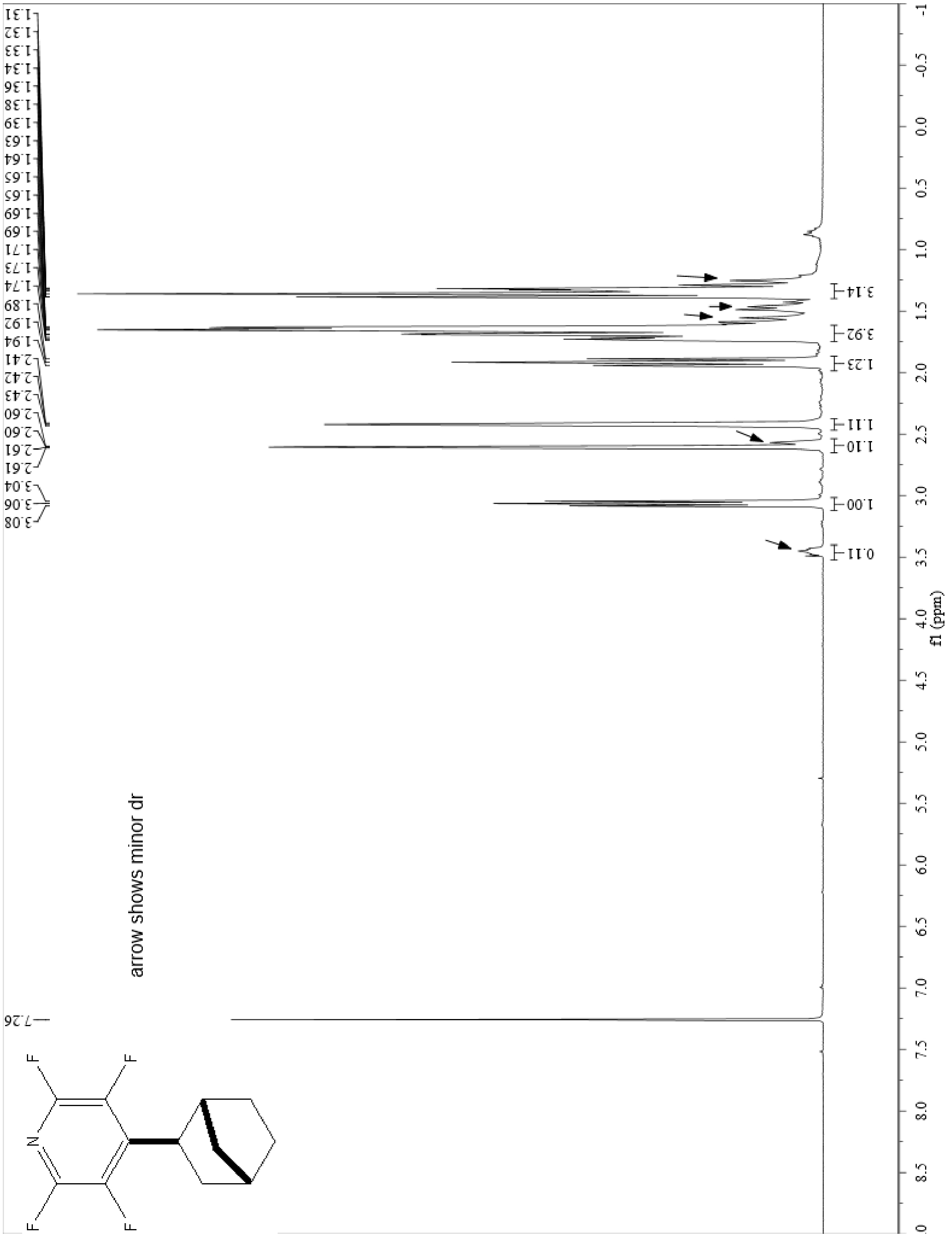

$^{13}C\{^{1}H\}$ NMR (101 MHz, Chloroform-*d*, @ rt) of S-15a 4-(bicyclo[2.2.1]heptan-2-yl)-2,3,5,6-tetrafluoropyridine

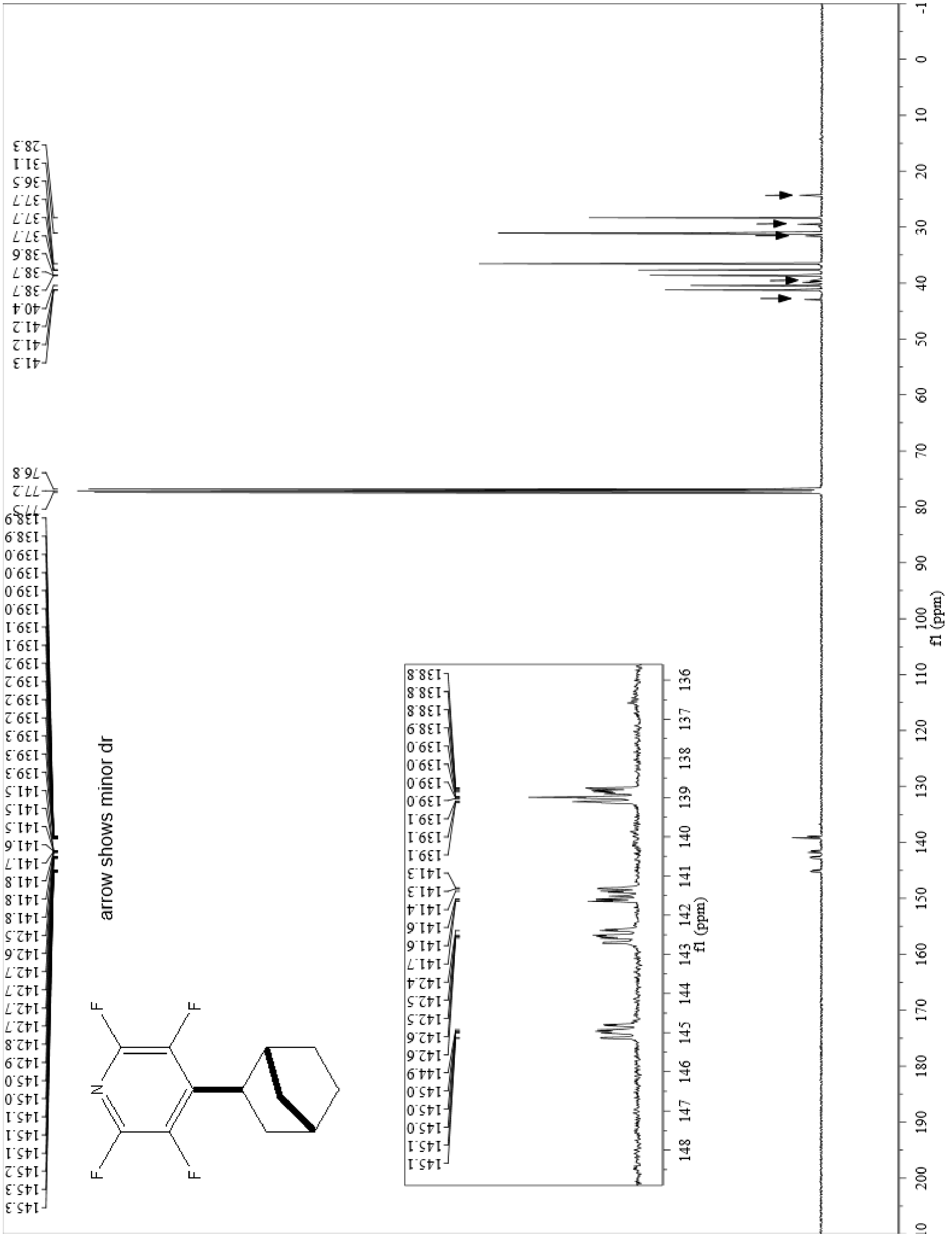

GC and MS of S-15a 4-(bicyclo[2.2.1]heptan-2-yl)-2,3,5,6-tetrafluoropyridine

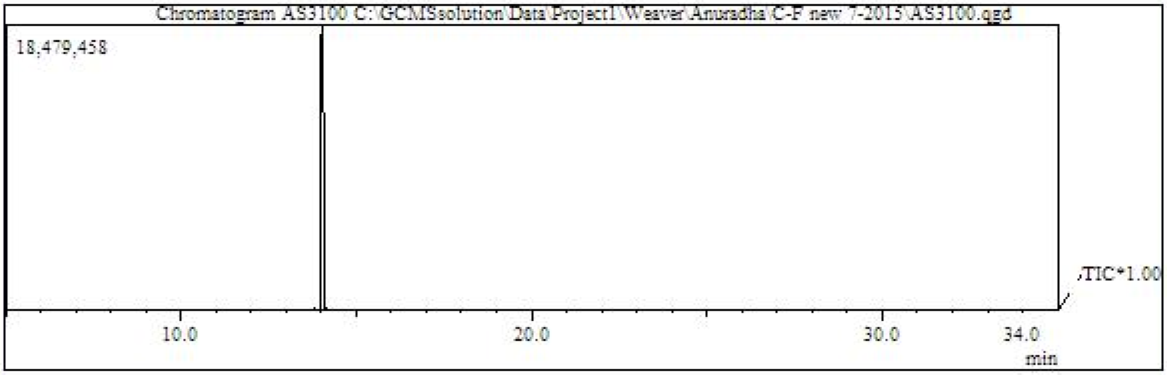

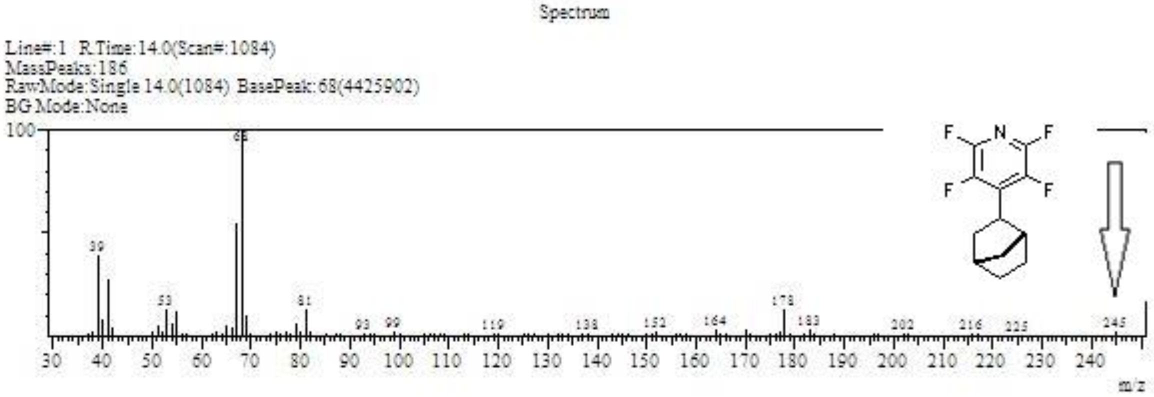

^{19}F NMR (376 MHz, Chloroform-*d*, @ rt) of S-16a dimethyl 5-(perfluoropyridin-4-yl)-7-oxabicyclo[2.2.1]heptane-2,3-dicarboxylate

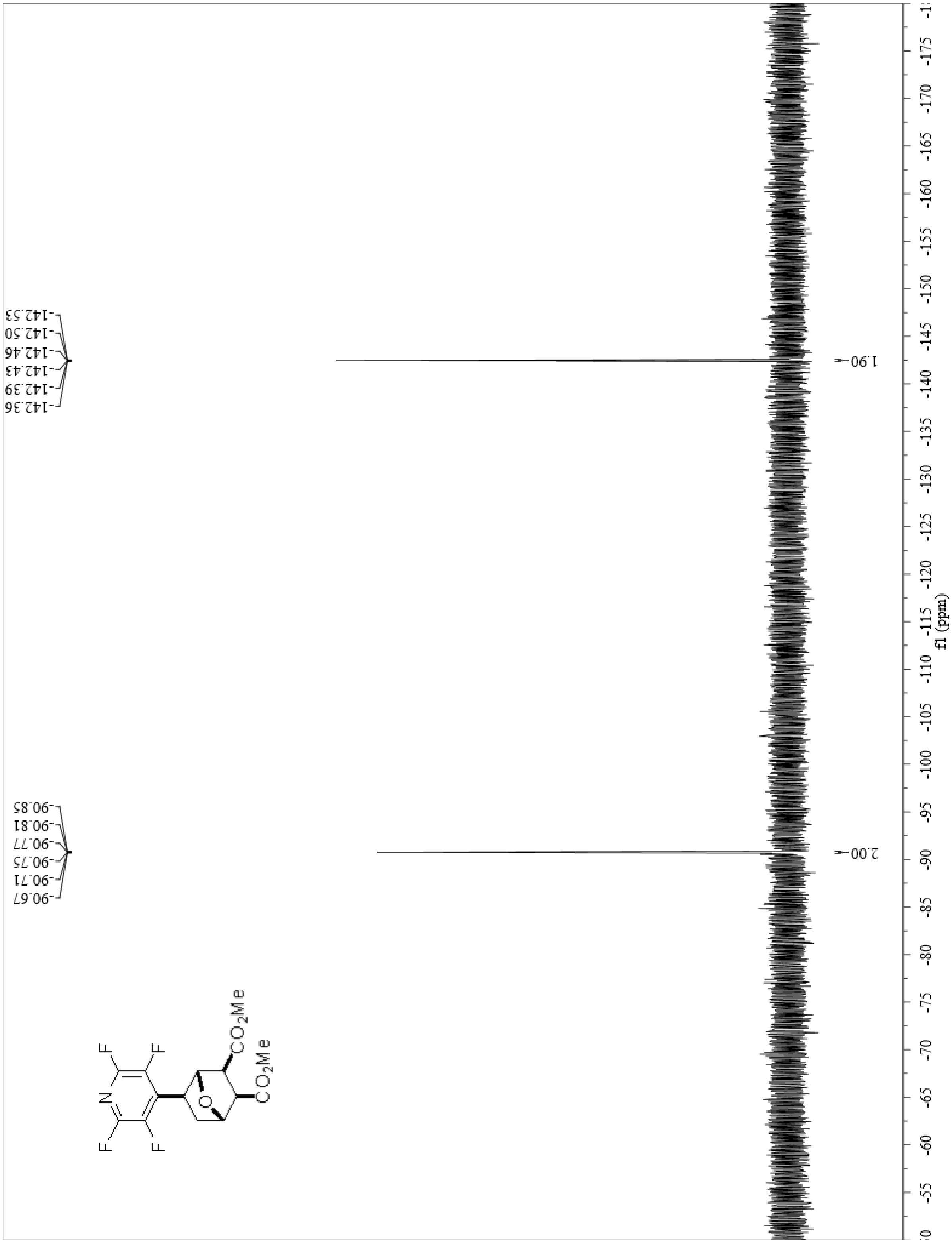

^{1}H NMR (400 MHz, Chloroform-*d*, @ rt) of S-16a dimethyl 5-(perfluoropyridin-4-yl)-7-oxabicyclo[2.2.1]heptane-2,3-dicarboxylate

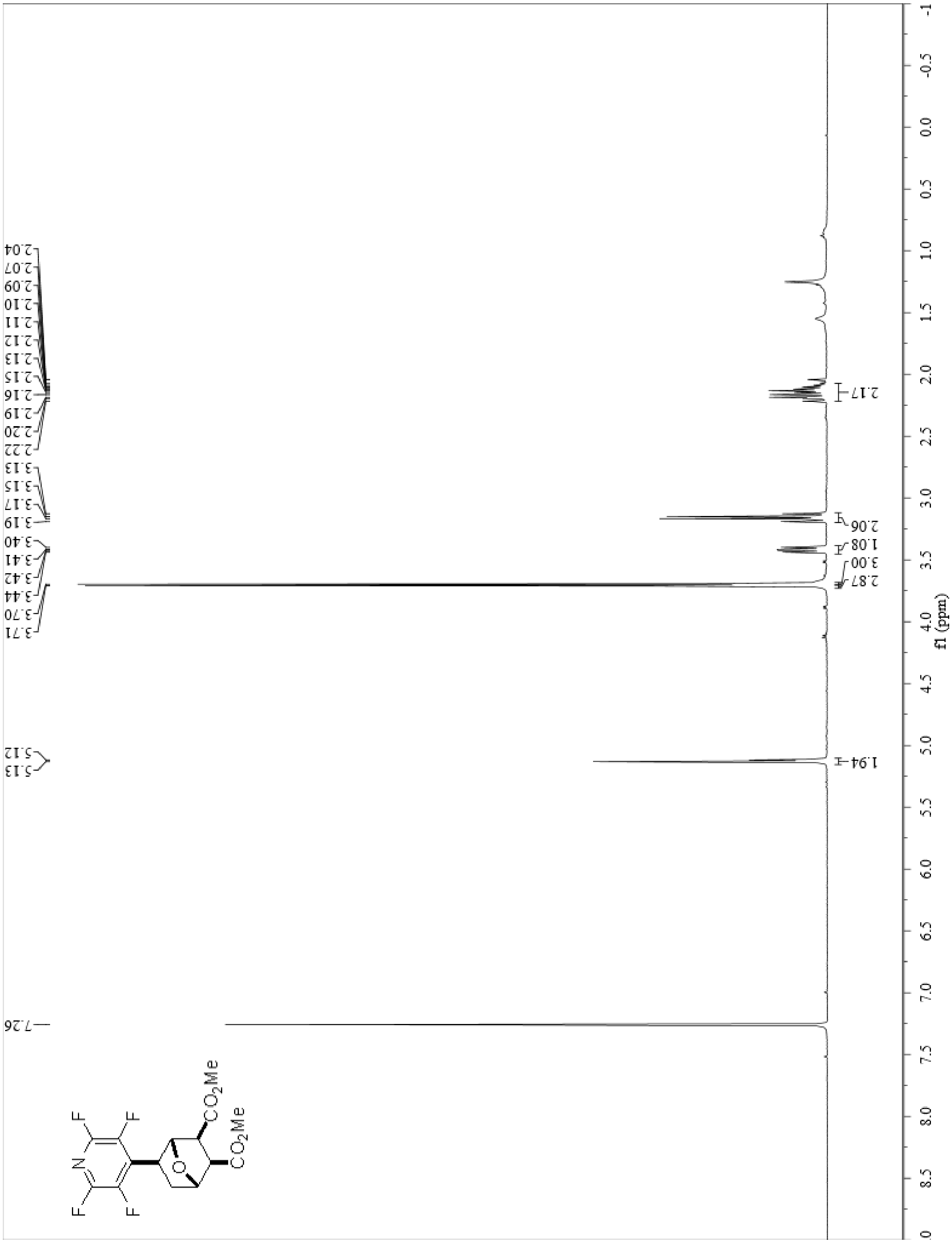

$^{13}C\{^1H\}$ NMR (101 MHz, Chloroform-*d*, @ rt) of S-16a dimethyl 5-(perfluoropyridin-4-yl)-7-oxabicyclo[2.2.1]heptane-2,3-dicarboxylate

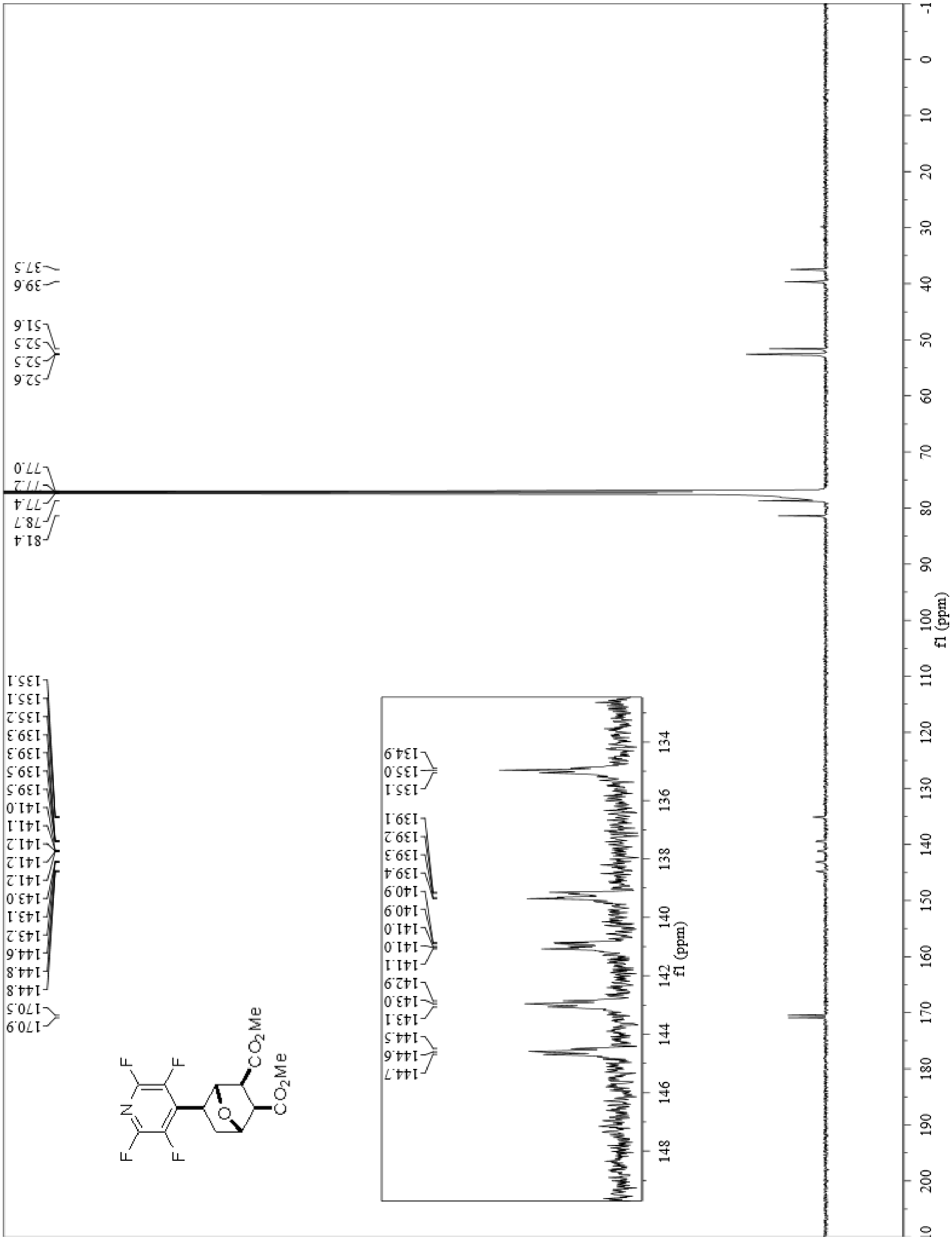

GC and MS of S-16a dimethyl 5-(perfluoropyridin-4-yl)-7-oxabicyclo[2.2.1]heptane-2,3-dicarboxylate

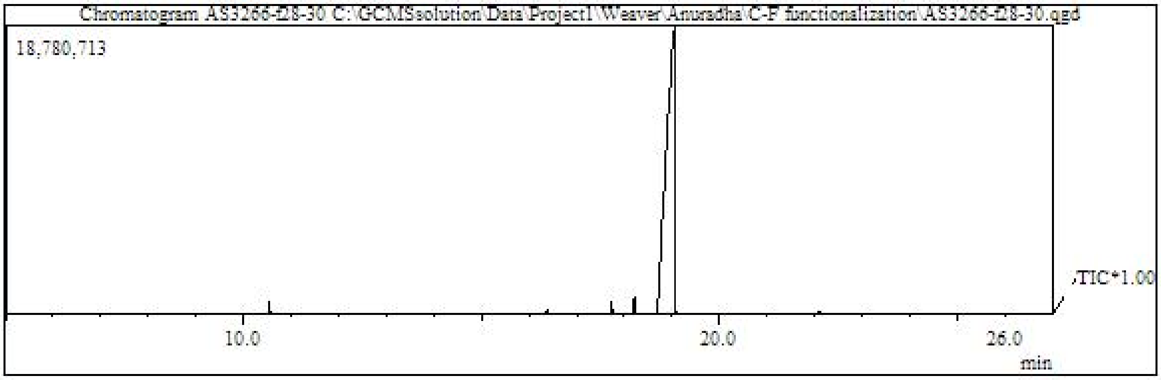

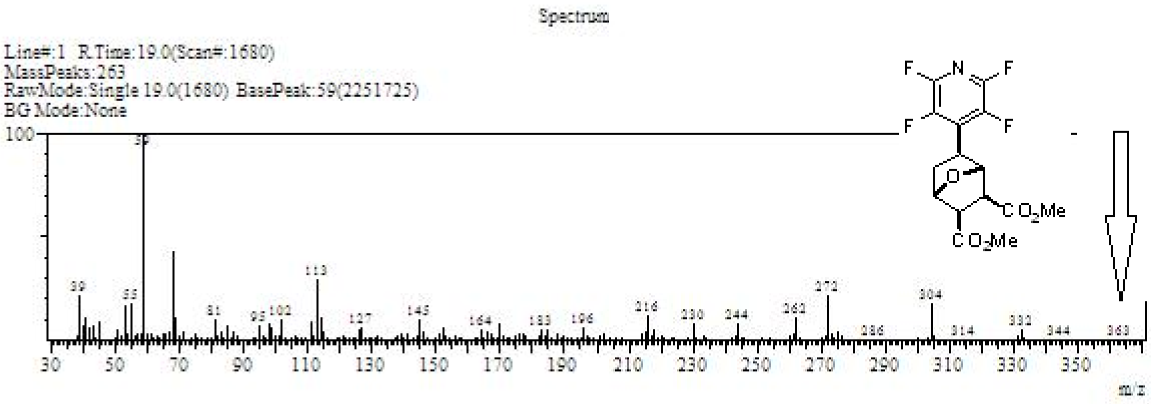

^{19}F NMR (376 MHz, Chloroform-*d*, @ rt) of S-17a 4-cyclohexyl-2,2',3,3',5,5',6,6'-octafluoro-1,1'-biphenyl

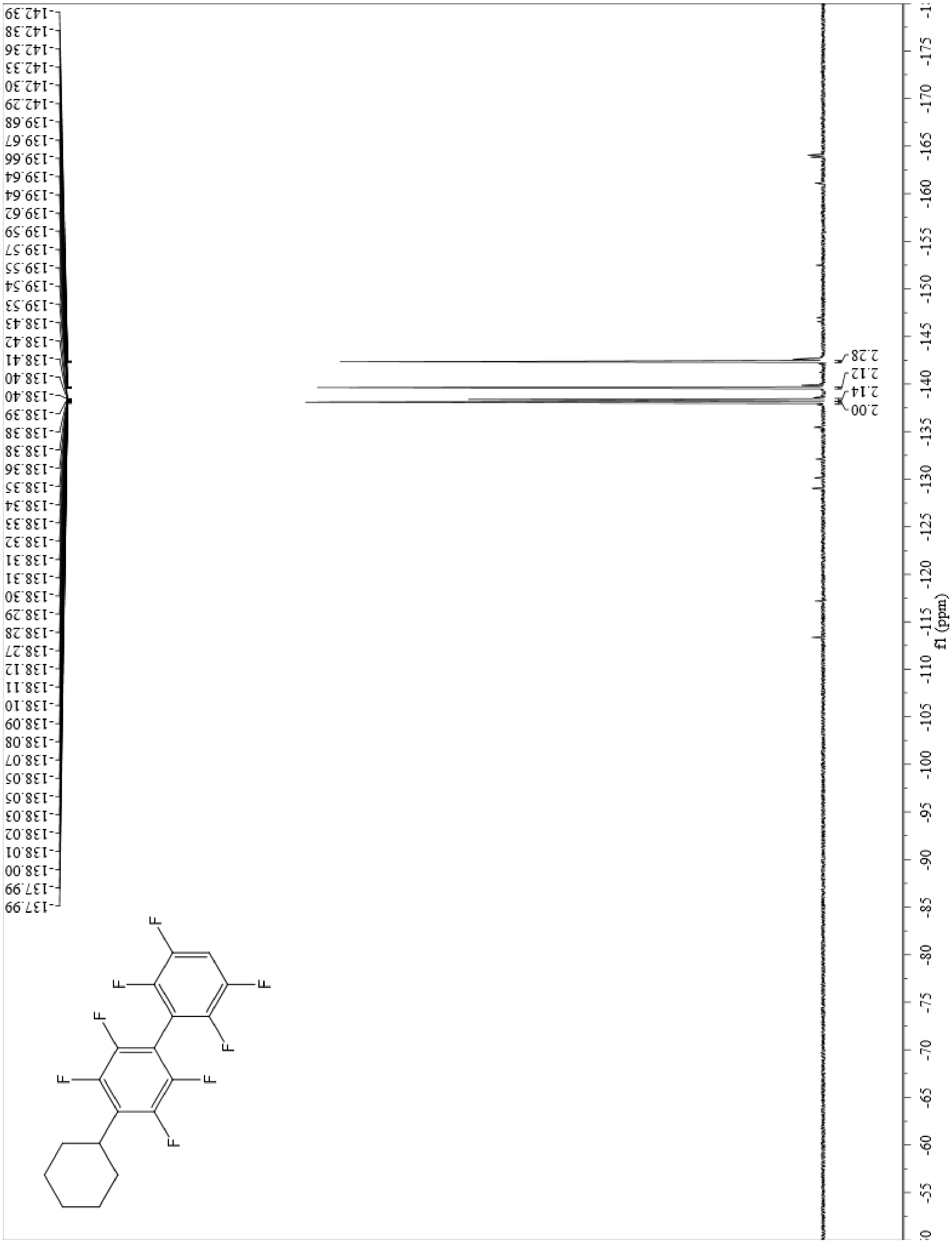

^{1}H NMR (400 MHz, Chloroform-*d*, @ rt) of S-17a 4-cyclohexyl-2,2',3,3',5,5',6,6'-octafluoro-1,1'-biphenyl

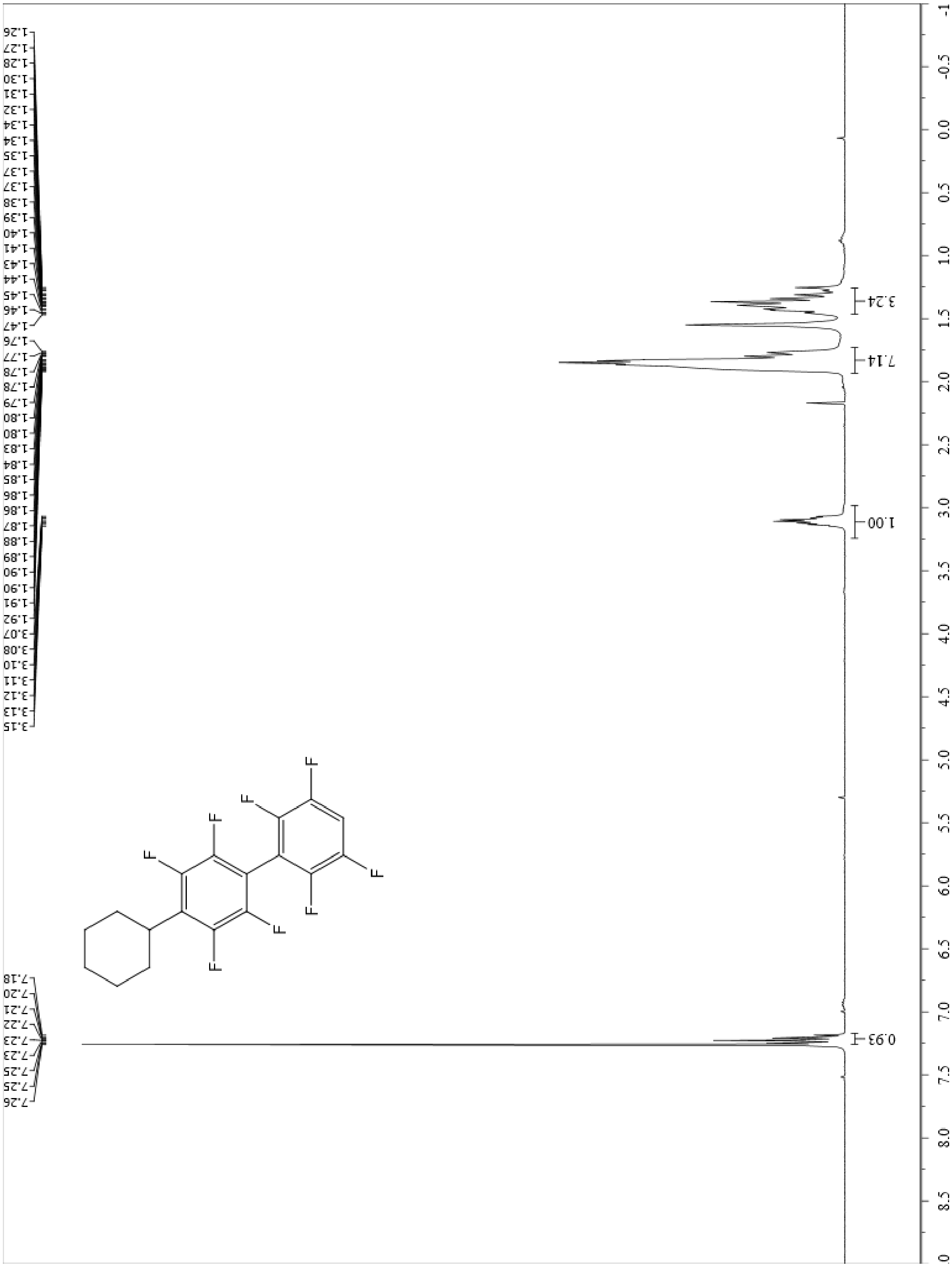

$^{13}C\{^{1}H\}$ NMR (101 MHz, Chloroform-*d*, @ rt) of S-17a 4-cyclohexyl-2,2',3,3',5,5',6,6'-octafluoro-1,1'-biphenyl

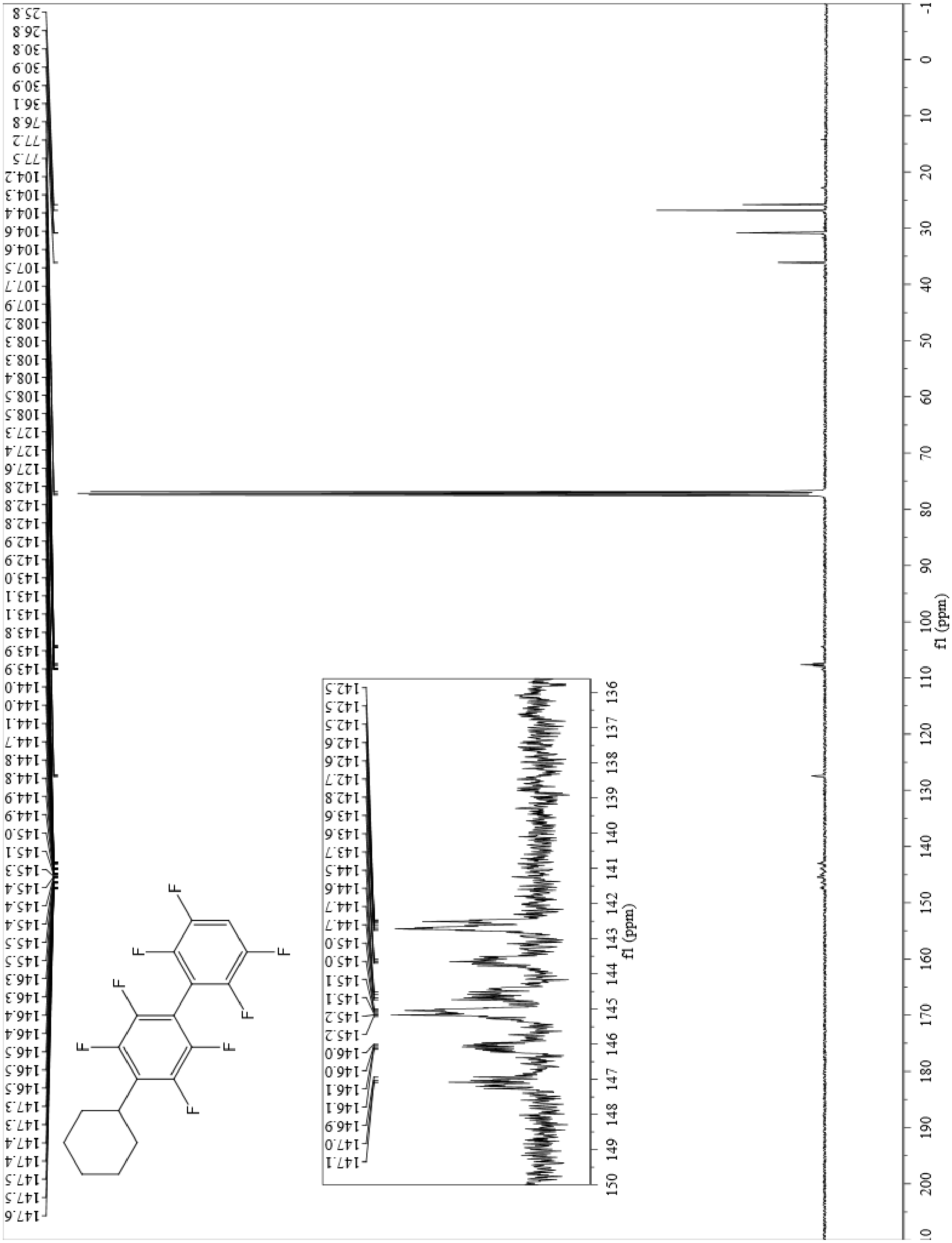

GC and MS of S-17a 4-cyclohexyl-2,2',3,3',5,5',6,6'-octafluoro-1,1'-biphenyl

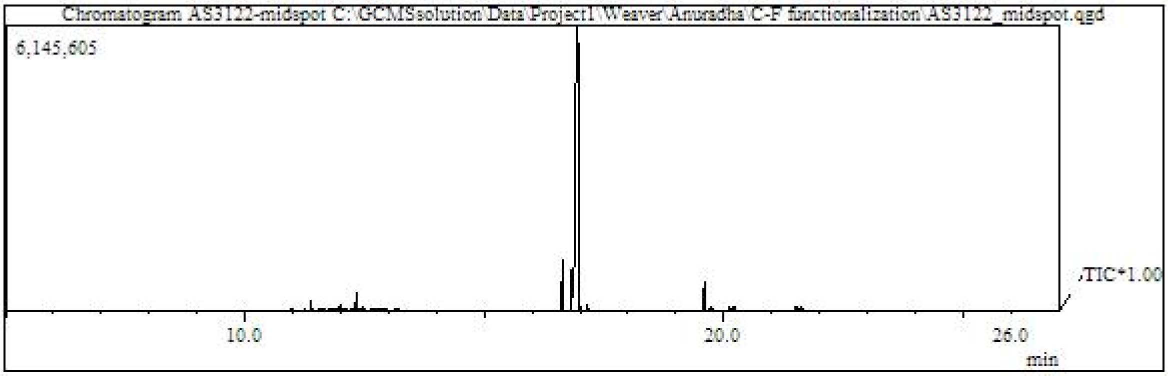

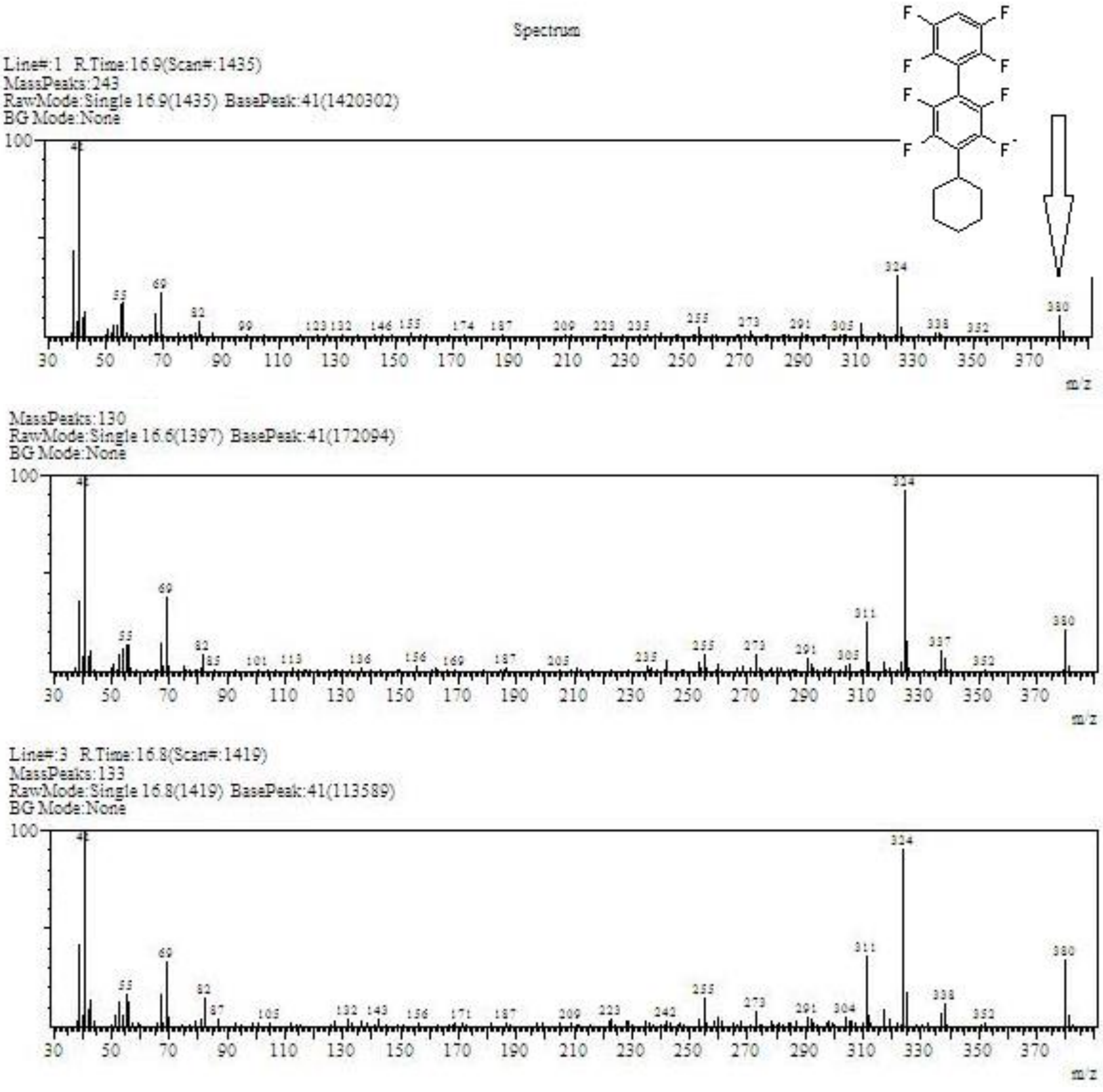

^{19}F NMR (376 MHz, Chloroform-*d*, @ rt) of S-18a ethyl 3-(2,3,5,6-tetrafluoro-4-(trifluoromethyl)phenyl)butanoate

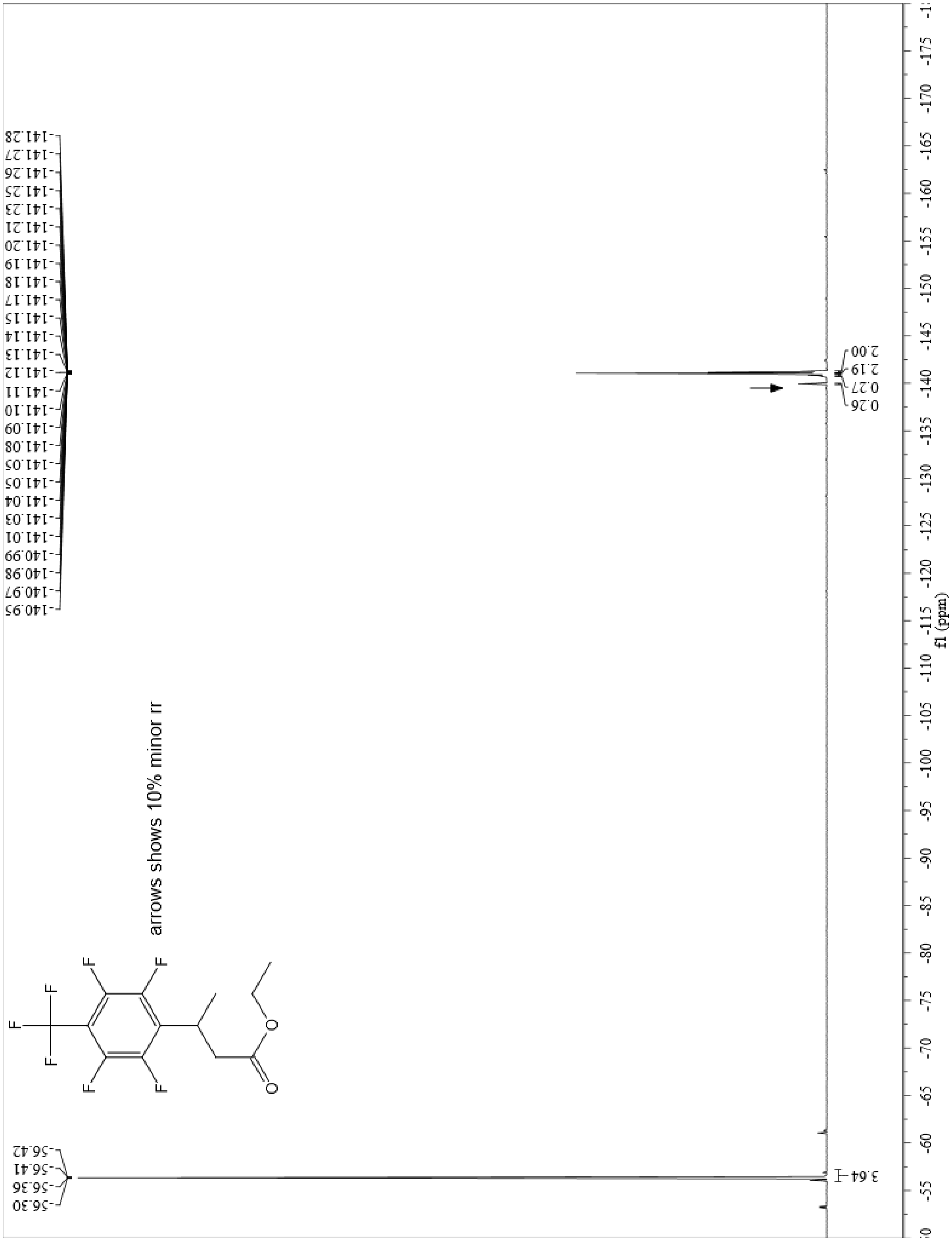

¹H NMR (400 MHz, Chloroform-*d*, @ rt) of S-18a ethyl 3-(2,3,5,6-tetrafluoro-4-(trifluoromethyl)phenyl)butanoate

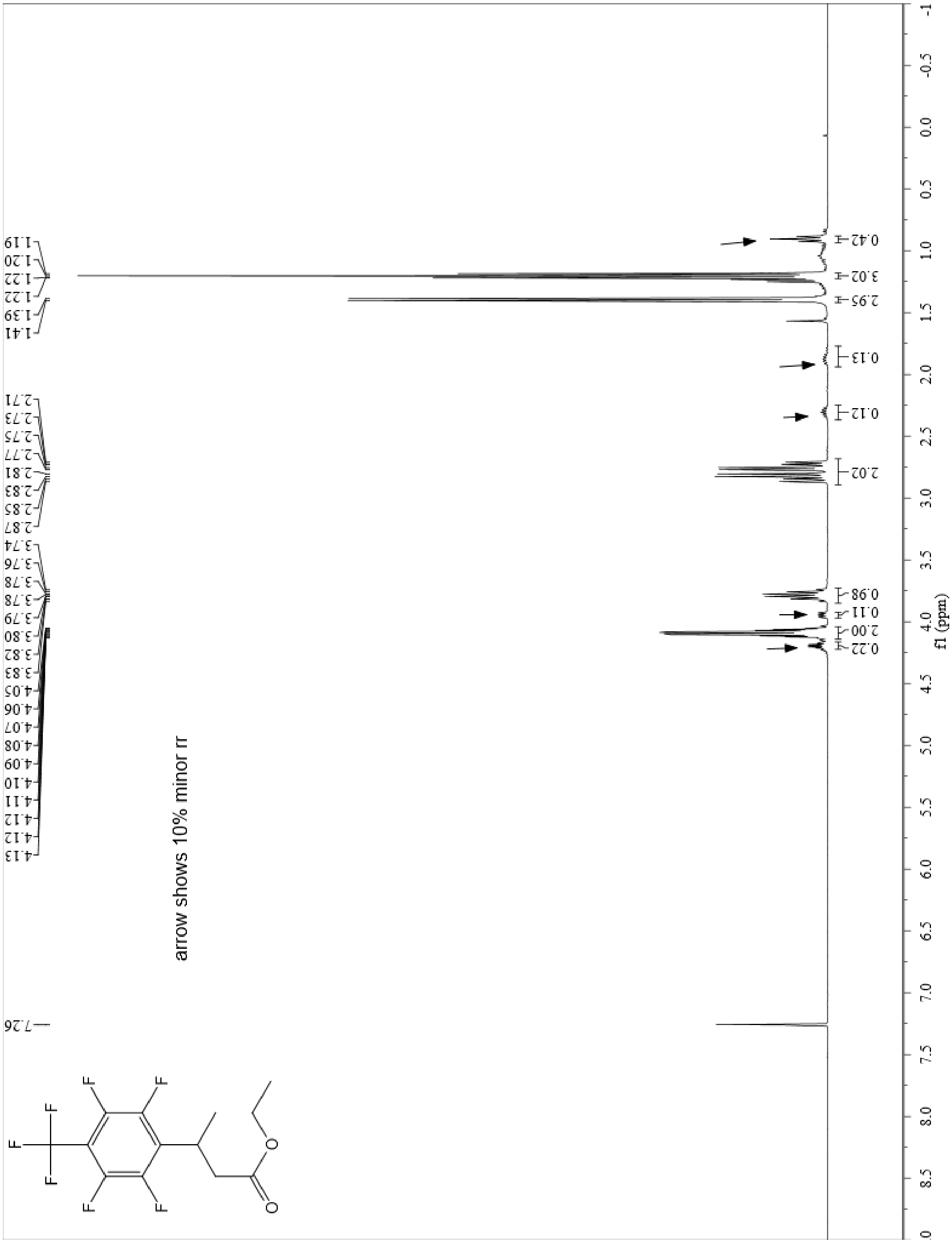

$^{13}C\{^{1}H\}$ NMR (101 MHz, Chloroform-*d*, @ rt) of S-18a ethyl 3-(2,3,5,6-tetrafluoro-4-(trifluoromethyl)phenyl)butanoate

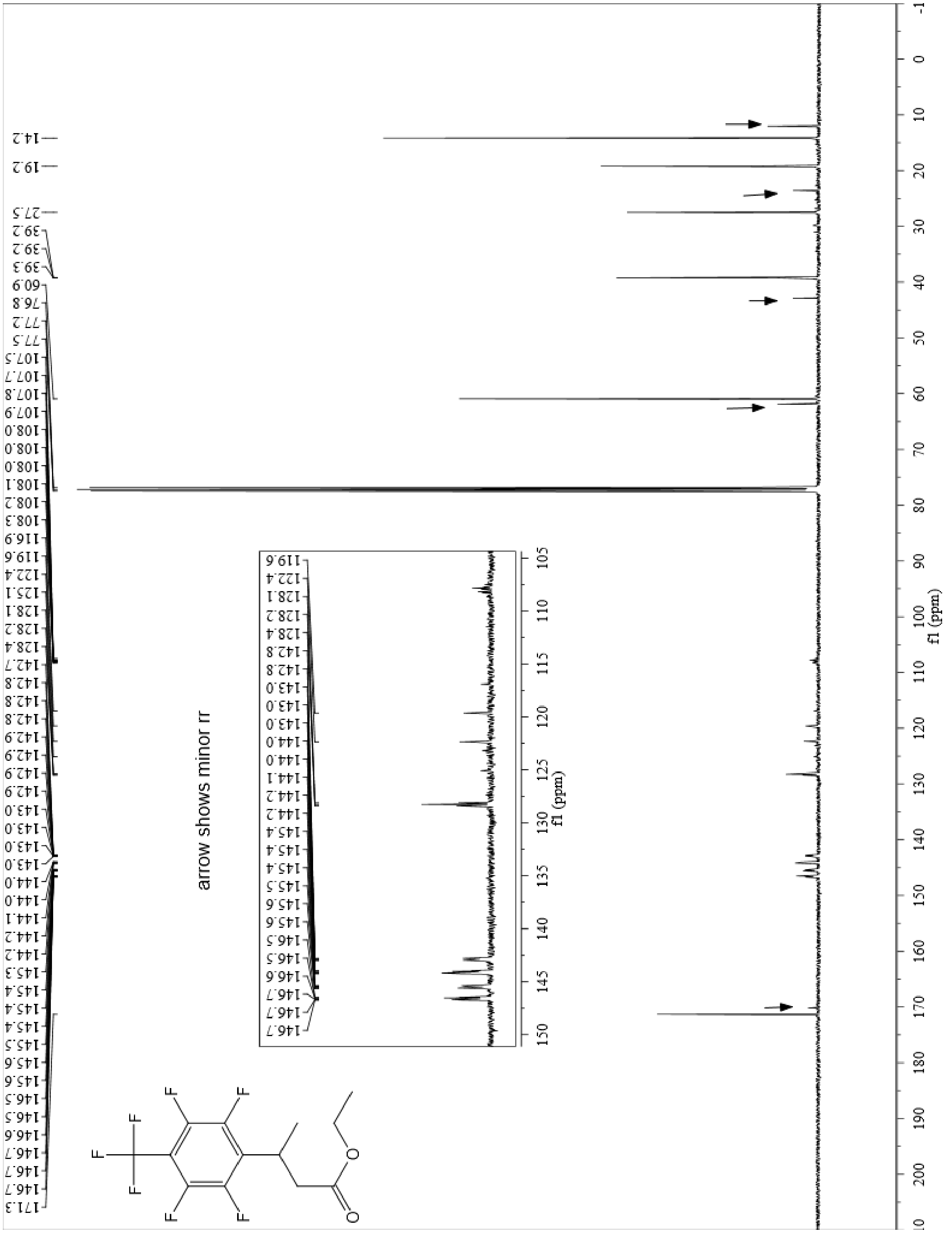

GC and MS of S-18a ethyl 3-(2,3,5,6-tetrafluoro-4-(trifluoromethyl)phenyl)butanoate

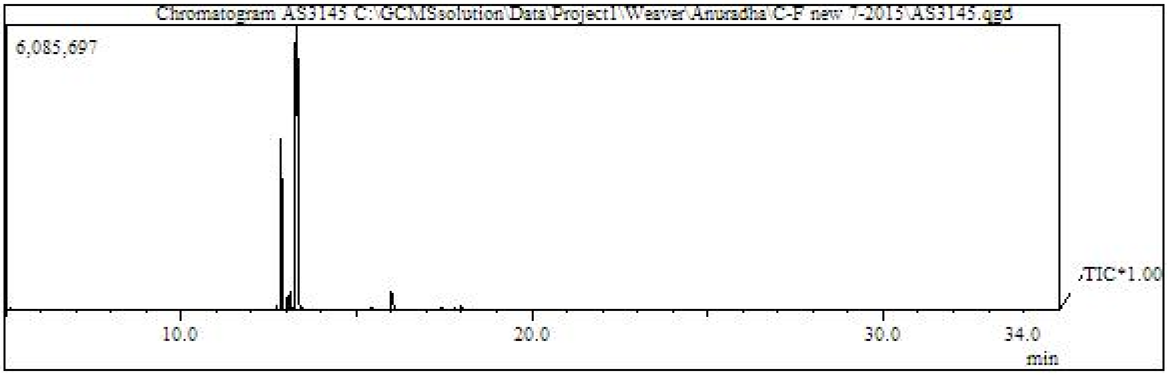

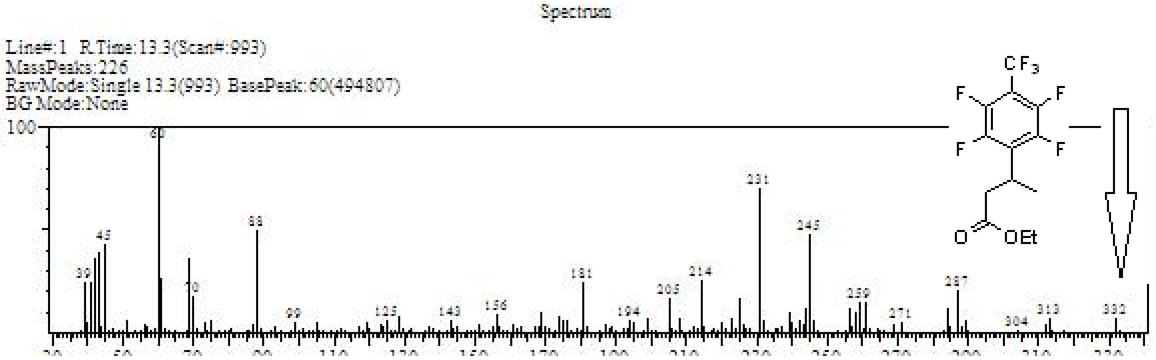

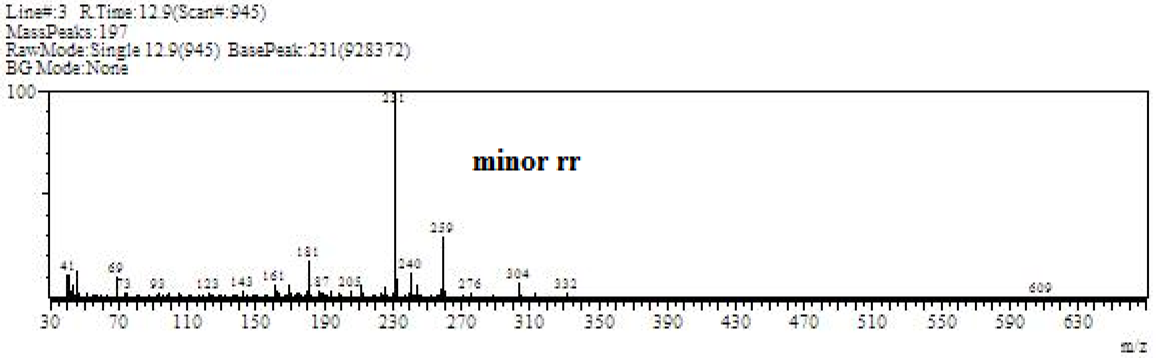

^{19}F NMR (376 MHz, Chloroform-*d*, @ rt) of S-19a methyl 3-(4-cyano-2,3,5,6-tetrafluorophenyl)butanoate

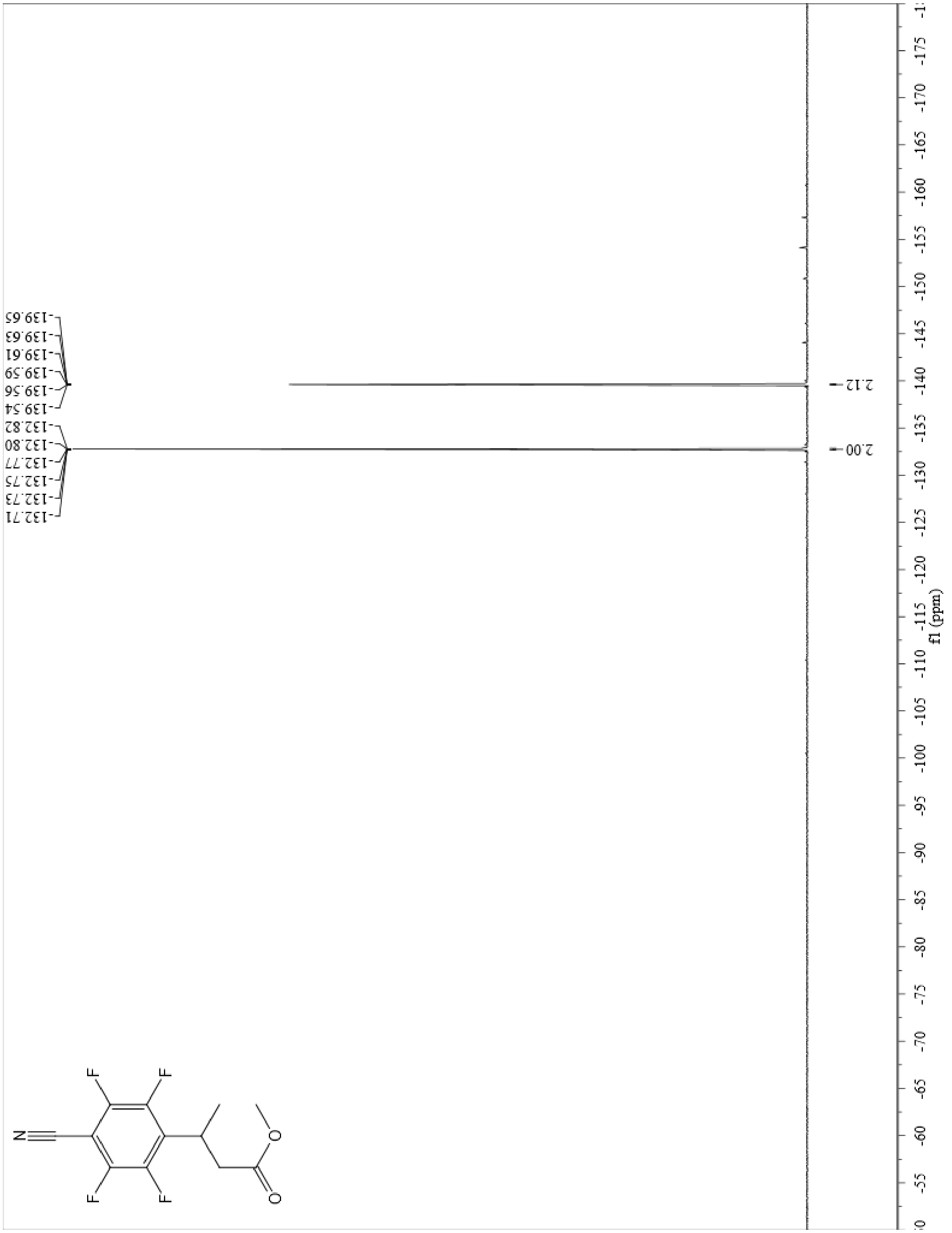

^{1}H NMR (400 MHz, Chloroform-*d*, @ rt) of S-19a methyl 3-(4-cyano-2,3,5,6-tetrafluorophenyl)butanoate

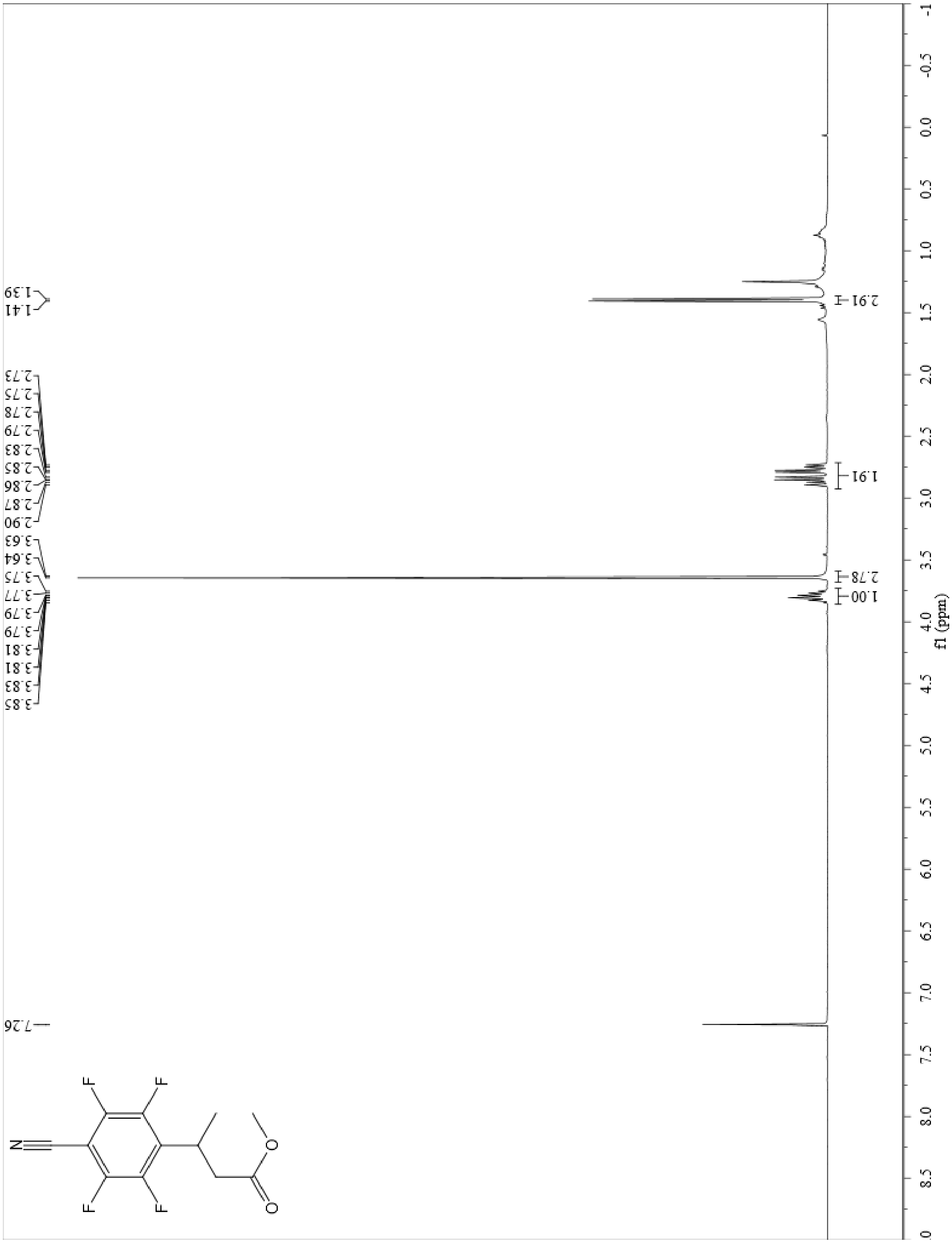

$^{13}C\{^1H\}$ NMR (101 MHz, Chloroform-*d*, @ rt) of S-19a methyl 3-(4-cyano-2,3,5,6-tetrafluorophenyl)butanoate

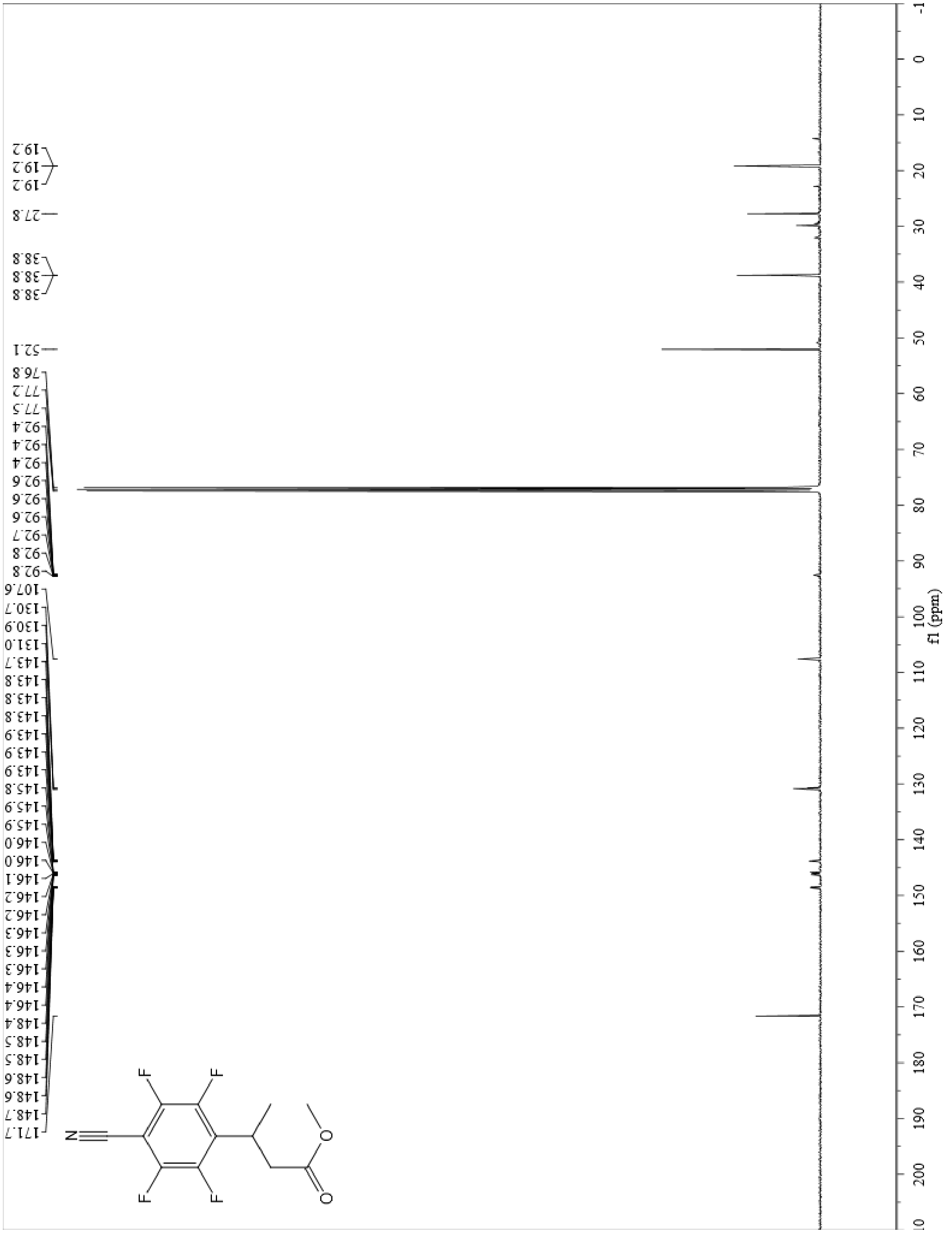

GC and MS of S-19a methyl 3-(4-cyano-2,3,5,6-tetrafluorophenyl)butanoate

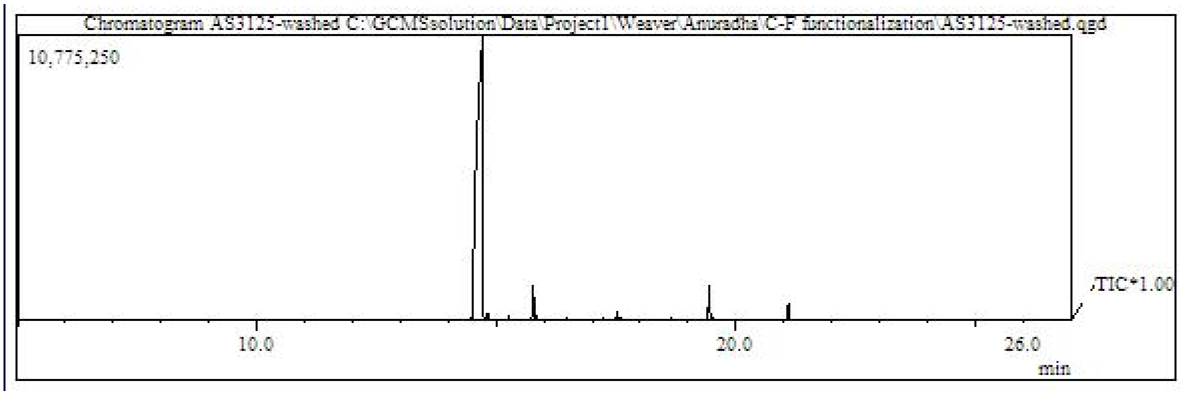

Spectrum

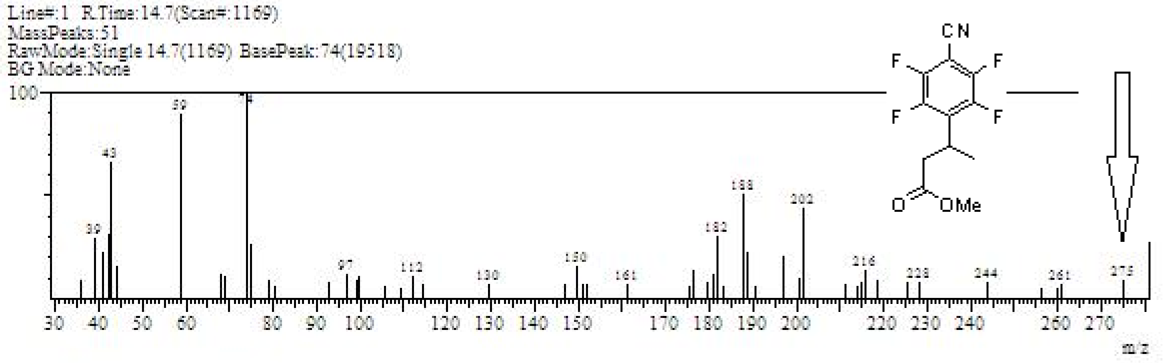

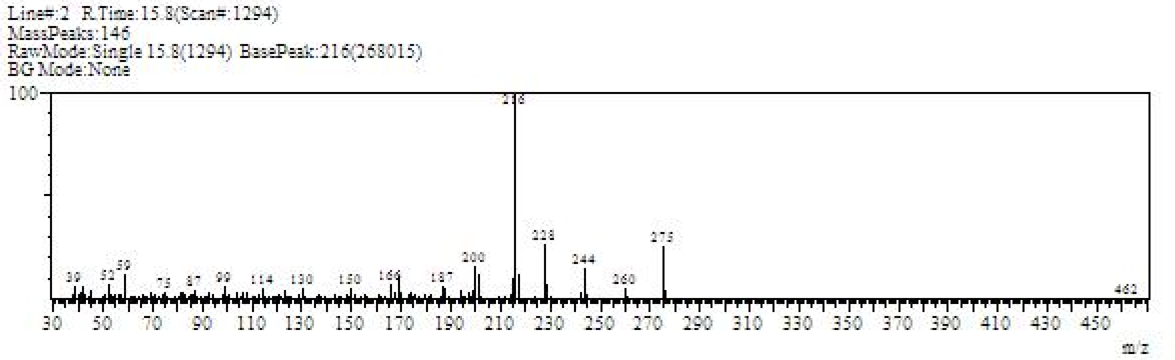

^{19}F NMR (376 MHz, Chloroform-*d*, @ rt) of S-20a ethyl 3-(4-acetyl-2,3,5,6-tetrafluorophenyl)butanoate

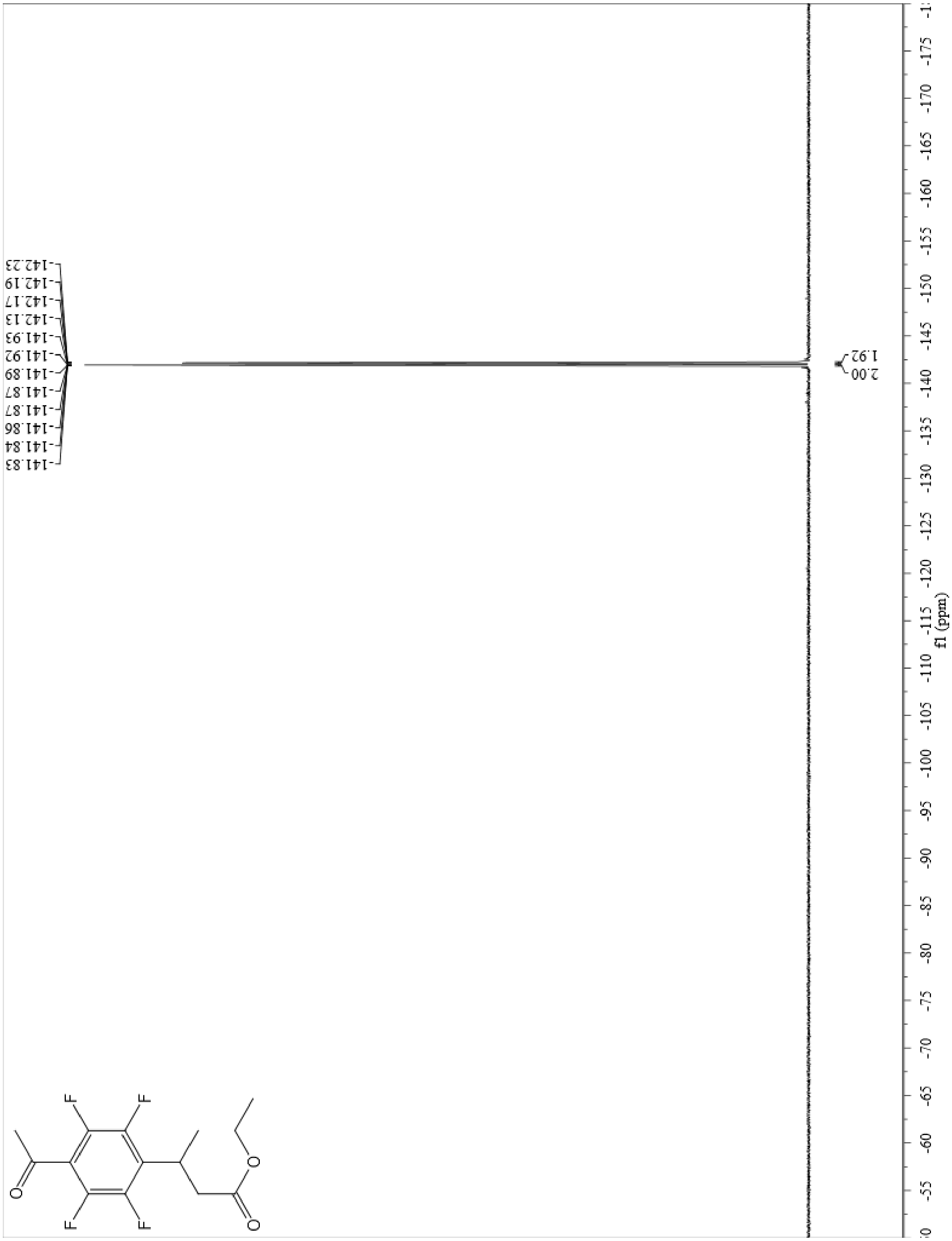

^{1}H NMR (400 MHz, Chloroform-*d*, @ rt) of S-20a ethyl 3-(4-acetyl-2,3,5,6-tetrafluorophenyl)butanoate

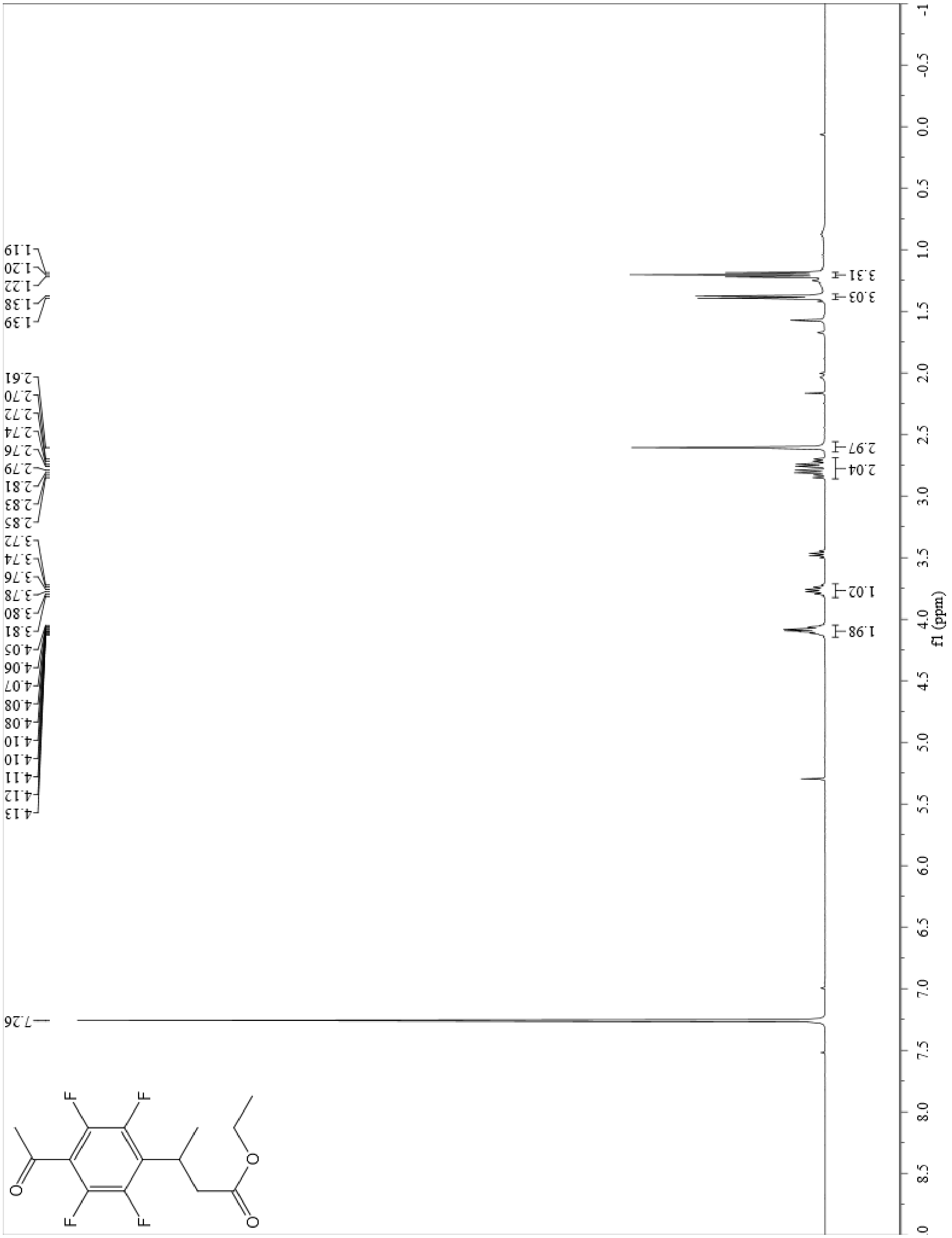

$^{13}C\{^{1}H\}$ NMR (101 MHz, Chloroform-*d*, @ rt) of S-20a ethyl 3-(4-acetyl-2,3,5,6-tetrafluorophenyl)butanoate

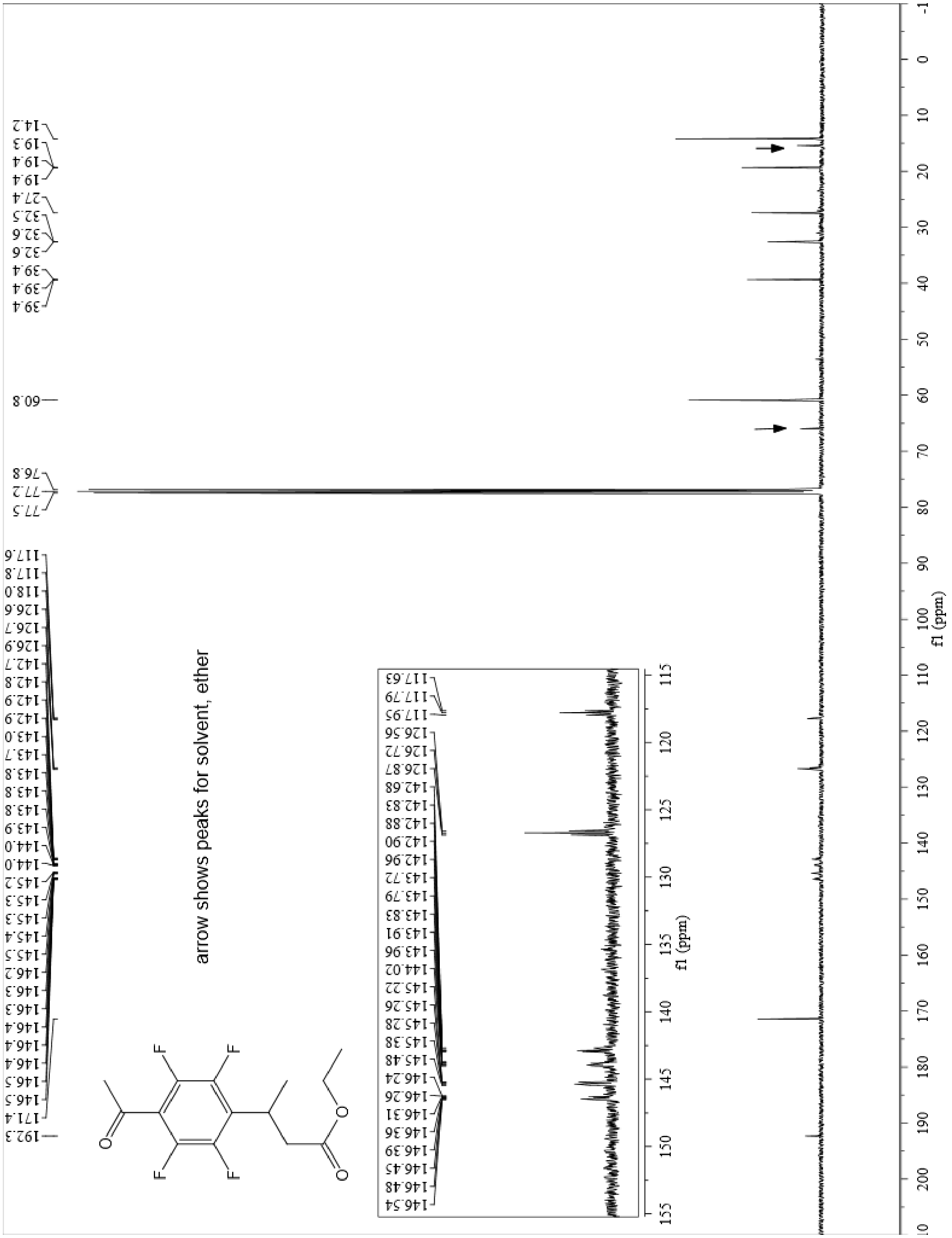

GC and MS of S-20a ethyl 3-(4-acetyl-2,3,5,6-tetrafluorophenyl)butanoate

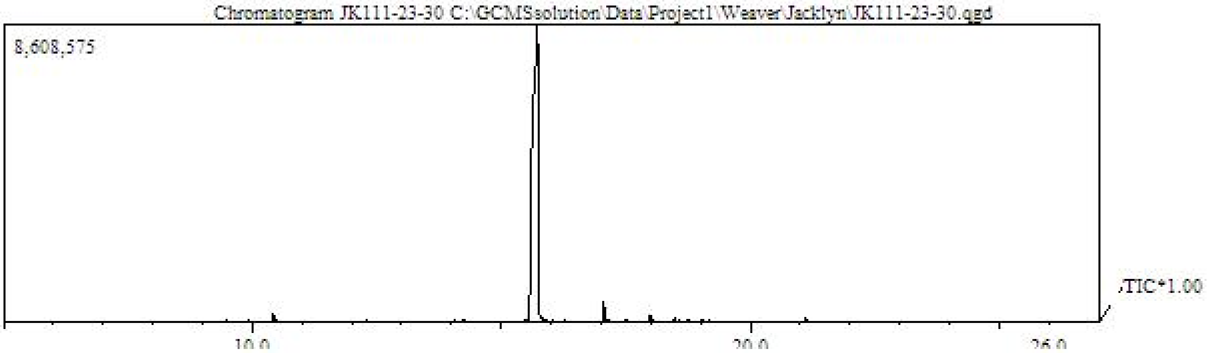

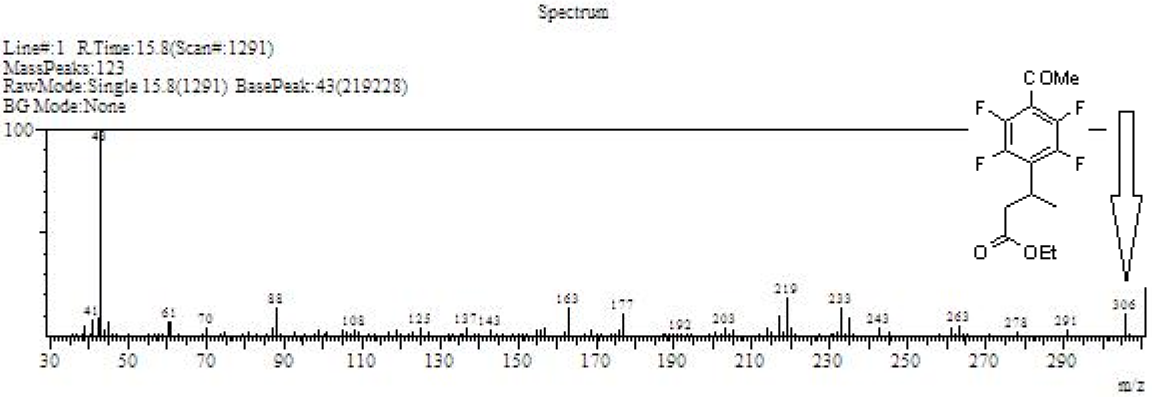

^{19}F NMR (376 MHz, Chloroform-*d*, @ rt) of S-21a 2,3,5,6-tetrafluoro-4-(2-methylcyclohexyl)benzonitrile (376 MHz, Chloroform-*d*, @ rt)

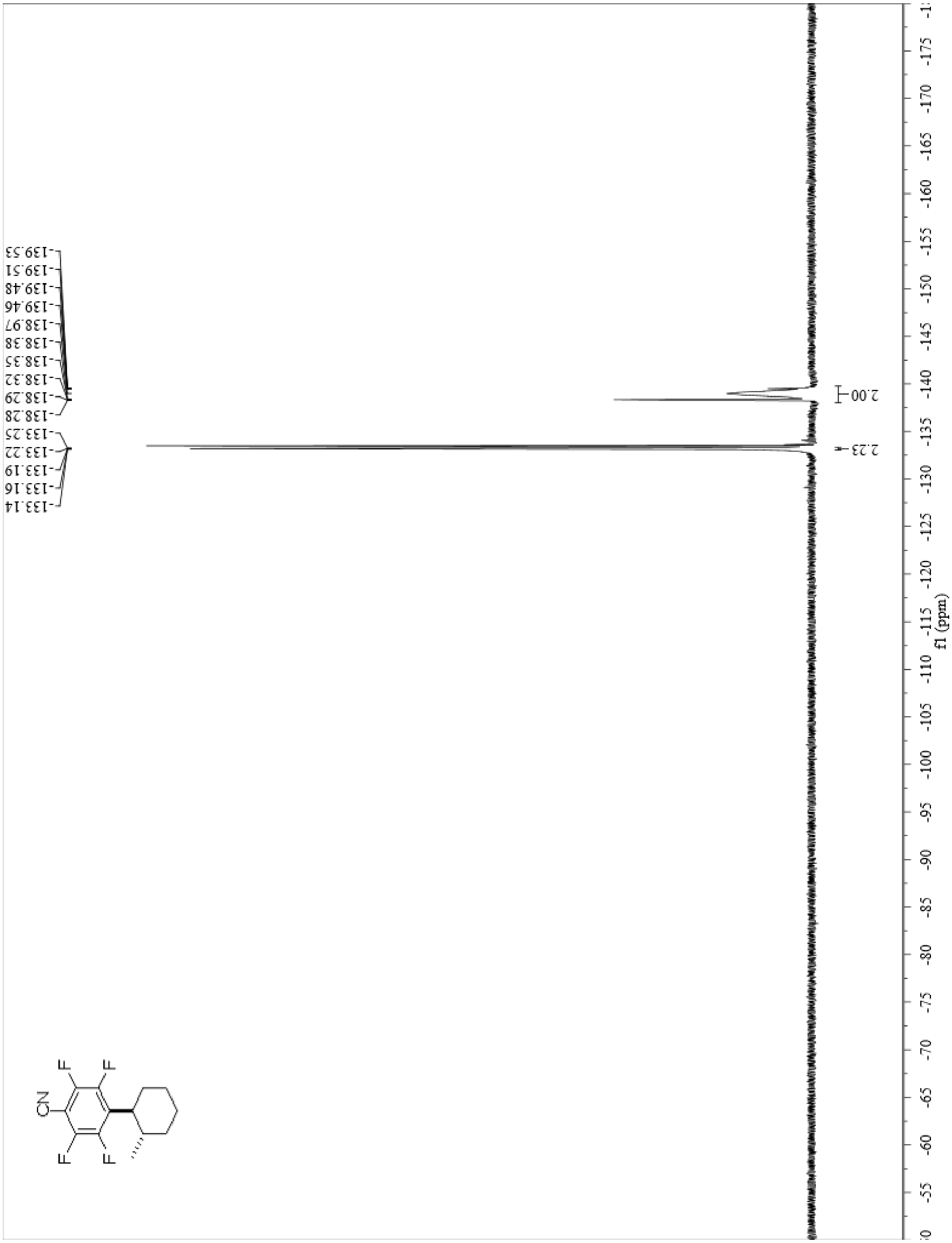

^{1}H NMR (400 MHz, Chloroform-*d*, @ rt) of S-21a 2,3,5,6-tetrafluoro-4-(2-methylcyclohexyl)benzonitrile

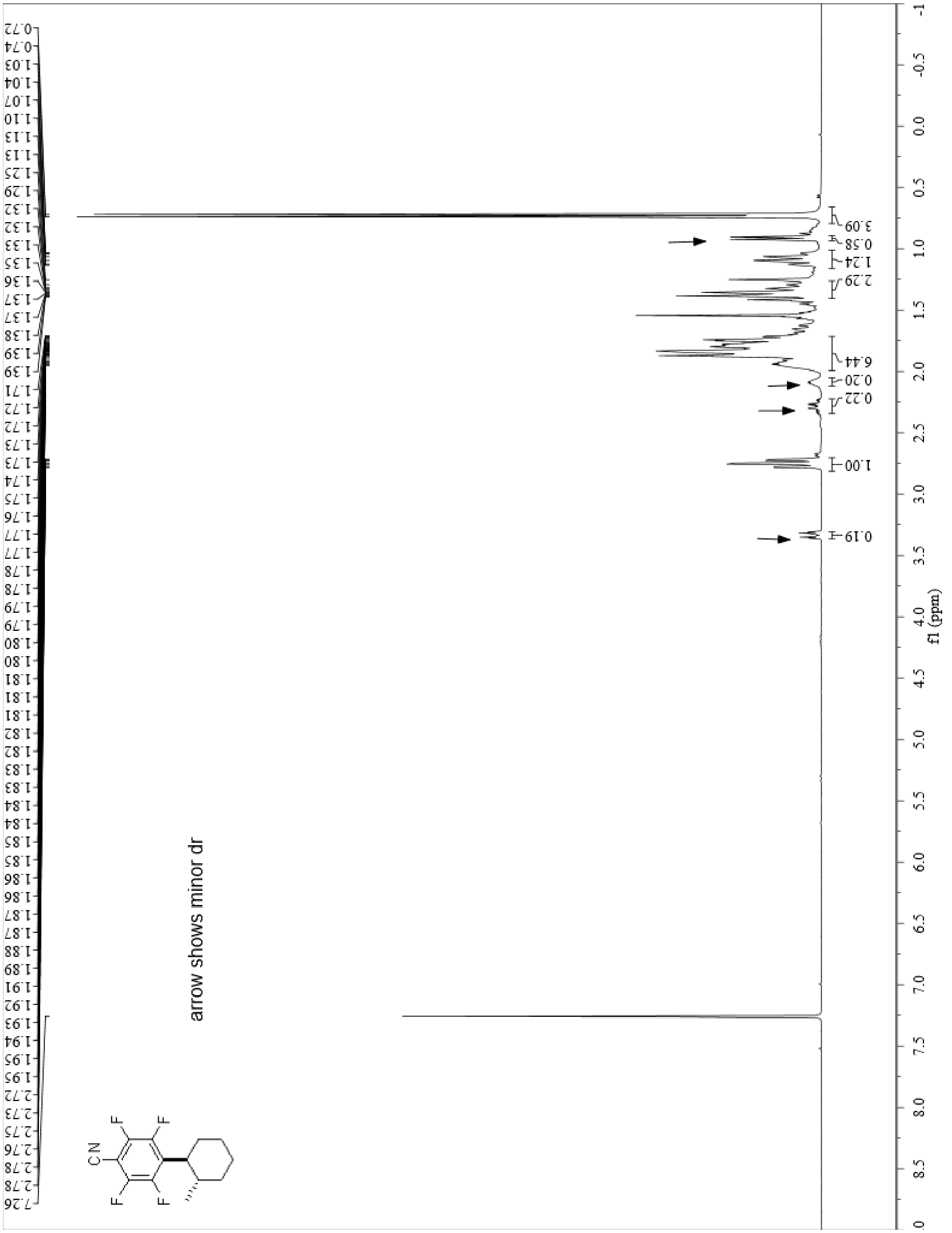

$^{13}C\{^{1}H\}$ NMR (101 MHz, Chloroform-*d*, @ rt) of S-21a 2,3,5,6-tetrafluoro-4-(2-methylcyclohexyl)benzonitrile

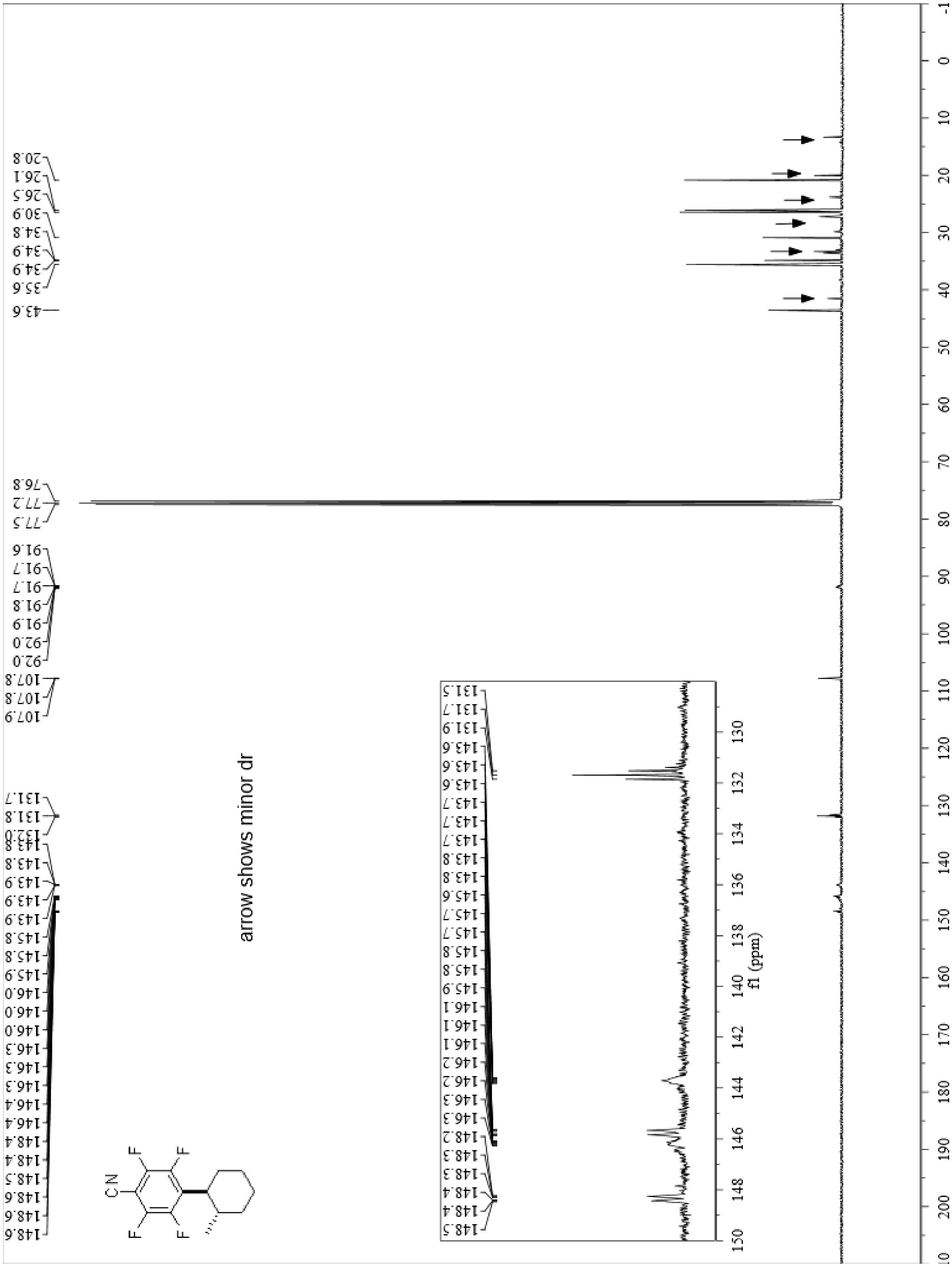

GC and MS of S-21a 2,3,5,6-tetrafluoro-4-(2-methylcyclohexyl)benzonitrile

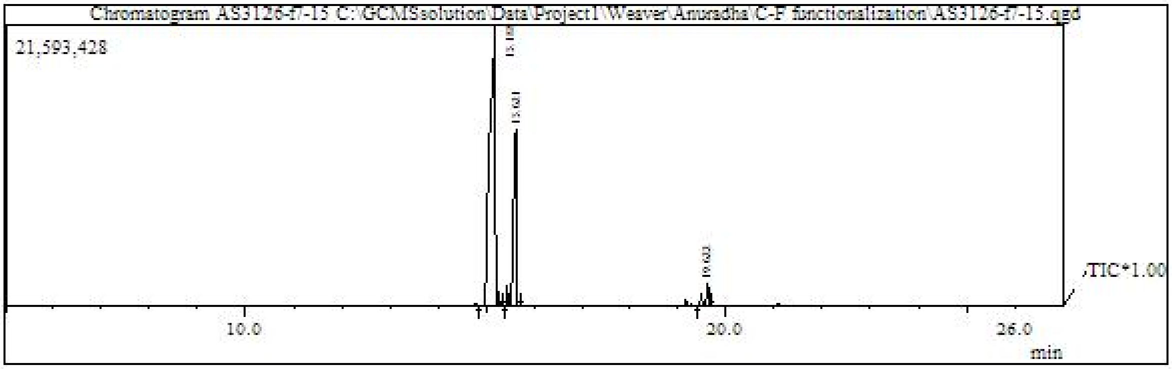

Peak Report TIC

Peak#	R.Time	I.Time	F.Time	Area	Area%	Height	Height%	A/H	Mark	Name
1	15.188	14.833	15.342	171855763	73.21	21562792	58.65	7.97	MI	
2	15.621	15.375	15.742	55503076	23.65	13545163	36.84	4.09	MI	
3	19.622	19.383	19.717	7372916	3.14	1657393	4.51	4.44	MI	
				234731755	100.00	36765348	100.00			

Line#:2 R.Time:15.0(Scan#:1205)
MassPeaks:189
RawMode:Single 15.0(1205) BasePeak:55(1161374)
BG Mode:None

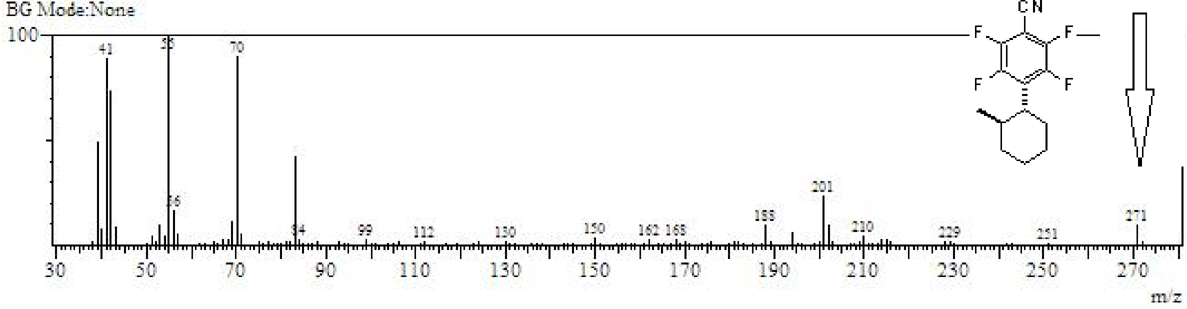

Line#:3 R.Time:15.6(Scan#:1278)
MassPeaks:67
RawMode:Single 15.6(1278) BasePeak:41(79575)
BG Mode:None

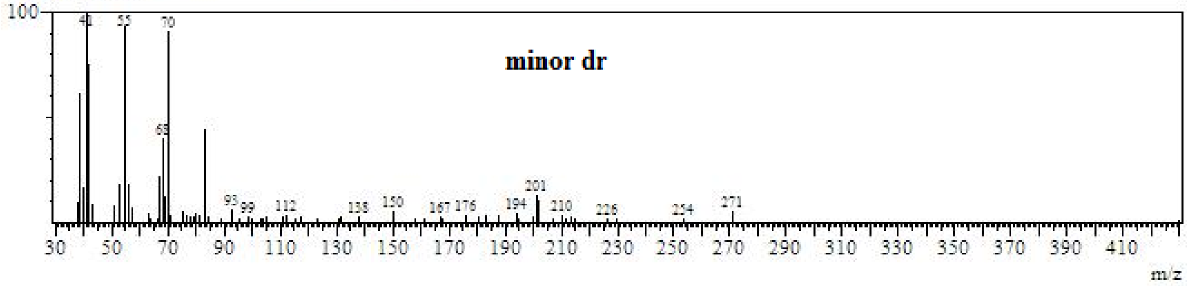

^{19}F NMR (376 MHz, Chloroform-*d*, @ rt) of S-22a 7-cyclohexyl-4,5,6-trifluoro-2-phenylbenzofuran

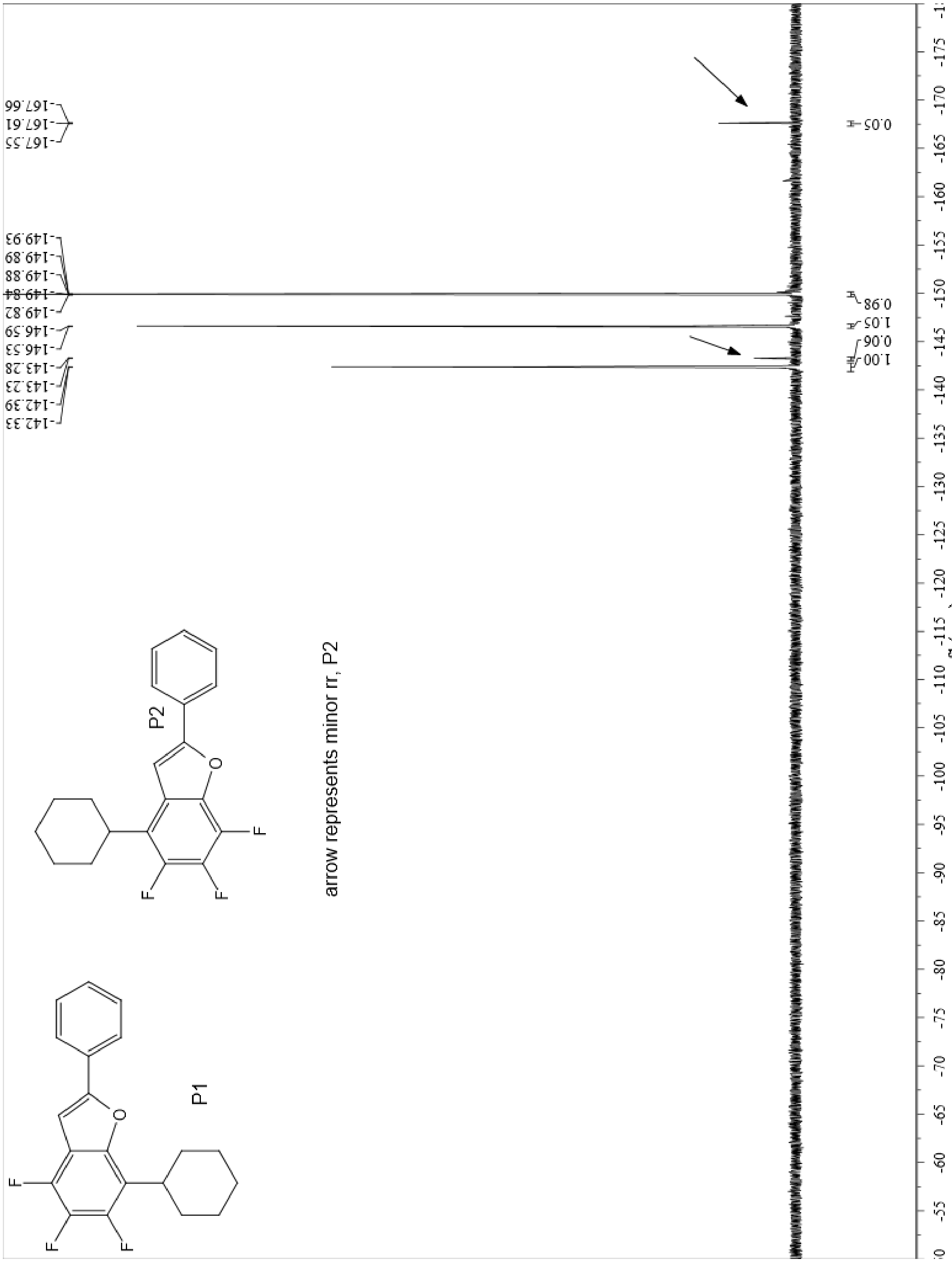

^{1}H NMR (400 MHz, Chloroform-*d*, @ rt) of S-22a 7-cyclohexyl-4,5,6-trifluoro-2-phenylbenzofuran

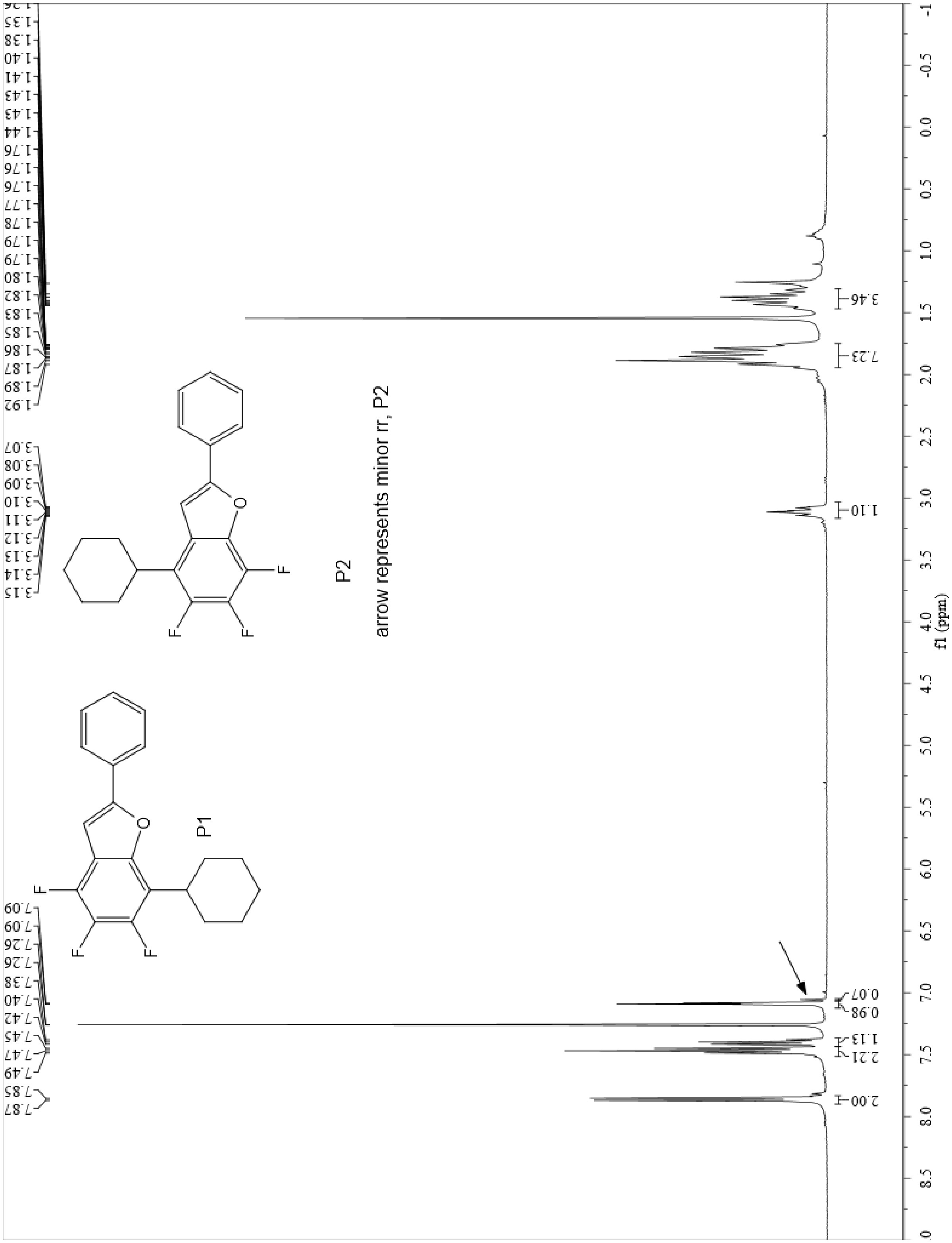

$^{13}C\{^{1}H\}$ NMR (101 MHz, Chloroform-*d*, @ rt) of S-22a 7-cyclohexyl-4,5,6-trifluoro-2-phenylbenzofuran

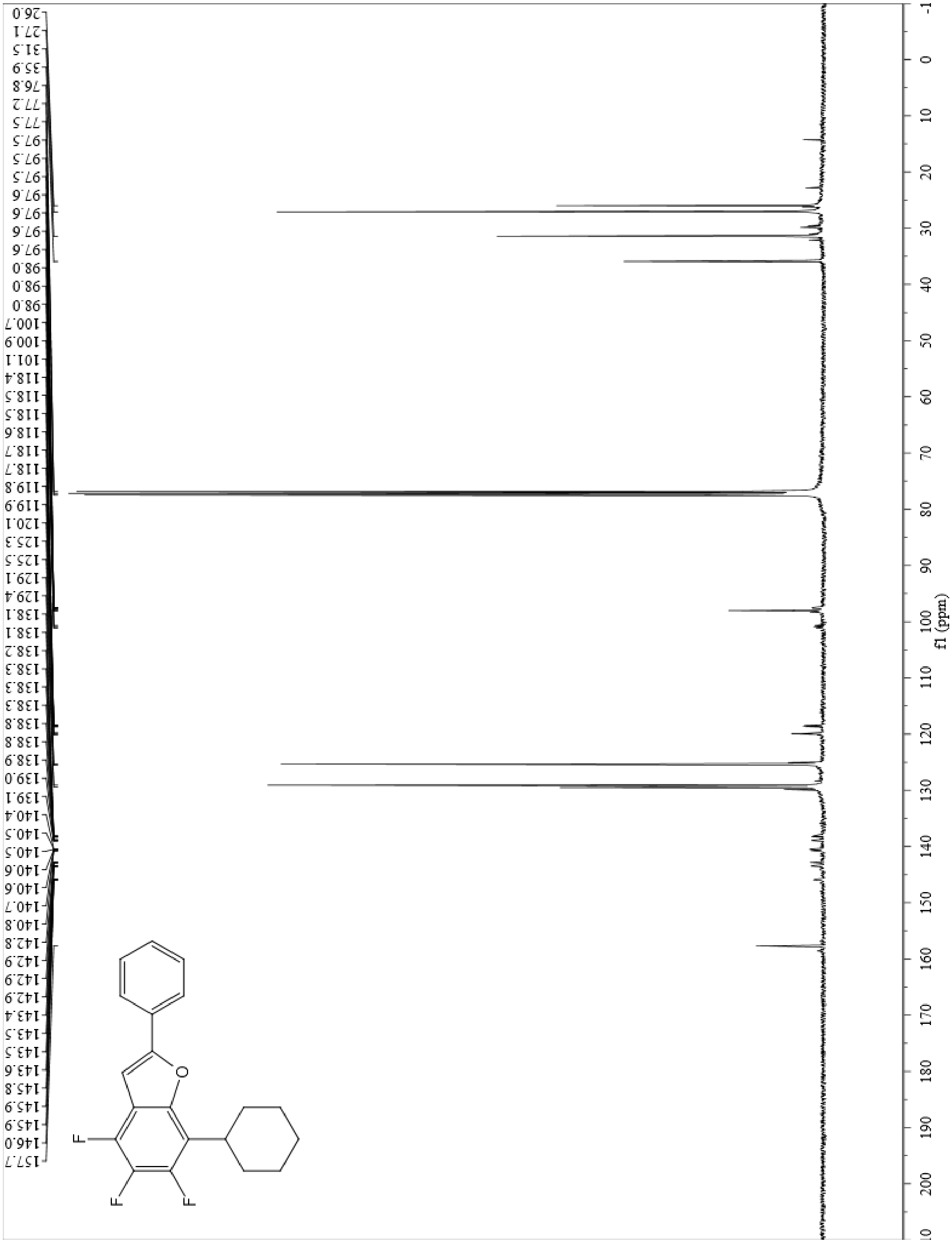

GC and MS of S-22a 7-cyclohexyl-4,5,6-trifluoro-2-phenylbenzofuran

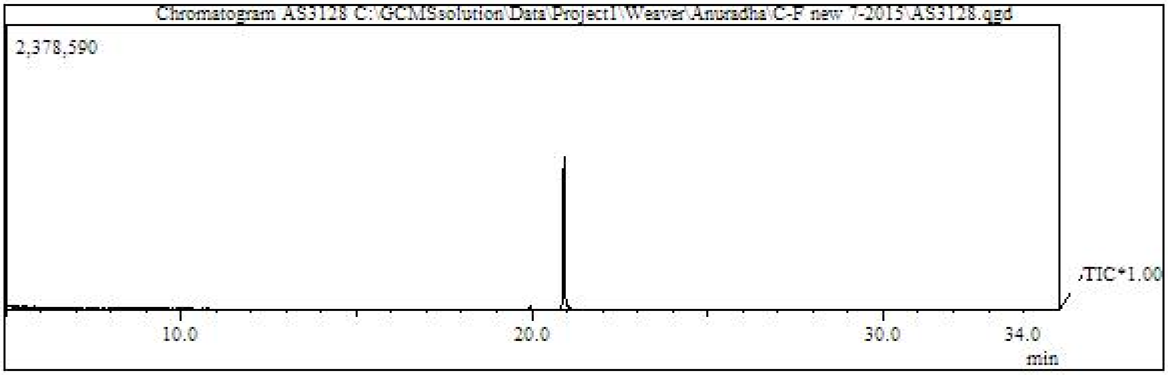

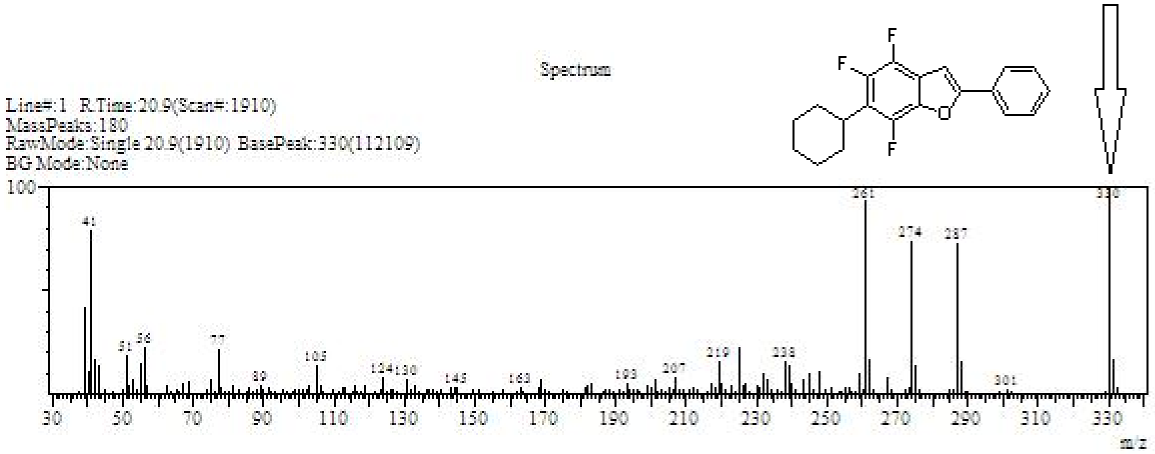

^{19}F NMR (376 MHz, Chloroform-*d*, @ rt) of S-23a 2-(2,4,5-trifluoro-3-phenoxy-6-(trifluoromethyl)phenyl)bicyclo[2.2.1]heptane

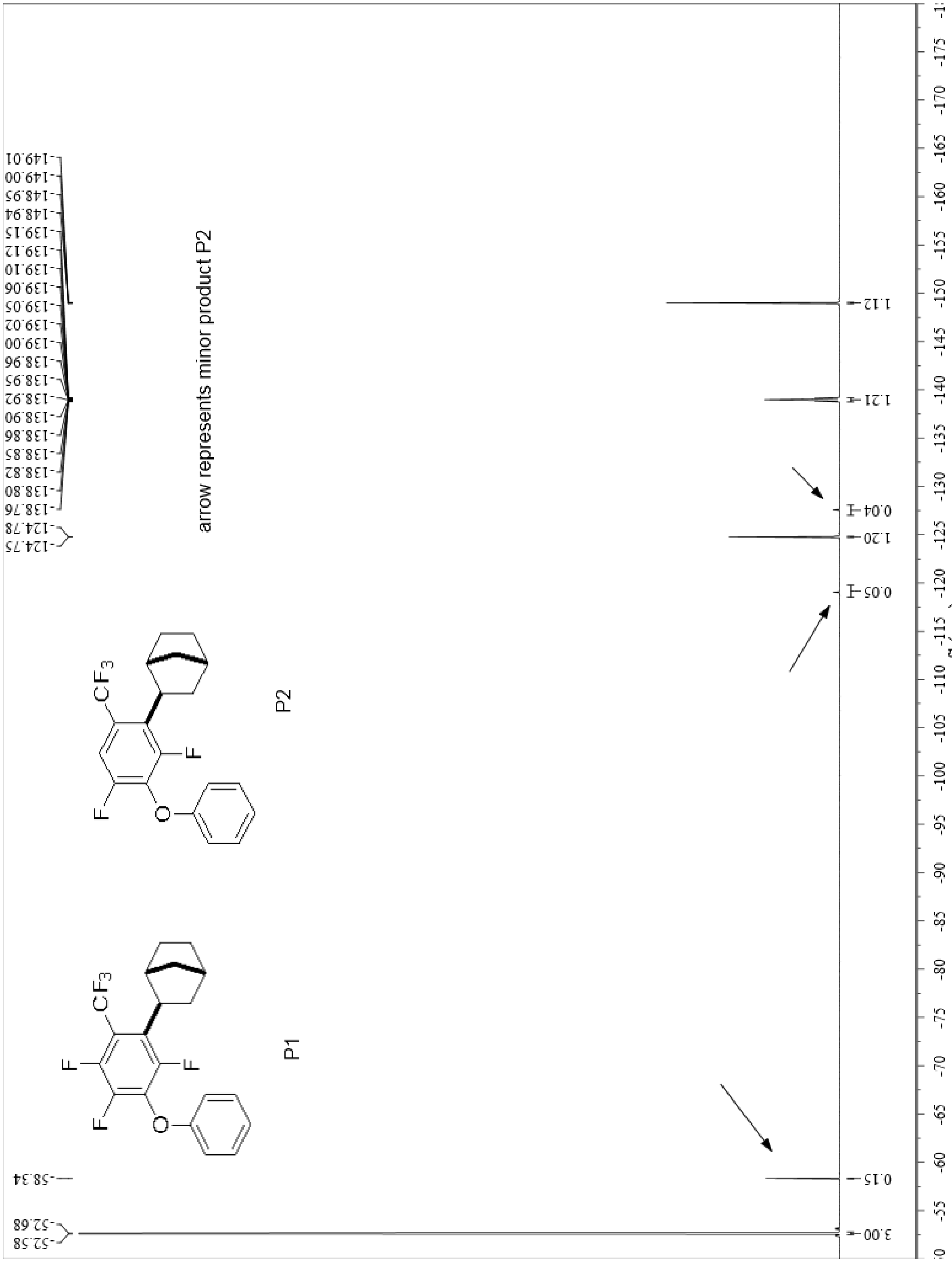

^{1}H NMR (400 MHz, Chloroform-*d*, @ rt) of S-23a 2-(2,4,5-trifluoro-3-phenoxy-6-(trifluoromethyl)phenyl)bicyclo[2.2.1]heptane

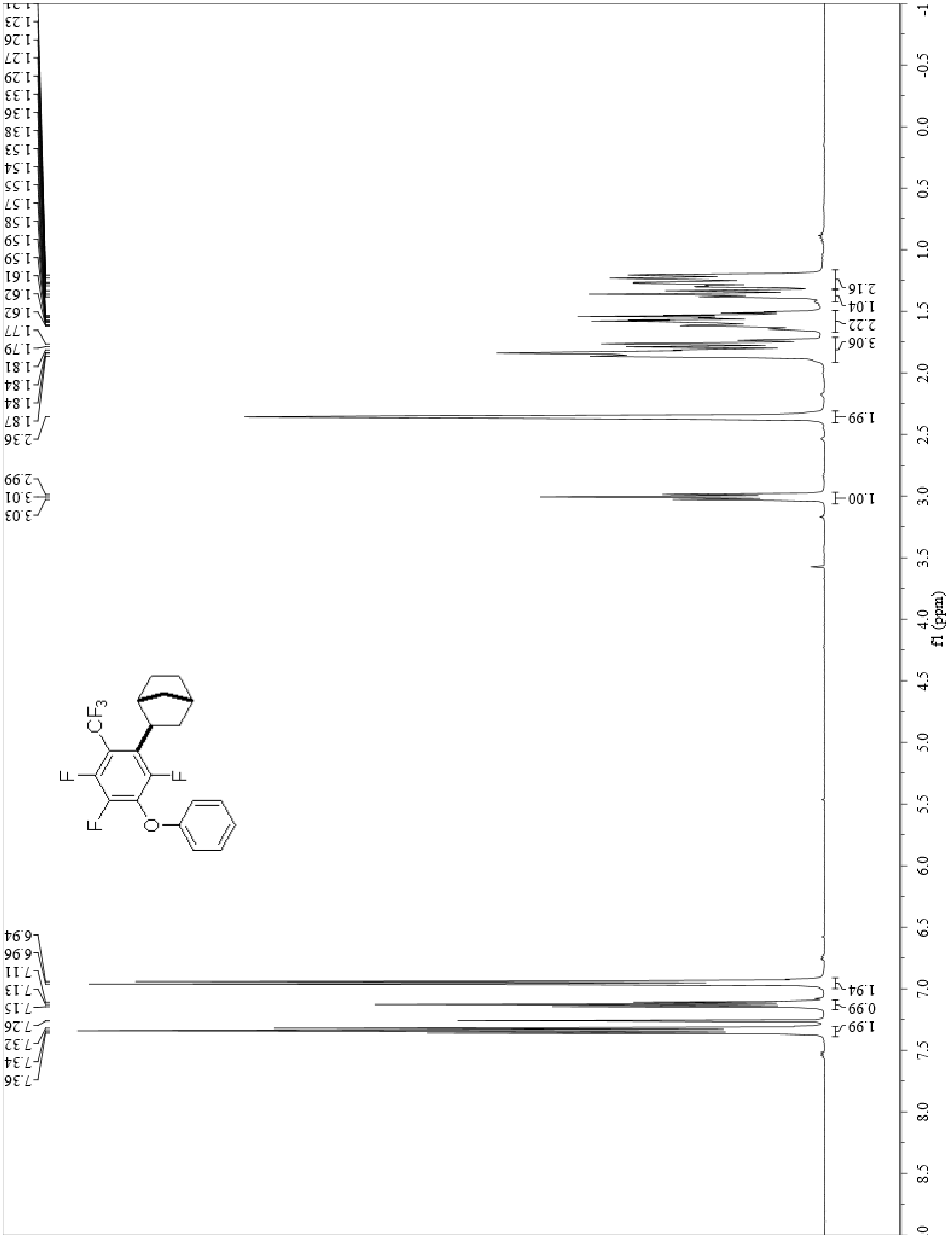

$^{13}C\{^{1}H\}$ NMR (101 MHz, Chloroform-*d*, @ rt) of S-23a 2-(2,4,5-trifluoro-3-phenoxy-6-(trifluoromethyl)phenyl)bicyclo[2.2.1]heptane

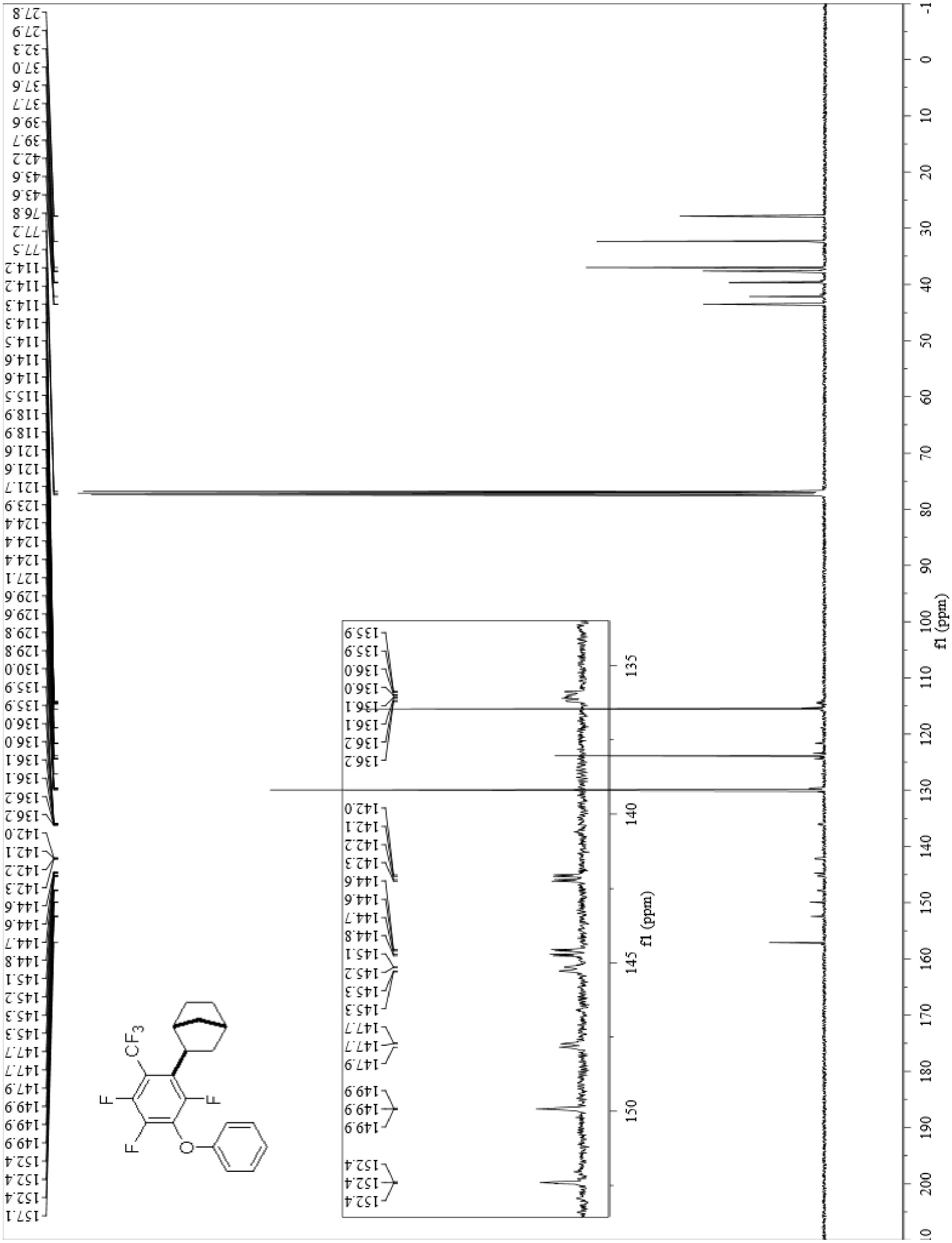

GC and MS of S-23a 2-(2,4,5-trifluoro-3-phenoxy-6-(trifluoromethyl)phenyl)bicyclo[2.2.1]heptane

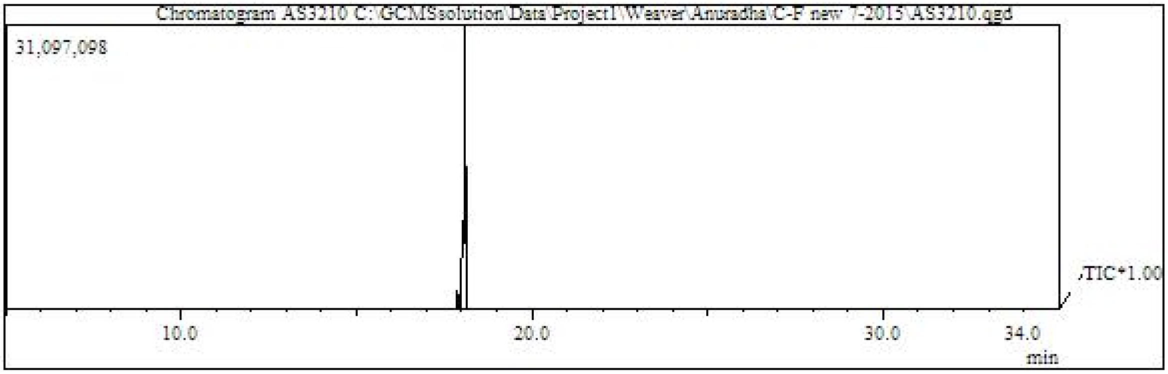

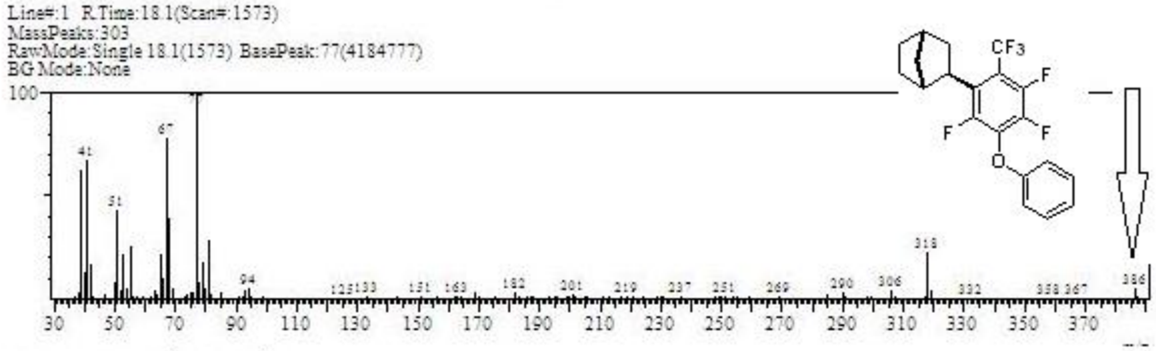

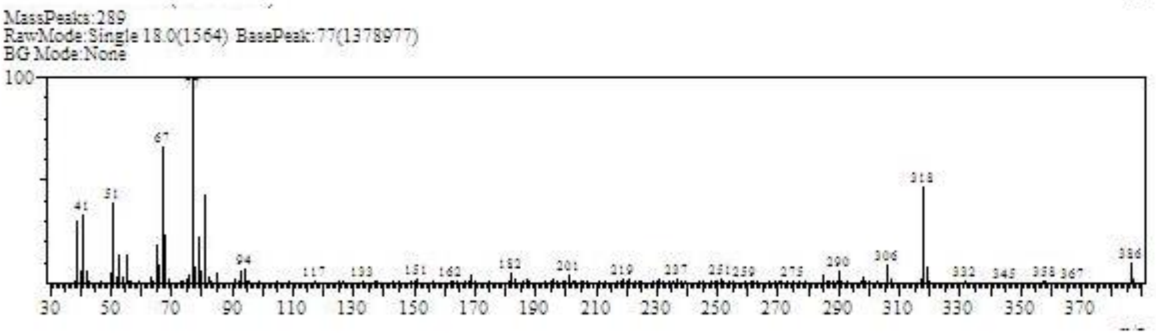

^{19}F NMR (376 MHz, Chloroform-*d*, @ rt) of S-24a methyl 2-(bicyclo[2.2.1]heptan-2-yl)-3,5,6-trifluoro-4-(2-methoxy-2-oxoethyl)benzoate

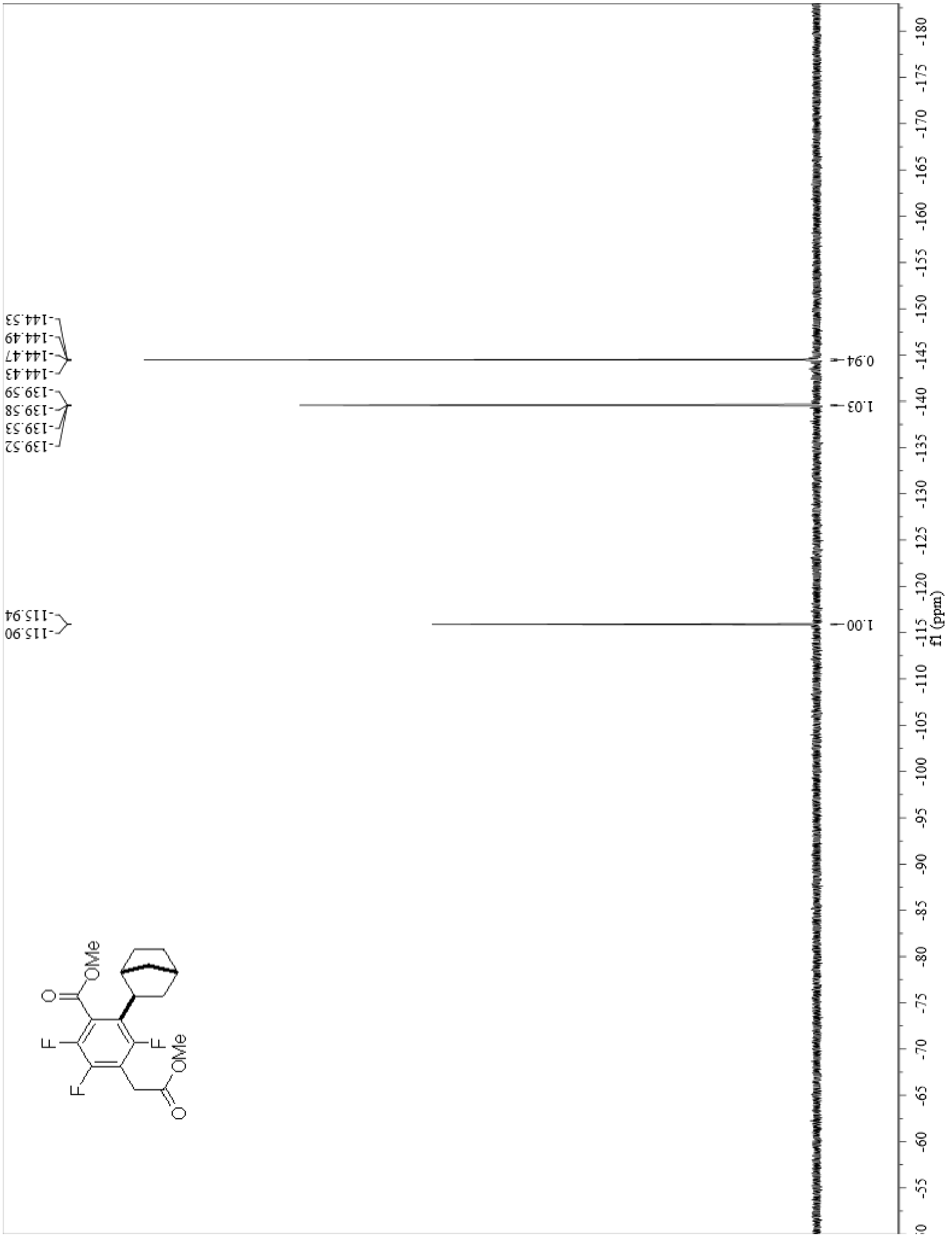

^{1}H NMR (400 MHz, Chloroform-*d*, @ rt) of S-24a methyl 2-(bicyclo[2.2.1]heptan-2-yl)-3,5,6-trifluoro-4-(2-methoxy-2-oxoethyl)benzoate

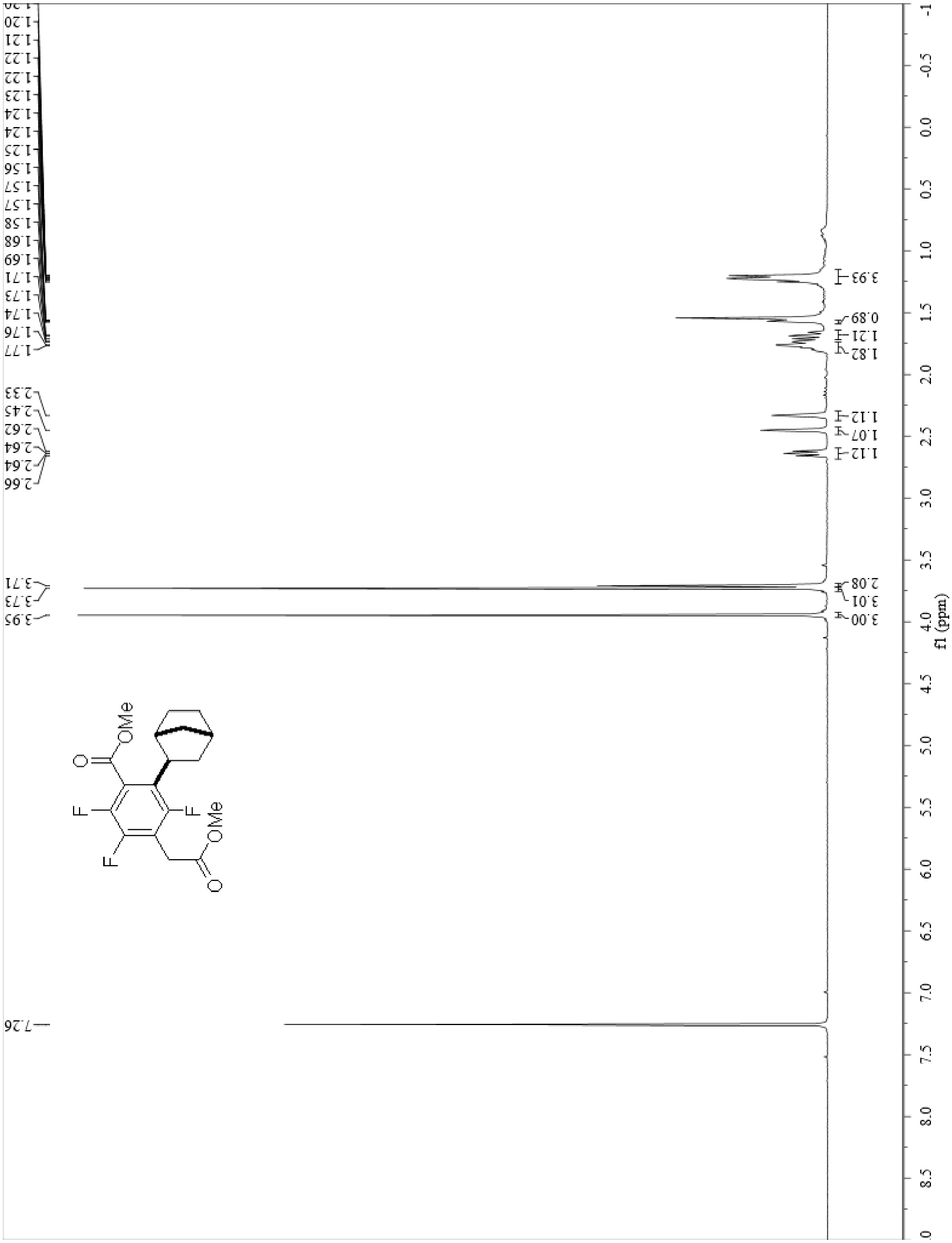

$^{13}C\{^{1}H\}$ NMR (101 MHz, Chloroform-*d*, @ rt) of S-24a methyl 2-(bicyclo[2.2.1]heptan-2-yl)-3,5,6-trifluoro-4-(2-methoxy-2-oxoethyl)benzoate

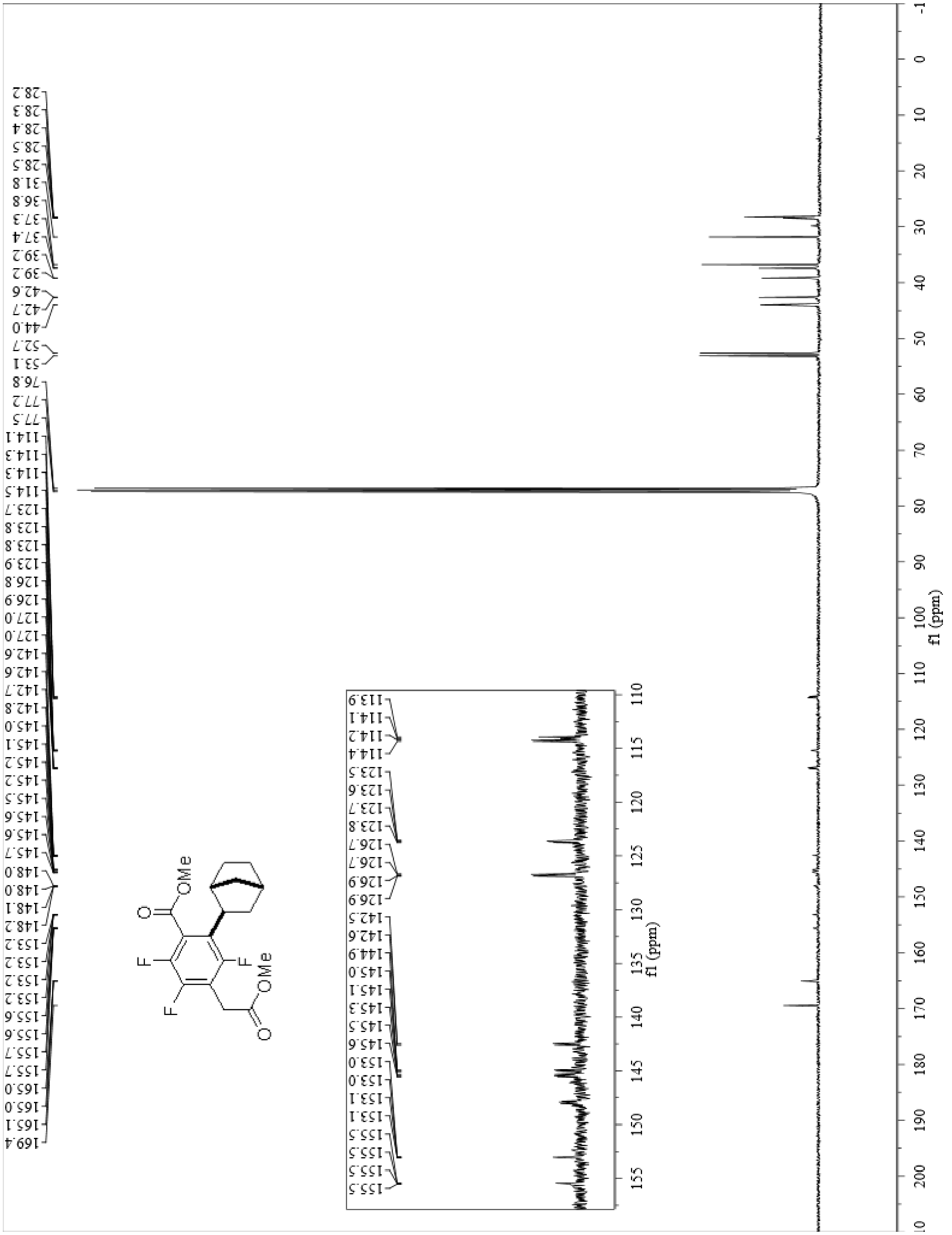

GC and MS of S-24a methyl 2-(bicyclo[2.2.1]heptan-2-yl)-3,5,6-trifluoro-4-(2-methoxy-2-oxoethyl)benzoate

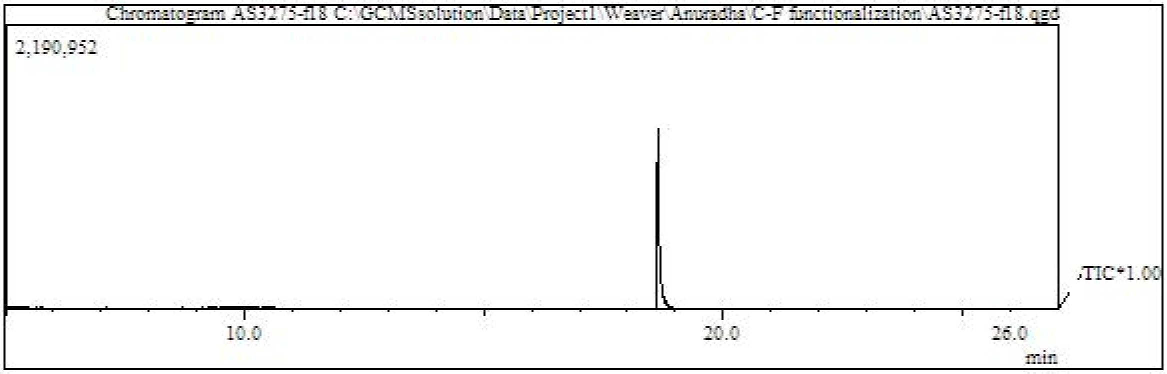

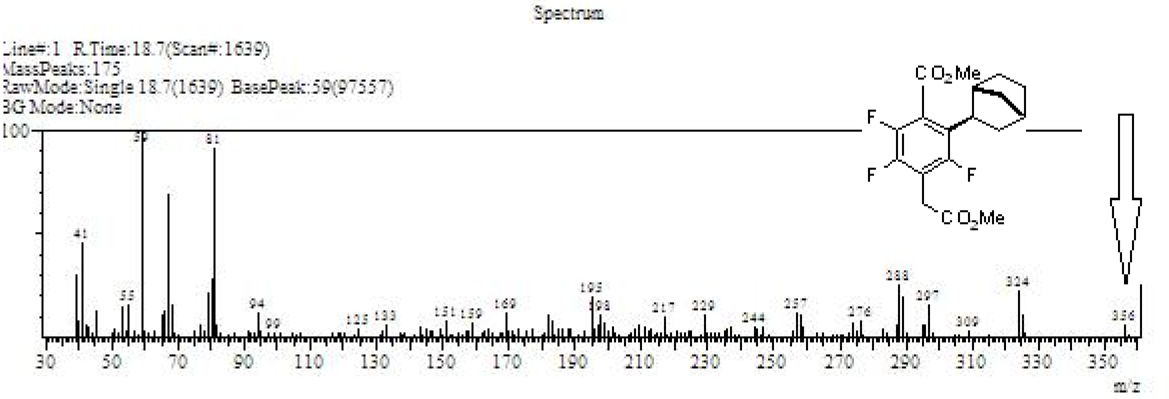

^{19}F NMR (376 MHz, Chloroform-*d*, @ rt) of S-25a N-(3-(bicyclo[2.2.1]heptan-2-yl)-2,5,6-trifluoro-4-(trifluoromethyl)phenyl)acetamide

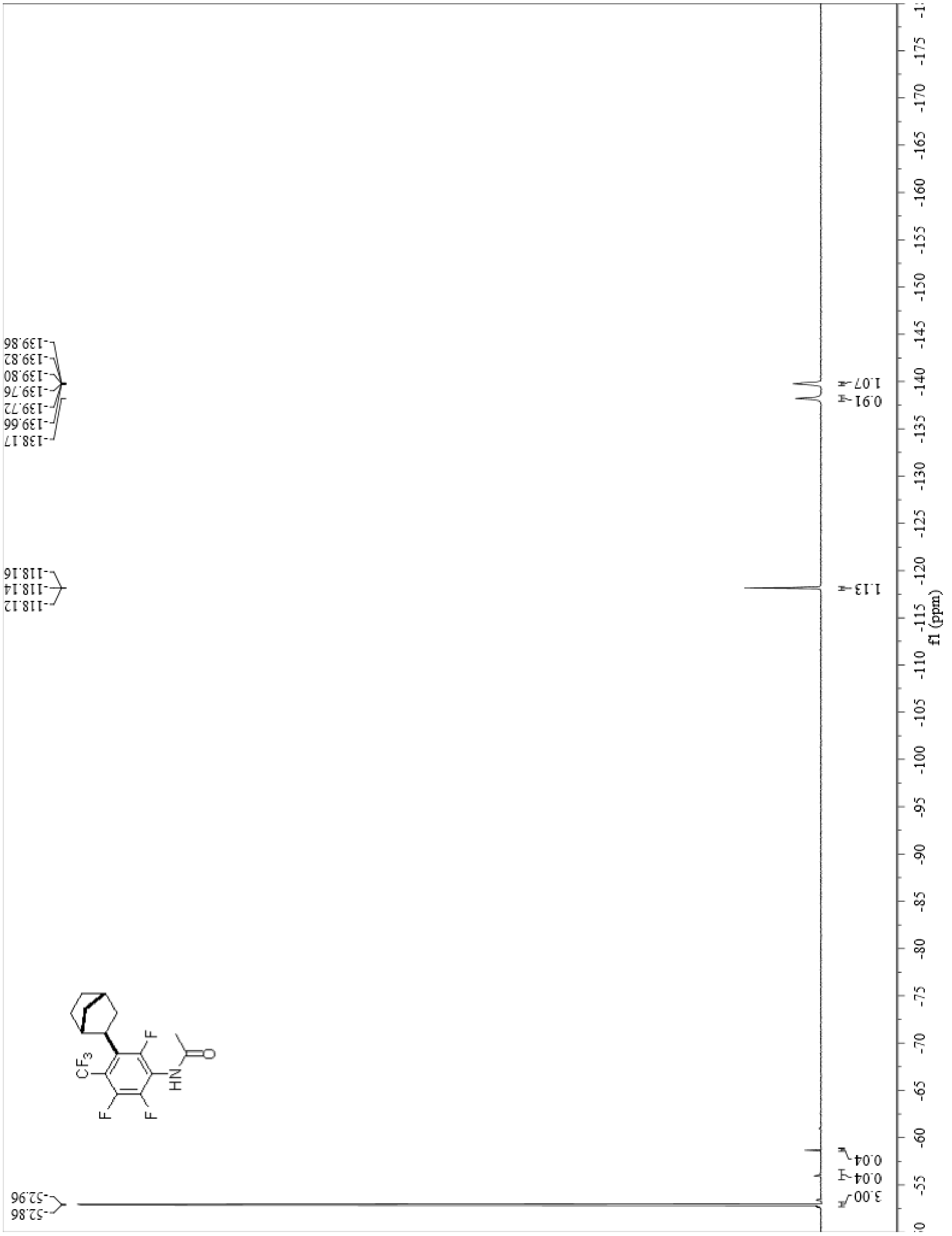

^{1}H NMR (400 MHz, Chloroform-*d*, @ rt) of S-25a N-(3-(bicyclo[2.2.1]heptan-2-yl)-2,5,6-trifluoro-4-(trifluoromethyl)phenyl)acetamide

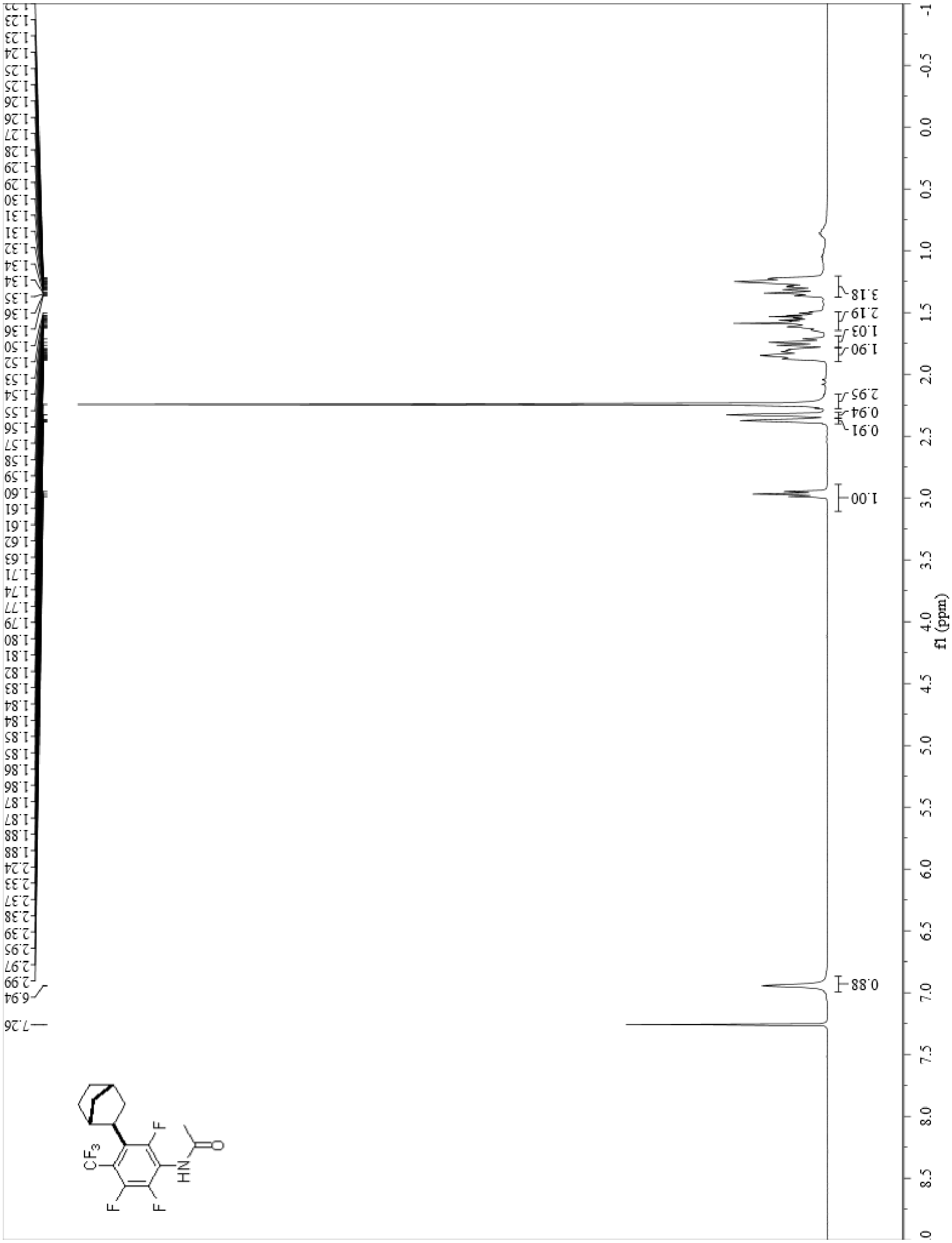

$^{13}C\{^1H\}$ NMR (101 MHz, Chloroform-*d*, @ rt) of S-25a N-(3-(bicyclo[2.2.1]heptan-2-yl)-2,5,6-trifluoro-4-(trifluoromethyl)phenyl)acetamide

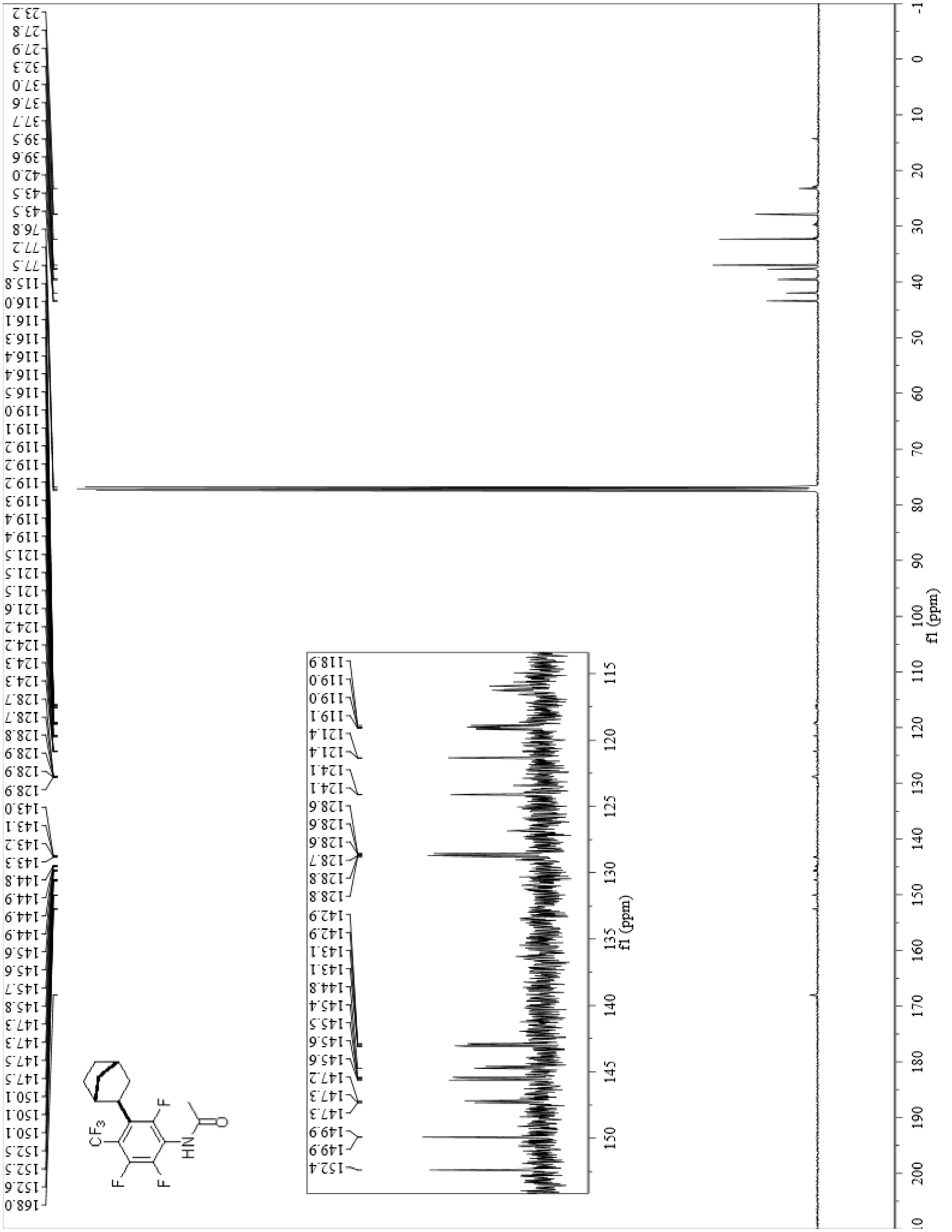

GC and MS of S-25a N-(3-(bicyclo[2.2.1]heptan-2-yl)-2,5,6-trifluoro-4-(trifluoromethyl)phenyl)acetamide

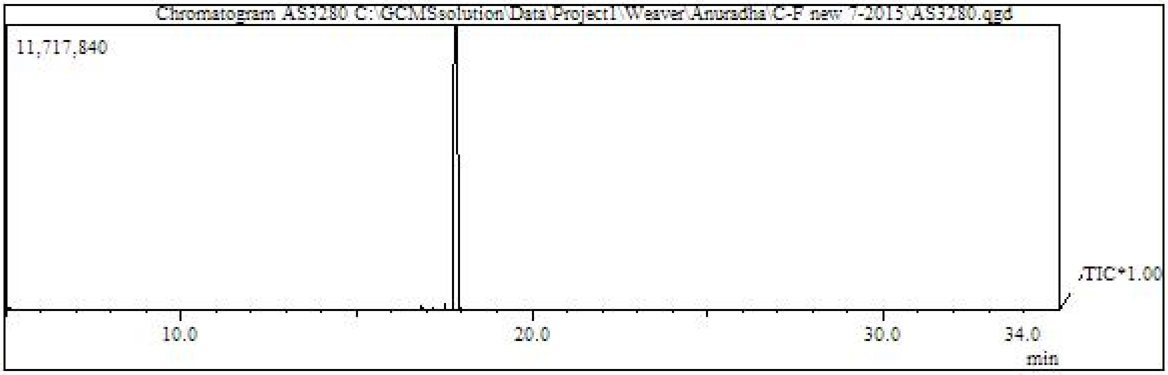

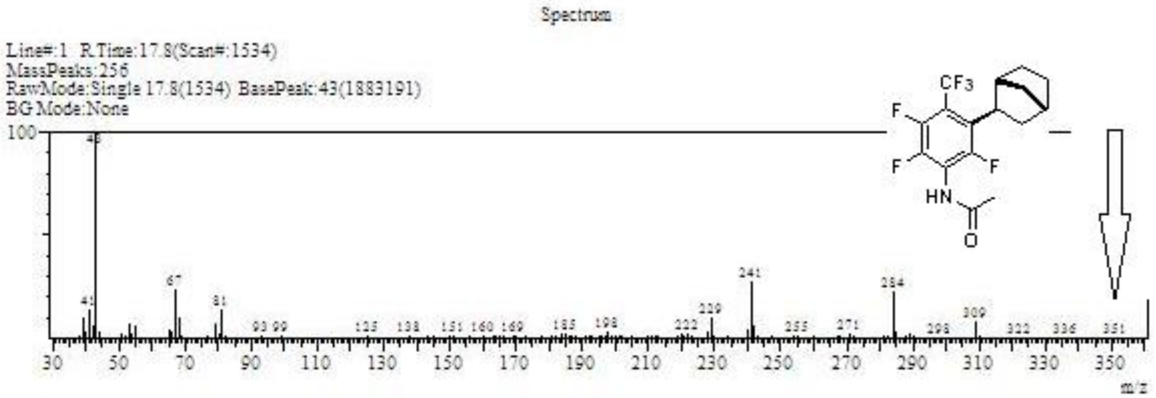

^{19}F NMR (376 MHz, Chloroform-*d*, @ rt) of S-26a methyl 2-(bicyclo[2.2.1]heptan-2-yl)-4-(2-((2-ethoxy-2-oxoethyl)amino)-2-oxoethyl)-3,5,6-trifluorobenzoate

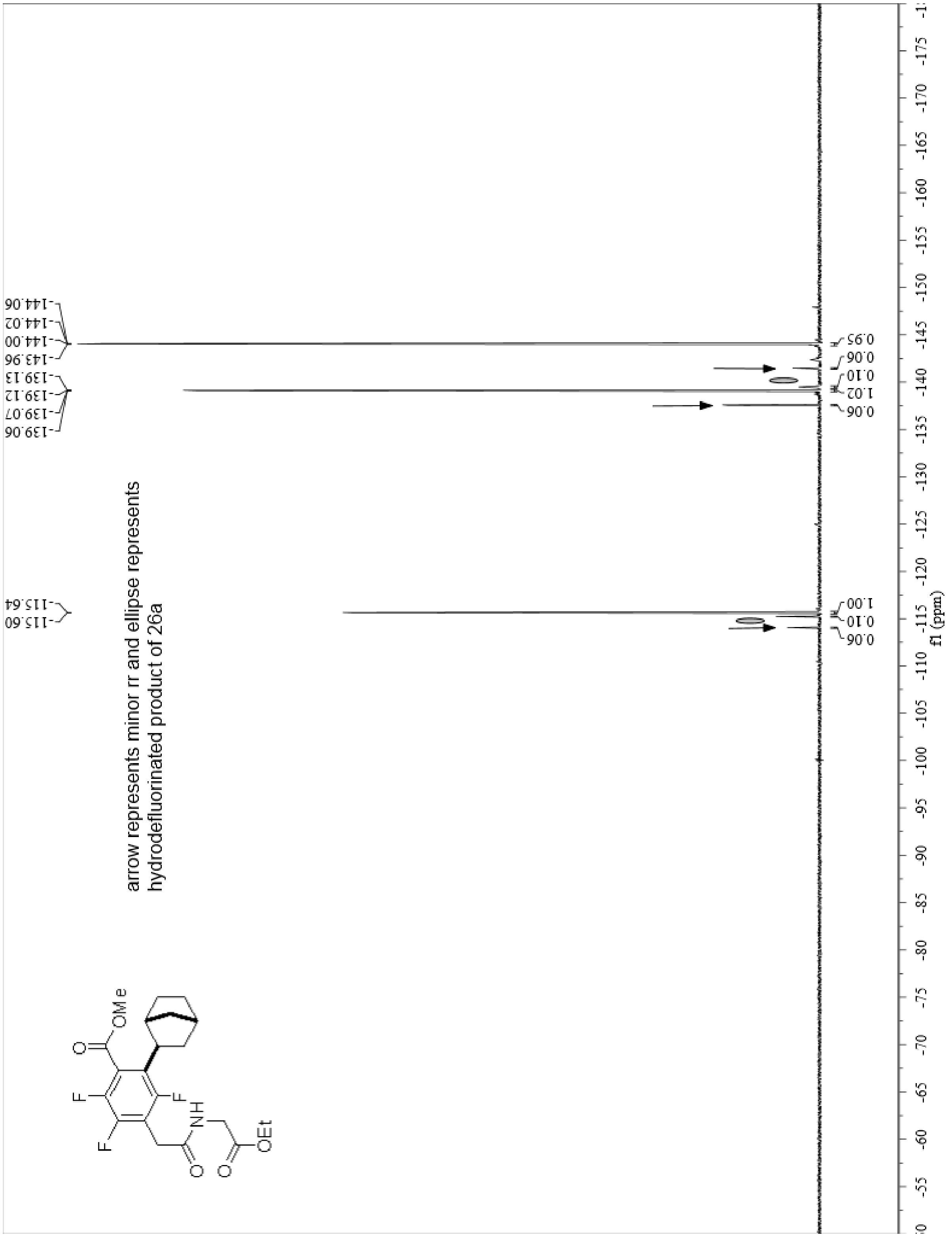

^{1}H NMR (400 MHz, Chloroform-*d*, @ rt) of S-26a methyl 2-(bicyclo[2.2.1]heptan-2-yl)-4-(2-((2-ethoxy-2-oxoethyl)amino)-2-oxoethyl)-3,5,6-trifluorobenzoate

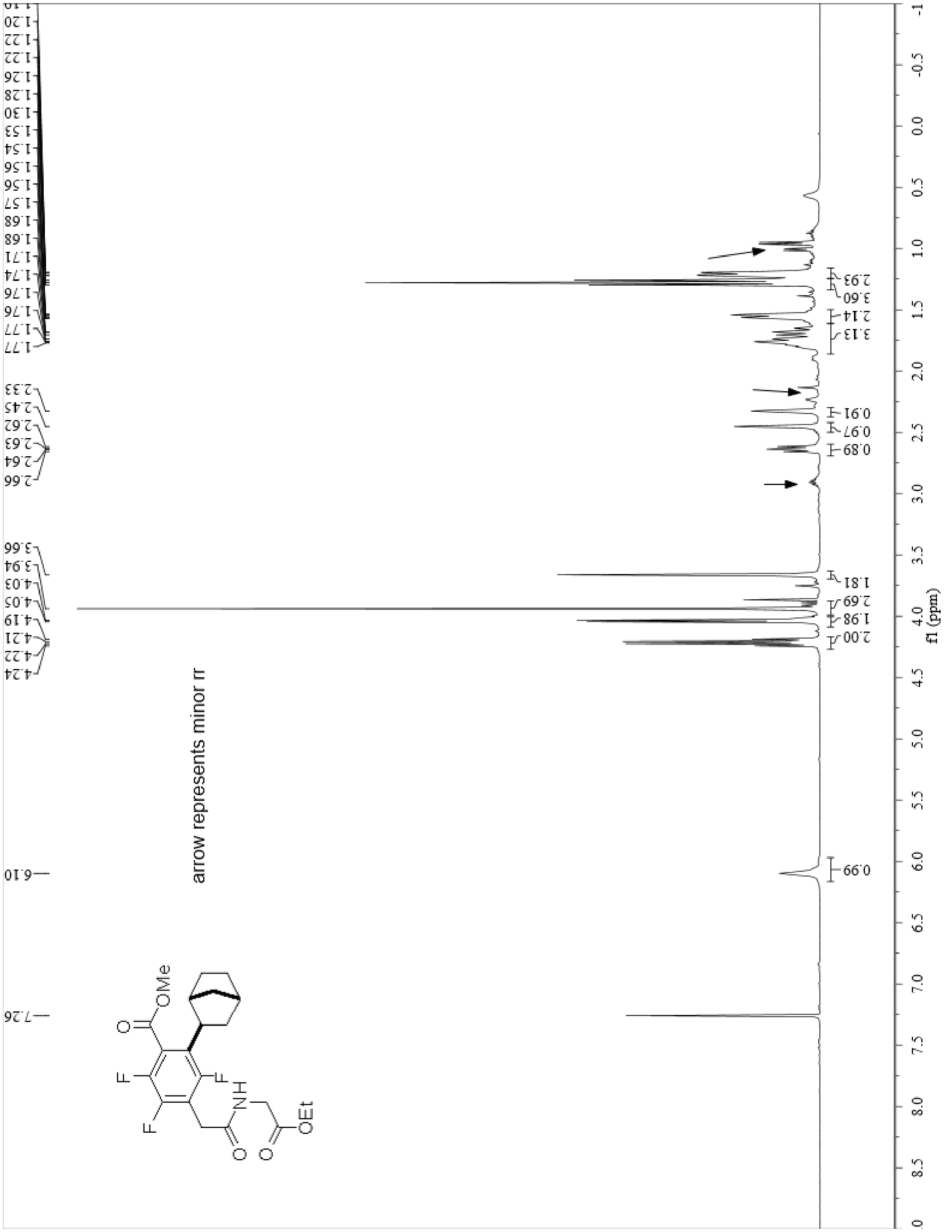

$^{13}C\{^{1}H\}$ NMR (101 MHz, Chloroform-*d*, @ rt) of S-26a methyl 2-(bicyclo[2.2.1]heptan-2-yl)-4-(2-((2-ethoxy-2-oxoethyl)amino)-2-oxoethyl)-3,5,6-trifluorobenzoate

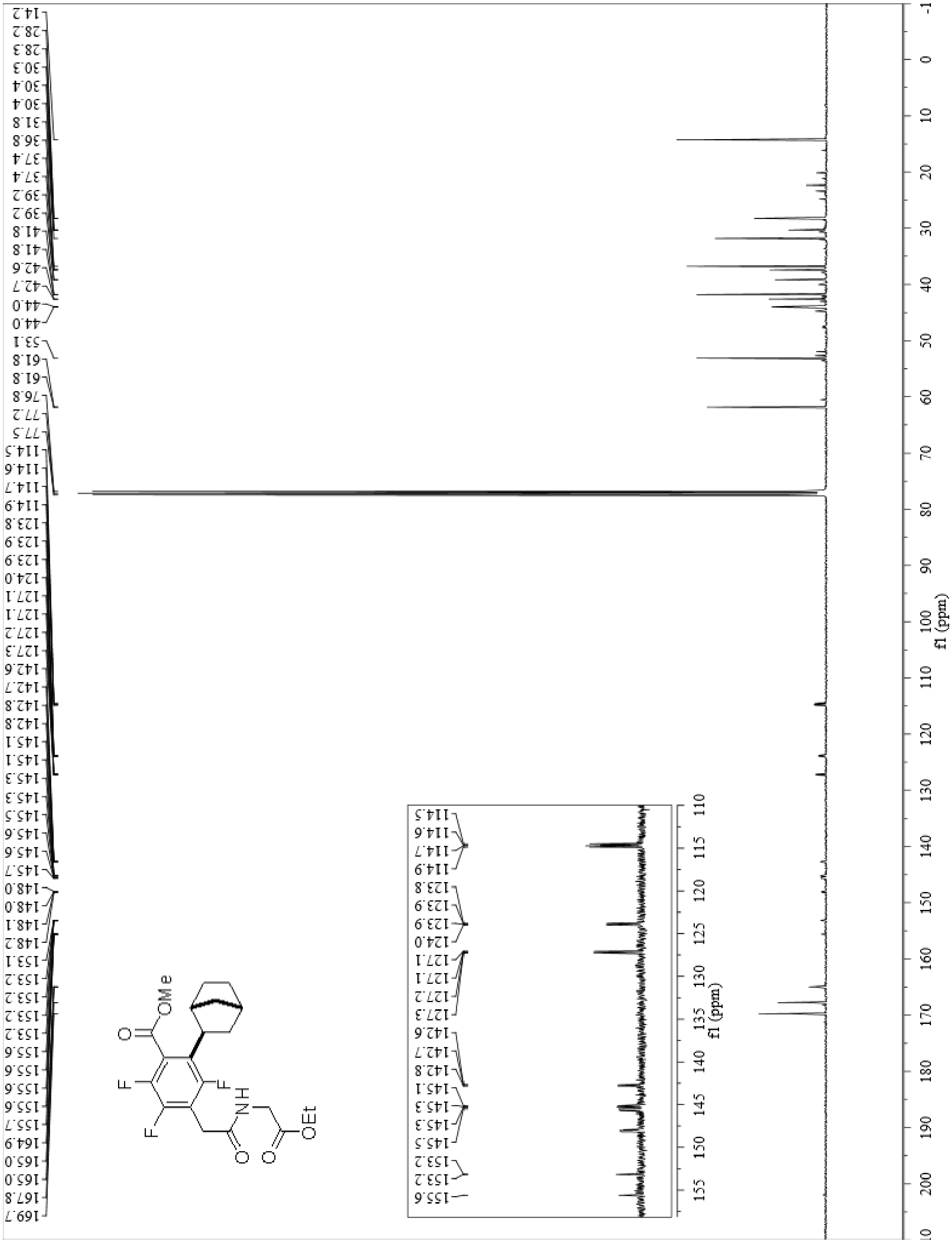

GC and MS of S-26a methyl 2-(bicyclo[2.2.1]heptan-2-yl)-4-(2-((2-ethoxy-2-oxoethyl)amino)-2-oxoethyl)-3,5,6-trifluorobenzoate

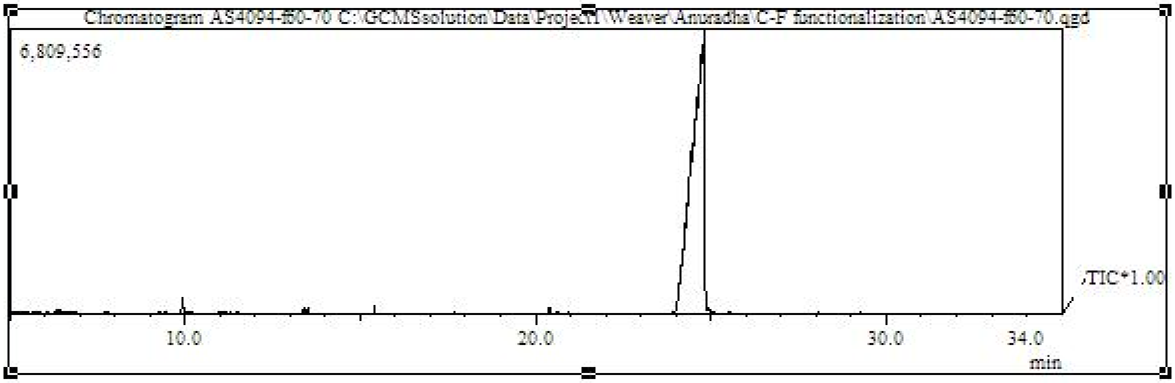

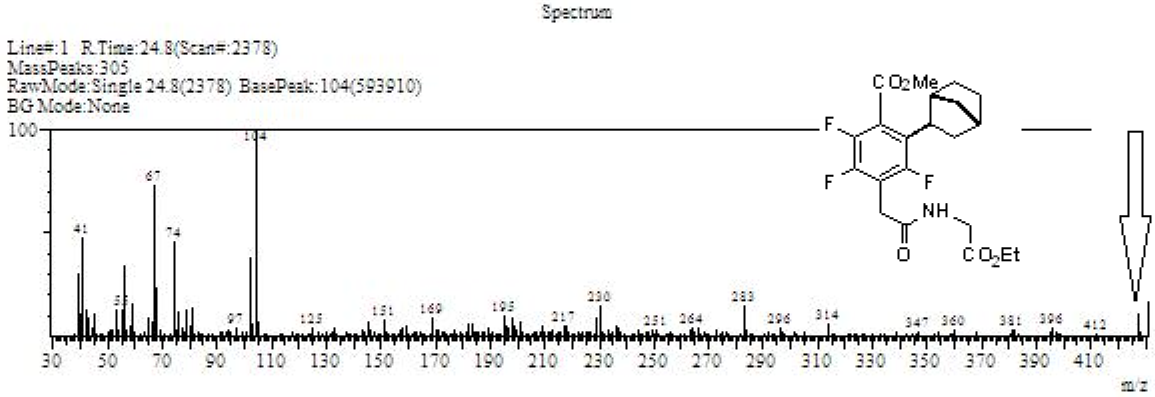

^{19}F NMR (376 MHz, Chloroform-*d*, @ rt) of S-27a ethyl (2-(2-(bicyclo[2.2.1]heptan-2-yl)-3,5,6-trifluoropyridin-4-yl)acetyl)glycinate

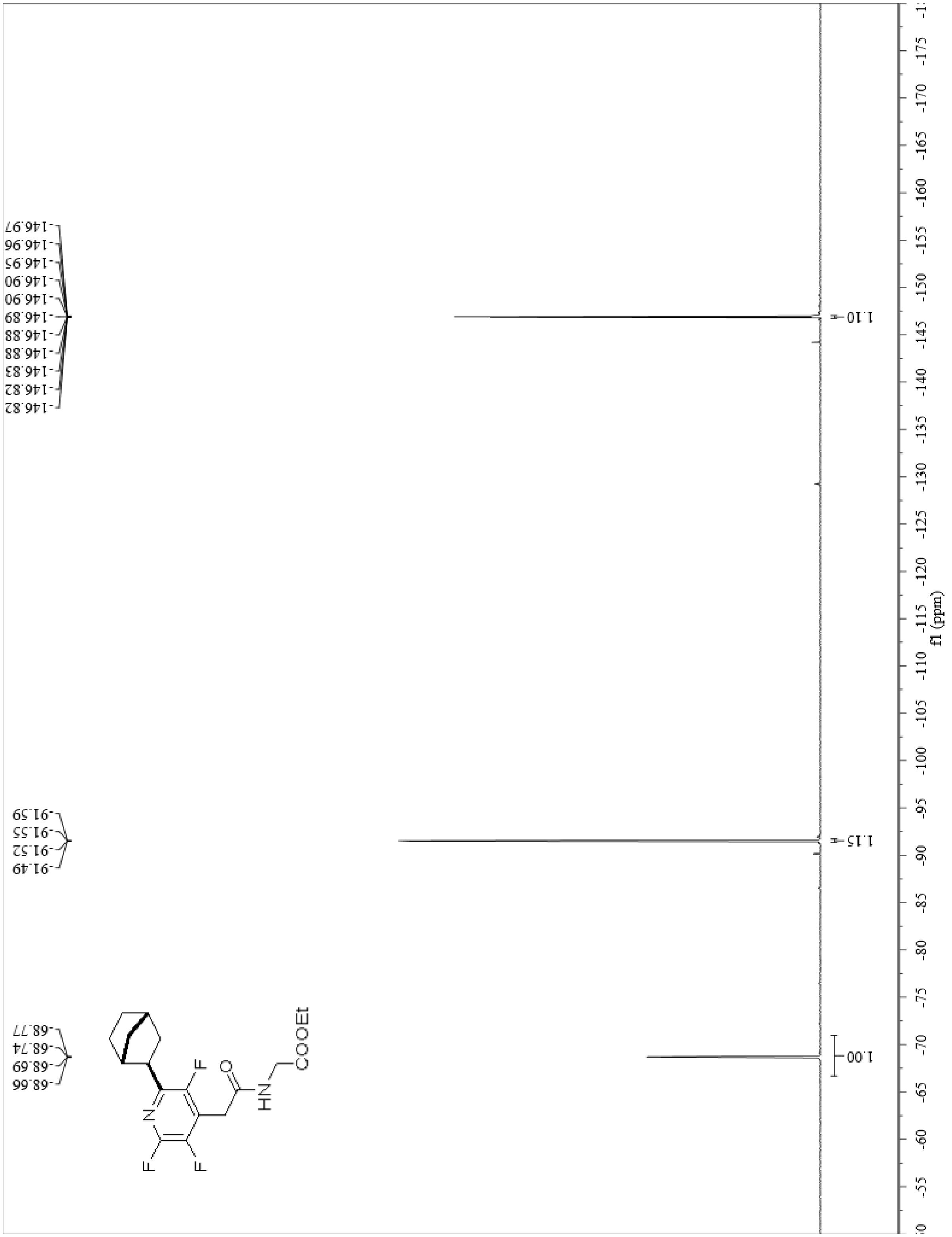

^{1}H NMR (400 MHz, Chloroform-*d*, @ rt) of S-27a ethyl (2-(2-(bicyclo[2.2.1]heptan-2-yl)-3,5,6-trifluoropyridin-4-yl)acetyl)glycinate

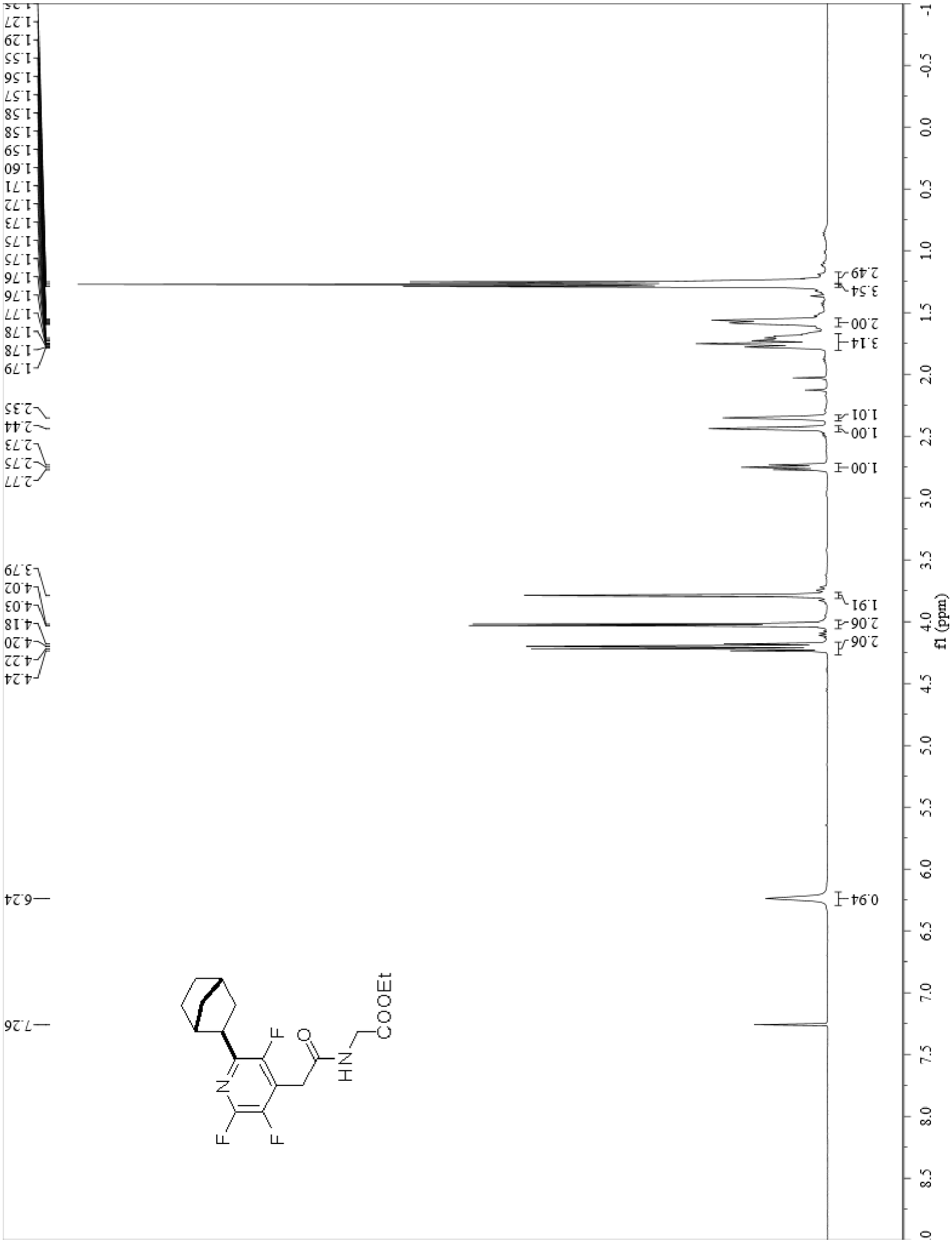

$^{13}C\{^{1}H\}$ NMR (101 MHz, Chloroform-*d*, @ rt) of S-27a ethyl (2-(2-(bicyclo[2.2.1]heptan-2-yl)-3,5,6-trifluoropyridin-4-yl)acetyl)glycinate

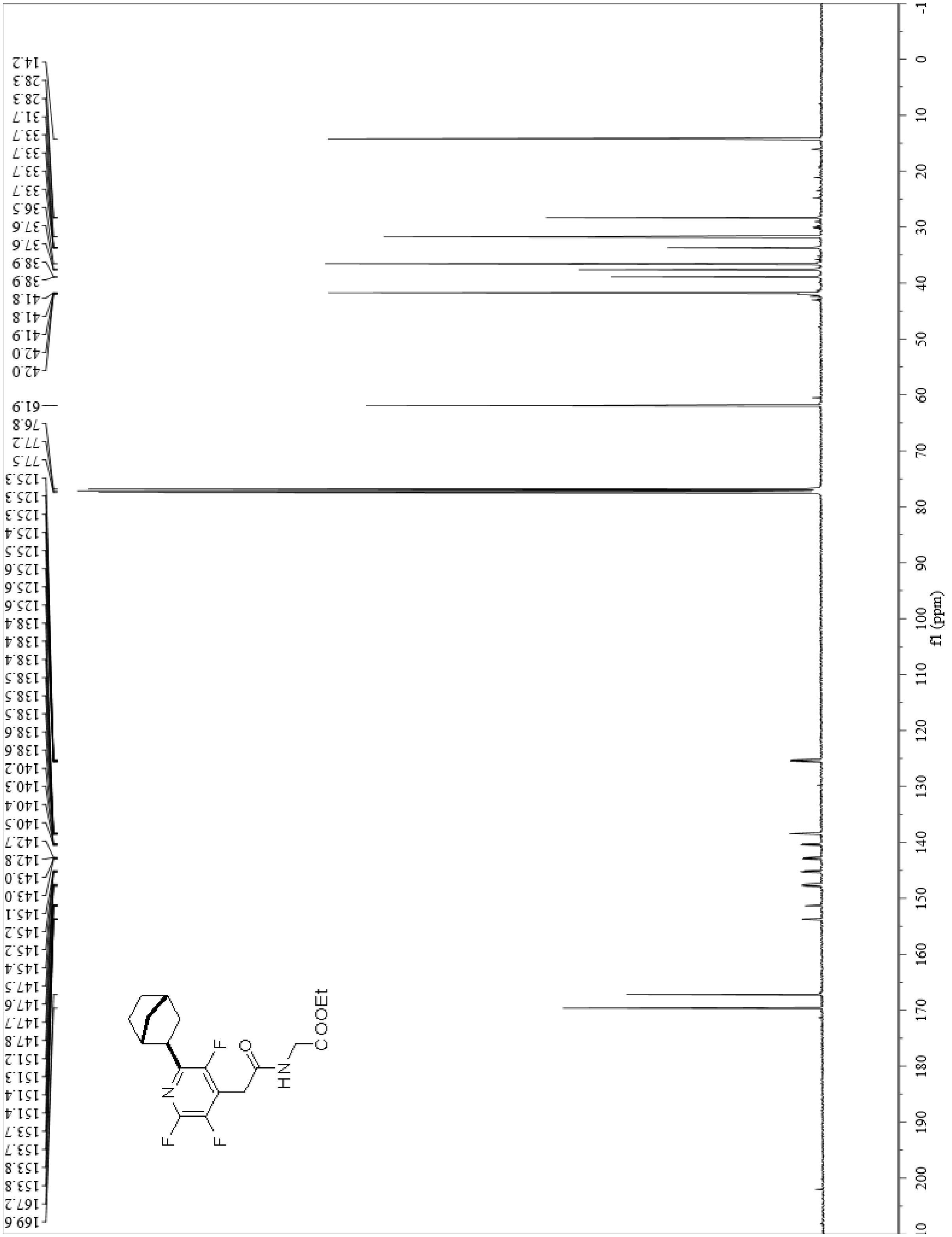

GC and MS of S-27a ethyl (2-(2-(bicyclo[2.2.1]heptan-2-yl)-3,5,6-trifluoropyridin-4-yl)acetyl)glycinate

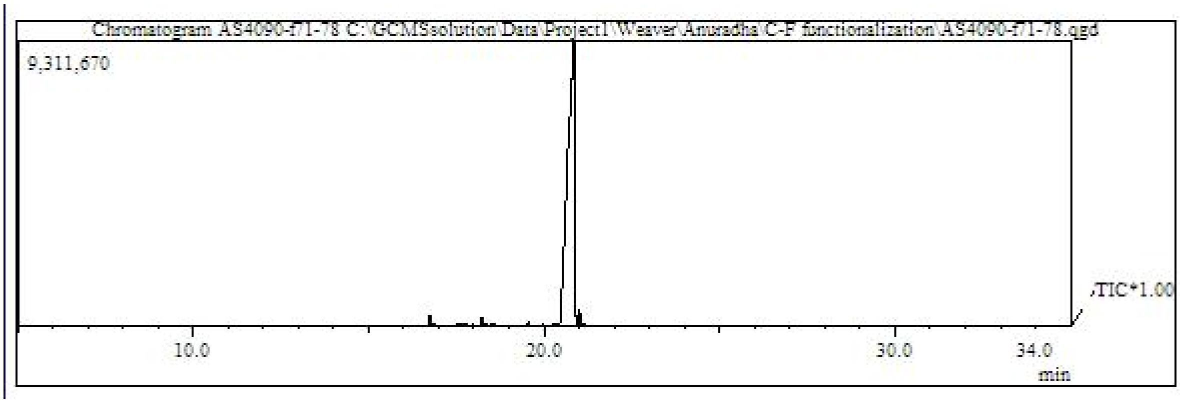

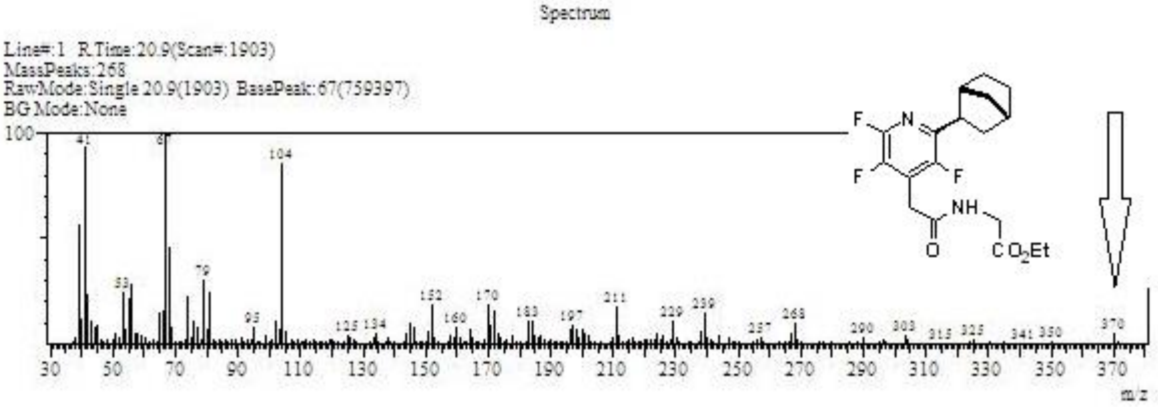

^{19}F NMR (376 MHz, Chloroform-*d*, @ rt) of S-28a dimethyl 5-(4-(2-((2-ethoxy-2-oxoethyl)amino)-2-oxoethyl)-3,5,6-trifluoropyridin-2-yl)-7-oxabicyclo[2.2.1]heptane-2,3-dicarboxylate

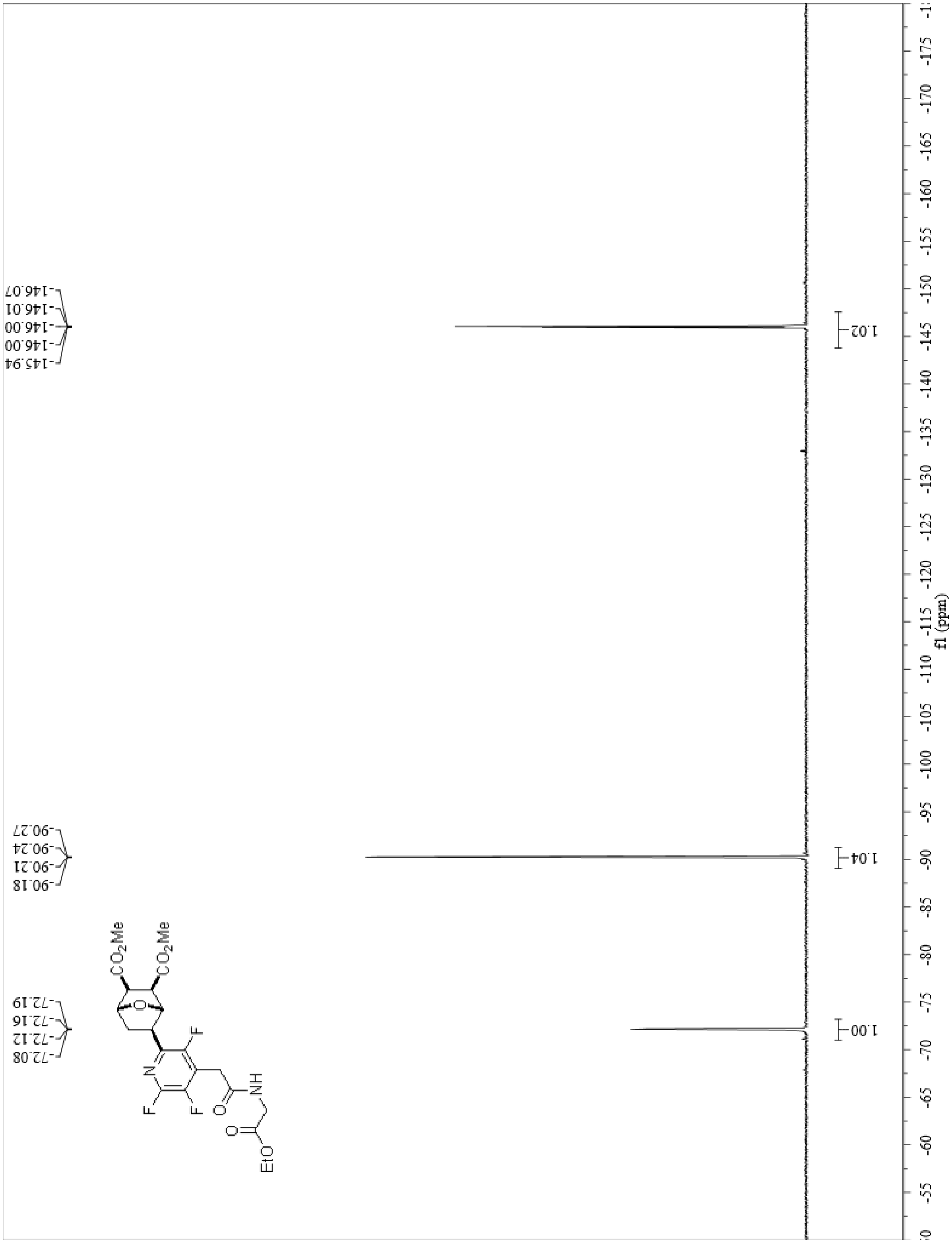

^{1}H NMR (400 MHz, Chloroform-*d*, @ rt) of S-28a dimethyl 5-(4-(2-((2-ethoxy-2-oxoethyl)amino)-2-oxoethyl)-3,5,6-trifluoropyridin-2-yl)-7-oxabicyclo[2.2.1]heptane-2,3-dicarboxylate

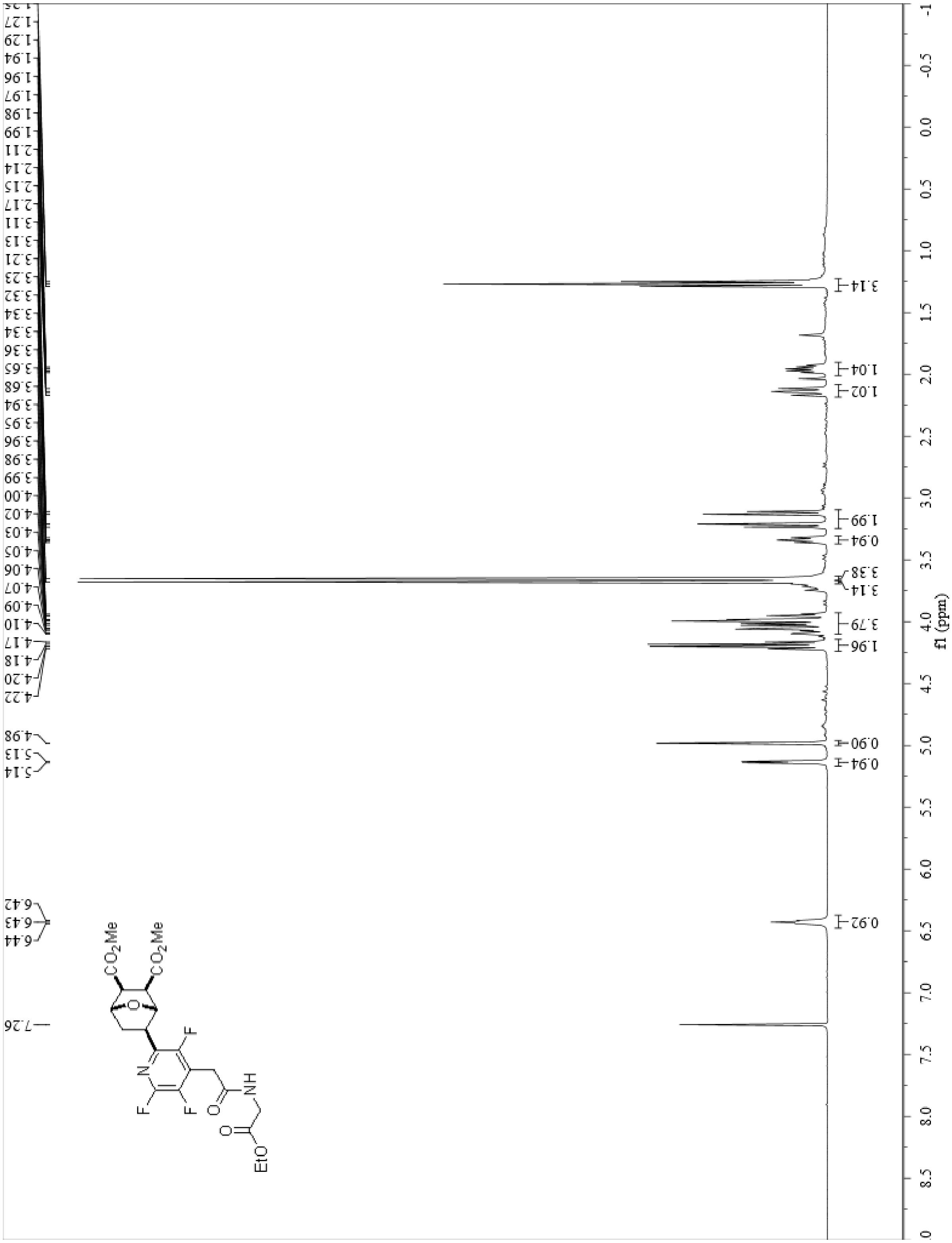

$^{13}C\{^{1}H\}$ NMR (101 MHz, Chloroform-*d*, @ rt) of S-28a dimethyl 5-(4-(2-((2-ethoxy-2-oxoethyl)amino)-2-oxoethyl)-3,5,6-trifluoropyridin-2-yl)-7-oxabicyclo[2.2.1]heptane-2,3-dicarboxylate

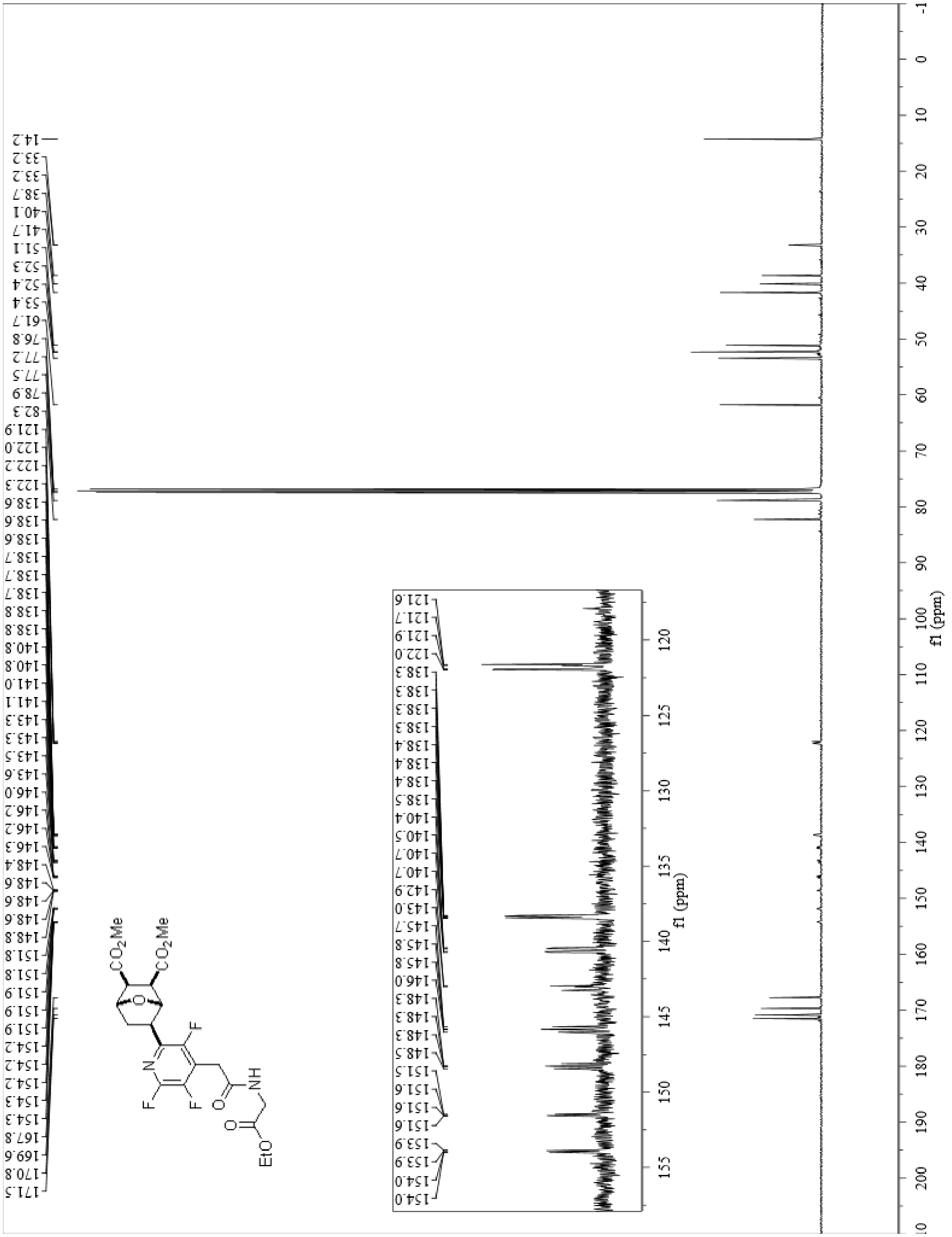

^{19}F NMR (376 MHz, acetone-d_6, @ rt) of S-29a dimethyl 5-(4-(2-((2-ethoxy-2-oxoethyl)amino)-2-oxoethyl)-2,5,6-trifluoropyridin-3-yl)-7-oxabicyclo[2.2.1]heptane-2,3-dicarboxylate

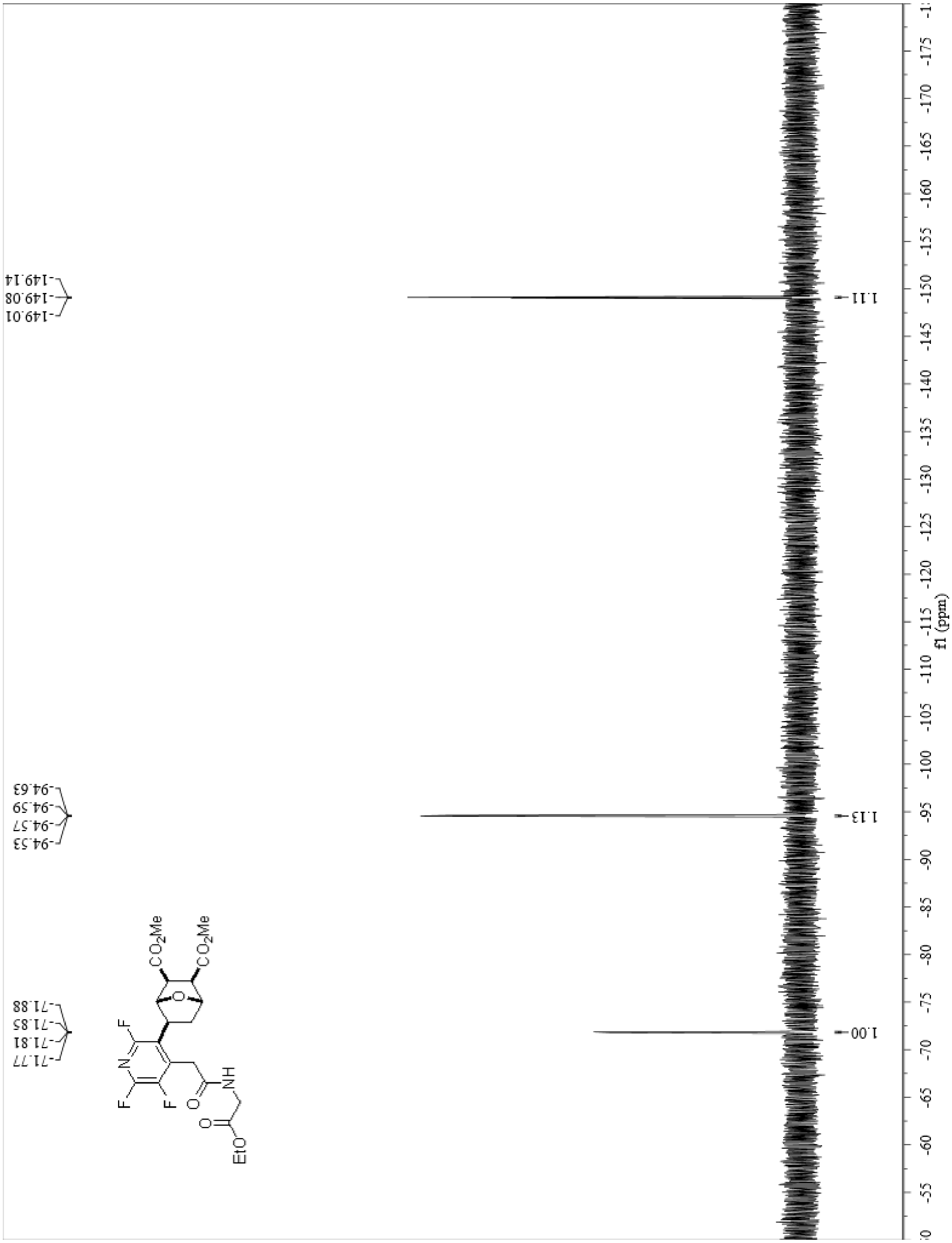

^{1}H NMR (400 MHz, acetone-d_6, @ rt) of S-29a dimethyl 5-(4-(2-((2-ethoxy-2-oxoethyl)amino)-2-oxoethyl)-2,5,6-trifluoropyridin-3-yl)-7-oxabicyclo[2.2.1]heptane-2,3-dicarboxylate

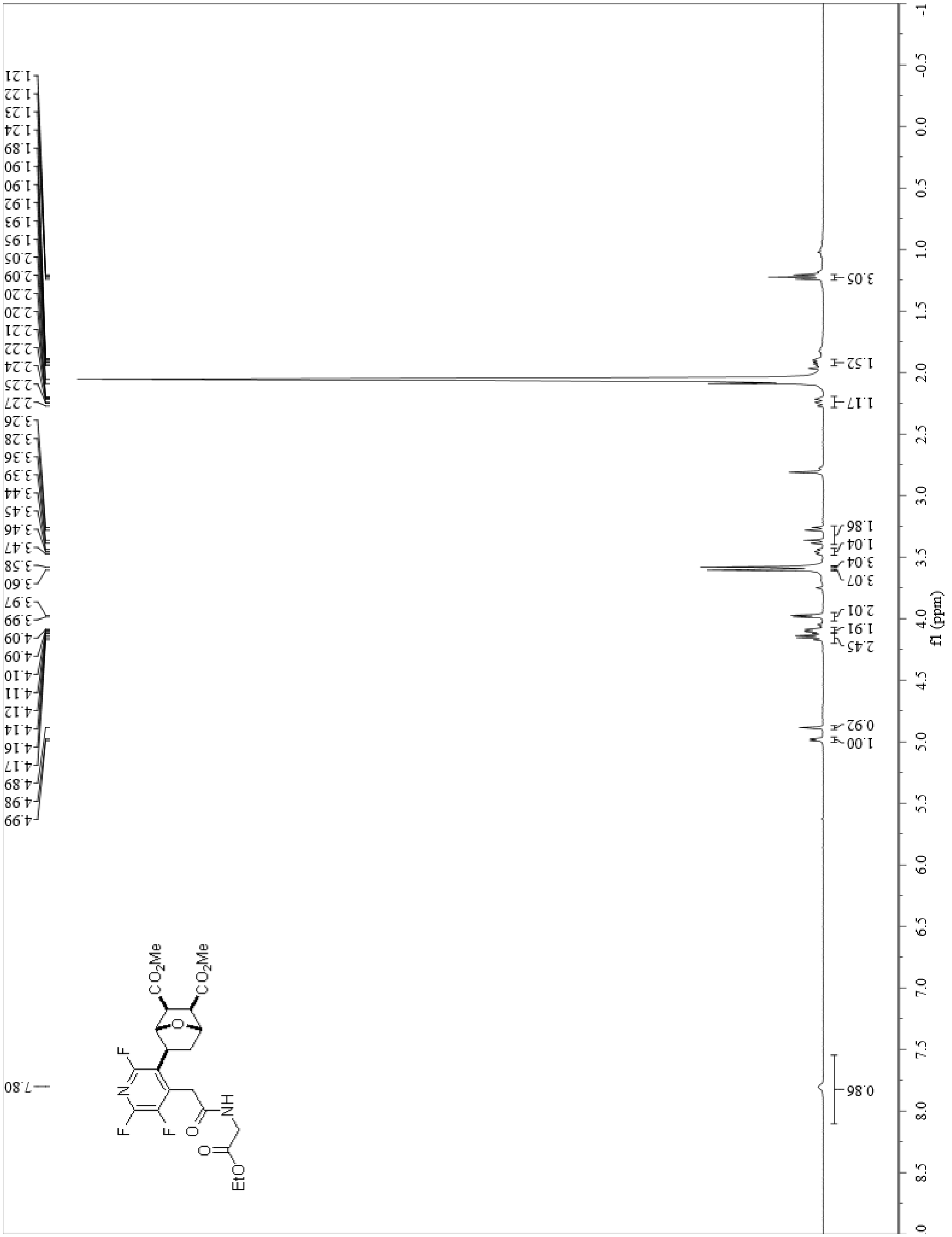

$^{13}C\{^{1}H\}$ NMR (101 MHz, acetone-d_6, @ rt) of S-29a dimethyl 5-(4-(2-((2-ethoxy-2-oxoethyl)amino)-2-oxoethyl)-2,5,6-trifluoropyridin-3-yl)-7-oxabicyclo[2.2.1]heptane-2,3-dicarboxylate

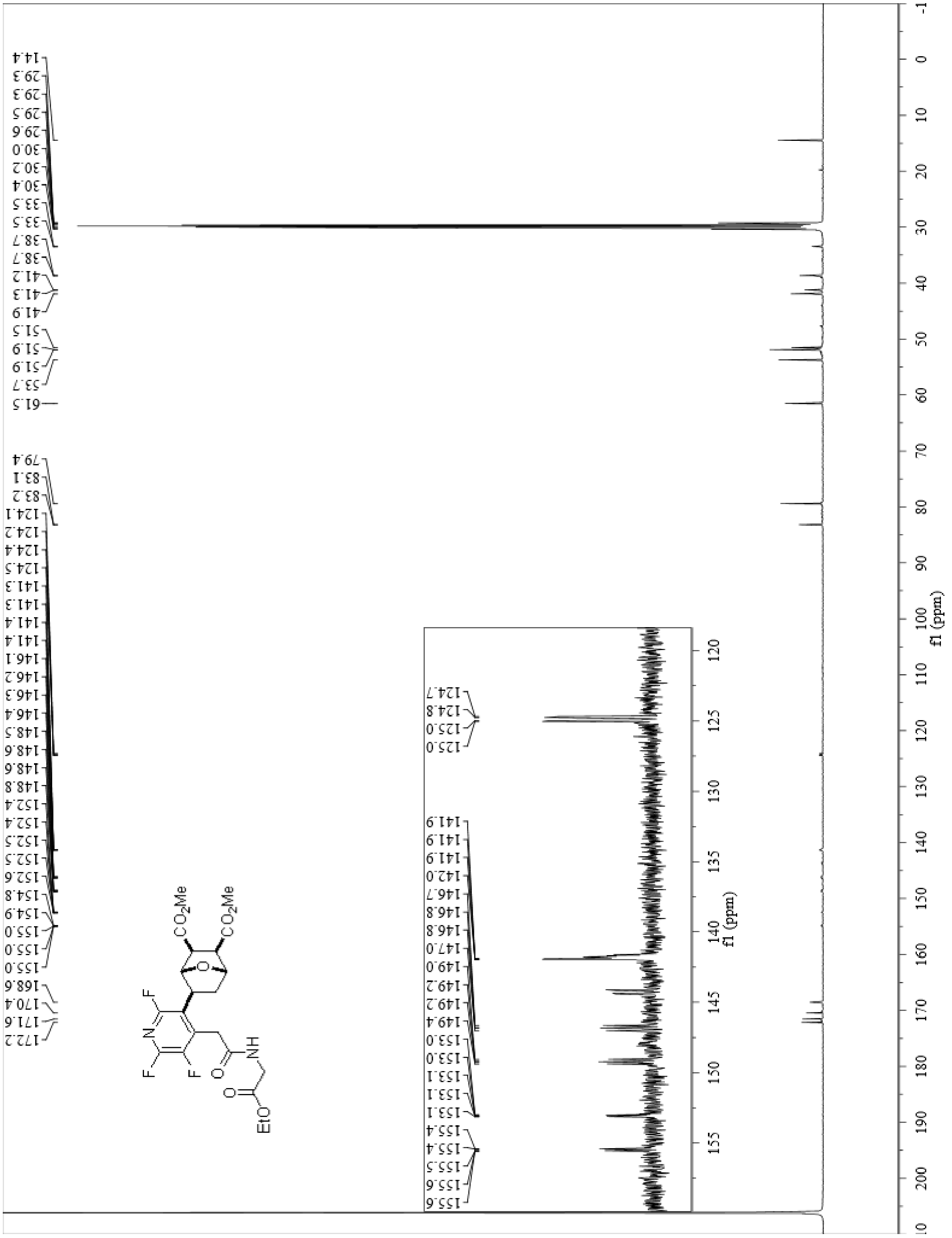

^{19}F NMR (376 MHz, Chloroform-*d*, @ rt) of S-30a (1'R,3'S)-methyl 2,3,5,6-tetrafluoro-3'-isopropyl-6'-methyl-1',2',3',4'-tetrahydro-[1,1'-biphenyl]-4-carboxylate

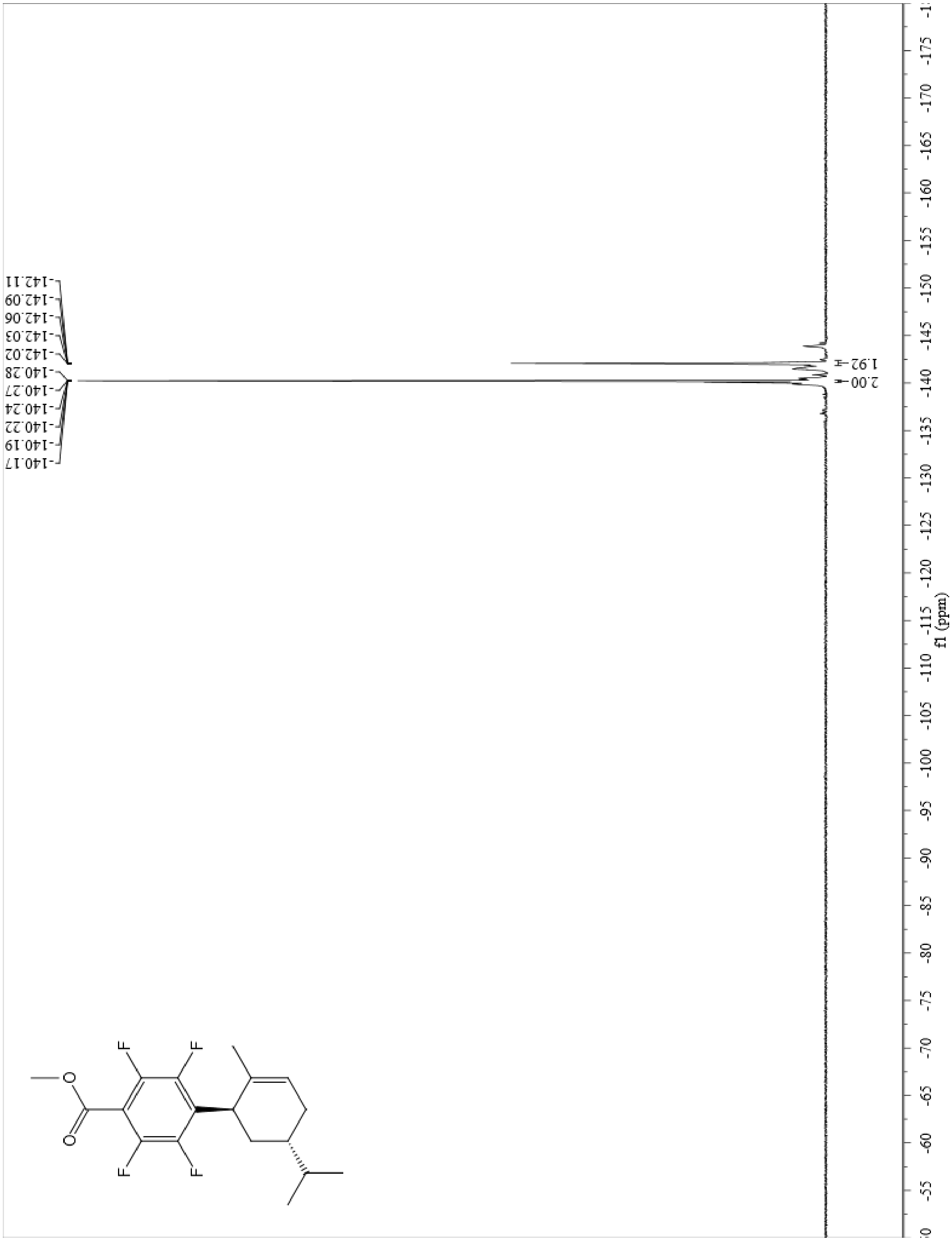

^{1}H NMR (400 MHz, Chloroform-*d*, @ rt) of S-30a (1'R,3'S)-methyl 2,3,5,6-tetrafluoro-3'-isopropyl-6'-methyl-1',2',3',4'-tetrahydro-[1,1'-biphenyl]-4-carboxylate

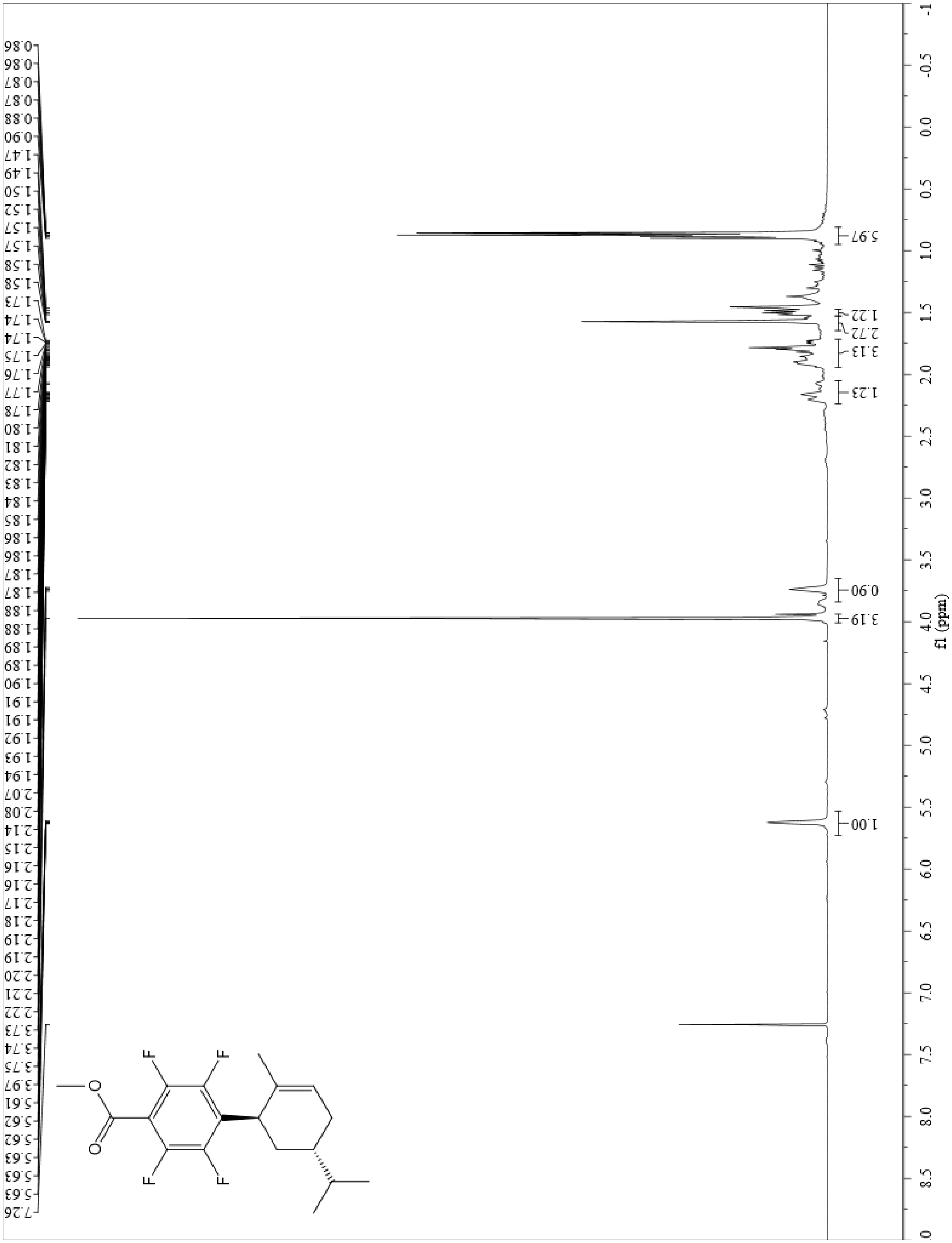

$^{13}C\{^{1}H\}$ NMR (101 MHz, Chloroform-*d*, @ rt) of S-30a (1'R,3'S)-methyl 2,3,5,6-tetrafluoro-3'-isopropyl-6'-methyl-1',2',3',4'-tetrahydro-[1,1'-biphenyl]-4-carboxylate

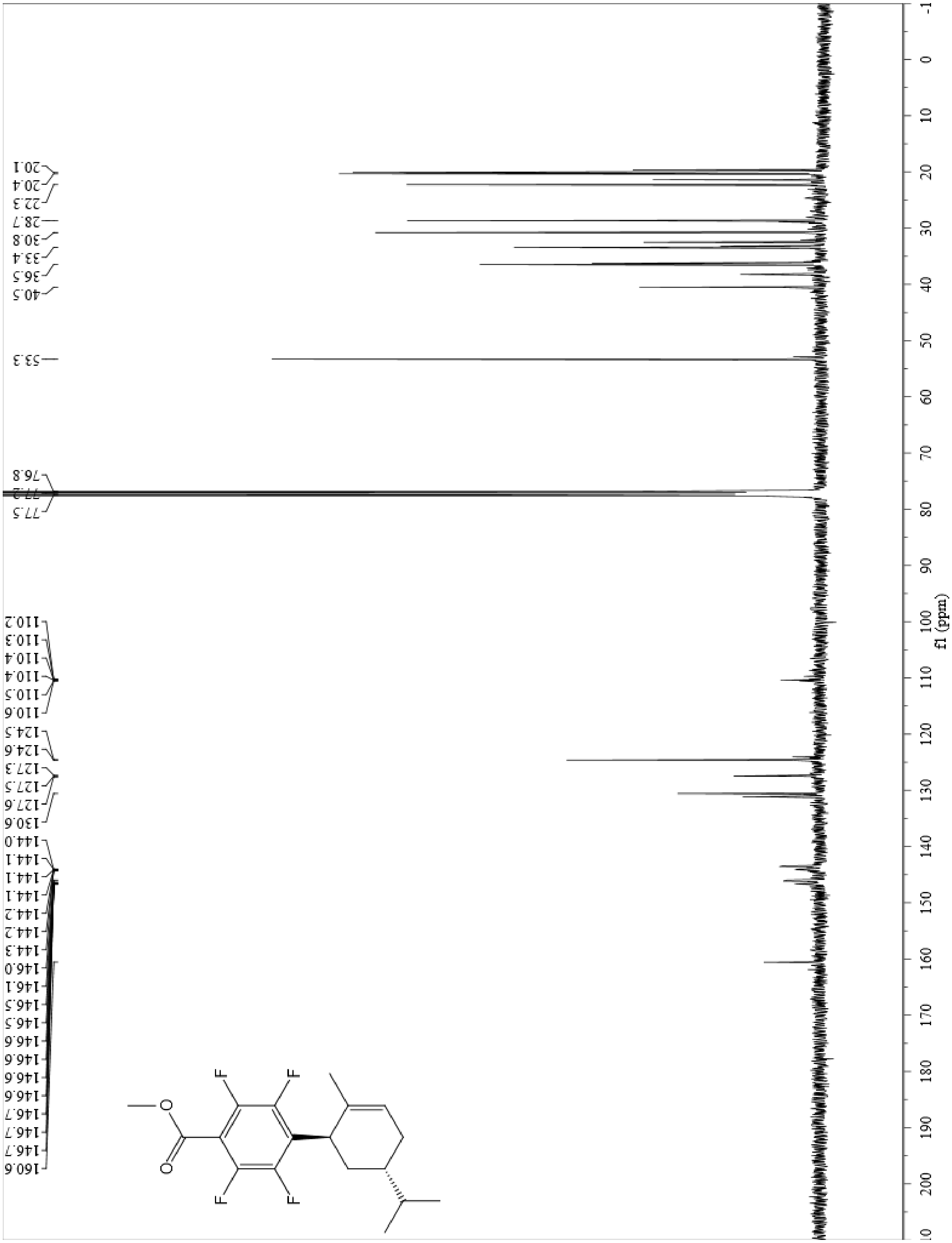

GC and MS of S-30a (1'R,3'S)-methyl 2,3,5,6-tetrafluoro-3'-isopropyl-6'-methyl-1',2',3',4'-tetrahydro-[1,1'-biphenyl]-4-carboxylate

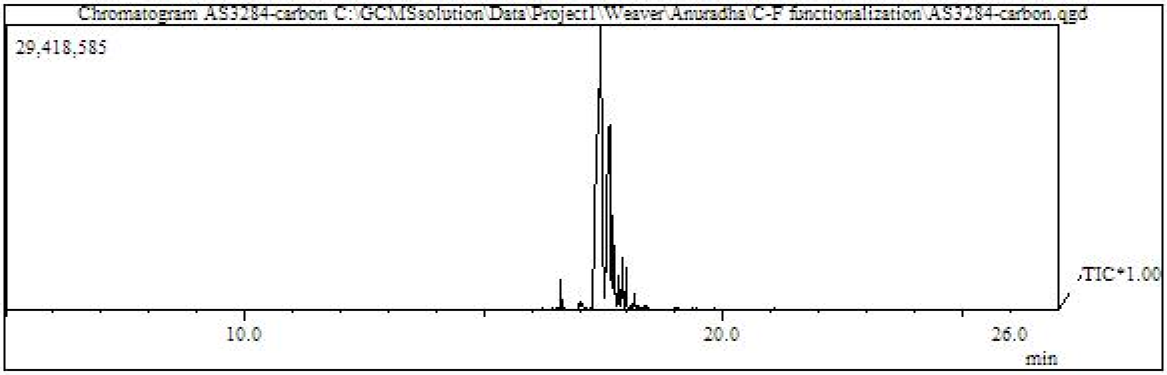

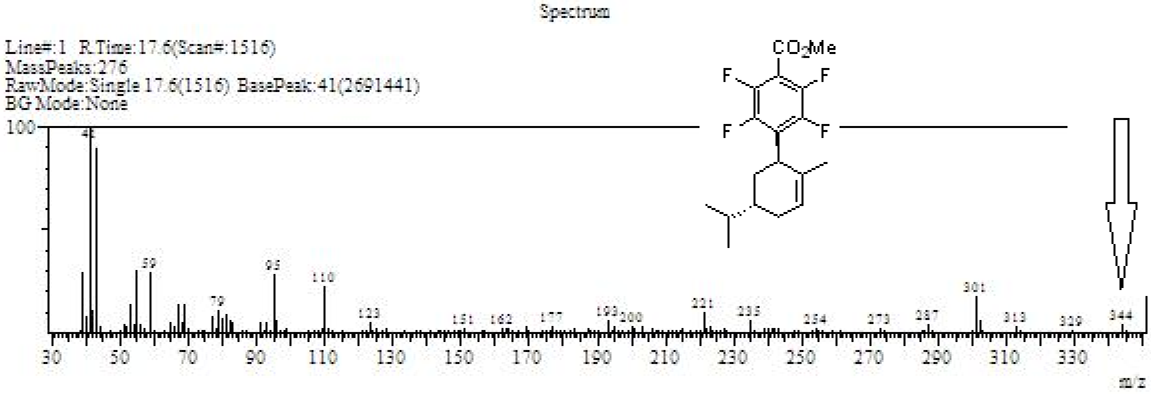

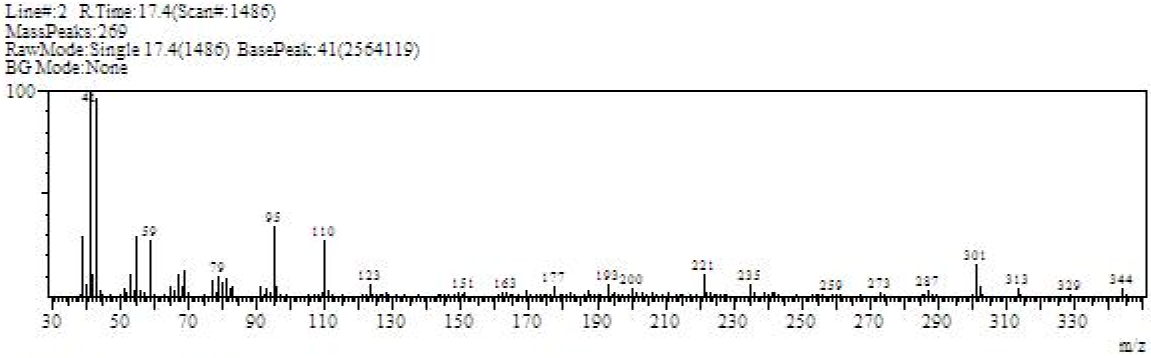

^{19}F NMR (376 MHz, acetone-d_6, @ rt) of S-31a dimethyl 5-(4-(2-((2-ethoxy-2-oxoethyl)amino)-2-oxoethyl)-3,5-difluoropyridin-2-yl)-7-oxabicyclo[2.2.1]heptane-2,3-dicarboxylate

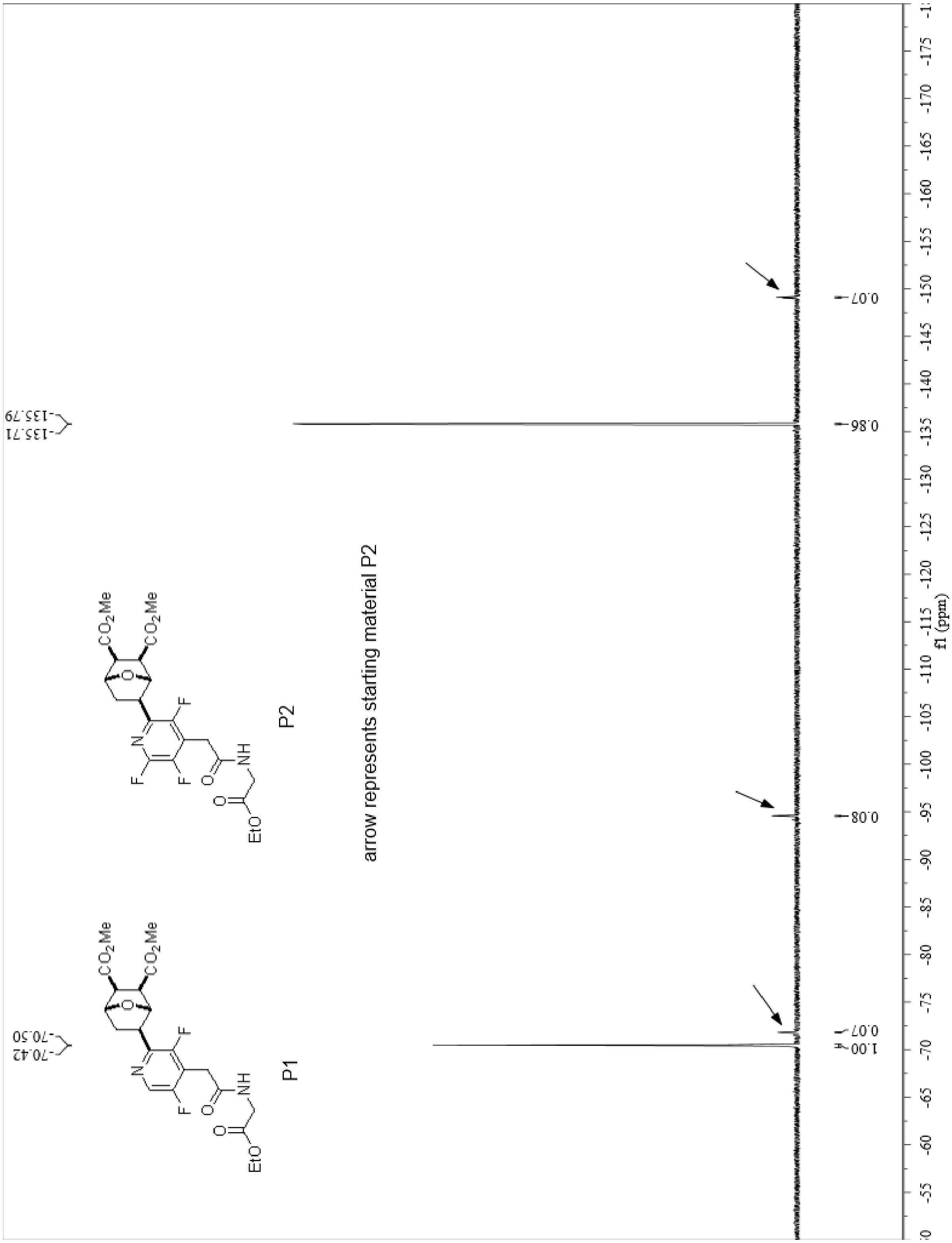

^{1}H NMR (400 MHz, acetone-d_6, @ rt) of S-31a dimethyl 5-(4-(2-((2-ethoxy-2-oxoethyl)amino)-2-oxoethyl)-3,5-difluoropyridin-2-yl)-7-oxabicyclo[2.2.1]heptane-2,3-dicarboxylate

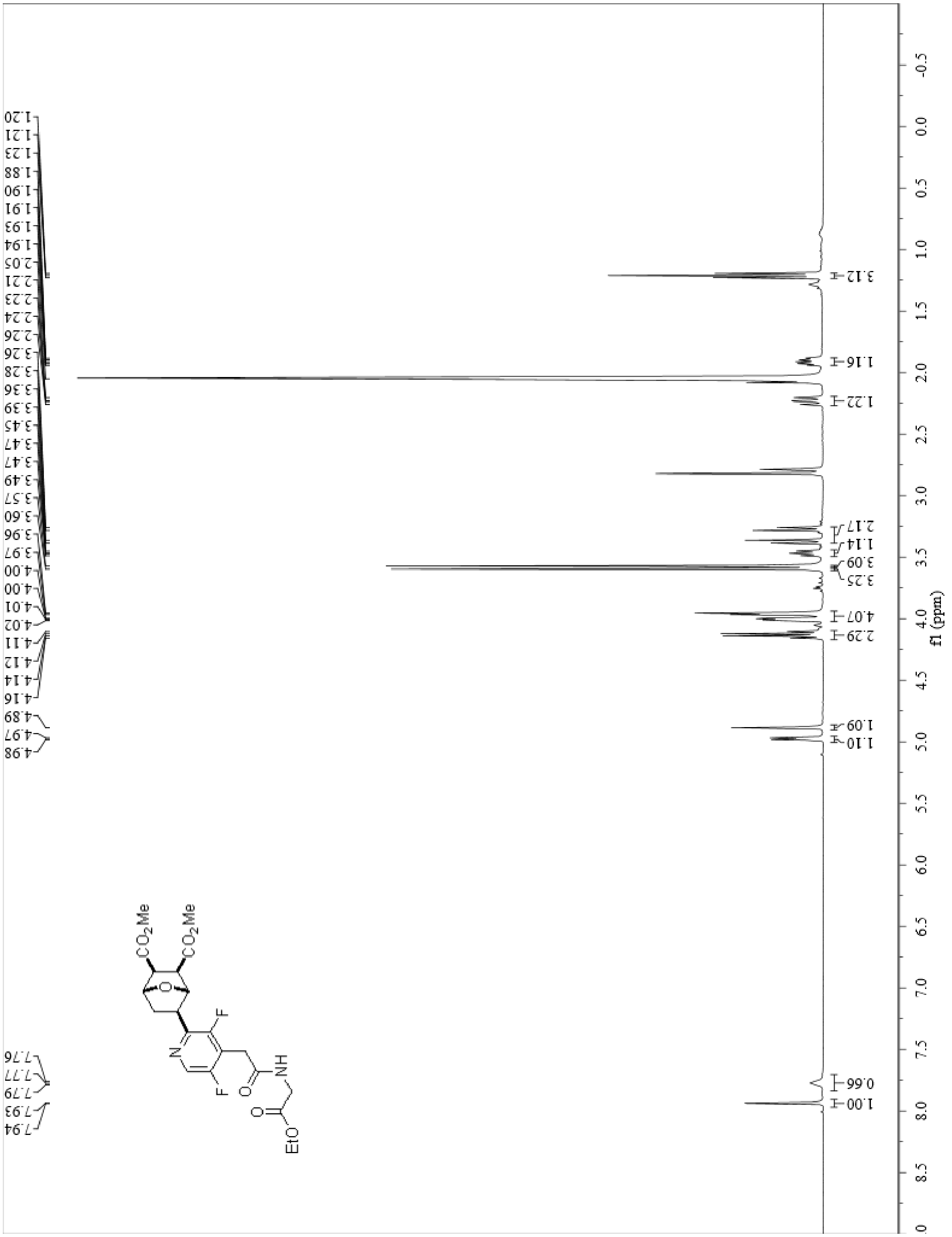

$^{13}C\{^{1}H\}$ NMR (151 MHz, acetone-d_6, @ rt) of S-31a dimethyl 5-(4-(2-((2-ethoxy-2-oxoethyl)amino)-2-oxoethyl)-3,5-difluoropyridin-2-yl)-7-oxabicyclo[2.2.1]heptane-2,3-dicarboxylate

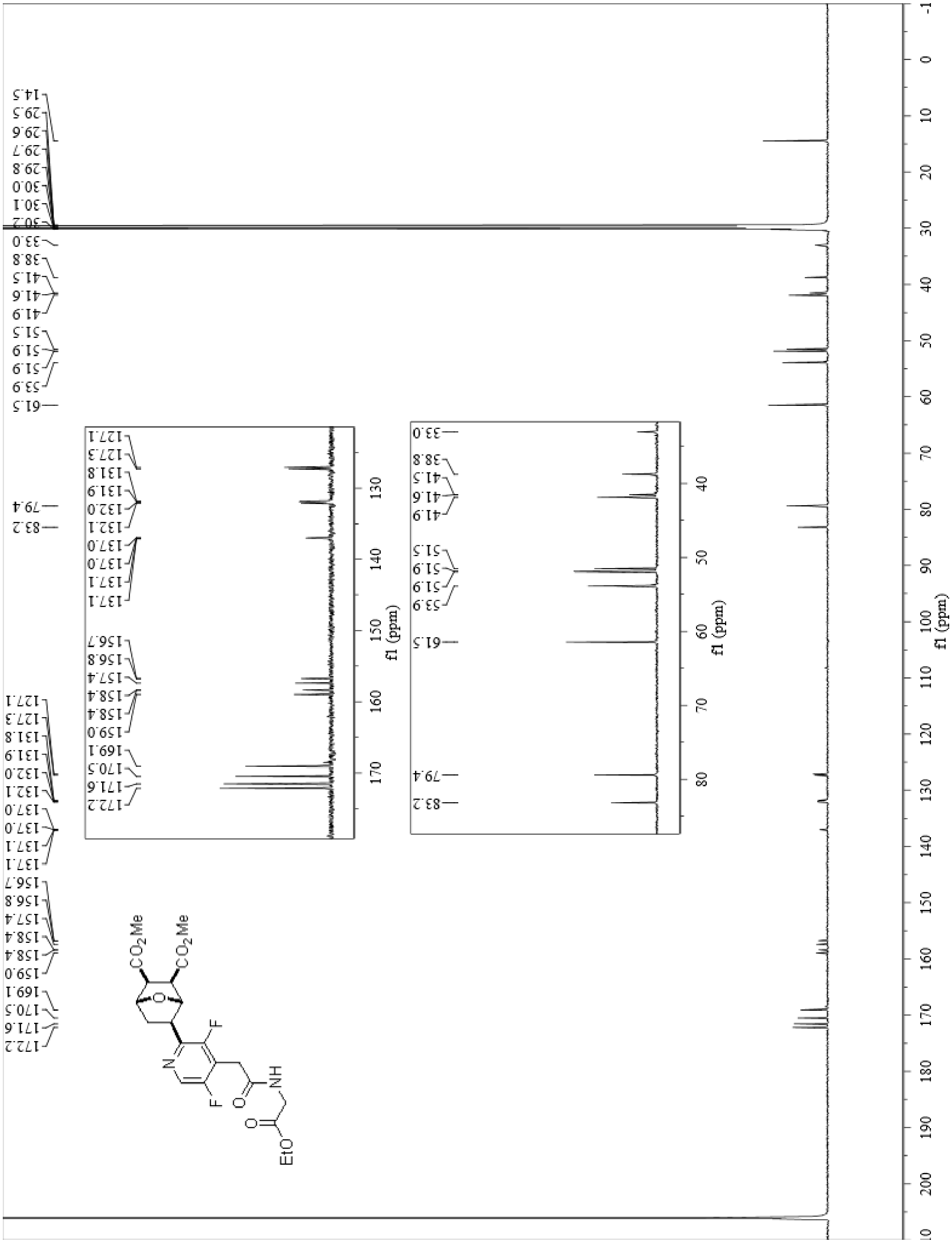

^{19}F NMR (376 MHz, acetone-*d*$_6$, @ rt) of S-32a dimethyl 5-(4-(2-((2-ethoxy-2-oxoethyl)amino)-2-oxoethyl)-2,5-difluoropyridin-3-yl)-7-oxabicyclo[2.2.1]heptane-2,3-dicarboxylate

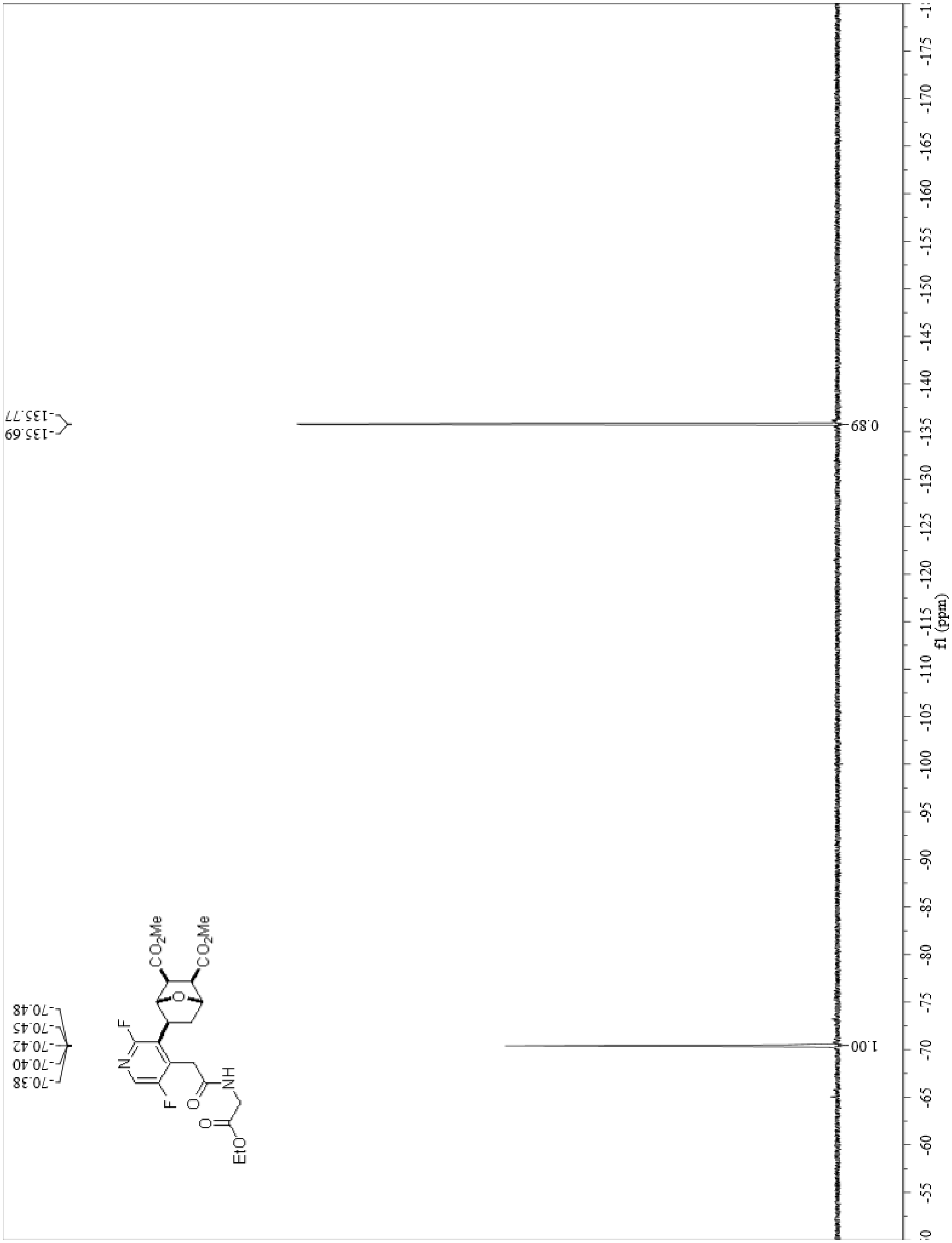

^{1}H NMR (400 MHz, acetone-d_6, @ rt) of S-32a dimethyl 5-(4-(2-((2-ethoxy-2-oxoethyl)amino)-2-oxoethyl)-2,5-difluoropyridin-3-yl)-7-oxabicyclo[2.2.1]heptane-2,3-dicarboxylate

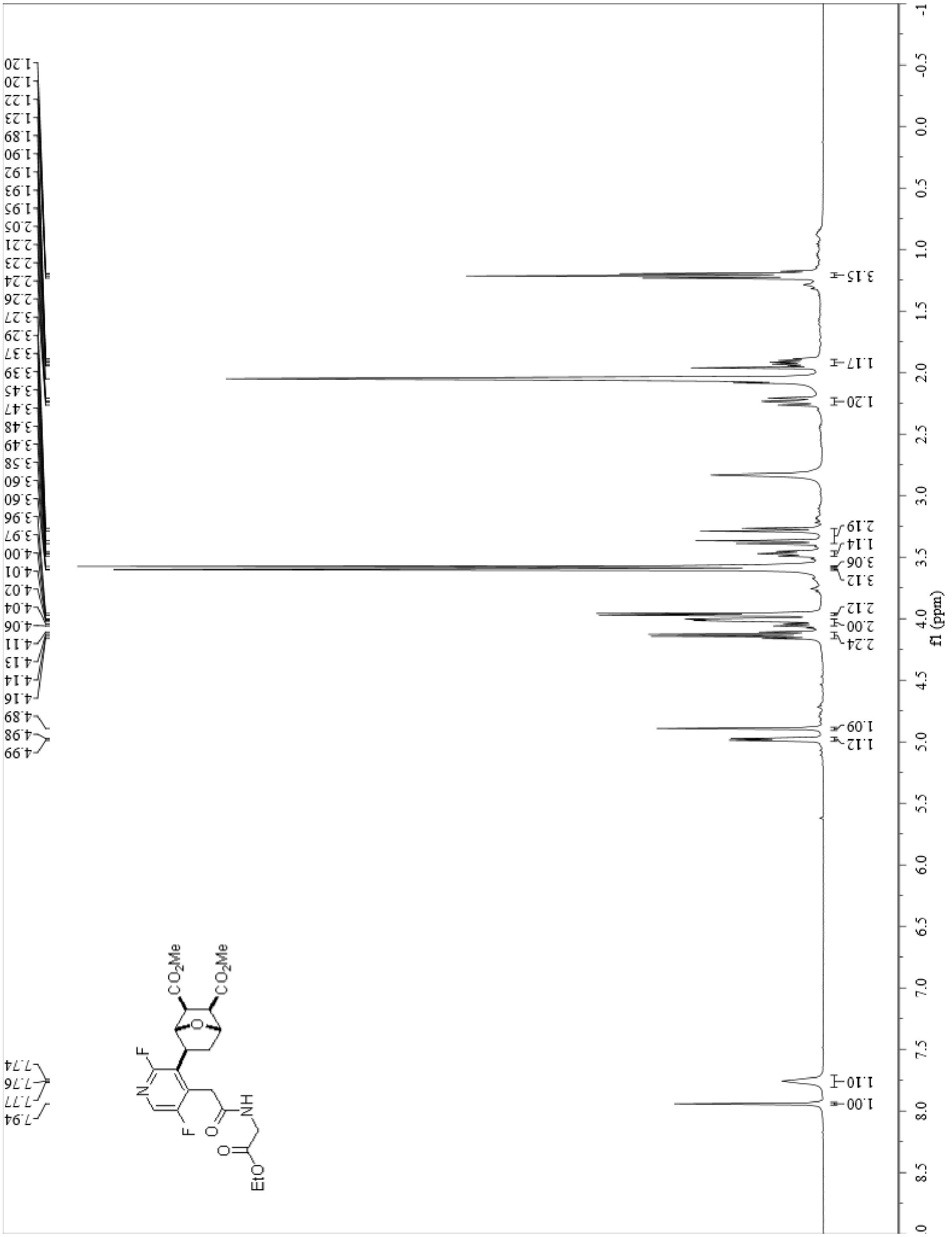

$^{13}C\{^{1}H\}$ NMR (101 MHz, acetone-d_6, @ rt) of S-32a dimethyl 5-(4-(2-((2-ethoxy-2-oxoethyl)amino)-2-oxoethyl)-2,5-difluoropyridin-3-yl)-7-oxabicyclo[2.2.1]heptane-2,3-dicarboxylate

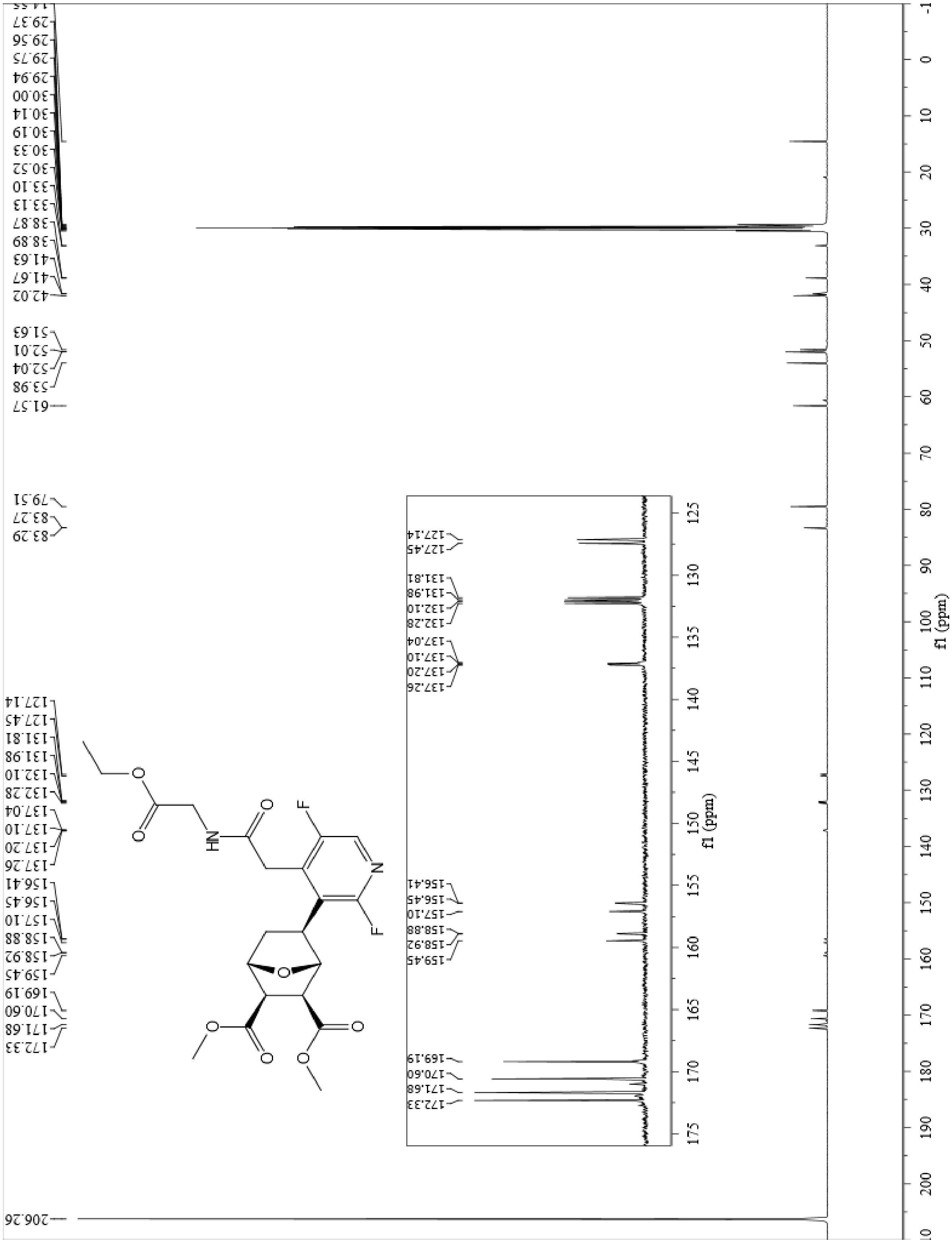

^{19}F NMR (376 MHz, acetone-d_6, @ rt) of S-32a (minor rr) methyl 6-(4-(2-((2-ethoxy-2-oxoethyl)amino)-2-oxoethyl)-2,6-difluoropyridin-3-yl)-3-methyl-7-oxabicyclo[2.2.1]heptane-2-carboxylate

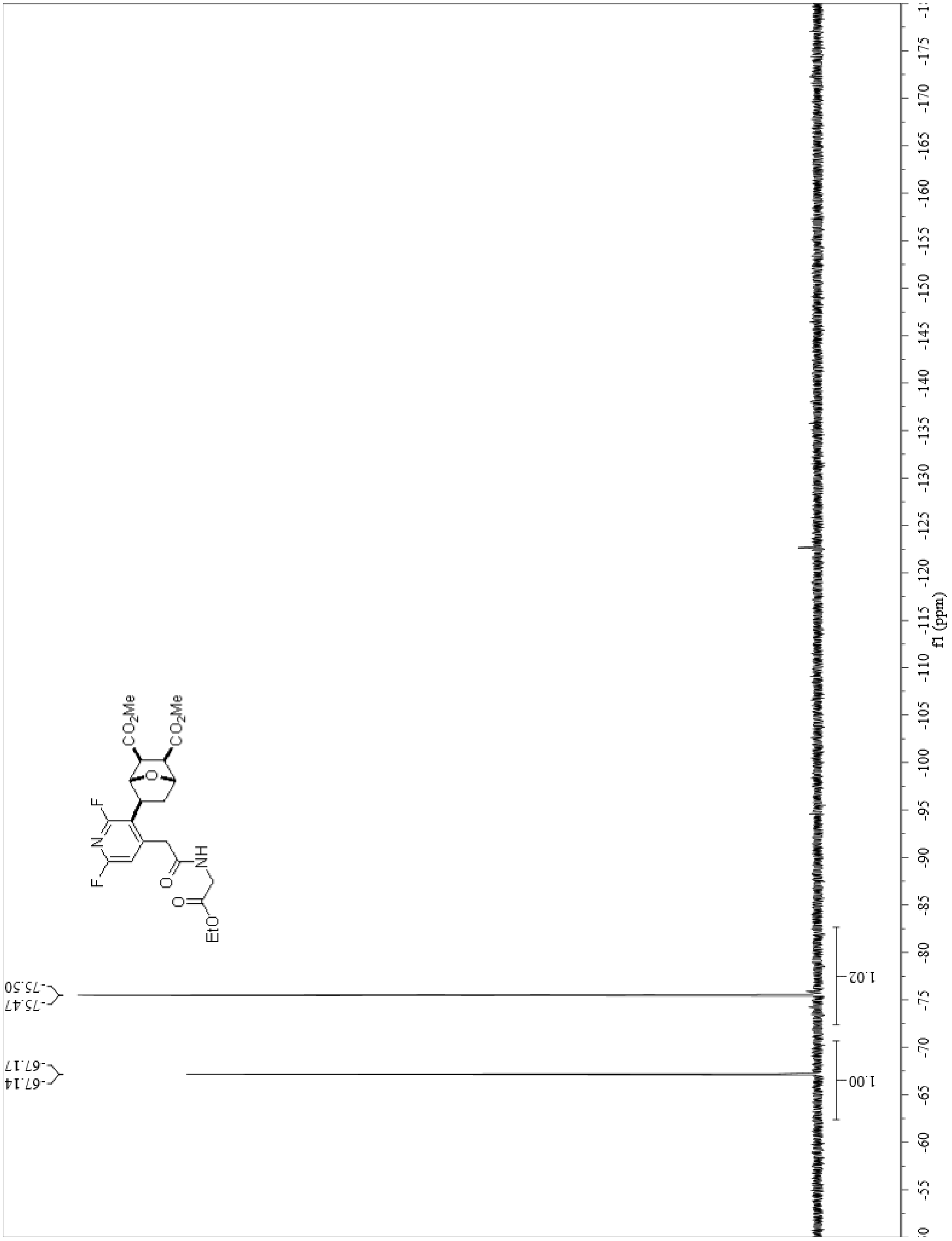

^{1}H NMR (400 MHz, acetone-*d*$_6$, @ rt) of S-32a (minor rr) methyl 6-(4-(2-((2-ethoxy-2-oxoethyl)amino)-2-oxoethyl)-2,6-difluoropyridin-3-yl)-3-methyl-7oxabicyclo[2.2.1]heptane-2-carboxylate

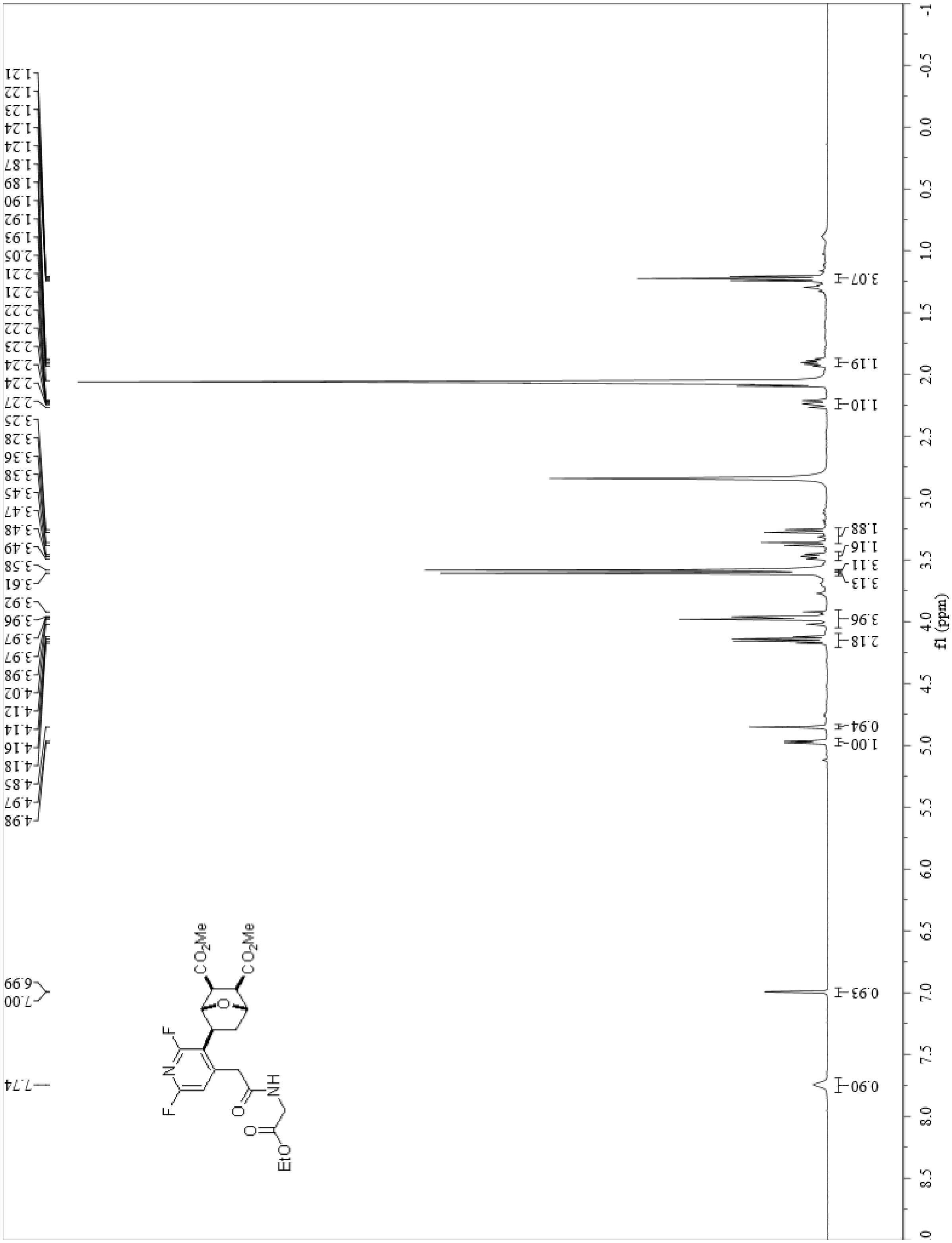

^{19}F NMR (376 MHz, Chloroform-*d*, @ rt) of S-33'a dimethyl 5-(2,5,6-trifluoro-4-(2-methoxy-2-oxoethyl)pyridin-3-yl)-7-oxabicyclo[2.2.1]heptane-2,3-dicarboxylate)

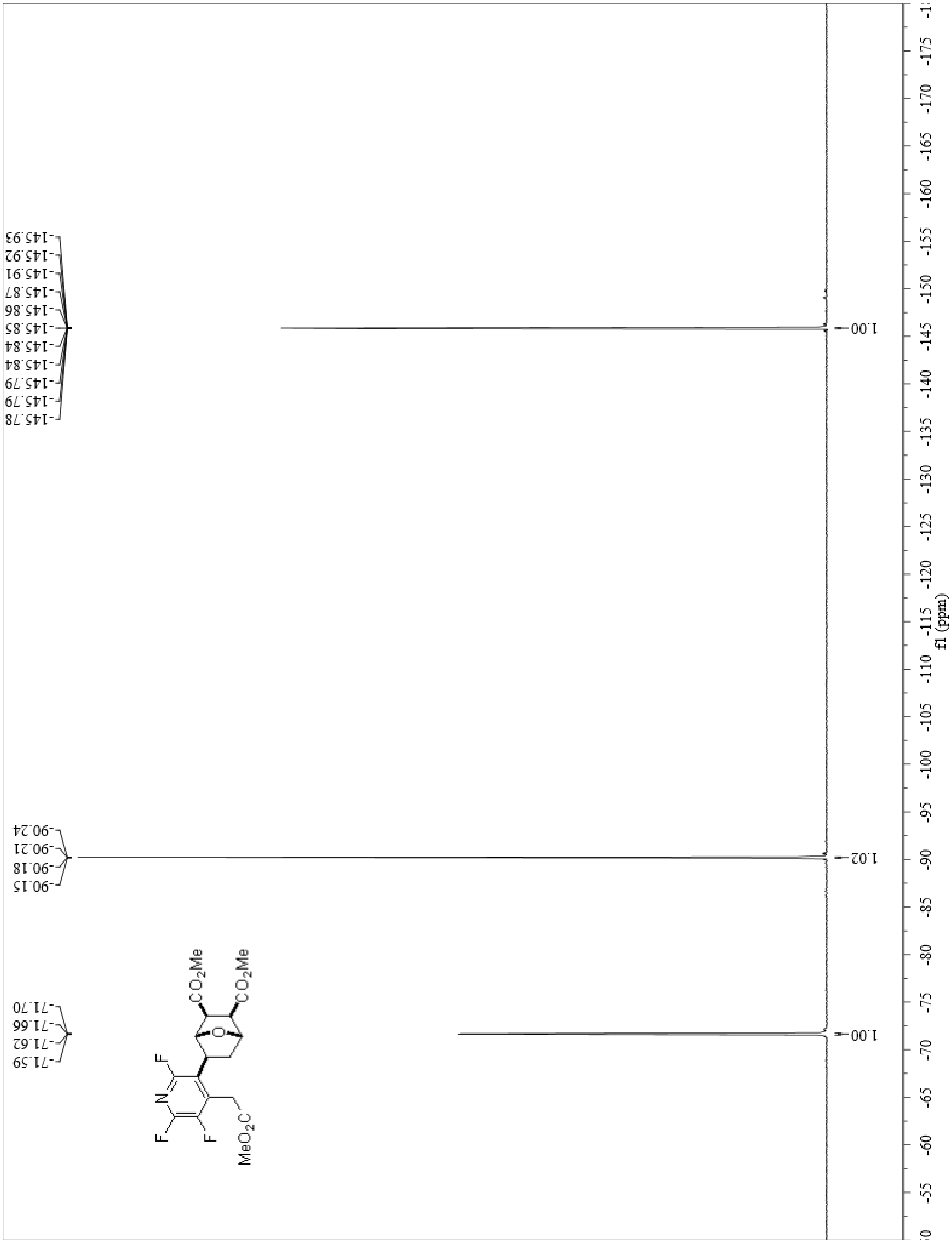

^{1}H NMR (400 MHz, Chloroform-*d*, @ rt) of S-33'a dimethyl 5-(2,5,6-trifluoro-4-(2-methoxy-2-oxoethyl)pyridin-3-yl)-7-oxabicyclo[2.2.1]heptane-2,3-dicarboxylate)

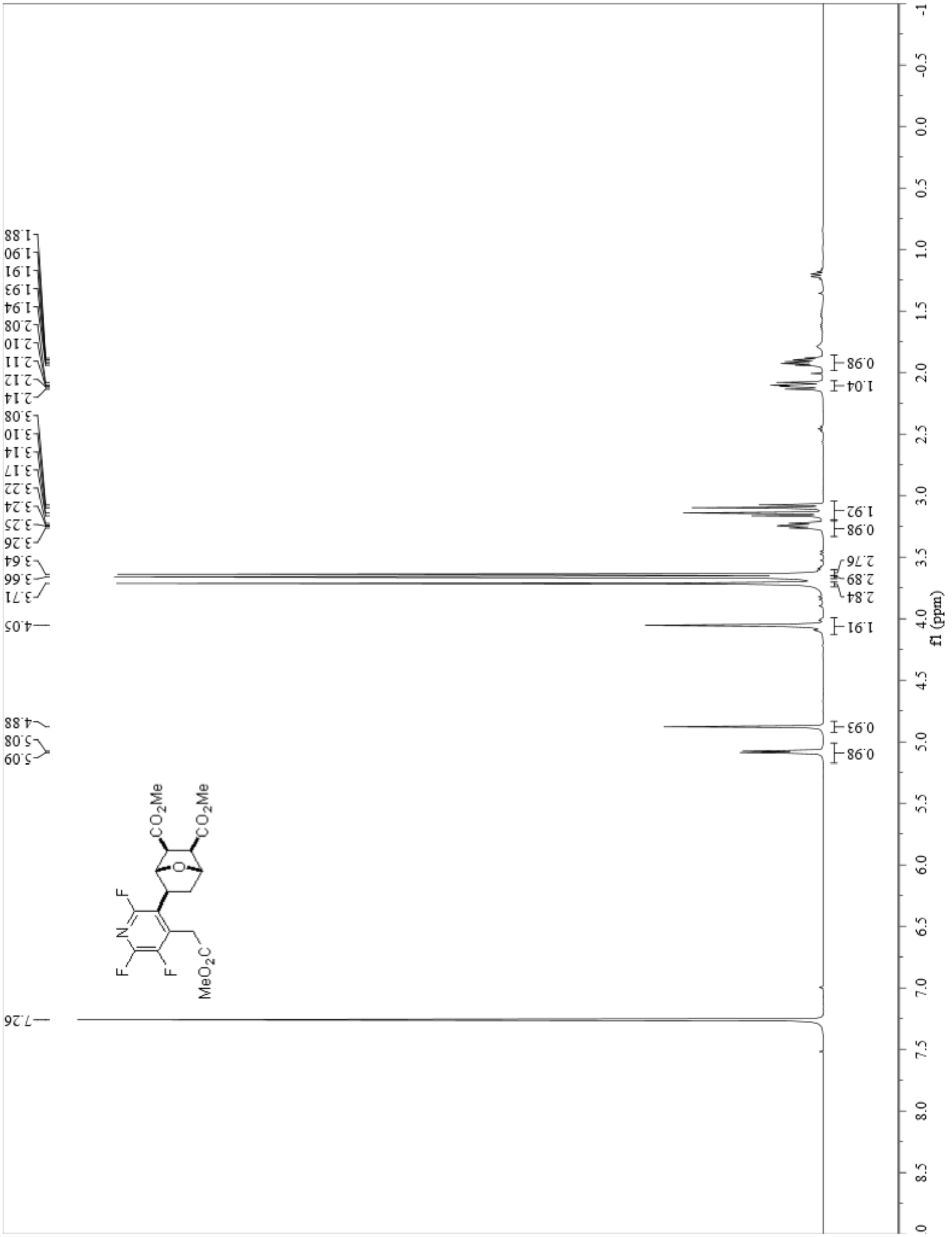

$^{13}C\{^{1}H\}$ NMR (101 MHz, Chloroform-*d*, @ rt) of S-33'a dimethyl 5-(2,5,6-trifluoro-4-(2-methoxy-2-oxoethyl)pyridin-3-yl)-7-oxabicyclo[2.2.1]heptane-2,3-dicarboxylate)

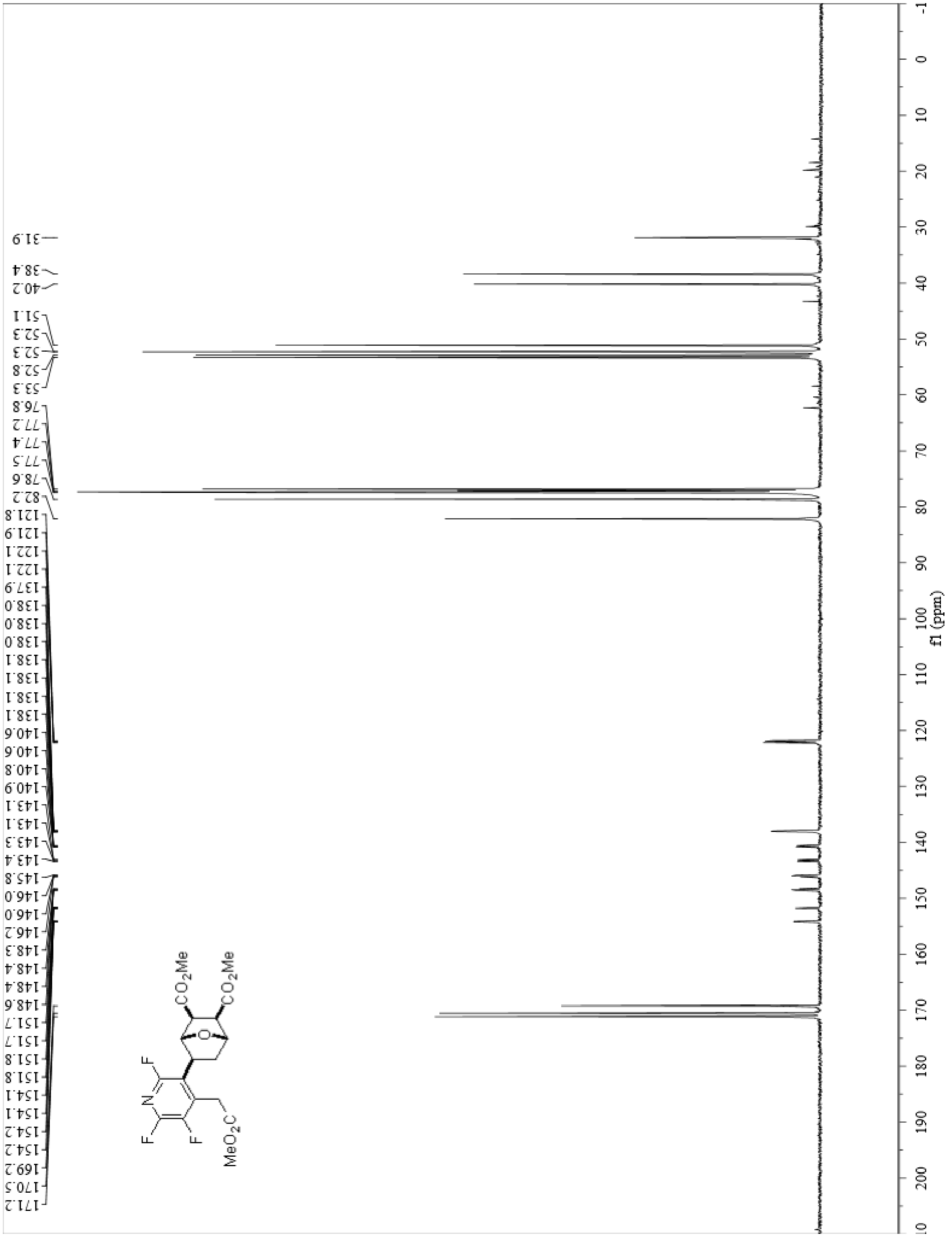

^{19}F NMR (376 MHz, Chloroform-*d*, @ rt) of S-33a dimethyl 5-(2,5-difluoro-4-(2-methoxy-2-oxoethyl)pyridin-3-yl)-7-oxabicyclo[2.2.1]heptane-2,3-dicarboxylate

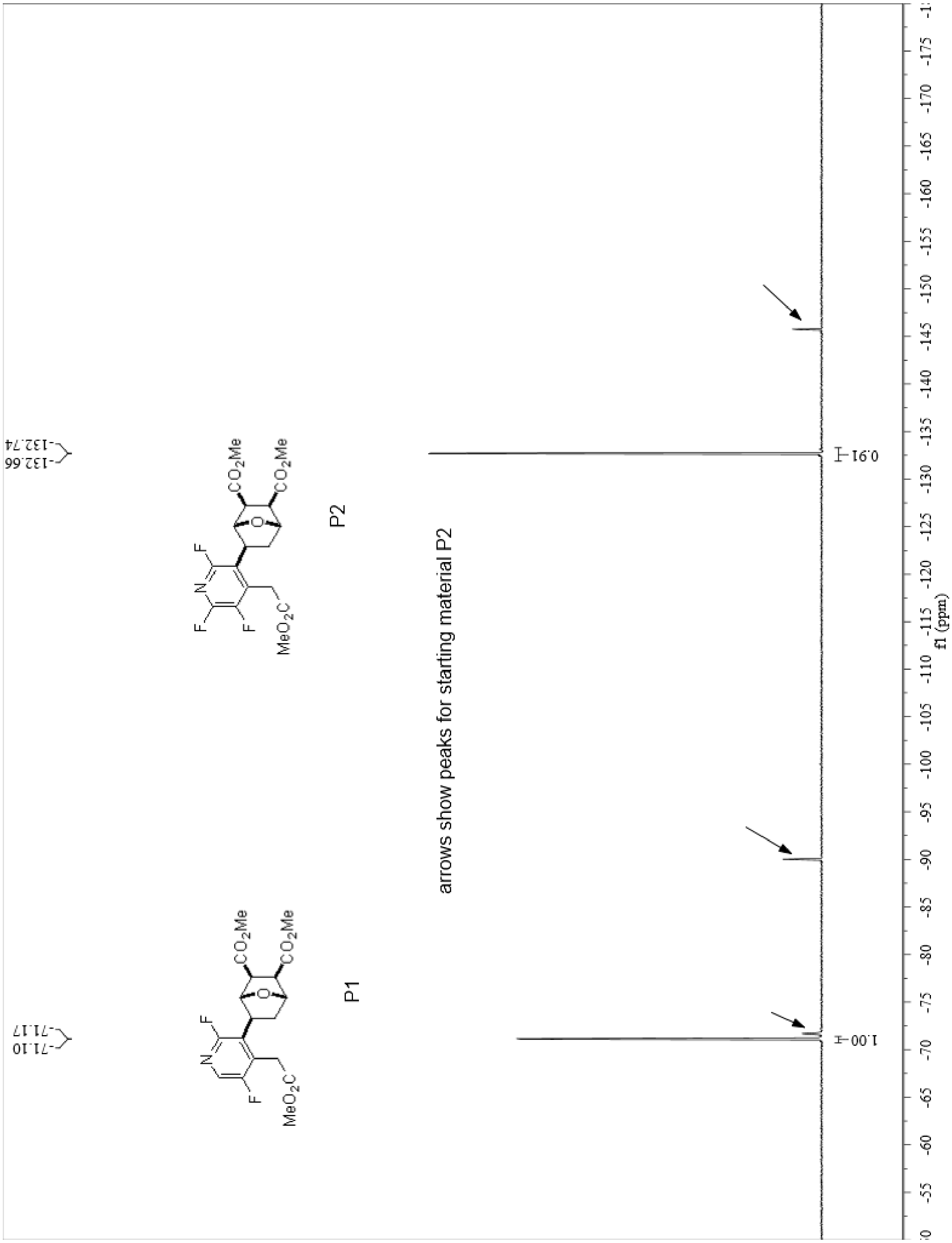

^{1}H NMR (400 MHz, Chloroform-*d*, @ rt) of S-33a dimethyl 5-(2,5-difluoro-4-(2-methoxy-2-oxoethyl)pyridin-3-yl)-7-oxabicyclo[2.2.1]heptane-2,3-dicarboxylate

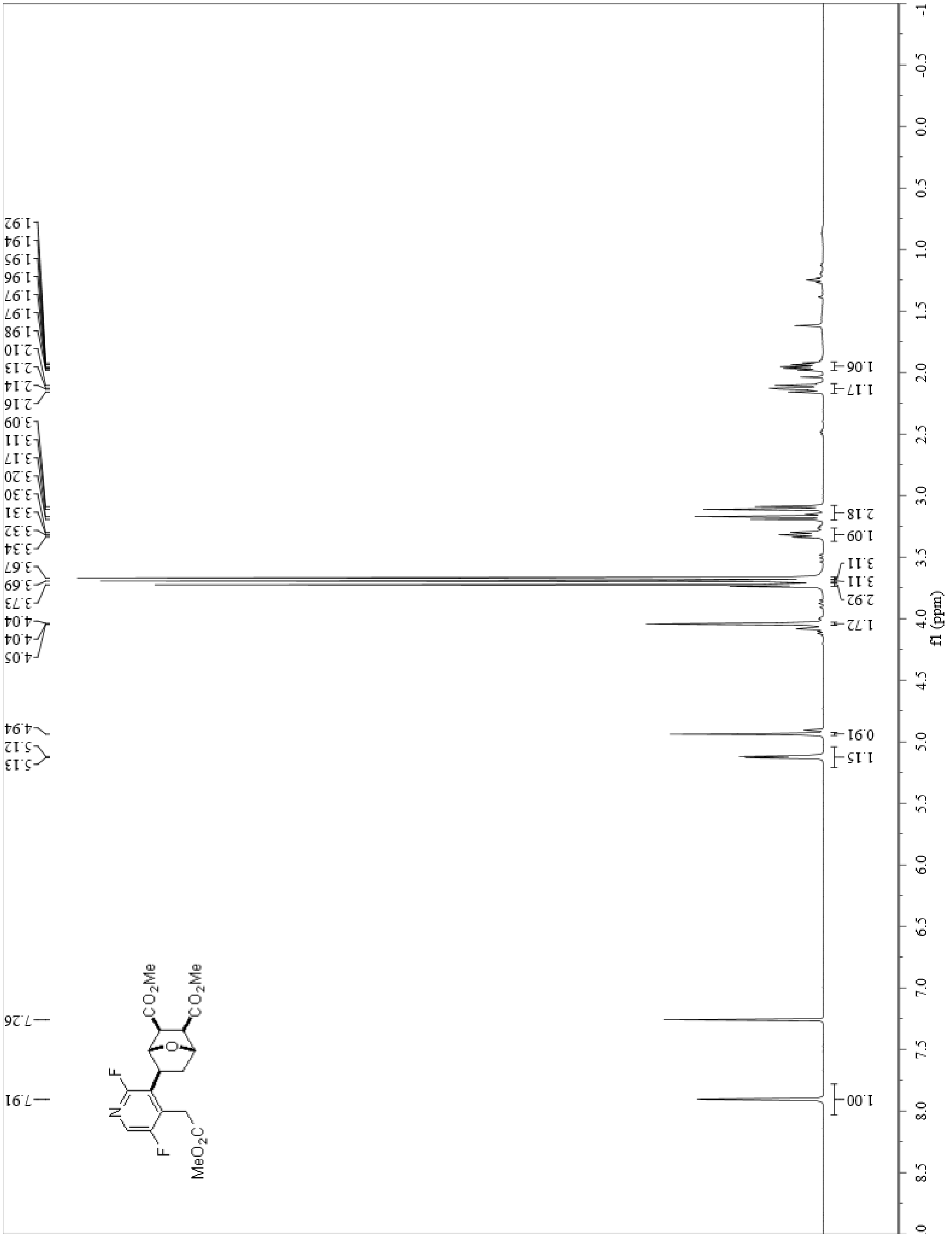

$^{13}C\{^{1}H\}$ NMR (101 MHz, Chloroform-*d*, @ rt) of S-33a dimethyl 5-(2,5-difluoro-4-(2-methoxy-2-oxoethyl)pyridin-3-yl)-7-oxabicyclo[2.2.1]heptane-2,3-dicarboxylate

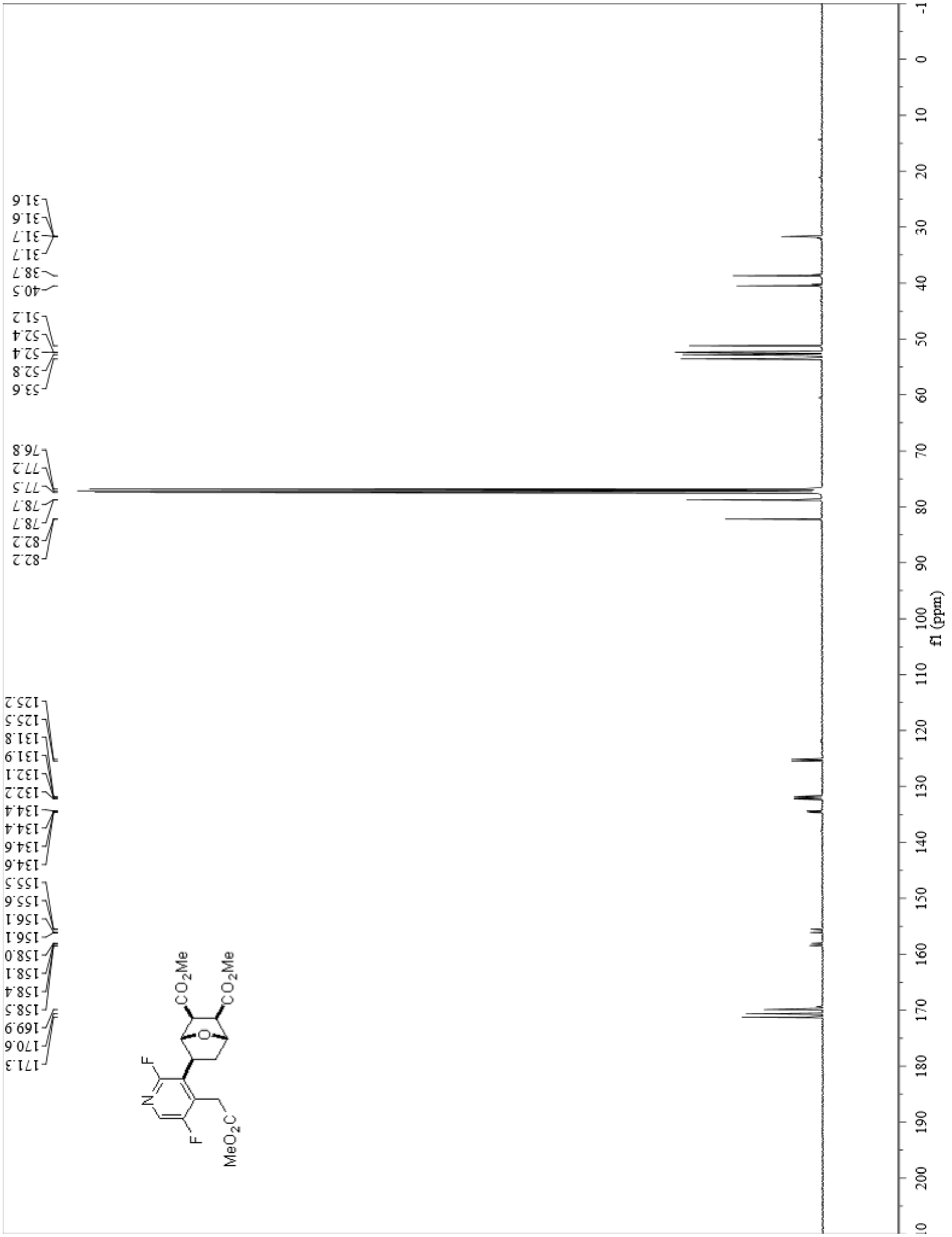

GC and MS of S-33a dimethyl 5-(2,5-difluoro-4-(2-methoxy-2-oxoethyl)pyridin-3-yl)-7-oxabicyclo[2.2.1]heptane-2,3-dicarboxylate

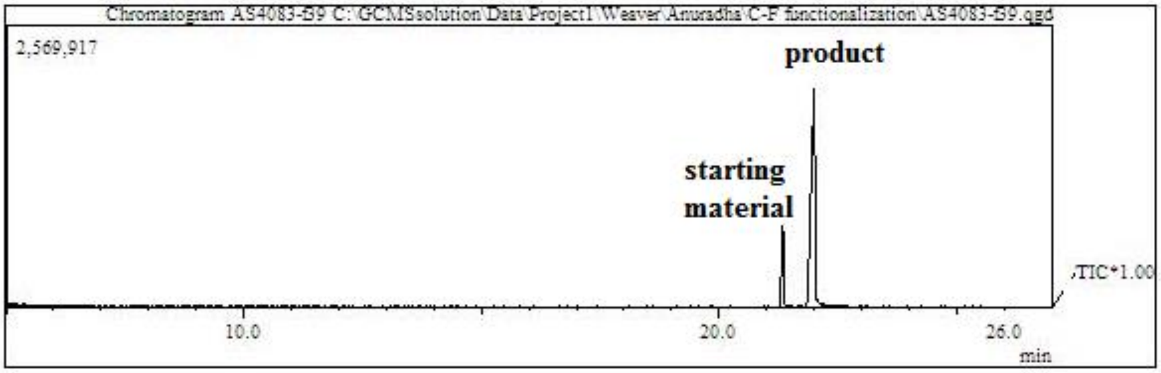

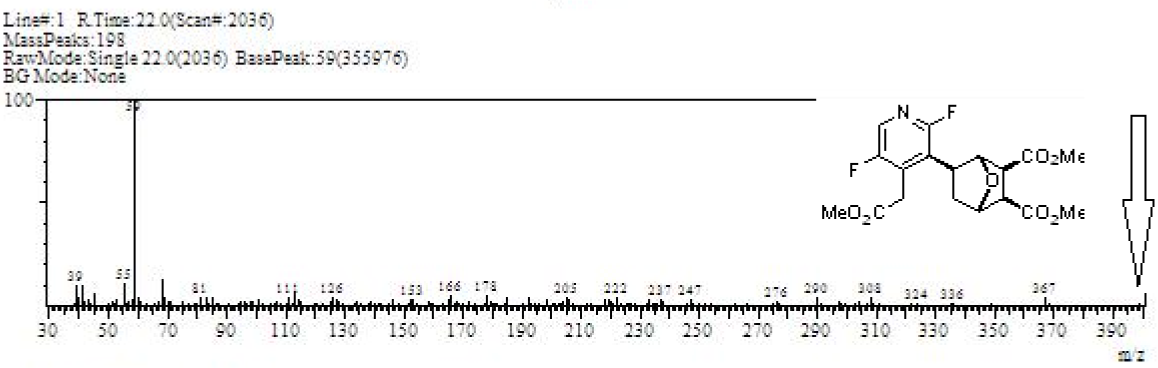

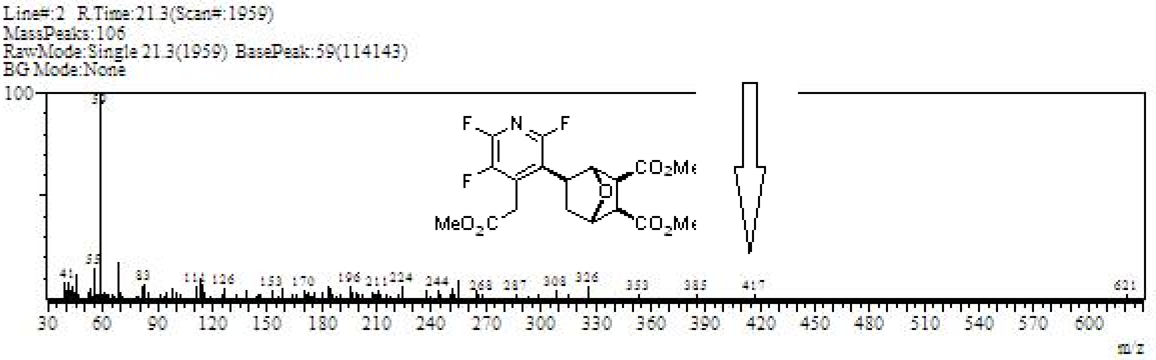

^{19}F NMR (376 MHz, Chloroform-*d*, @ rt) of S-34a methyl 2-(bicyclo[2.2.1]heptan-2-yl)-3,5-difluoro-4-(2-methoxy-2-oxoethyl)benzoate

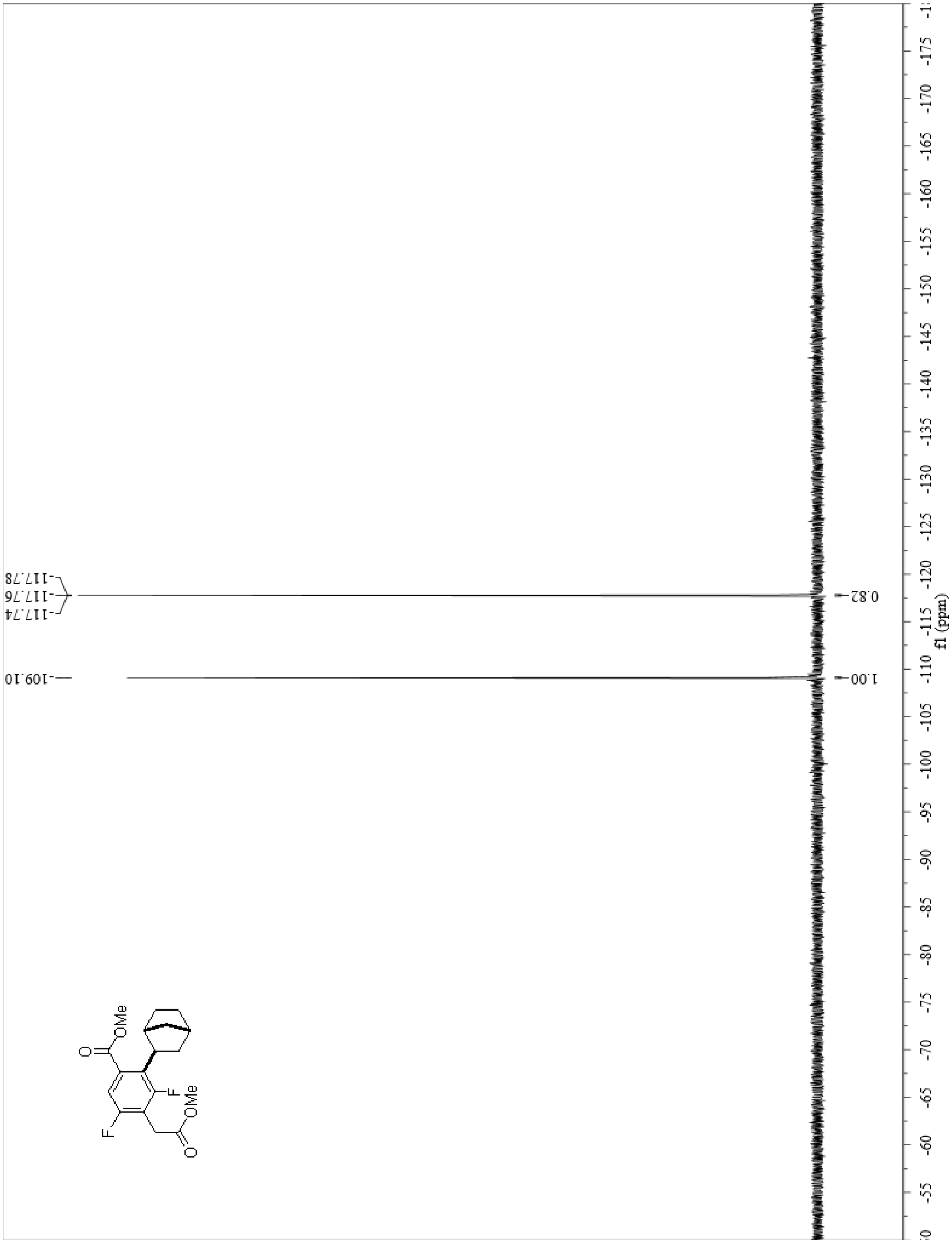

^{1}H NMR (400 MHz, Chloroform-*d*, @ rt) of S-34a methyl 2-(bicyclo[2.2.1]heptan-2-yl)-3,5-difluoro-4-(2-methoxy-2-oxoethyl)benzoate

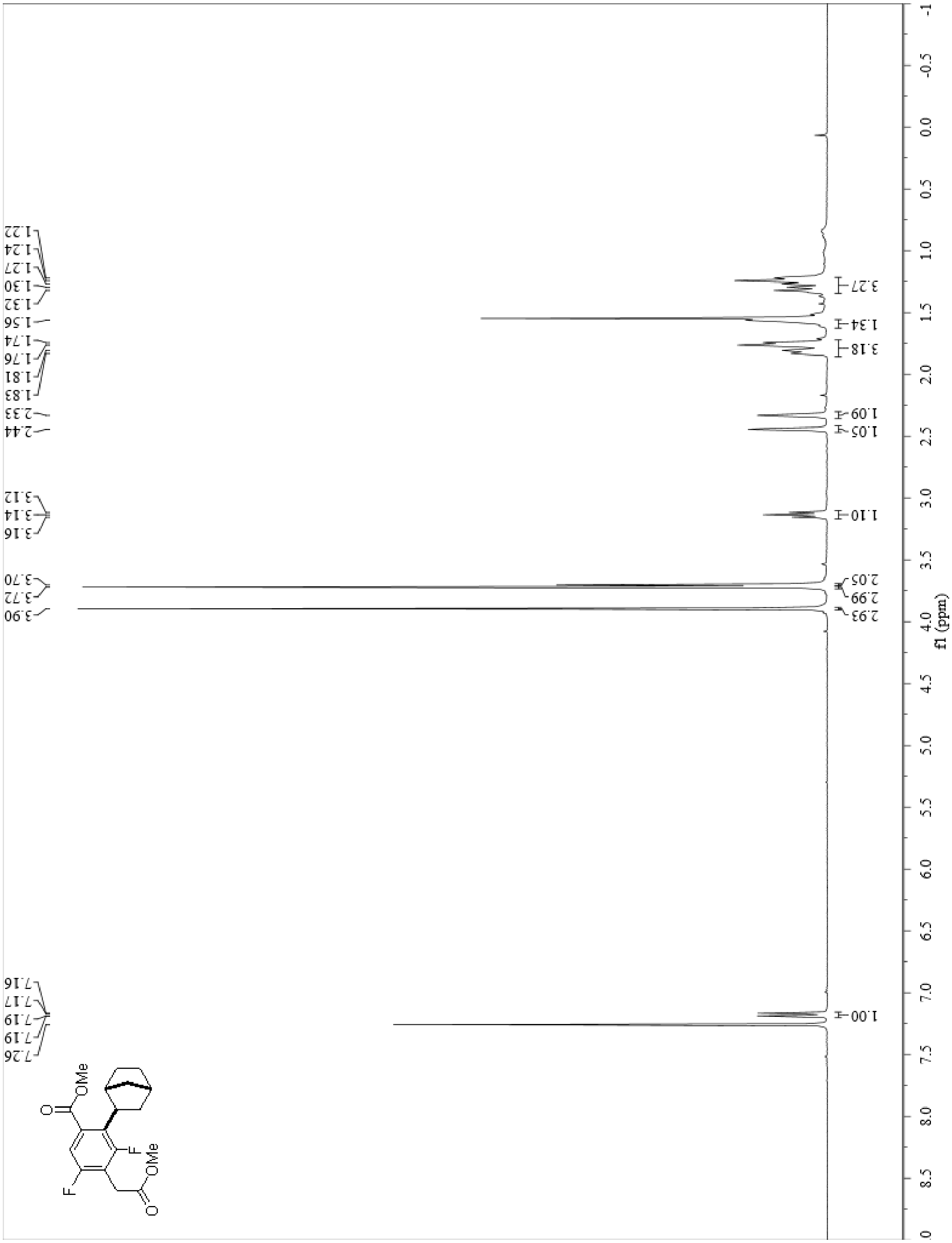

$^{13}C\{^{1}H\}$ NMR (101 MHz, Chloroform-*d*, @ rt) of S-34a methyl 2-(bicyclo[2.2.1]heptan-2-yl)-3,5-difluoro-4-(2-methoxy-2-oxoethyl)benzoate

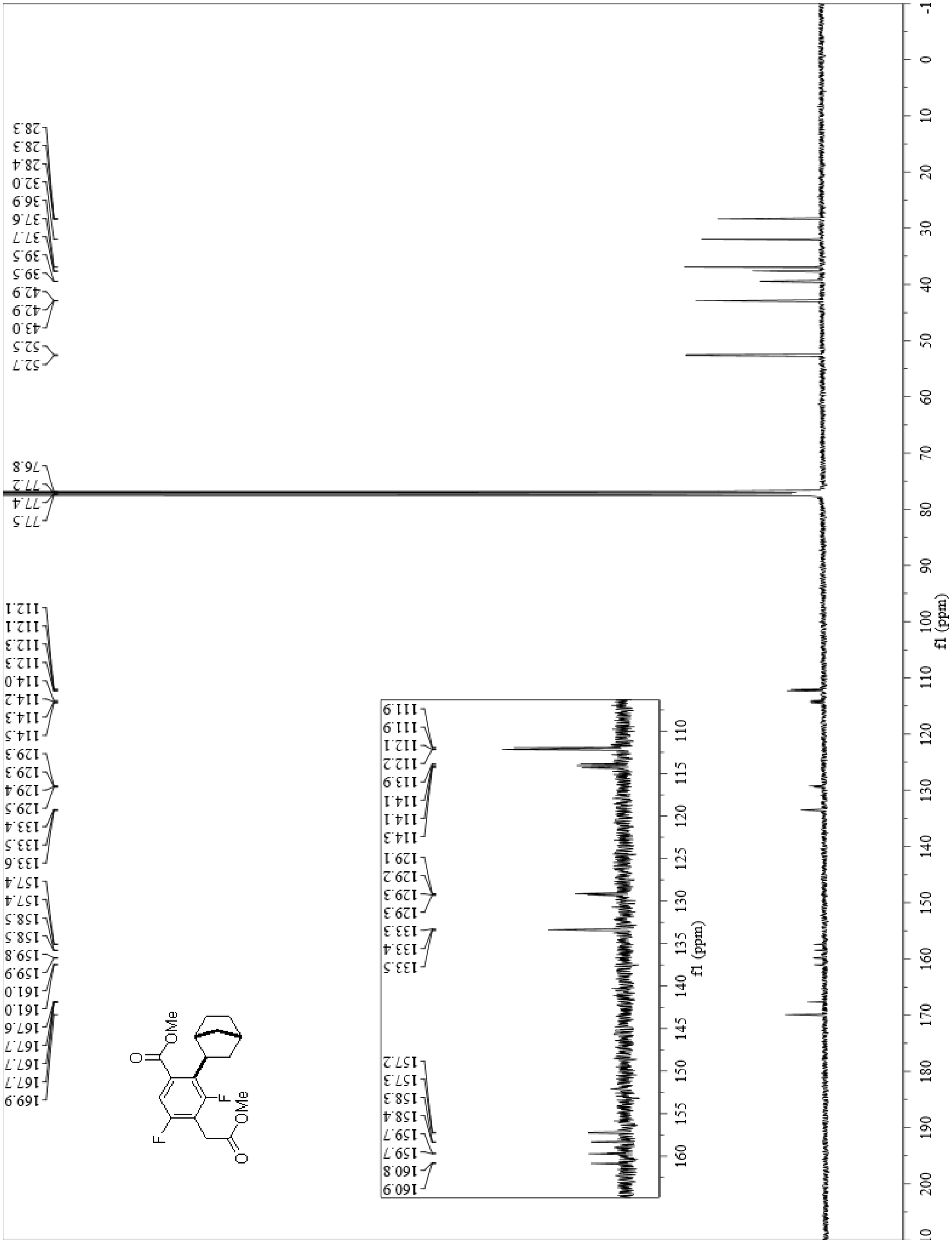

GC and MS of S-34a methyl 2-(bicyclo[2.2.1]heptan-2-yl)-3,5-difluoro-4-(2-methoxy-2-oxoethyl)benzoate

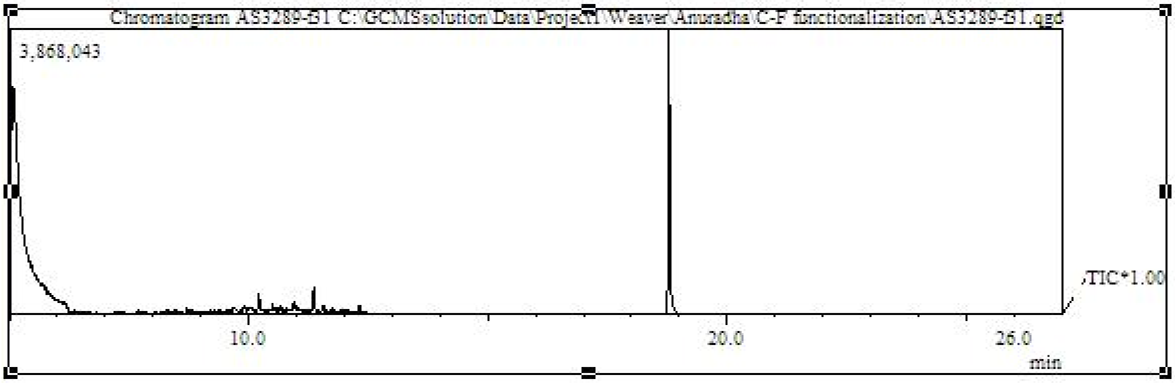

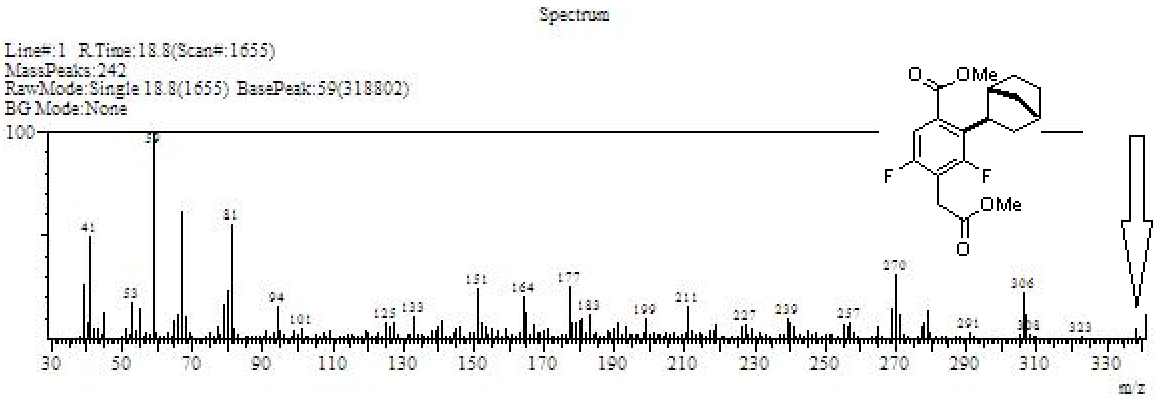

^{19}F NMR (376 MHz, Chloroform-*d*, @ rt) of S-35a N-(3-(bicyclo[2.2.1]heptan-2-yl)-2,6-difluoro-4-(trifluoromethyl)phenyl)acetamide

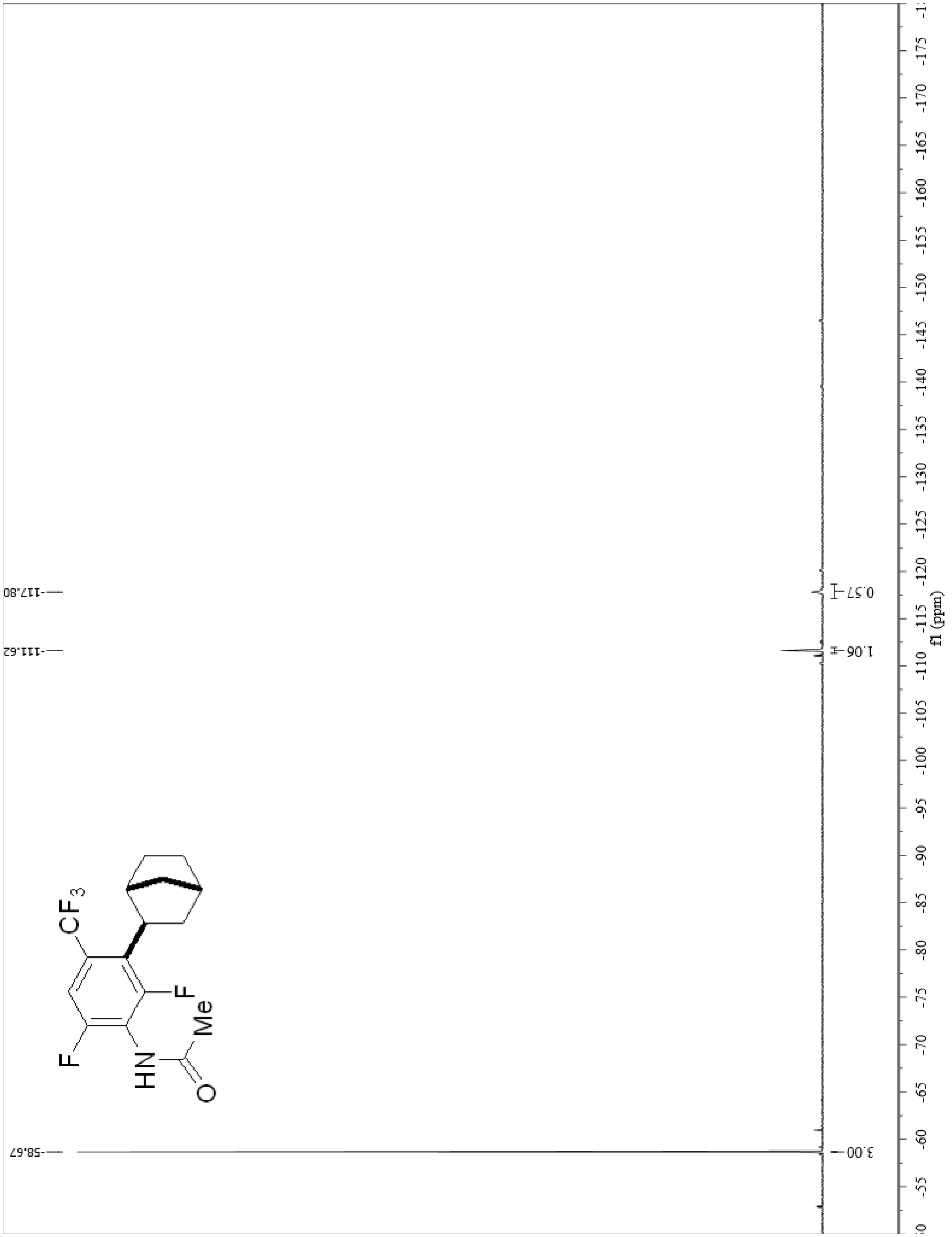

^{1}H NMR (400 MHz, Chloroform-*d*, @ rt) of S-35a N-(3-(bicyclo[2.2.1]heptan-2-yl)-2,6-difluoro-4-(trifluoromethyl)phenyl)acetamide

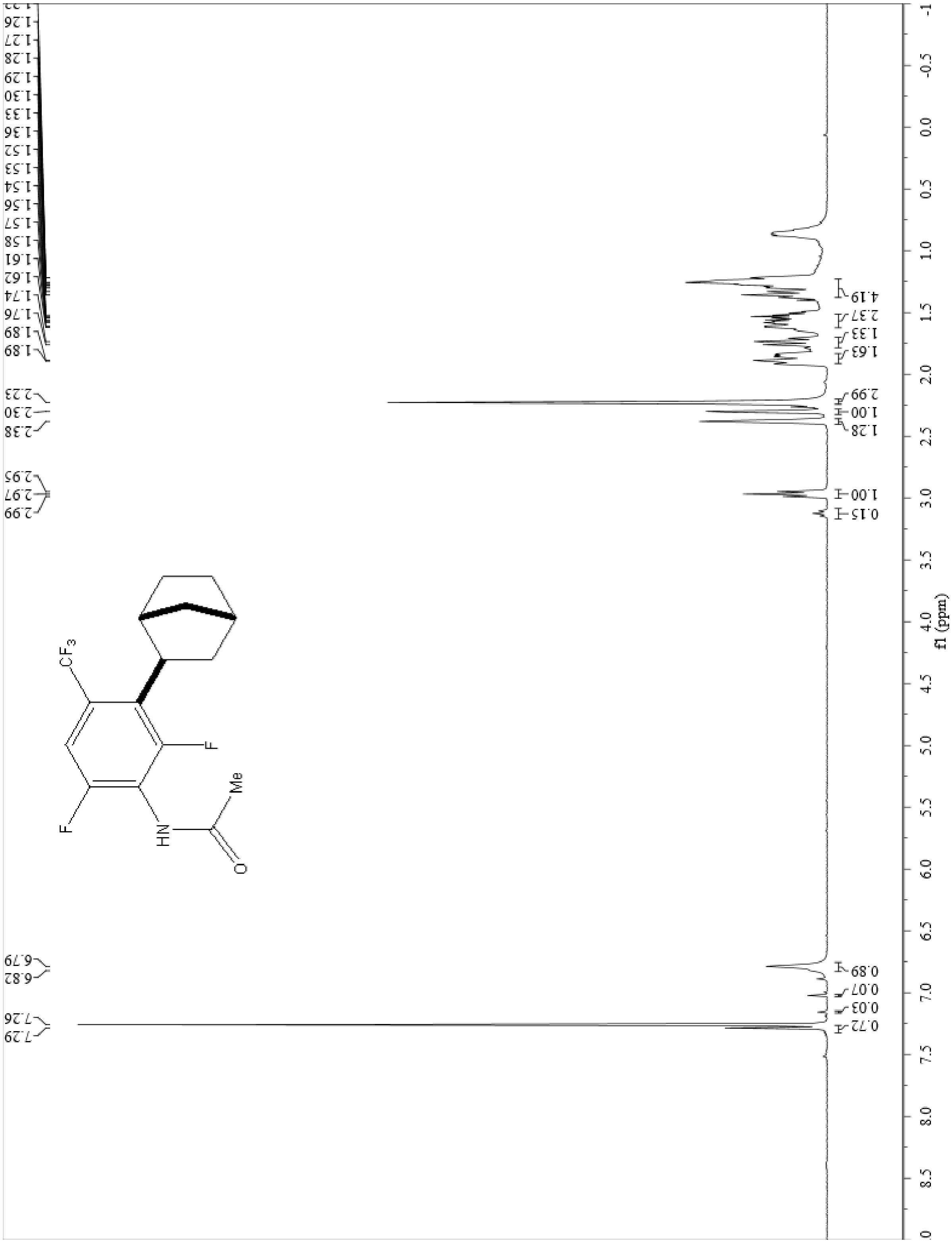

$^{13}C\{^{1}H\}$ NMR (101 MHz, Chloroform-*d*, @ rt) of S-35a N-(3-(bicyclo[2.2.1]heptan-2-yl)-2,6-difluoro-4-(trifluoromethyl)phenyl)acetamide

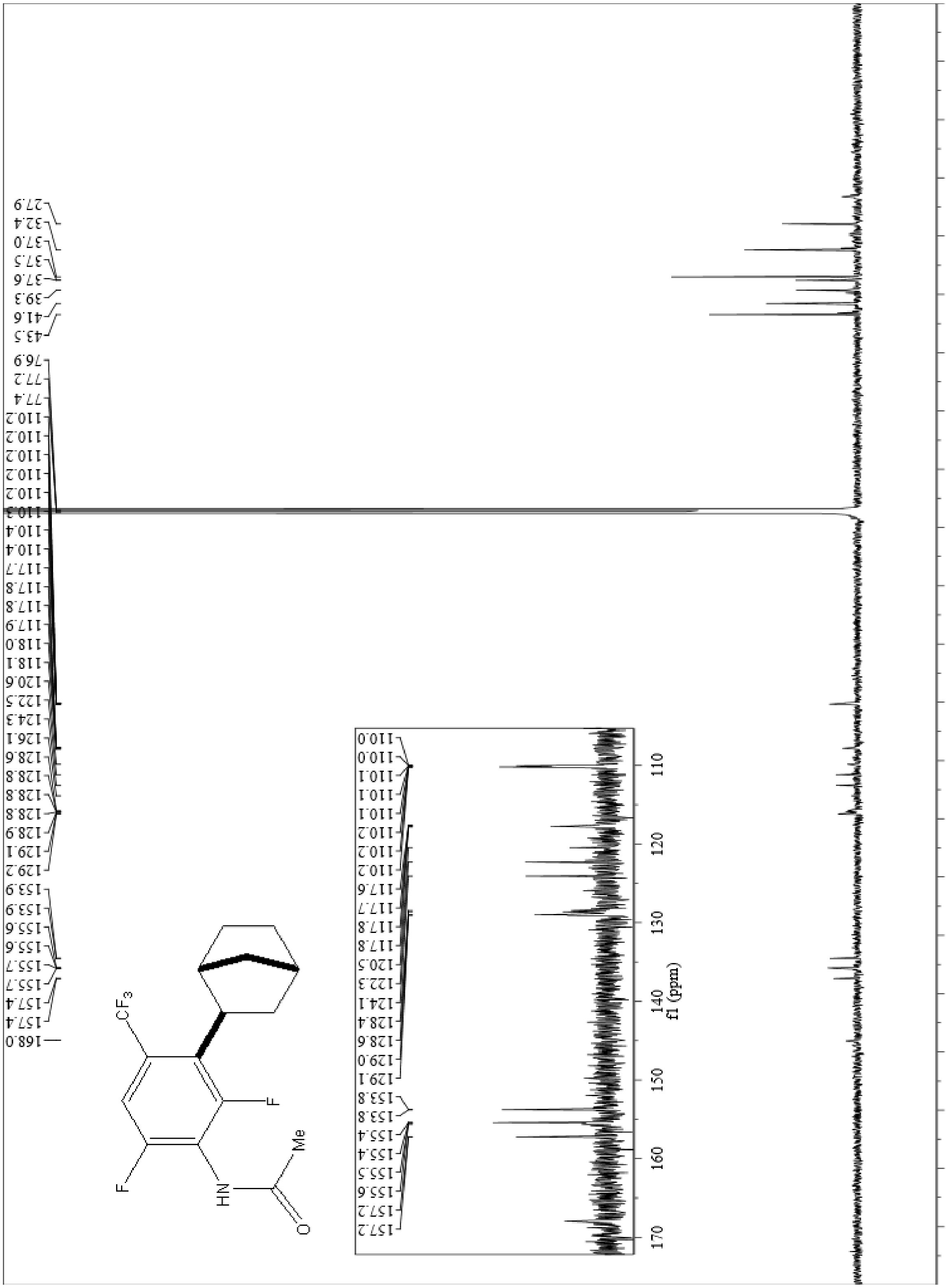

GC and MS of S-35a N-(3-(bicyclo[2.2.1]heptan-2-yl)-2,6-difluoro-4-(trifluoromethyl)phenyl)acetamide

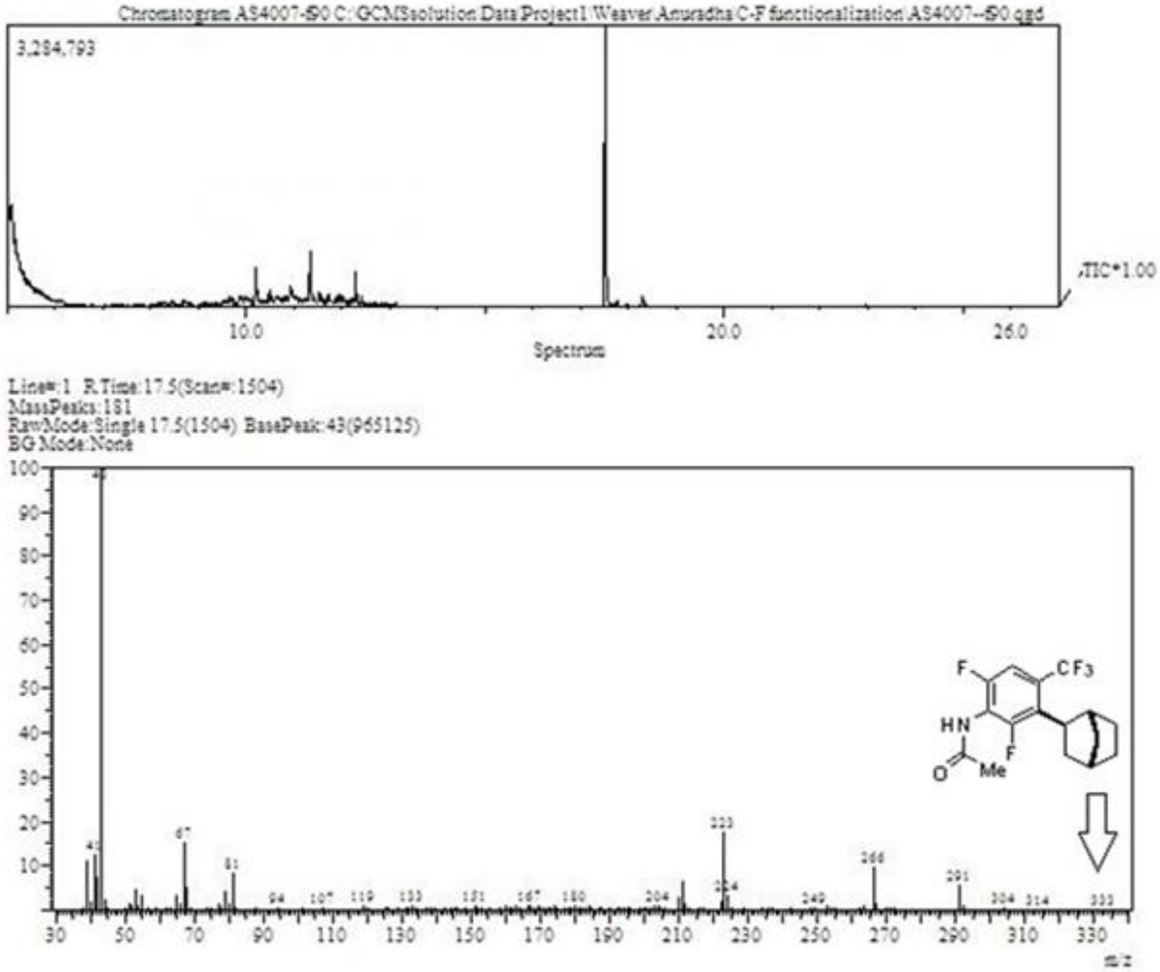

^{19}F NMR (376 MHz, Chloroform-*d*, @ rt) of S-36a' ethyl (2-(3-(bicyclo[2.2.1]heptan-2-yl)-2,5,6-trifluoro-4-(trifluoromethyl)phenyl)acetyl)glycinate

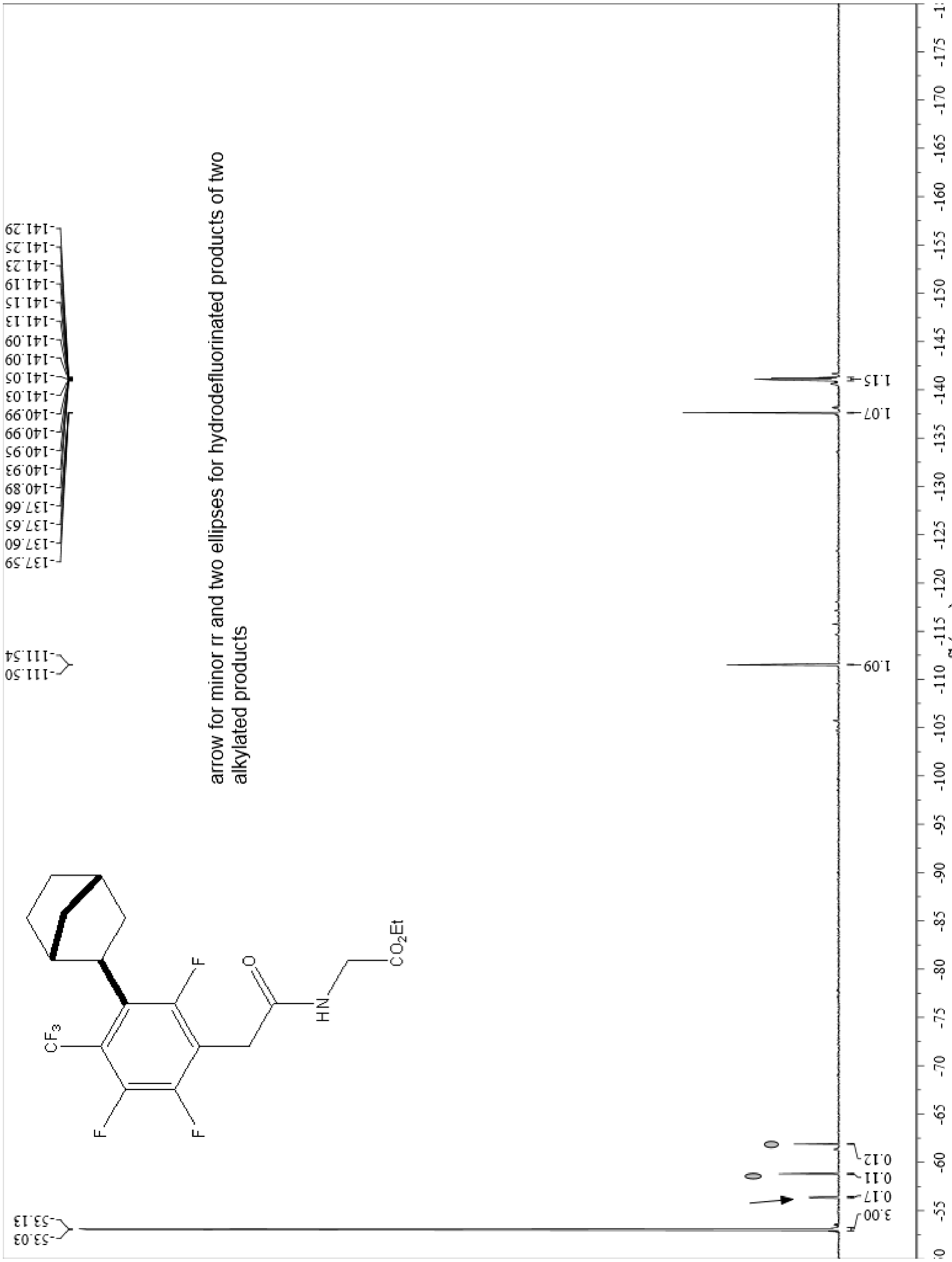

^{1}H NMR (400 MHz, Chloroform-*d*, @ rt) of S-36a' ethyl (2-(3-(bicyclo[2.2.1]heptan-2-yl)-2,5,6-trifluoro-4-(trifluoromethyl)phenyl)acetyl)glycinate

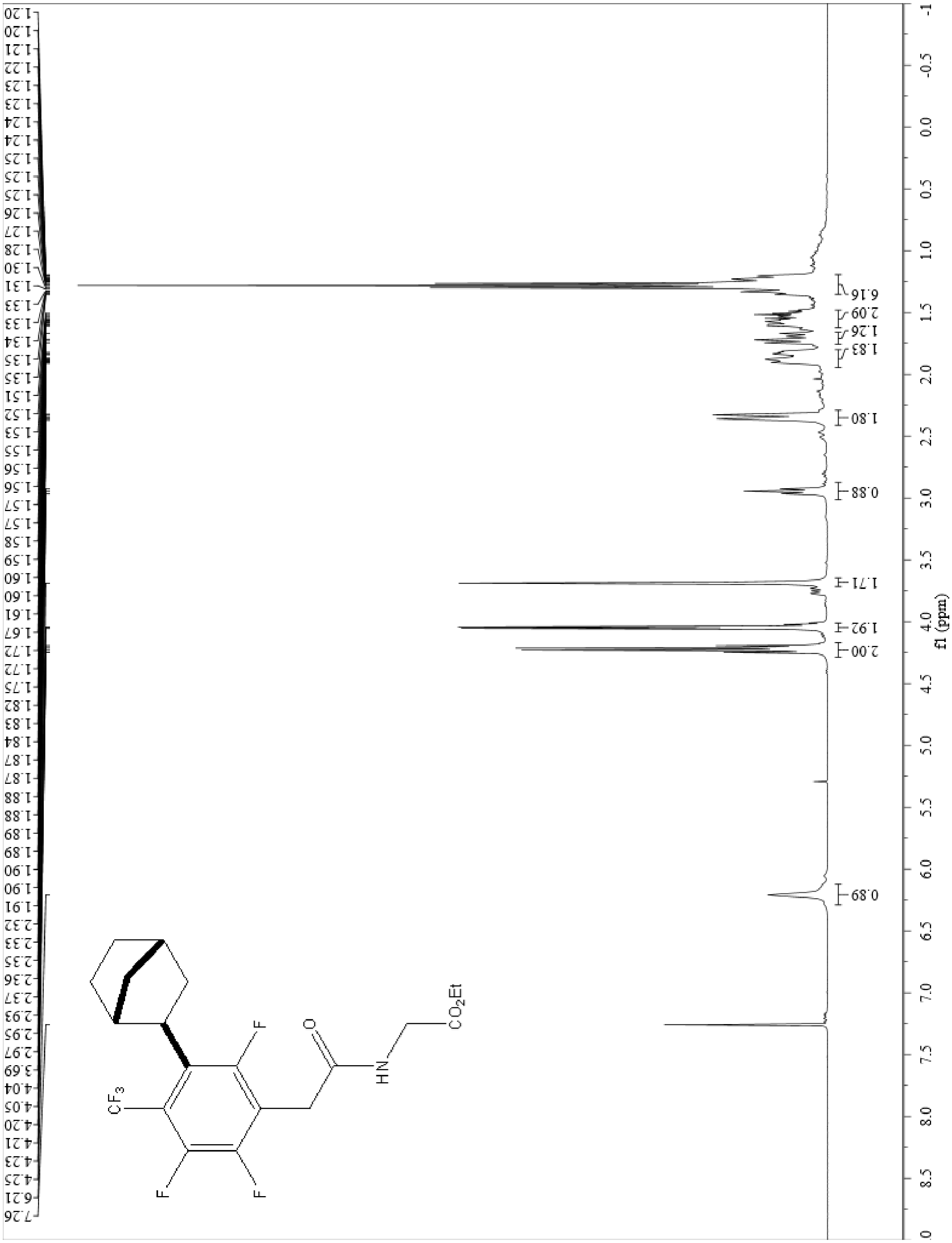

$^{13}C\{^1H\}$ NMR (101 MHz, Chloroform-*d*, @ rt) of S-36a' ethyl (2-(3-(bicyclo[2.2.1]heptan-2-yl)-2,5,6-trifluoro-4-(trifluoromethyl)phenyl)acetyl)glycinate

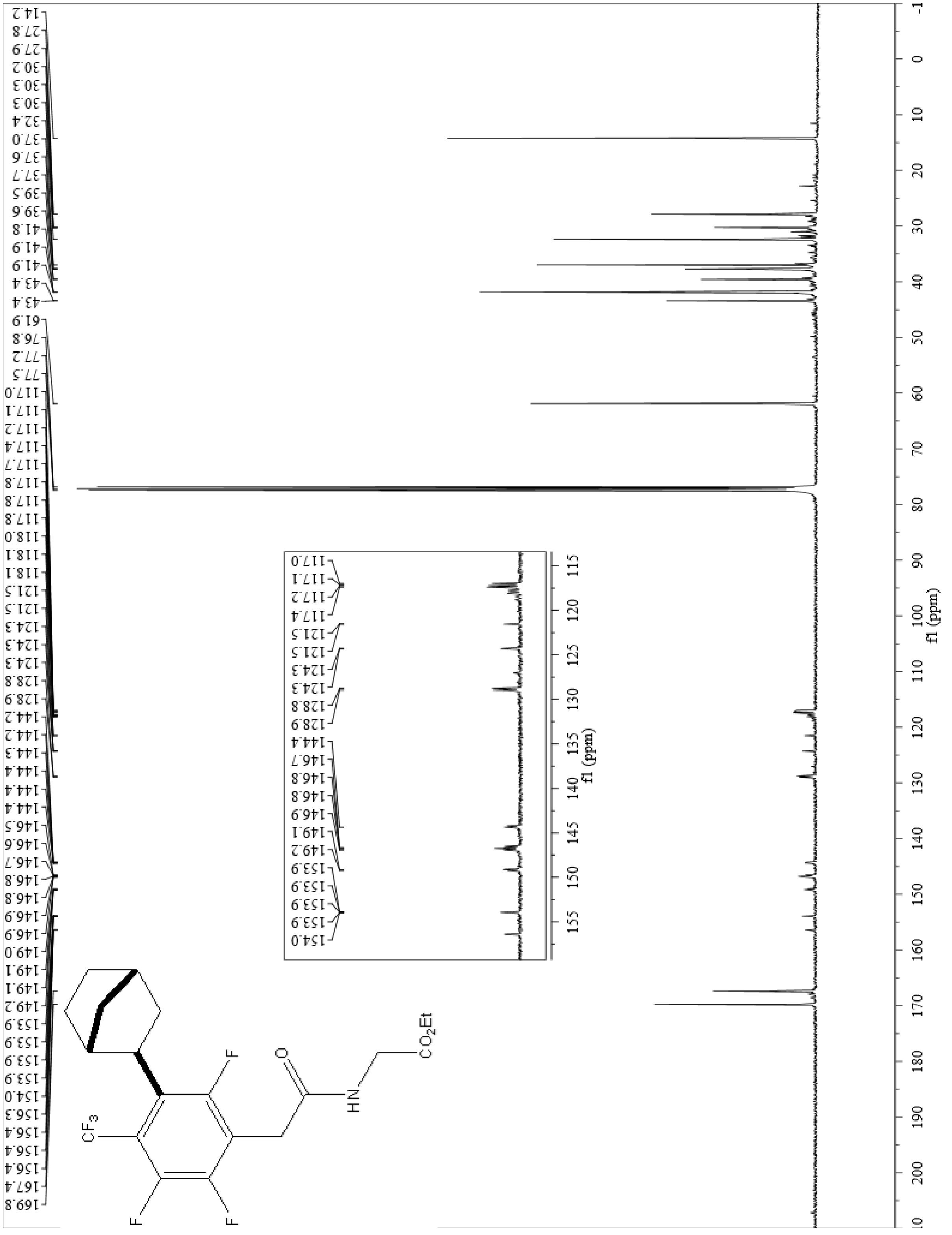

GC and MS of S-36a' ethyl (2-(3-(bicyclo[2.2.1]heptan-2-yl)-2,5,6-trifluoro-4-(trifluoromethyl)phenyl)acetyl)glycinate

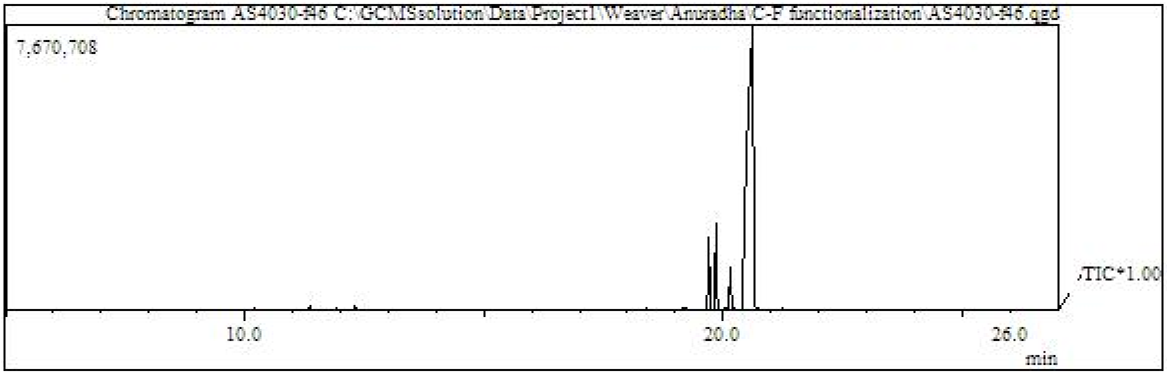

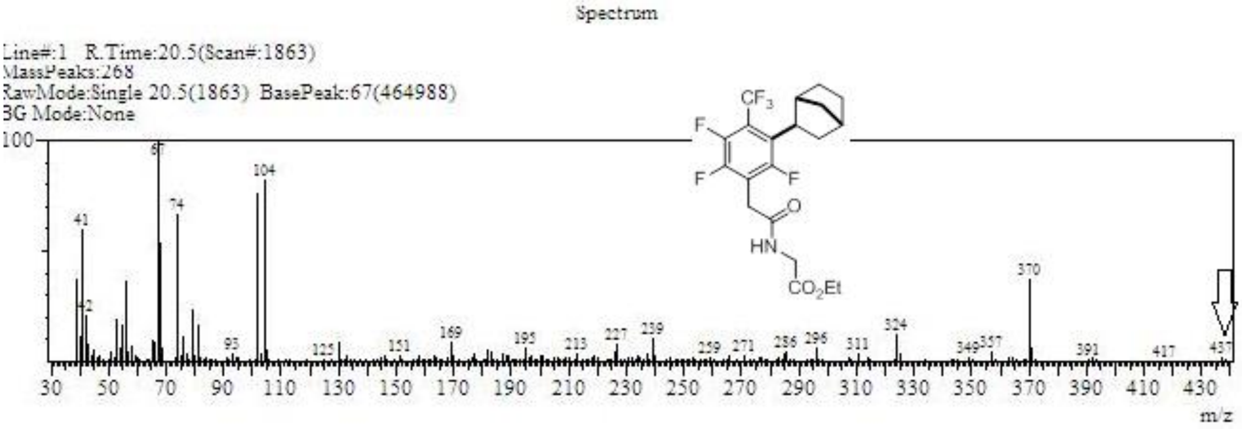

Spectrum

Line#:1 R.Time:20.2(Scan#:1820)
MassPeaks:163
RawMode:Single 20.2(1820) BasePeak:104(150754)
BG Mode:None

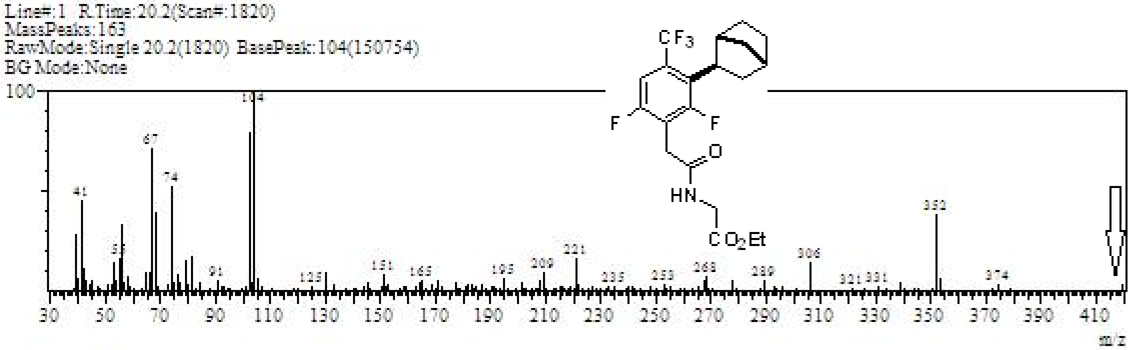

Line#:2 R.Time:19.9(Scan#:1786)
MassPeaks:96
RawMode:Single 19.9(1786) BasePeak:104(56190)
BG Mode:None

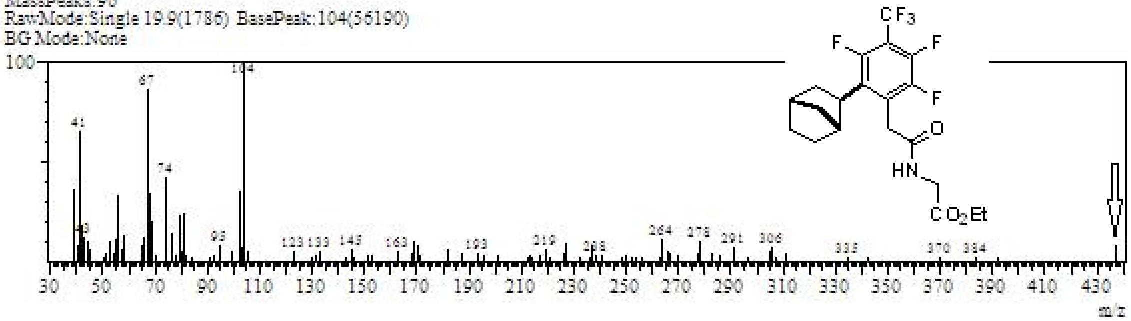

Line#:3 R.Time:19.7(Scan#:1766)
MassPeaks:157
RawMode:Single 19.7(1766) BasePeak:104(163665)
BG Mode:None

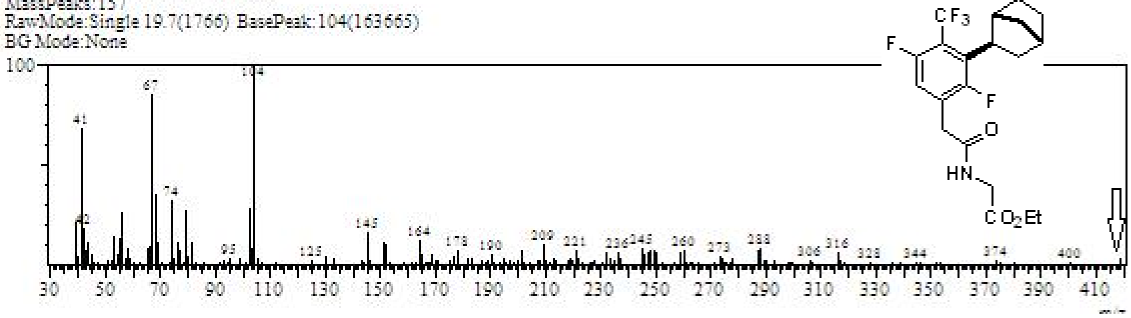

^{19}F NMR (376 MHz, Chloroform-*d*, @ rt) of S-36a ethyl (2-(3-(bicyclo[2.2.1]heptan-2-yl)-2,5,6-trifluoro-4-(trifluoromethyl)phenyl)acetyl)glycinate

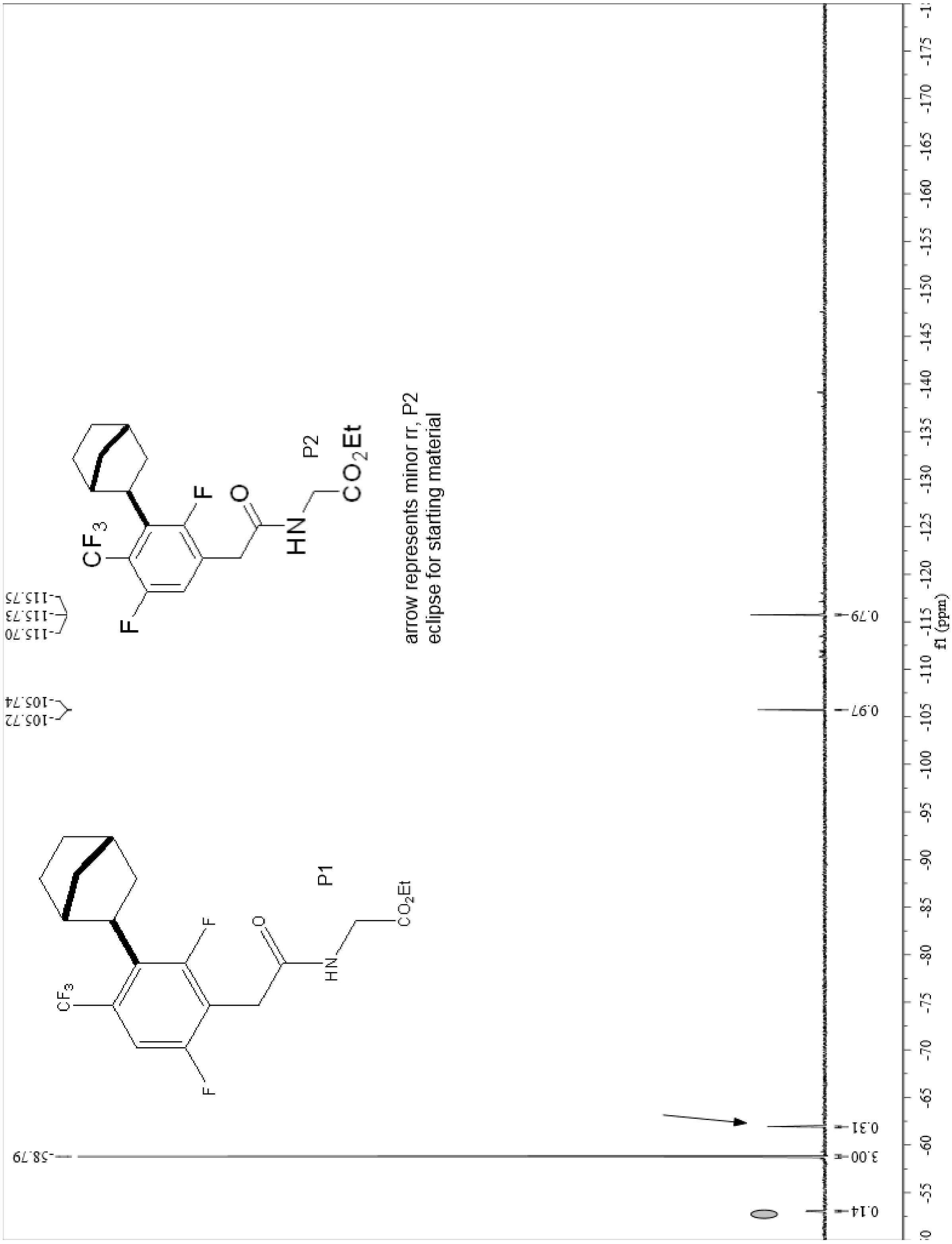

^{1}H NMR (400 MHz, Chloroform-*d*, @ rt) of S-36a ethyl (2-(3-(bicyclo[2.2.1]heptan-2-yl)-2,5,6-trifluoro-4-(trifluoromethyl)phenyl)acetyl)glycinate

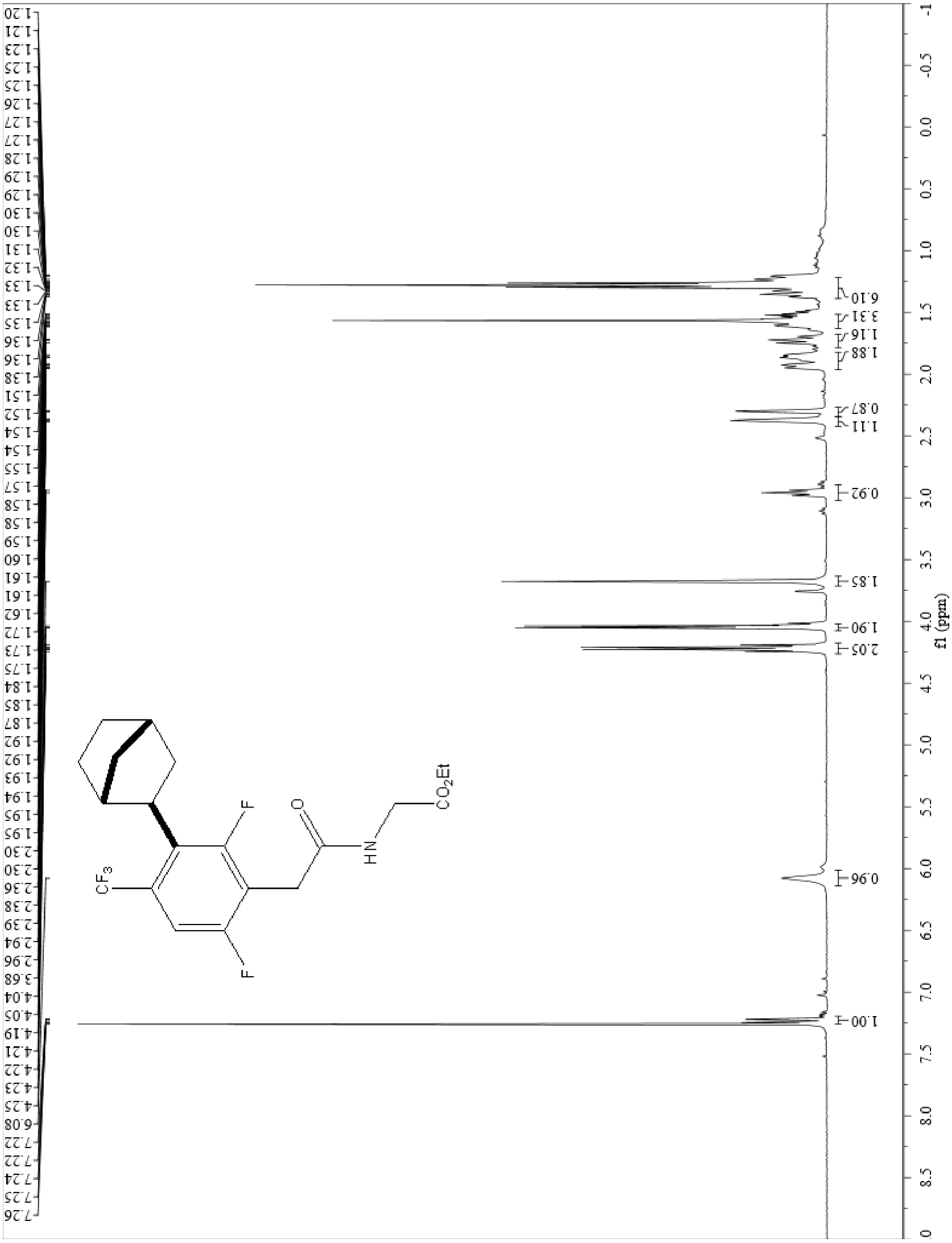

$^{13}C\{^1H\}$ NMR (151 MHz, Chloroform-*d*, @ rt) of S-36a ethyl (2-(3-(bicyclo[2.2.1]heptan-2-yl)-2,5,6-trifluoro-4-(trifluoromethyl)phenyl)acetyl)glycinate

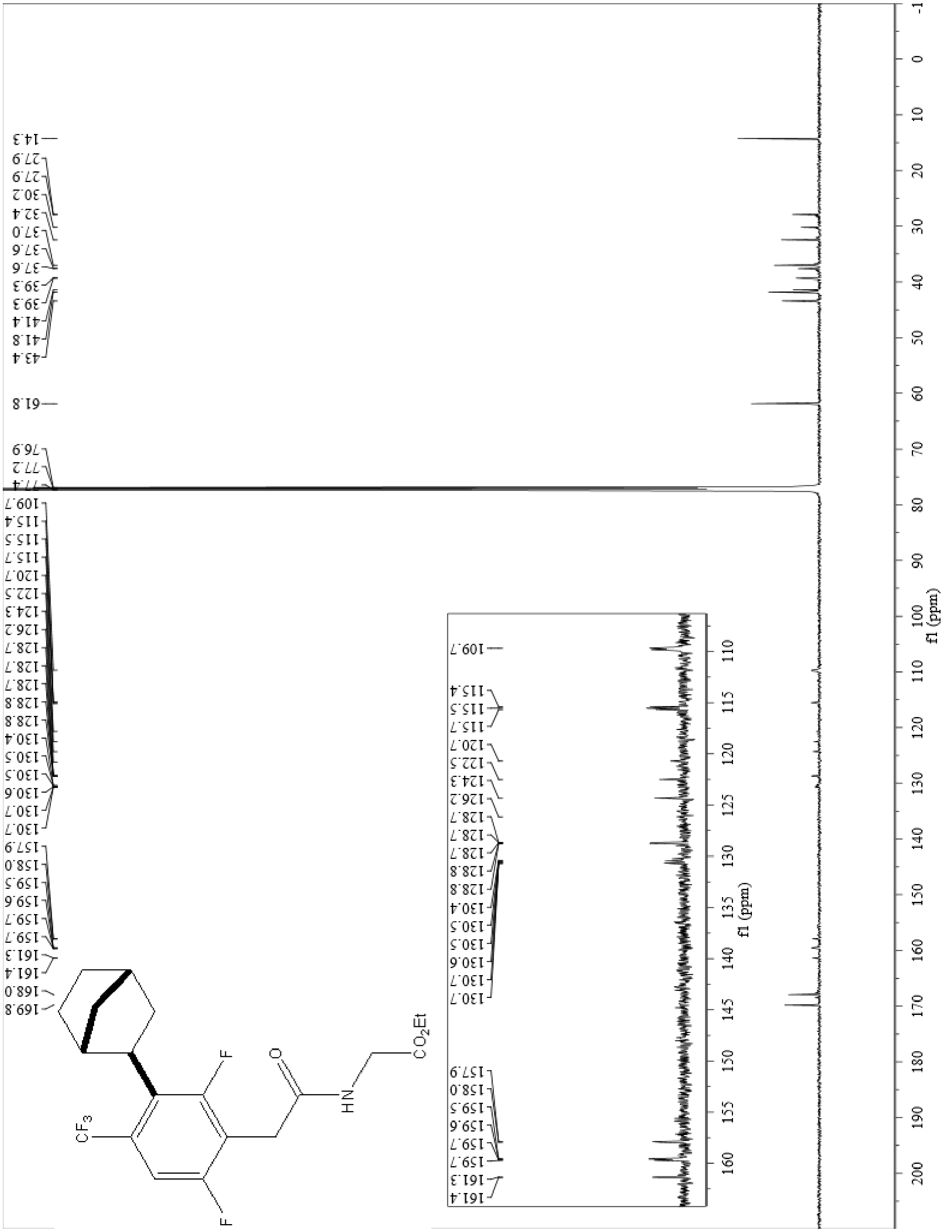

GC and MS of S-36a ethyl (2-(3-(bicyclo[2.2.1]heptan-2-yl)-2,5,6-trifluoro-4-(trifluoromethyl)phenyl)acetyl)glycinate

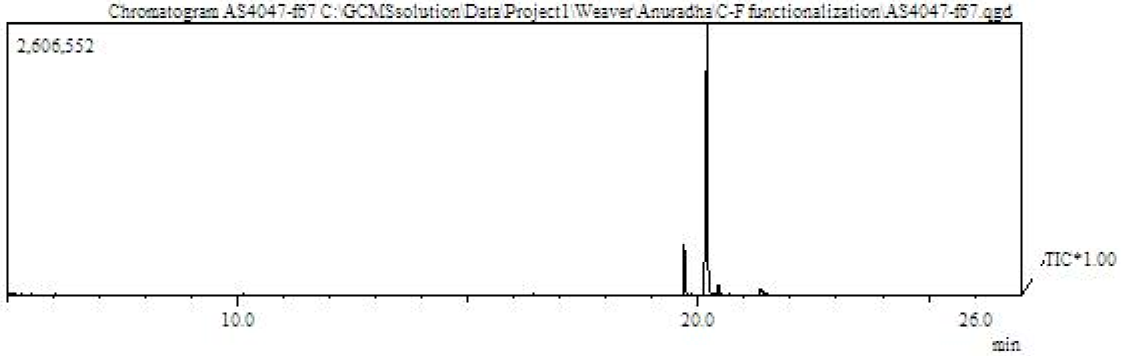

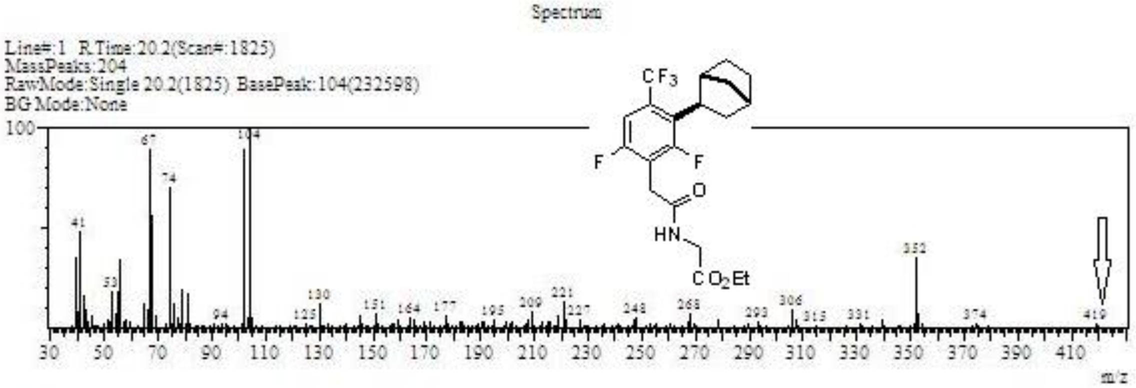

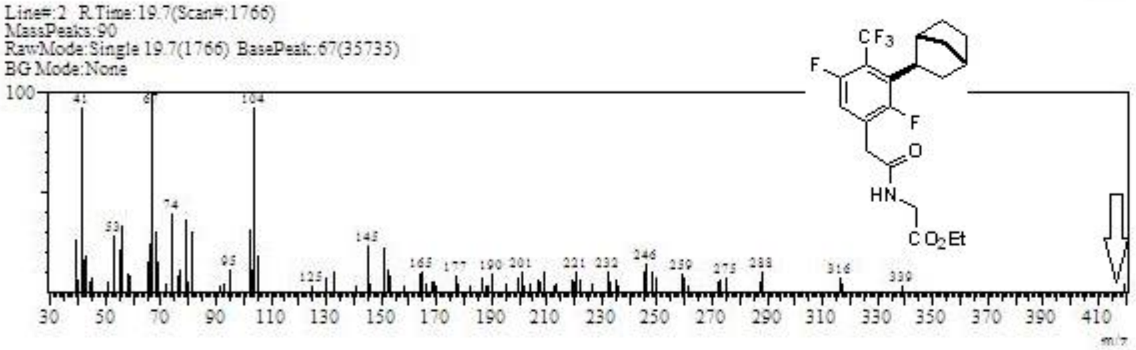

Supplement: Supplementary file 1 [file SC-006-C5SC03013G-s001.pdf]
